# Efforts toward the Total Synthesis of Elisabethin A

**DOI:** 10.1021/acs.joc.2c01914

**Published:** 2022-10-25

**Authors:** Maximilian Kaiser, David Schönbauer, Katharina Schragl, Matthias Weil, Peter Gaertner, Valentin S. Enev

**Affiliations:** † Institute of Applied Synthetic Chemistry, TU Wien, Getreidemarkt 9/163, 1060Wien, Austria; ‡ Institute of Chemical Technologies and Analytics, TU Wien, Getreidemarkt 9/164, 1060Wien, Austria

## Abstract

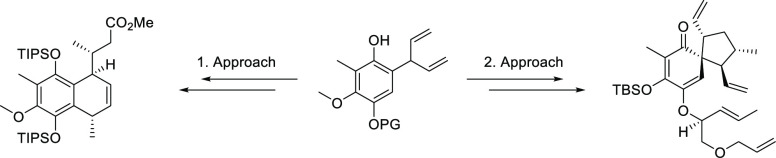

We describe our efforts toward the total synthesis of the natural product elisabethin A. The first route was guided by the proposed biosynthesis, assembling the 6,6-ring system before forming the five-membered ring including the quaternary carbon. The second approach includes a high yielding cyclization under Mitsunobu conditions as a key step. It allowed the preparation of an unusual and highly functionalized bicyclic 6,5-spiro compound. Both routes share a common advanced precursor obtained from an “underdeveloped” Claisen rearrangement of an aryl dienyl ether.

## Introduction

**Figure 1:**
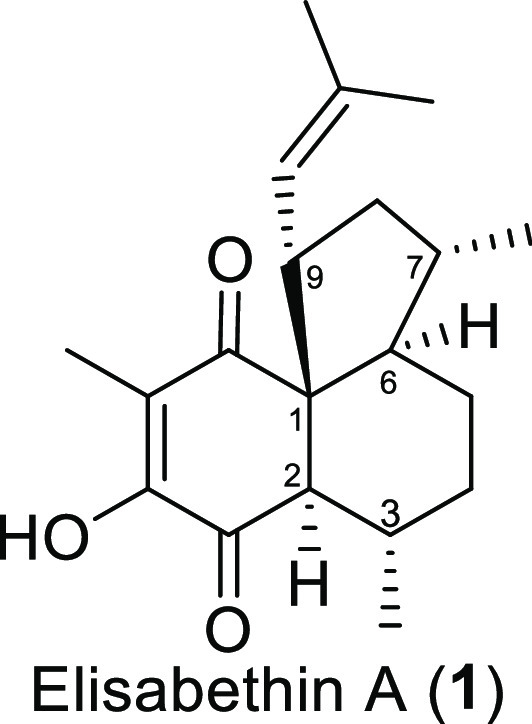
Structure of elisabethin A.

Elisabethin A (**1**) was discovered in 1998 as the first member of the novel elisabethane family.^1^ It was isolated from the Caribbean gorgonian *Pseudopterogorgia elisabethae* harvested in the sea near San Andrés Island, Colombia (Figure 1). From approximately 1 kg of dried animal, 25 mg of elisabethin A (**1**) was isolated as the crystalline material. 2D-NMR analysis and X-ray crystallography allowed the determination of the relative configuration, whereas the absolute configuration remains unclear. This diterpene with the molecular formula C_20_H_28_O_3_ consists of a tricyclic *cis*-*trans*-fused 5,6,6-ring system. It bears six contiguous stereocenters, namely, quaternary all-carbon spiro centers C-1, C-2, C-3, C-6, C-7, and C-9. An additional feature is the highly oxidized and fully substituted enedione moiety.

**Figure 2:**
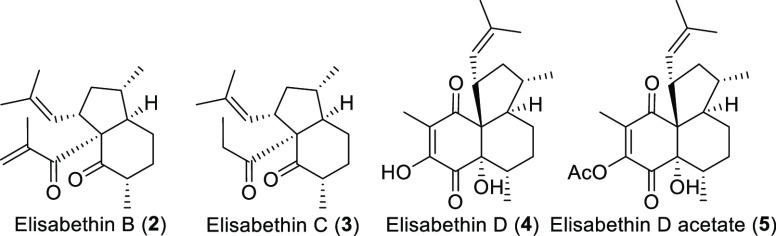
Members of the elisabethane family.

From the same marine organism, various novel metabolites were isolated, namely, elisabethin B (**2**), elisabethin C (**3**), elisabethin D (**4**), and its corresponding acetate (**5**) (Figure 2).^1,2^

Additionally, *P. elisabethae* was the source for elisapterosin B (**6**) and tetracyclic colombiasin A (**7**).^2,3^ Their novel carbon framework paired with the high density of stereochemical information has made these two molecules attractive targets for total synthesis during the past two decades.^4−7^

**Figure 3:**
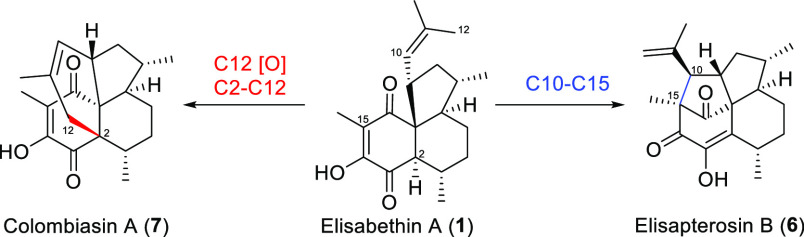
Precursor role of elisabethin A (**1**).

It is speculated that the two latter natural products could be synthesized from elisabethin A (**1**) as their common precursor (Figure 3). Elisapterosin B (**6**) might be prepared *via* C10–C15 bond formation, whereas colombiasin A (**7**) could be accessible *via* C12 oxidation and subsequent connection to C2. Despite this potential, none of the published total syntheses relied on this elisabethin A precursor strategy. In 2003, Rawal et al. demonstrated the feasibility of such a possible interconversion in their intended (***ent***)-elisabethin A (**1**) synthesis.^8^

While being met with the unsolvable issue of C-2 epimerization (elisabethin A numbering throughout), the synthesis was rerouted to access (***ent***)-elisapterosin B. This allowed the confirmation of the stereochemical assignment of key intermediates prepared during this approach. In 2003, Mulzer reported the successful completion of the total synthesis of elisabethin A (**1**). Interestingly, Rawal and Mulzer shared a common intermediate despite being the opposite enantiomer. In Rawal et al.’s case, the C-2 epimerization was reported to be infeasible, whereas Mulzer and Heckrodt claimed a successful transformation of their antipode. This discrepancy and the fact that the spectra of the synthesized and natural materials were not superimposable soon gave rise to considerable doubt with respect to the stereochemical assignment of their final compound.^9,10^ In 2014, Mulzer et al. reported a second approach to elisabethin A (**1**) but was not able to prove their claims made in 2003.^11^

Herein, we wish to report two different approaches toward the total synthesis of elisabethin A (**1**). Both were running in parallel with the idea to push forward with the most promising approach to completion.

## Results and Discussion

### First Approach

**Scheme 1:**
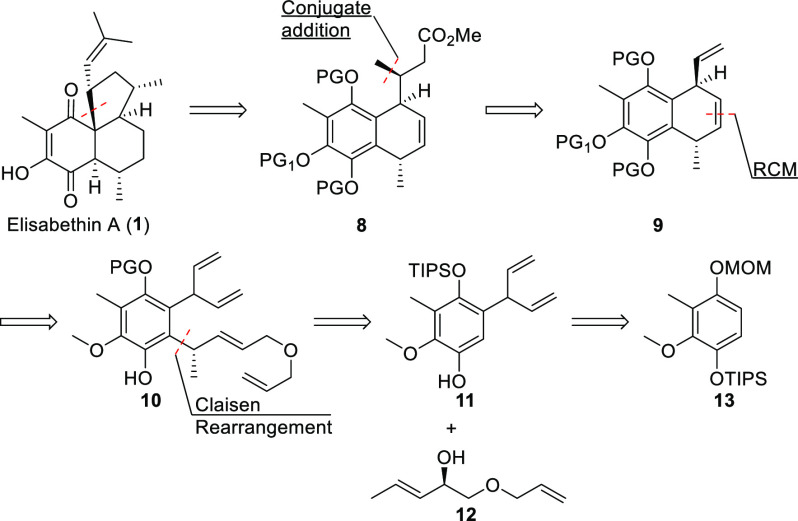
Retrosynthetic Analysis of Elisabethin A (**1**)

Our first approach was strongly guided by preliminary results.^12,13^ Accordingly, target molecule **1** could be traced back to bicyclic ester **8** (Scheme 1). Introduction of the C-7 methyl group was planned to be achievable by conjugate addition to the corresponding α,β-unsaturated ester. Further simplification by means of functional group manipulation would lead to compound **9**. Although our first-generation synthesis of the precursor for bicyclic intermediate **9** was successful, the lack of selectivity prompted us to investigate a new strategy to prepare this material. The idea was to perform desymmetrization in the course of RCM reaction of **10**, either under a substrate control or by using chiral catalysis.^14,15^

Further disconnection in this second-generation approach allowed the identification of two building blocks, chiral alcohol **12** and phenol **11**, which was derived from compound **13**.^12^ The first issue to overcome in this approach was whether compound **11** could be synthesized with reasonable effort.

**Scheme 2:**
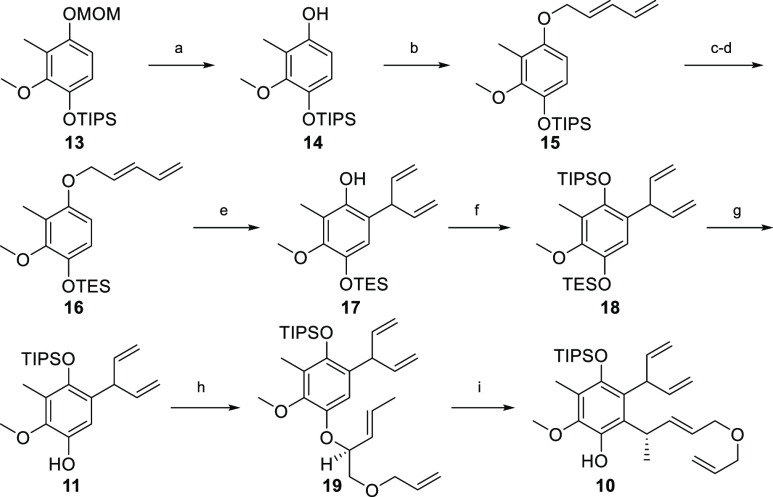
Synthesis of Compound **10** Reagents and conditions: (a) ZnBr_2_, EtSH, DCM, −32 to −5 °C, 2.5 h, 89%; (b) *n*-BuLi, (*E*)-5-bromopenta-1,3-diene, 1.4:1 THF/DMF, −35 °C to rt, 15 h, 97%; (c) TBAF, THF, rt, 2 min, 95%; (d) TESCl, imidazole, DCM, rt, 20 min, 95%; (e) EuFOD, PhMe, 110 °C, 4 h, 84%; (f) *n*-BuLi, TIPSOTf, THF, −85 to −45 °C, 1.5 h, 92%; (g) TFA, 4:1 THF/H_2_O, rt, 30 min, 93%; (h) PBu_3_, ADDP, **12**, PhMe, 0 °C to rt, 2 h, 69%; (i) EuFOD, *o*-xylene, 120 °C, 18 h, 55% (+31% **11**).

The synthesis commenced with a large-scale preparation of orthogonally protected hydroquinone **13** from commercially available vanillin (see Experimental Section). Selective cleavage of the MOM group^16^ followed by merging with (*E*)-5-bromopenta-1,3-diene^17^ under basic conditions gave rise to dienyl-ether **15** in 97% yield (Scheme 2).^18^ The following two-step protecting group manipulation produced compound **16** in 90% yield over two steps. This exchange demands some comments. First, the preparation of compound **13** from vanillin was not feasible with OTES protection. Second, TIPS protection of the upper phenolic OH was found to be critical in the later course of the synthesis (see below). Therefore, the lower OTIPS in compound **15** needed to be exchanged to an orthogonal protecting group that could be easily cleaved in the presence of the upper TIPS group.

With compound **16** at hand, the stage was set for the first Claisen rearrangement. After intensive investigations, we found that 5 mol % EuFOD in hot toluene triggered a clean rearrangement of **16**, giving the desired phenol **17** as a single product in 84% yield. This protocol demonstrated a considerable improvement compared to the current state-of-the-art literature,^19−21^ allowing the selective preparation of the desired compound in the multigram scale.

Protection of **17** as a TIPS ether (*n*-BuLi and TIPSOTf) followed by selective cleavage of the TES group afforded phenol **11** in 86% yield over two steps. The next stage called for a substrate-controlled C–O/C-C chirality transfer to install a tertiary stereogenic center in the key intermediate **10**. Accordingly, phenol **11** was coupled with alcohol **12** under Mitsunobu conditions (Bu_3_P and ADDP) and the resultant allyl ether **19** was subjected to a second EuFOD-catalyzed Claisen rearrangement to give phenol **10** in 55% yield.^22,23^ This allowed us to proceed with the second key transformation–diastereosective construction of the cyclohexene moiety.

**Scheme 3:**
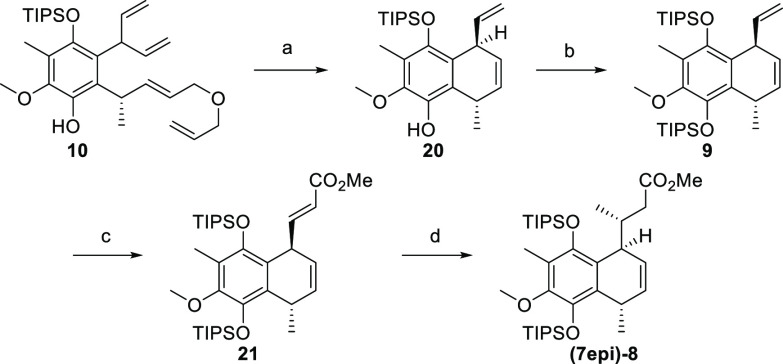
Synthesis of Compound **(7epi)-8** Reagents and conditions: (a) Grubbs II, DCM, 40 °C, 2 h, 92%; (b) *n*-BuLi, TIPSCl, 1:1 THF/DMF, −80 to −35 °C, 1.5 h, 97%; (c) GH-II, methyl acrylate, PhMe, 80 °C, 25 h, 51% (+41% **9**); (d) CuCl, TMSCl, MeMgBr, THF, −42 to −22 °C, 1 h, quant.

Subjection of phenol **10** to Grubbs II smoothly triggered a relay-RCM furnishing compound **20** in 92% yield (Scheme 3).^24^ We were delighted to find that product formation proceeded with a full substrate control, affording the desired compound as a single diastereomer. TIPS protection proved to be of utmost importance in this step, as other phenol protecting groups led to significantly lower diastereoselectivity.^25^ The stereochemical assignment was achieved through NOESY experiments and was later confirmed by X-ray diffraction at the stage of the TIPS-protected product **9** (CCDC no. 2161382). According to the retrosynthetic plan, the next task was C-elongation to introduce an α,β-unsaturated ester moiety. This was accomplished by Ru-catalyzed cross-metathesis, and the desired compound **21** was obtained in 51% yield. With this material at hand, the stage was set for the introduction of a C-7 stereocenter. The treatment of compound **21** with TMSCl and MeMgBr in the presence of a catalytic amount of CuCl cleanly formed the corresponding 1,4-addition product as a single diastereomer in quantitative yield.^26^ Unfortunately, X-ray crystallography revealed undesired C-7 configuration, forming **(7epi)-8** (CCDC no. 2161383). All attempts to overwrite the substrate control using a chiral catalyst proved unsuccessful.^27−29^ Notably, other cuprate conditions lead to no conversion.^30−33^

**Figure 4:**
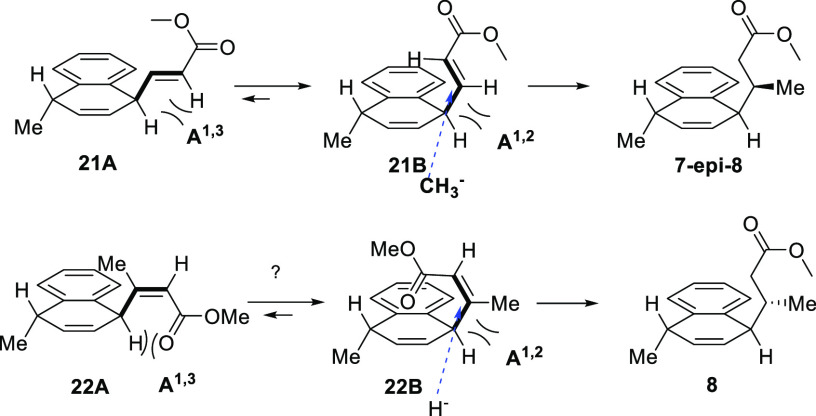
Possible explanation for the observed diastereoselectivity.

The observed stereochemical outcome of the 1,4-addition suggested that the reaction did not proceed *via***21A** but conformer **21B** (A_1,3_ > A_1,2_ strain) (Figure 4, aromatic substitution omitted for clarity). Hence, the bulky C-14 and C-17 OTIPS groups are successfully blocking the backside of the molecule and the metalorganic nucleophile can only approach the molecule from the front side (blue arrow) to give an undesired diastereomer. In attempts to overcome this issue, we decided to prepare ester **8** by conjugate reduction of **(*Z*)-22** with the expectation that in this case, A_1,3_ strain in **22A** would be dominant and secure **22B** as a reactive conformer.

**Scheme 4:**
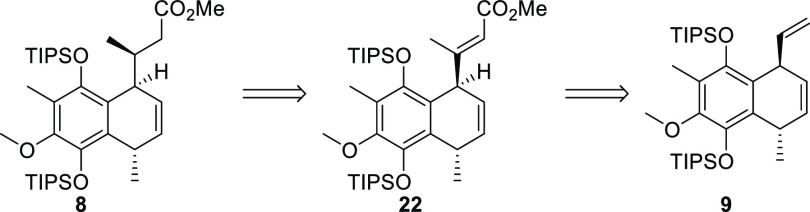
Retrosynthetic Analysis of Ester **8**

We anticipated that ester **22** could be derived from compound **9** by minor functional group manipulation (Scheme 4).

**Scheme 5:**
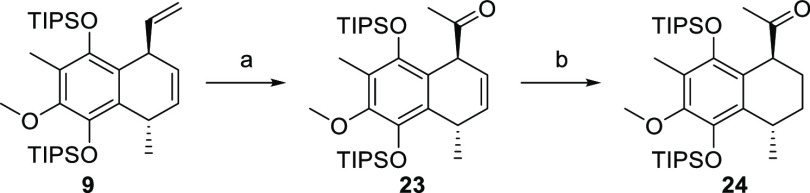
Synthesis of Compound **24** Reagents and conditions: (a) PdCl_2_(MeCN)_2_, BQ, 9:3:0.75 DMAC/DCE/H_2_O, 36 °C, 5 h, 92%; (b) [Ir(cod)(PCy_3_)(py)]PF_6_, H_2_, DCM, rt, 1 h, 99%.

Accordingly, compound **9** was subjected to standard Wacker oxidation conditions and ketone **23** was obtained in 92% yield (Scheme 5). Unfortunately, C-elongation under HWE or Peterson olefination conditions proved unsuccessful and only the starting material was isolated.^34−40^

**Scheme 6:**
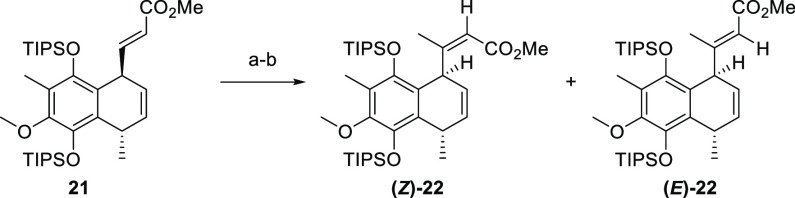
Synthesis of Compound **22** Reagents and conditions: (a) CuCl, TMSCl, MeMgBr, THF, −50 to −20 °C, 45 min, then PhSeCl; (b) H_2_O_2_, pyridine, CHCl_3_, rt, 30 min, 12% **(*Z*)-22**, 20% **(*E*)-22** (over two steps).

The same lack of reactivity was observed with ketone **24**. Faced with this early setback, the plan was adapted and compound **22** was prepared in a two-step sequence. In the first step, compound **21** was subjected to conjugate addition under standard conditions (CuCl, TMSCl, and MeMgBr) followed by addition of PhSeCl to give the corresponding α-phenyl selenide.^41^ Selective oxidation then triggered selenoxide elimination, forming a set of separable esters, compound **(*Z)*-22** and **(*E*)-22** (Scheme 6).^42^

**Scheme 7:**
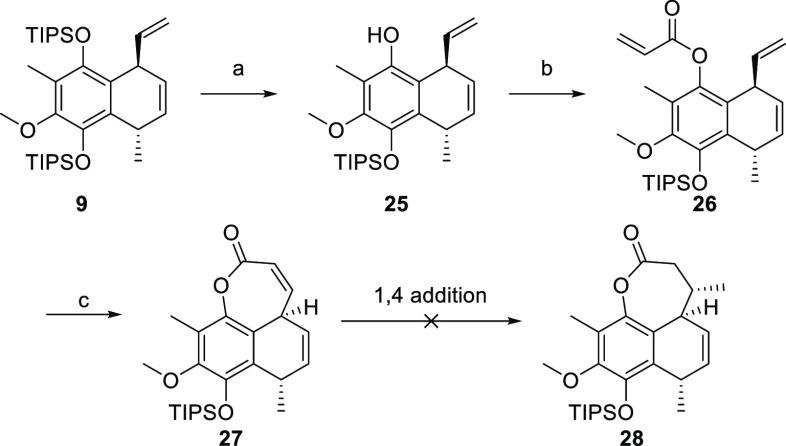
Synthesis of Compound **27** Reagents and conditions: (a) TBAF, AcOH, THF, 0 °C to rt, 2 h, 40% (+54% starting material); (b) NaH, acryloyl chloride, THF, −23 to −20 °C, 1.5 h, 83%; (c) GH-II, PhMe, 80 °C, 21 h, 98%.

Unfortunately, all attempts to reduce esters **(*Z*)-22** and **(*E*)-22** into the desired C-7 methyl ester **8** were unsuccessful.^43−50^ Inspired by our second approach (see below), in our next attempt to create a C-7 chiral center, we planned to perform conjugated addition of methyl cuprate to lactone **27** (Scheme 7), a reaction that was successful for a similar substrate.

The sequence commenced with selective deprotection (1:2 TBAF/AcOH) of compound **9** to give phenol **25** in 40% yield (Scheme 7).^51^ The reaction was stopped short before 50% conversion, and the remaining starting material was recycled. The treatment of **25** with acryloyl chloride provided **26**, which was subjected to RCM to form lactone **27** in excellent yield. To our great disappointment, the desired 1,4-addition proved to be infeasible, refusing access to lactone **28**. One explanation for the observed lack of reactivity could be that the α,β-unsaturated lactone is out of conjugation. This theory was underpinned by ^1^H-NMR evidence. The chemical shifts of the α- and β-protons (α: 5.76 ppm; β: 6.51 ppm) suggest a low degree of polarization compared to similar α,β-unsaturated systems, e.g., compound **(rac)-35** (α: 5.90 ppm; β: 6.85 ppm). These unsuccessful attempts for direct introduction of C-7 methyl with desired configuration compelled a change in our strategy.

### Second Approach

**Scheme 8:**
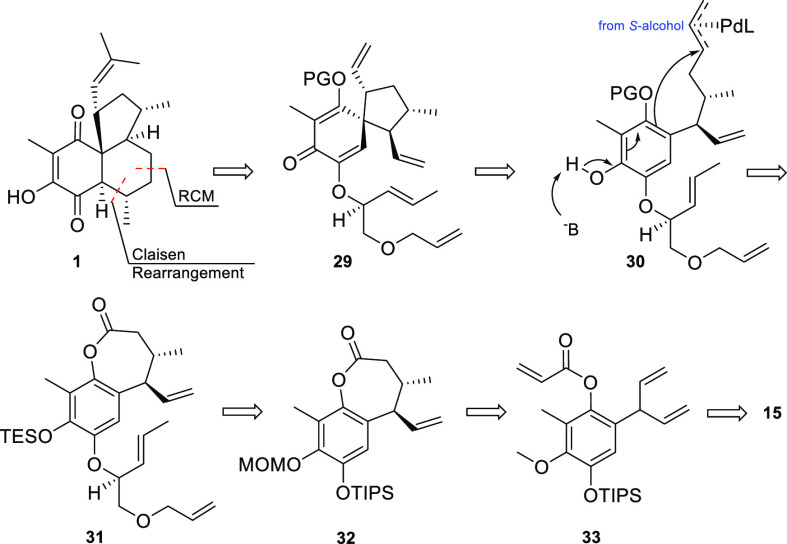
Second-Generation Retrosynthetic Analysis of Elisabethin A (**1**)

For our second approach, elisabethin A (**1**) was simplified by scission of the C-2–C-3 bond as well as the C-4–C-5 bond (Scheme 8). The first connection should be accomplished by a Claisen rearrangement, and the second should be accomplished *via* RCM. Carving out the second six-membered ring allowed the identification of enantiopure spiro compound **29**. The said advanced intermediate would be assembled from a (9*S*)-alcohol-derivative **30** by Pd-catalyzed allylic alkylation.^52^ Compound **30** could be traced back to chiral ether **31**, which in turn should be derived from lactone **32**. Finally, enantioenriched lactone **32** could be accessible from ester **33***via* the Claisen rearrangement, followed by enantioselective RCM and 1,4-addition. The known compound **15** was chosen as a starting material for this approach.

**Scheme 9:**
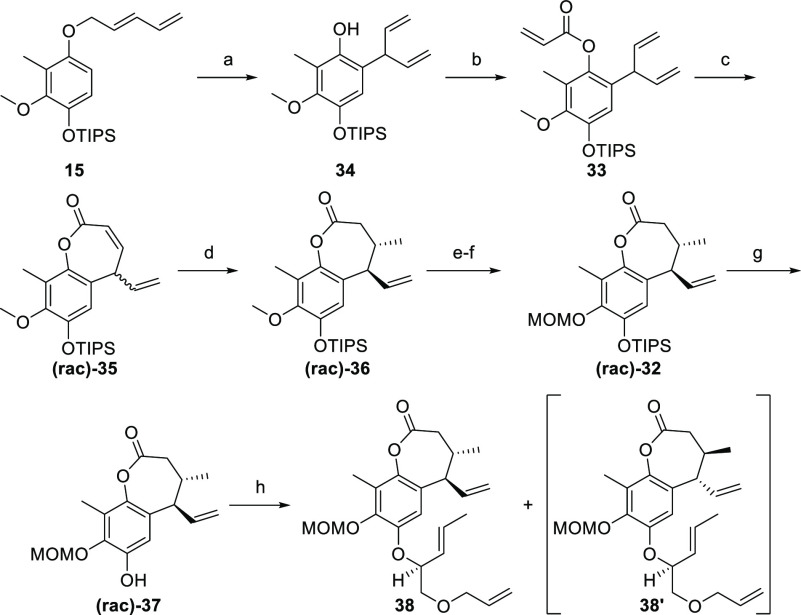
Synthesis of Compound **38** Reagents and conditions: (a) EuFOD, PhMe, 110 °C, 4 h, 97%; (b) acryloyl chloride, Et_3_N, DCM, 0 °C to rt, 2 h, 93%; (c) GH-II, PhMe, 80 °C, 15 h; (d) CuCl, LiCl, TMSCl, MeMgBr, THF, −40 °C, 15 min, 42% (over two steps); (e) AlCl_3_, Me_2_S, DCM, rt, 30 min, 89%; (f) MOMCl, LiHMDS, NaI, THF, −35 to −26 °C, 25 min, 94%; (g) TBAF, THF, rt, 3 min, 77%; (h) PBu_3_, ADDP, **12**, PhMe, rt, 2 h, 36% (+36% **38′**).

Guided by the retrosynthetic plan, the known ether **15** was elaborated into phenol **34** by EuFOD catalysis (Scheme 9). Next, phenol **34** was converted into compound **33**, which was directly subjected to RCM in the presence of a chiral Mo catalyst.^53^ Unfortunately, all our attempts to perform this enantioselective cyclization failed and only the starting material was isolated.^54,55^ Contrastingly, the same reaction in the presence of an HG-II catalyst afforded racemic material **(rac)-35**. At this point, we decided to go ahead with racemic lactone and to explore the feasibility of the intended five-membered ring spiro cyclization.

Accordingly, organo cuprate addition produced racemic lactone **(rac)-36** in 42% yield over two steps. The trans relationship of the newly introduced C-7 methyl and C-6 vinyl substituents was confirmed by X-ray diffraction (CCDC no. 2161384). To elude late-stage difficulties, the methoxy protection needed to be exchanged. Thus, a two-step protocol was employed, commencing with methoxy cleavage (AlCl_3_ and Me_2_S) and followed by MOM installation, which gave rise to lactone **(rac)-32**.^56^ Subsequently, fluoride-mediated TIPS removal revealed phenol **(rac)-37**, setting the stage for the Mitsunobu etherification. The merging of compound **(rac)-37** and chiral alcohol **12** furnished ether **38** and its C-6/C-7 epimer **38′** (1:1, 72% total yield) accompanied by minor amounts of their corresponding SN2′ products (not shown).

**Scheme 10:**
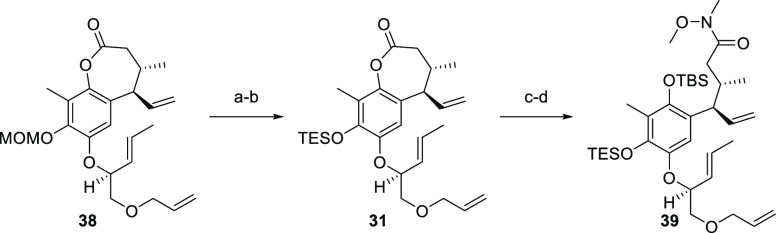
Synthesis of Compound **39** Reagents and conditions: (a) MgBr_2_·Et_2_O, EtSH, DCM, 4 °C to rt, 1.5 h, 95%; (b) TESOTf, 2,6-lutidine, DCM, −85 °C to rt, 25 min; (c) MeONHMe·HCl, AlMe_3_, PhMe, −20 to −15 °C, 45 min, 93% (over two steps); (d) TBSOTf, 2,6-lutidine, DCM, −92 °C to rt, 83% (+11% **31**).

Preliminary experiments in our group suggested that MOM was also not a suitable protecting group for late-stage deprotection. Therefore, it was exchanged again in a two-step sequence. Selective cleavage removed the acetal protecting group of **38**, leaving the ether residue unchanged (Scheme 10).^57^ To our delight, X-ray diffraction of this material confirmed the depicted relative stereochemistry (see Experimental Section, compound **S7**, and Supporting Information, CCDC no. 2161381). In the second step, TES was installed and the obtained silyl ether **31** was directly converted into the corresponding Weinreb amide. Phenol protection by means of TBS ether led to the formation of compound **39**.

**Scheme 11:**
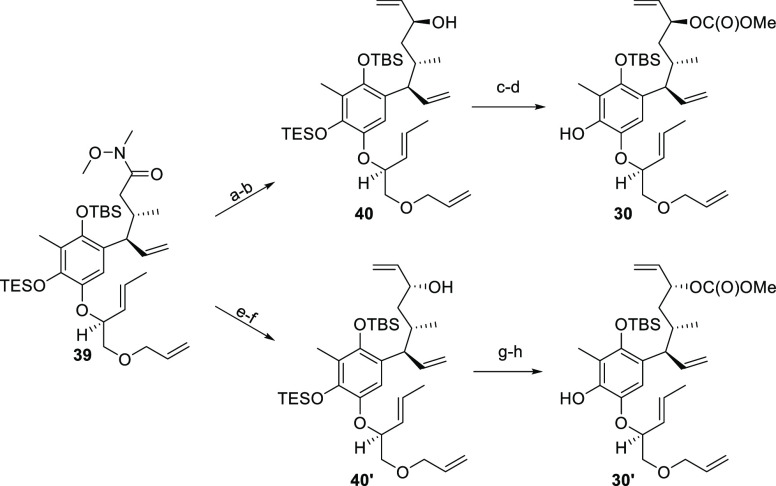
Synthesis of Compound Cyclization Precursors **30** and **30′** Reagents and conditions: (a) CH_2_=CHMgBr, THF, 0 °C to rt, 1 h; (b) DIBAL, DCM, −90 °C, 15 min, 70% (two steps, dr = 11:1); (c) ClC(*O*)OMe, pyridine, DCM, −20 to −5 °C, 2 h; (d) TFA, 4:1 THF/H_2_O, rt, 1.5 h, 99% (two steps); (e) DIBAL, PhMe, −91 °C, 2 min, 98%; (f) CH_2_=CHMgBr, THF, 4 °C to rt, 20 min, 45% (dr = 2:1); (g) ClC(*O*)OMe, pyridine, DCM, −20 to 10 °C, 2 h; (h) TFA, 4:1 THF/H_2_O, rt, 2 h and 50 min, 92% (two steps).

The retrosynthetic analysis depicted in Scheme 8 suggested that (9*S*)-alcohol was required for the following spiro cyclization. However, to investigate this crucial transformation in detail, it was decided to prepare the corresponding (9*R*)-alcohol as well. Therefore, two slightly different sequences from compound **39** were conducted in parallel (Scheme 11). First, vinyl Grignard addition gave rise to the corresponding ketone and the following reduction with DIBAL produced (9*S*)-allylic alcohol **40** in 70% isolated yield over two steps and dr = 11:1.^58^ The stereochemistry of (9*S*)-allylic alcohol **40** was unambiguously confirmed by X-ray diffraction (see the Supporting Information). Conversion of compound **40** into the corresponding methyl carbonate proceeded uneventfully. Subsequent acid-mediated TES removal gave rise to phenol **30** in virtually quantitative yield over two steps. To access the corresponding (9*R)*-allyl carbonate **30′**, compound **39** was subjected to DIBAL reduction. The obtained aldehyde was immediately treated with vinyl Grignard to give (9*R*)-allylic alcohol **40′** as a major epimer in 45% yield over two steps (dr = 2:1). The following two-step sequence was identical to the preparation of phenol **30**. Carbonate formation and subsequent TES deprotection transformed compound **40′** into cyclization precursor **30′** in 92% yield. With both epimers **30** and **30′** at hand, the stage was set to investigate the cyclization event.

**Scheme 12:**
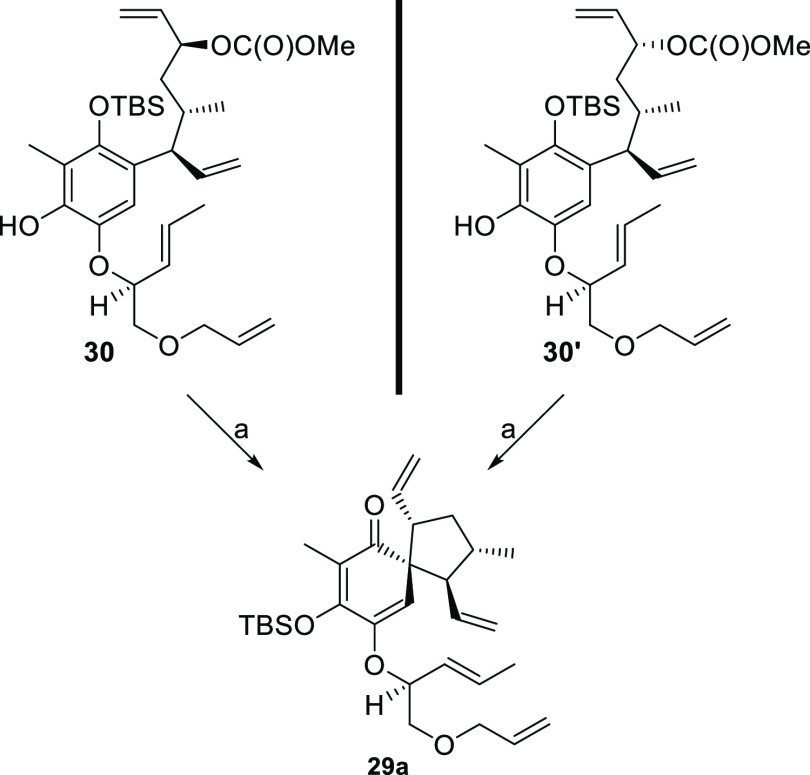
Synthesis of Compound **29a** Reagents and conditions: (a) Pd(dba)_2_, PPh_3_, DCM, rt, 0.5 h, 21% from **30**; 18% from **30′**.

Subjecting phenol **30** to Pd catalysis triggered the desired spiro formation (Scheme 12).^52^ The product was obtained in 21% yield after column chromatography. Gratifyingly, NOESY experiments confirmed the expected stereochemical outcome of the reaction. To our surprise, HMBC experiments suggested compound **29a** to be the reaction product instead of the expected material **29** (see the Supporting Information for details). To gain more insight into this transformation, phenol **30′** was subjected to the same reaction conditions. Surprisingly, compound **29a** was isolated in 18% yield as the major product. The observed product distribution could be explained by an equilibrium eroding the initial stereo information before the cyclization event took place.^59^ Notably, the Pd-catalyzed cyclizations could only be carried out in DCM and CD_2_Cl_2_ respectively and all attempts of improving yield and selectivity by means of solvent change (THF, MeCN, PhMe, DMF or 1,4-dioxane) were fruitless.^60^ Additionally, application of various monodentate ligands (P(2-furyl)_3_, P(OEt)_3_, P(OPh)_3_, AsPh_3_) or Trost’s chiral bidentate ANDEN ligand led to a complete cessation of the reaction.^52,61^ In consideration of this, we choose to pursue an alternative process for the construction of the spirocenter. Inspired by literature, we intended to conduct this reaction in a SN2-like manner under Mitsunobu conditions.^62^ We had good reason to be confident as initial results suggested the feasibility of this approach.

**Scheme 13:**
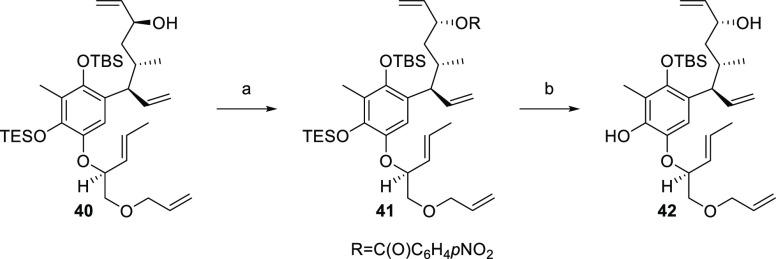
Synthesis of Compound **42** Reagents and conditions: (a) *p*-NO_2_C_6_H_4_CO_2_H, PPh_3_, DIAD, PhMe, rt, 40 min, 63%; (b) K_2_CO_3_, 2.5:1 EtOH/iPrOH, rt, 48 h, 76%.

In parallel to the synthetic efforts depicted in Scheme 11, we investigated the inversion of the C-9 stereocenter of compound **41** by Mitsunobu reaction (Scheme 13). Therefore, (9*S*)-allylic alcohol **40** was treated with standard conditions to obtain ester **41** in 63% yield, together with 19% of **41** lacking TES protection (not shown). Additionally, traces of a spiro-compound could be identified, conceivably formed by acid-mediated TES cleavage and following cyclization. The synthesis continued with ester hydrolysis and simultaneous cleavage of TES-liberating phenol **42**.

**Scheme 14:**
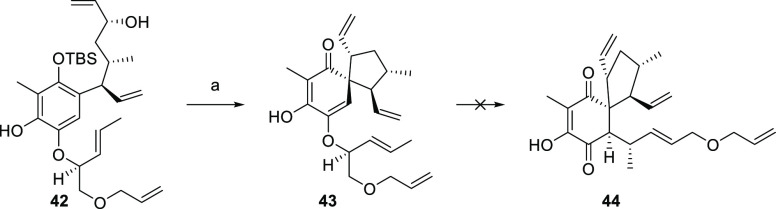
Synthesis of Compound **43** Reagents and conditions: (a) PPh_3_, DIAD, PhMe, 0 °C, 2 h, 94%.

With the desired precursor **42** at hand, we were ready to test the envisioned spiro formation. To our great delight, treatment with PPh_3_ and DIAD in dry toluene at 0 °C cleanly formed compound **43** (Scheme 14). Interestingly, this transformation proceeded with the cleavage of TBS protection. This facile method for the construction of spiro [4,5] ketones was found superior to the Pd catalysis approach with respect to yield and selectivity. To the best of our knowledge, this represents the first example of a spiro [4,5] ketone formation under Mitsunobu conditions. The generality of this method is subject to current investigations within our group. With this satisfying result at hand, we turned our attention toward the remaining six-membered ring of target molecule **1**. Hence, next on the agenda was the Claisen rearrangement of allyl-vinyl ether **43** to compound **44**. Unfortunately, all attempts to conduct this transformation proved fruitless and the desired material could not be obtained.^63−66^ This final dead end marked the end of our efforts, and this approach was abandoned.

## Conclusions

The synthetic challenges presented led to the improvement and discovery of underdeveloped and unknown reactions. The aryl-dienyl ether Claisen rearrangement proved to be highly reliable as well as high yielding and was successfully applied in both approaches. Although the final goal of preparing the desired target compound **1** was denied to us, our efforts allowed the synthesis of several more advanced intermediates. During our first approach, we achieved the synthesis of compounds **21** and **(7epi)-8** in 17 and 18 linear steps with average respective yields per step of 86 and 87%, respectively. The main feature of the second approach was the spiro cyclization, which could be achieved in two different ways. The corresponding material **29a**—accessible *via* Pd catalysis—was prepared in 24 linear steps (average 81% per steps). The unprecedented formation of a spiro [4,5] ketone under Mitsunobu conditions allowed the preparation of compound **43** over 24 linear steps in 1.6% overall yield. The lessons we have learnt during this endeavor will be considered when developing the next-generation approach toward the completion of the total synthesis.

## Experimental Section

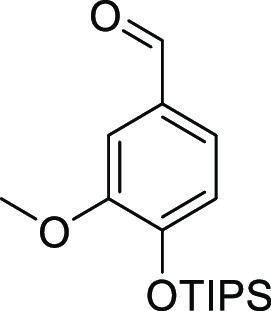



### 3-Methoxy-4-((triisopropylsilyl)oxy)benzaldehyde (**S1**)

A 500 mL Schlenk flask was charged with commercially available vanillin (40 g, 263 mmol, 1.00 equiv) in 200 mL of DMF/THF, and DIPEA (89.5 mL, 67.96 g, 526 mmol, 2 equiv) was added, causing a color change from pale yellow to strong orange. After addition of TIPSCl (61.9 mL, 55.76 g, 289 mmol, 1.1 equiv), the color changed to turbid yellow with a white precipitate forming. After 1 h, TLC (petroleum ether/ethyl acetate, 4:1) confirmed full conversion. The reaction was quenched by addition of solid NH_4_Cl, and the solvents were evaporated under reduced pressure. The residue was taken up in Et_2_O, filtered, and concentrated in vacuo. It was dried on high vacuum overnight, and the desired product was obtained as a red oil in quantitative yield (81.1 g, 263 mmol). The material was used in the next reaction without further purification. ^1^H-NMR (400 MHz, CDCl_3_): δ = 9.83 (s, 1H), 7.38 (d, *J* = 1.9 Hz, 1H), 7.34 (dd, *J* = 8.0, 2.0 Hz, 1H), 6.97 (d, *J* = 8.0 Hz, 1H), 3.85 (s, 3H), 1.32–1.21 (m, 3H), 1.08 (d, *J* = 7.5 Hz, 18H); ^13^C{^1^H}-NMR (101 MHz, CDCl_3_): δ = 191.1, 151.9, 151.7, 130.7, 126.3, 120.2, 110.1, 55.5, 17.9, 13.0; IR [ATR]: ν = 2944, 2892, 2867, 2733, 1696, 1684, 1592, 1506, 1464, 1422, 1390, 1286, 1265, 1238, 1193, 1152, 1124, 1071, 1034, 1016, 997, 961, 938, 896, 882, 819, 781, 729, 705 cm^–1^; HRMS (ESI): exact mass calculated for C_17_H_29_O_3_Si [(M + H)^+^], 309.1880; found, 309.1865.

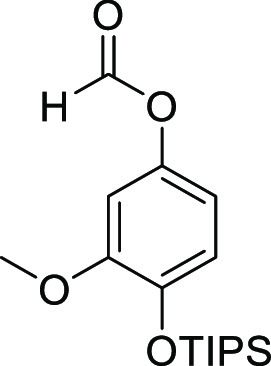



### 3-Methoxy-4-((triisopropylsilyl)oxy)phenyl Formate (**S2**)

A 500 mL Schlenk flask was charged with compound **S1** (40.0 g, 129.6 mmol, 1 equiv) in 250 mL of CHCl_3_. To the strong red solution was added *m*-CPBA (75%, 44.8 g, 194 mmol, 1.5 equiv) in six portions over 75 min. The flask was heated to reflux overnight, and TLC (toluene) on the next day confirmed full conversion. The reaction mixture was transferred into a 1000 mL Erlenmeyer flask and neutralized with 200 mL of saturated NaHCO_3_ solution. Layers were separated, and the organic layer was washed with NaHCO_3_ solution, followed by 20 mL of H_2_O and 20 mL of brine. The dark red solution was dried over MgSO_4_ and concentrated in vacuo. The product was obtained as a dark red oil and directly subjected to following ester hydrolysis without further purification. ^1^H-NMR (400 MHz, CDCl_3_): δ = 8.28 (s, 1H), 6.85 (d, *J* = 8.6 Hz, 1H), 6.64 (d, *J* = 2.8 Hz, 1H), 6.58 (dd, *J* = 8.6, 2.8 Hz, 1H), 3.79 (s, 3H), 1.30–1.18 (m, 3H), 1.09 (d, *J* = 7.3 Hz, 18H); ^13^C{^1^H}-NMR (101 MHz, CDCl_3_): δ = 159.8, 151.5, 144.0, 143.9, 120.3, 112.5, 105.7, 55.7, 18.0, 13.0; IR [ATR]: ν = 2944, 2892, 2867, 1765, 1741, 1601, 1506, 1464, 1450, 1416, 1384, 1314, 1278, 1263, 1227, 1188, 1155, 1102, 1035, 1016, 997, 961, 882, 787, 711 cm^–1^; HRMS (ESI): exact mass calculated for C_16_H_28_O_3_Si [(M – CO)], 296.1808; found, 296.1806.

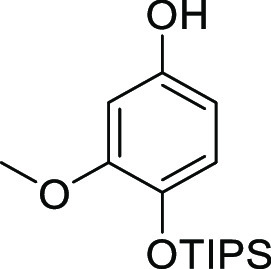



### 3-Methoxy-4-((triisopropylsilyl)oxy)phenol (**S3**)

Compound **S2** (42 g, 129 mmol, 1.00 equiv) was dissolved in 250 mL of MeOH, and K_2_CO_3_ (1.45 g, 26 mmol, 0.2 equiv) was added, causing an instant color change from red to black. After 15 min, TLC (petroleum ether/ethyl acetate, 6:1) showed full conversion and the reaction was quenched with solid NH_4_Cl. It was filtered and concentrated in vacuo. The desired product was obtained as a brown solid in 92% yield (35.5 g, 118.7 mmol) over two steps. The material was used in the next reaction without further purification. ^1^H-NMR (400 MHz, CDCl_3_): δ = 6.71 (d, *J* = 8.5 Hz, 1H), 6.40 (d, *J* = 2.9 Hz, 1H), 6.24 (dd, *J* = 8.5, 2.9 Hz, 1H), 3.74 (s, 3H), 1.27–1.16 (m, 3H), 1.08 (d, *J* = 7.3 Hz, 18H); ^13^C{^1^H}-NMR (101 MHz, CDCl_3_): δ = 151.5, 150.3, 139.4, 120.5, 106.4, 100.9, 55.5, 18.0, 12.9; IR [ATR]: ν = 3411, 2942, 2890, 2865, 2051, 1980, 1602, 1513, 1461, 1450, 1381, 1353, 1293, 1281, 1230, 1214, 1200, 1164, 1112, 1073, 1061, 1025, 1016, 993, 951, 932, 902, 881, 806, 732, 716 cm^–1^; HRMS (ESI): exact mass calculated for C_16_H_29_O_3_Si [(M + H)^+^], 297.1880; found, 297.1880.

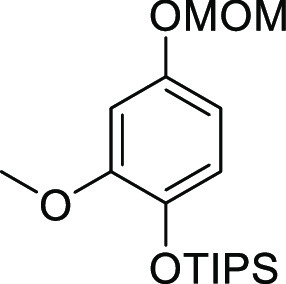



### Triisopropyl(2-methoxy-4-(methoxymethoxy)phenoxy)silane (**S4**)

A 1000 mL three-necked flask was charged with compound **S3** (34.6 g, 117 mmol, 1 equiv) dissolved in 750 mL of dry DCM. DIPEA (40 mL, 233 mmol, 2 equiv) was added, and the flask was equipped with a reflux condenser and argon balloon before MOMCl (16.8 mL, 222 mmol, 1.9 equiv) was added dropwise. After full addition, the reaction mixture was heated to reflux for 4 h. After TLC (petroleum ether/ethyl acetate, 6:1) confirmed full conversion, the reaction was quenched by addition of solid NaHCO_3_ and H_2_O. Layers were separated, the aqueous layer was extracted three times with DCM, and the combined organic layer was washed with H_2_O (4×), followed by brine. The dark red solution was dried over MgSO_4_ and concentrated in vacuo. The desired product was obtained as a dark oil in 97% yield (38.5 g, 113.5 mmol). The product was used in the next reaction without further purification. ^1^H-NMR (400 MHz, CDCl_3_): δ = 6.77 (d, *J* = 8.7 Hz, 1H), 6.58 (d, *J* = 2.8 Hz, 1H), 6.48 (dd, *J* = 8.7, 2.8 Hz, 1H), 5.10 (s, 2H), 3.78 (s, 3H), 3.48 (s, 3H), 1.28–1.18 (m, 3H), 1.09 (d, *J* = 7.3 Hz, 18H); ^13^C{^1^H}-NMR (101 MHz, CDCl_3_): δ = 152.1, 151.4, 140.6, 120.3, 107.3, 102.3, 95.4, 56.0, 55.5, 18.0, 12.9; IR [ATR]: ν = 2944, 2892, 2866, 1591, 1508, 1464, 1451, 1418, 1403, 1384, 1367, 1305, 1268, 1227, 1189, 1151, 1127, 1111, 1077, 1014, 998, 937, 921, 882, 836, 805, 731 cm^–1^; HRMS (ESI): exact mass calculated for C_18_H_33_O_4_Si [(M + H)^+^], 341.2143; found, 341.2107.

### Triisopropyl(2-methoxy-4-(methoxymethoxy)-3-methylphenoxy)silane (**13**)

A 1000 mL Schlenk flask was charged with compound **S4** (39.8 g, 117 mmol, 1 equiv) in 400 mL of dry THF. It was cooled to −50 °C, and *n*-BuLi (1.6 M, 88 mL, 140 mmol, 1.2 equiv) was added dropwise over 20 min. During addition, the color changed from bright red to strong dark red. Note that if an excess of *n*-BuLi was used in this transformation, double methylation (C-15 and C-1) was observed. The reaction mixture was allowed to warm to −10 °C over 1.5 h at which point D_2_O quenching confirmed full lithiation. It was cooled to −20 °C, and MeI (15 mL, 234 mmol, 2 equiv) was added dropwise over 5 min. The reaction was allowed to warm to room temperature overnight, upon which TLC (petroleum ether/ethyl acetate, 10:1) confirmed full conversion. The mixture was quenched by addition of solid NH_4_Cl and filtered. The residue solution was washed with H_2_O and brine, dried over MgSO_4_, and concentrated in vacuo. The desired product was obtained as a yellow oil in quantitative yield (41.4 g, 117 mmol). The product was used in the next reaction without further purification. ^1^H-NMR (400 MHz, CDCl_3_): δ = 6.67 (d, *J* = 8.9 Hz, 1H), 6.63 (d, *J* = 9.5 Hz, 1H), 5.11 (s, 2H), 3.77 (s, 3H), 3.49 (s, 3H), 2.17 (s, 3H), 1.33–1.19 (m, 3H), 1.10 (d, *J* = 7.4 Hz, 18H); ^13^C{^1^H}-NMR (101 MHz, CDCl_3_): δ = 152.1, 151.4, 140.6, 120.3, 107.3, 102.3, 95.4, 56.0, 55.5, 18.0, 12.9; IR [ATR]: ν = 2944, 2894, 2867, 1591, 1484, 1464, 1418, 1404, 1390, 1297, 1274, 1248, 1222, 1186, 1153, 1101, 1065, 1038, 1013, 991, 922, 881, 869, 824, 804, 761, 734 cm^–1^; HRMS (ESI): exact mass calculated for C_19_H_33_O_4_Si [M^+^], 353.2143; found, 353.2141.

### 3-Methoxy-2-methyl-4-((triisopropylsilyl)oxy)phenol (**14**)

A 250 mL three-necked flask was charged with compound **13** (20.25 g, 57 mmol, 1 equiv) in 60 mL of dry DCM, and ZnBr_2_ (12.9 g, 57 mmol, 1 equiv) was added, causing a color change from red to brown. The mixture was cooled to −32 °C, and EtSH (21.1 mL, 286 mmol, 5 equiv) was added quickly in one portion. The reaction was allowed to warm to −5 °C over 2.5 h, upon which TLC (petroleum ether/ethyl acetate, 10:1) confirmed full conversion. Note that with higher reaction temperature or fewer equivalents of EtSH, significant amounts of undesired side products **14′** were obtained. The reaction was quenched by addition of 20 mL of saturated NaHCO_3_ solution, allowed to warm to room temperature, and stirred for an additional 1.5 h. Layers were separated, and the aqueous layer was extracted three times with DCM. The combined organic layer was washed with H_2_O (2×) and brine, dried over MgSO_4_, and concentrated in vacuo. The crude oil was purified by flash chromatography (petroleum ether/ethyl acetate, 10:1), and the pure product was obtained as a yellow oil in 89% yield (15.7 g, 50.7 mmol). ^1^H-NMR (400 MHz, CDCl_3_): δ = 6.60 (d, *J* = 8.7 Hz, 1H), 6.41 (d, *J* = 8.6 Hz, 1H), 4.42 (bs, 1H), 3.78 (s, 3H), 2.17 (s, 3H), 1.34–1.19 (m, 3H), 1.10 (d, *J* = 7.4 Hz, 18H); ^13^C{^1^H}-NMR (101 MHz, CDCl_3_): δ = 149.7, 148.3, 143.2, 118.9, 117.3, 110.0, 60.3, 18.1, 12.9, 9.2; IR [ATR]: ν = 3341, 2967, 2936, 2863, 2846, 1598, 1496, 1460, 1422, 1381, 1367, 1304, 1262, 1211, 1174, 1156, 1075, 1034, 995, 919, 908, 891, 885, 821, 799, 758, 709 cm^–1^; HRMS (ESI): exact mass calculated for C_17_H_31_O_3_Si [(M + H)^+^], 311.2037; found, 311.2050.

### (*E*)-Triisopropyl(2-methoxy-3-methyl-4-(penta-2,4-dien-1-yloxy)phenoxy)silane (**15**)

A 500 mL three-necked flask was charged with compound **14** (5.0 g, 16 mmol, 1 equiv) in 100 mL of dry THF. The pale-yellow solution was cooled to −35 °C, and *n*-BuLi (1.6 M, 10 mL, 16 mmol, 1 equiv) was added dropwise over 5 min. After 15 min, 5-bromopenta-1,3-diene (3.53 g, 18 mmol, 1.1 equiv, 73% in pentane) dissolved in 70 mL of dry DMF was added dropwise over 30 min. The reaction was allowed to warm to room temperature overnight, upon which TLC (petroleum ether/ethyl acetate, 12:1) confirmed full conversion. It was quenched by addition of solid NH_4_Cl, filtered, and concentrated in vacuo. The oily residue was taken up in ethyl acetate and little H_2_O. Layers were separated, and the aqueous layer was extracted three times with ethyl acetate. The combined organic layer was washed with H_2_O and brine, dried over MgSO_4_, and concentrated in vacuo. The crude oil was purified by flash chromatography (petroleum ether/ethyl acetate, 10:1), and the pure product was obtained as a yellow oil in 97% yield (5.9 g, 15.5 mmol). ^1^H-NMR (400 MHz, CDCl_3_): δ = 6.65 (d, *J* = 8.8, 1H), 6.45 (d, *J* = 8.9 Hz, 1H), 6.42–6.32 (m, 2H), 5.98–5.87 (m, 1H), 5.31–5.18 (m, 1H), 5.18–5.07 (m, 1H), 4.52–4.49 (m, 2H), 3.78 (s, 3H), 2.18 (s, 3H), 1.33–1.22 (m, 3H), 1.11 (d, *J* = 7.4 Hz, 18H); ^13^C{^1^H}-NMR (101 MHz, CDCl_3_): δ = 151.5, 149.9, 143.6, 136.4, 133.0, 129.3, 121.8, 117.9, 116.7, 107.3, 69.0, 60.2, 18.1, 12.9, 9.4; IR [ATR]: ν = 2944, 2892, 2866, 1605, 1483, 1463, 1417, 1383, 1368, 1274, 1253, 1220, 1185, 1164, 1103, 1037, 999, 951, 904, 881, 822, 793, 762, 733 cm^–1^; HRMS (ESI): exact mass calculated for C_22_H_37_O_3_Si [(M + H)^+^], 377.2506; found, 377.2494.

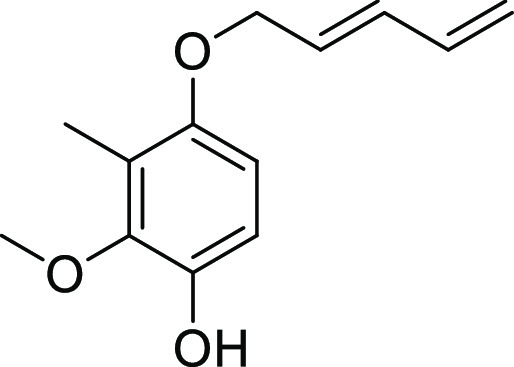



### (*E*)-2-Methoxy-3-methyl-4-(penta-2,4-dien-1-yloxy)phenol (**S5**)

A 250 mL round-bottom flask was charged with compound **15** (4.22 g, 11.21 mmol, 1 equiv) in 75 mL of dry THF, and TBAF (1.0 M in THF, 11.8 mL, 11.8 mmol, 1.05 equiv) was added. After 2 min, TLC (petroleum ether/ethyl acetate, 10:1) confirmed full conversion. The reaction was quenched by addition of saturated NH_4_Cl solution, and the aqueous layer was extracted twice with ethyl acetate. The combined organic layer was washed twice with NH_4_Cl and H_2_O, followed by brine. It was dried over MgSO_4_ and concentrated in vacuo. The crude red oil was dried over high vacuum at 40 °C overnight. The received brown solid was purified by flashed chromatography over silica (gradient: petroleum ether/ethyl acetate, 5:1–3:1–1:1). The desired product was obtained as a pale brown solid in 95% yield (2.35 g, 10.67 mmol). ^1^H-NMR (400 MHz, CDCl_3_): δ = 6.73 (d, *J* = 8.8, 1H), 6.54 (d, *J* = 8.8 Hz, 1H), 6.45–6.32 (m, 2H), 5.98–5.84 (m, 1H), 5.35–5.19 (m, 2H), 5.19–5.08 (m, 1H), 4.54–4.48 (m, 2H), 3.78 (s, 3H), 2.21 (s, 3H); ^13^C{^1^H}-NMR (101 MHz, CDCl_3_): δ = 151.0, 146.1, 143.3, 136.4, 133.2, 129.2, 120.7, 118.0, 111.8, 108.7, 69.3, 61.0, 9.6; HRMS (ESI): exact mass calculated for C_13_H_17_O_3_ [(M + H)^+^], 221.1172; found, 221.1176.

### (*E*)-Triethyl(2-methoxy-3-methyl-4-(penta-2,4-dien-1-yloxy)phenoxy)silane (**16**)

A 100 mL round-bottom flask was charged with compound **S5** (2.33 g, 10.58 mmol, 1 equiv) and imidazole (1.51 g, 22.21 mmol, 2.1 equiv) in 35 mL of dry DCM. TESCl (2.66 mL, 15.87 mmol, 1.5 equiv) was added dropwise to the orange solution. An immediate color change to yellow was observed accompanied by precipitation. TLC (petroleum ether/ethyl acetate, 5:1) after 20 min confirmed full conversion, and the reaction was quenched by addition of solid NaHCO_3_. It was filtered over silica with DCM and concentrated in vacuo. It was additionally dried on high vacuum overnight, and the desired product was obtained as an orange oil in 95% yield (3.35 g, 10.01 mmol). ^1^H-NMR (400 MHz, CDCl_3_): δ = 6.63 (d, *J* = 8.8 Hz, 1H), 6.46 (d, *J* = 8.8 Hz, 1H), 6.42–6.33 (m, 2H), 5.99–5.86 (m, 1H), 5.31–5.18 (m, 1H), 5.18–5.09 (m, 1H), 4.52–4.48 (m, 2H), 3.77 (s, 3H), 2.17 (s, 3H), 1.00 (t, *J* = 8.4, 9H), 0.74 (q, *J* = 8.3 Hz, 6H); ^13^C{^1^H}-NMR (101 MHz, CDCl_3_): δ = 151.8, 150.1, 143.0, 136.4, 133.0, 129.3, 121.6, 117.9, 117.2, 107.4, 69.0, 60.1, 9.4, 6.8, 5.2; HRMS (ESI): exact mass calculated for C_19_H_31_O_3_Si [(M + H)^+^], 335.2037; found, 335.2038.

### 3-Methoxy-2-methyl-6-(penta-1,4-dien-3-yl)-4-((triethylsilyl)oxy)phenol (**17**)

The starting material was azeotropically dried three times with toluene prior to use. A 1000 mL Schlenk flask was charged with compound **16** (9.0 g, 26.9 mmol, 1 equiv) in 270 mL of dry toluene. The pale-yellow solution was schlenked 10–15 times, then EuFOD (660 mg, 0.63 mmol, 2.3 mol %) was added in one portion, and the mixture was schlenked again 10–15 times. The flask was placed in a 110 °C oil bath and heated for 2.5 h. After TLC (petroleum ether/ethyl acetate, 10:1) confirmed full conversion, the reaction was allowed to cool to room temperature. It was quenched by addition of H_2_O, diluted with toluene, and stirred for 15 h. Layers were separated, and the organic layer was washed with H_2_O (5×) followed by brine. It was dried over MgSO_4_, filtered, and concentrated. The crude brown material was flashed over silica, and the desired product was collected as a yellow oil in 84% yield (7.56 g, 22.60 mmol). ^1^H-NMR (400 MHz, CDCl_3_): δ = 6.49 (s, 1H), 6.04 (ddd, *J* = 17.3, 10.3, 6.2 Hz, 2H), 5.24 (dt, *J* = 10.3, 1.5 Hz, 2H), 5.14 (dt, *J* = 17.3, 1.6 Hz, 2H), 4.75 (broad s, 1H), 4.21–4.12 (m, 1H), 3.76 (s, 3H), 2.15 (s, 3H), 0.99 (t, *J* = 7.9 Hz, 9H), 0.72 (q, *J* = 7.9 Hz, 6H); ^13^C{^1^H}-NMR (101 MHz, CDCl_3_): δ = 148.7, 146.6, 142.3, 138.7, 136.4, 133.1, 129.3, 122.2, 118.1, 116.5, 60.1, 47.7, 9.4, 6.8, 5.2; HRMS (ESI): exact mass calculated for C_19_H_31_O_3_Si [(M + H)^+^], 335.2037; found, 335.2024.

### Triethyl(2-methoxy-3-methyl-5-(penta-1,4-dien-3-yl)-4-((triisopropylsilyl)oxy)phenoxy)silane (**18**)

A 500 mL Schlenk flask was charged with compound **17** (7.56 g, 22.60 mmol, 1 equiv) in 230 mL of dry THF, and the yellow mix was cooled to −85 °C. After 5 min, *n*-BuLi (1.60 M, 17.0 mL, 27.12 mmol, 1.20 equiv) was added dropwise over 11 min. A change in color was observed from yellow over brown to dark red after full addition. The mixture was stirred at −85 °C for 30 min before TIPSOTf (6.68 mL, 24.86 mmol, 1.10 equiv) was added dropwise over 30 min. The reaction was allowed to slowly warm up, and after 1.5 h at −45 °C, TLC (petroleum ether/ethyl acetate, 20:1) confirmed full conversion. It was quenched with saturated NaHCO_3_ solution, diluted with toluene, and removed from cooling. The biphasic mixture was stirred for 16 h, and then the aqueous layer was extracted twice with ethyl acetate. The combined organic layer was washed with H_2_O and brine and dried over MgSO_4_. Solvents were removed in vacuo, and the crude residue was filtered over silica. The desired product was obtained as a colorless oil in 92% yield (10.19 g, 20.76 mmol). ^1^H-NMR (400 MHz, CDCl_3_): δ = 6.48 (s, 1H), 5.93 (ddd, *J* = 17.3, 10.3, 5.8 Hz, 2H), 5.12 (dt, *J* = 10.3, 1.6 Hz, 2H), 5.00 (dt, *J* = 17.3, 1.7 Hz, 2H), 4.46–4.40 (m, 1H), 3.75 (s, 3H), 2.18 (s, 3H), 1.38–1.23 (m, 3H), 1.11 (d, *J* = 7.5 Hz, 18H), 0.97 (t, *J* = 7.9 Hz, 9H), 0.71 (q, *J* = 8.3 Hz, 6H); ^13^C{^1^H}-NMR (101 MHz, CDCl_3_): δ = 148.3, 147.1, 142.6, 140.2, 127.1, 122.8, 118.3, 115.3, 59.9, 44.7, 18.3, 14.5, 11.5, 6.8, 5.2; HRMS (ESI): exact mass calculated for C_28_H_49_O_3_Si_2_ [(M – H)^+^], 489.3215; found, 489.3182.

### 2-Methoxy-3-methyl-5-(penta-1,4-dien-3-yl)-4-((triisopropylsilyl)oxy)phenol (**11**)

A 1000 mL round-bottom flask was charged with compound **18** (10.19 g, 20.76 mmol, 1 equiv) in 280 mL of THF, and TFA (10% in H_2_O, 71.0 mL, 62.28 mmol, 3 equiv) was added. After 30 min, TLC (petroleum ether/ethyl acetate, 20:1) confirmed full conversion. The reaction was quenched by addition of saturated NaHCO_3_ solution to pH = 7. The aqueous layer was extracted twice with ethyl acetate, and the combined organic layer was washed with H_2_O and brine. It was dried over MgSO_4_, filtered, and concentrated in vacuo. The crude material was filtered over silica, the solvent was removed in vacuo, and the pure product was dried on high vacuum overnight. The desired product was obtained as orange solids in 93% yield (7.28 g, 19.33 mmol). ^1^H-NMR (400 MHz, CDCl_3_): δ = 6.59 (d, *J* = 0.7 Hz, 1H), 5.94 (ddd, *J* = 17.3, 10.3, 6.0 Hz, 2H), 5.24–5.19 (m, 1H), 5.12 (ddd, *J* = 10.3, 1.6 Hz, 2H), 5.01 (ddd, *J* = 17.3, 1.7 Hz, 2H), 4.47–4.41 (m, 1H), 3.74 (s, 3H), 2.22 (d, *J* = 0.6 Hz, 3H), 1.36–1.25 (m, 4H), 1.11 (d, *J* = 7.4 Hz, 18H); ^13^C{^1^H}-NMR (101 MHz, CDCl_3_): δ = 146.2, 144.3, 142.9, 140.0, 128.5, 121.8, 115.5, 112.5, 60.8, 44.9, 18.3, 14.5, 11.6; HRMS (ESI): exact mass calculated for C_22_H_37_O_3_Si [(M + H)^+^], 377.2506; found, 377.2490.

### (*S*,*E*)-(4-((1-(Allyloxy)pent-3-en-2-yl)oxy)-3-methoxy-2-methyl-6-(penta-1,4-dien-3-yl)phenoxy)triisopropylsilane (**19**)

A 250 mL Schlenk flask was charged with compound **18** (1.82 g, 4.83 mmol, 1 equiv) and chiral alcohol **12** (824 mg, 5.80 mmol, 1.2 equiv) in 170 mL of dry toluene. The mixture was cooled in an ice bath, and PBu_3_ (93%, 1.67 mL, 6.28 mmol, 1.3 equiv) was added. After 5 min, ADDP (1.46 g, 5.80 mmol, 1.2 equiv) dissolved in 25 mL of dry toluene was added, causing a color change from yellow to brown. After 10 min, precipitation was observed. TLC (petroleum ether/ethyl acetate, 5:1) after 2 h confirmed full consumption of the starting material. Et_2_O was added to further precipitate OPBu_3_. The mixture was stirred for a few minutes and then flashed with pure Et_2_O over silica topped with Celite. The solvent was removed in vacuo, and the crude material was purified by column chromatography. The desired product was obtained as a pale-yellow oil in 69% yield (1.68 g, 3.35 mmol). ^1^H-NMR (400 MHz, CDCl_3_): δ = 6.55 (s, 1H), 5.99–5.83 (m, 3H), 5.65 (dqd, *J* = 15.5, 6.5, 0.8 Hz, 1H), 5.46 (ddq, *J* = 15.4, 7.6, 1.6 Hz, 1H), 5.33–5.23 (m, 1H), 5.21–5.14 (m, 1H), 5.13–5.06 (m, 2H), 5.05–4.90 (m, 2H), 4.69–4.60 (m, 1H), 4.46–4.40 (m, 1H), 4.11–4.04 (m, 2H), 3.79 (s, 3H), 3.69 (dd, *J* = 10.4, 6.4 Hz, 1H), 3.59 (dd, *J* = 10.4, 4.4 Hz, 1H), 2.17 (s, 3H), 1.67–1.60 (m, 3H), 1.34–1.24 (m, 3H), 1.09 (d, *J* = 7.4 Hz, 18H); ^13^C{^1^H}-NMR (101 MHz, CDCl_3_): δ = 148.2, 147.4, 144.8, 140.2, 140.2, 134.9, 130.7, 128.2, 126.6, 122.7, 117.1, 116.7, 115.3, 115.3, 79.9, 73.0, 72.5, 60.4, 44.7, 18.3, 17.9, 14.5, 11.4; HRMS (ESI): exact mass calculated for C_30_H_48_O_4_SiNa [(M + Na)^+^], 523.3214; found, 523.3214; [*α*]_D_^20^ = +19.72 (c 1.40, CH_2_Cl_2_).

### (*S*,*E*)-2-(5-(Allyloxy)pent-3-en-2-yl)-6-methoxy-5-methyl-3-(penta-1,4-dien-3-yl)-4-((triisopropylsilyl)oxy)phenol (**10**)

The starting material was azeotropically dried three times with toluene prior to use. A Schlenk tube was charged with compound **19** (0.90 g, 1.80 mmol, 1 equiv) in 18 mL of dry *o*-xylene. The solution was schlenked 10 times, then EuFOD (93 mg, 90 μmol, 5 mol %) was added, and the mix was schlenked again 10 times. The pale-yellow solution was placed in a 120 °C oil bath and heated for 8 h. Then, another portion of EuFOD (70 mg, 68 μmol, 3.8 mol %) was added and the reaction was heated again to 120 °C for 10 h. After TLC (DCM) confirmed full conversion, the reaction was cooled to room temperature and the solvent was removed in vacuo. The crude mixture was purified by column chromatography (DCM), and the desired product was obtained as a yellow oil in 55% yield (494 mg, 0.99 mmol). Phenol **11** was collected in 31% yield (211 mg, 0.56 mmol). ^1^H-NMR (400 MHz, CDCl_3_): δ = 6.17–6.03 (m, 3H), 5.98–5.83 (m, 1H), 5.57–5.46 (m, 1H), 5.40 (s, 1H), 5.24 (dq, *J* = 17.2, 1.7 Hz, 1H), 5.18–5.11 (m, 3H), 5.12–4.97 (m, 2H), 4.89–4.80 (m, 1H), 4.01–3.89 (m, 4H), 3.80–3.68 (m, 4H), 2.19 (s, 3H), 1.35 (d, *J* = 7.0 Hz, 3H), 1.31–1.21 (m, 3H), 1.10 (d, *J* = 7.4 Hz, 18H); ^13^C{^1^H}-NMR (101 MHz, CDCl_3_): δ = 146.5, 145.2, 142.8, 140.7, 140.4, 138.3, 135.1, 128.1, 127.0, 125.3, 119.2, 117.0, 115.6, 115.0, 71.1, 70.6, 60.8, 43.3, 37.1, 30.0, 18.7, 18.4, 14.4, 11.8; HRMS (ESI): exact mass calculated for C_30_H_48_O_4_SiNa [(M + Na)^+^], 523.3214; found, 523.3214; [*α*]_D_^20^ = −8.69 (c 0.70, CH_2_Cl_2_).

### (5*S*,8*S*)-2-Methoxy-3,8-dimethyl-4-((triisopropylsilyl)oxy)-5-vinyl-5,8-dihydronaphthalen-1-ol (**20**)

The starting material was azeotropically dried with toluene prior to use. A 250 mL Schlenk flask was charged with compound **10** (663 mg, 1.32 mmol, 1 equiv) in 130 mL of dry DCM. Two freeze–thaw cycles were carried out to degas the colorless solution before a Grubbs second-generation catalyst (70 mg, 66 μmol, 5 mol %) was added. The reaction was heated to reflux in a water bath, and after 2 h, TLC (petroleum ether/ethyl acetate, 10:1) confirmed full conversion. The flask was removed from heating and stirred on air for 30 min. The mixture was filtered over silica, and the desired product was obtained as slightly greenish solids in 92% yield (489 mg, 1.21 mmol). ^1^H-NMR (400 MHz, CDCl_3_): δ = 6.11 (ddd, *J* = 17.4, 10.3, 5.4 Hz, 1H), 5.84 (ddd, *J* = 10.2, 3.5, 1.6 Hz, 1H), 5.74 (ddd, *J* = 10.2, 4.2, 1.8 Hz, 1H), 5.43 (s, 1H), 4.91 (ddd, *J* = 10.3, 1.5 Hz, 1H), 4.85 (ddd, *J* = 17.4, 1.6 Hz, 1H), 4.20–4.15 (m, 1H), 3.73 (s, 3H), 3.57–3.48 (m, 1H), 2.20 (s, 3H), 1.34–1.24 (m, 6H), 1.13–1.04 (m, 18H); ^13^C{^1^H}-NMR (101 MHz, CDCl_3_): δ = 145.9, 143.8, 141.8, 141.0, 130.6, 124.1, 123.8, 119.1, 113.0, 60.8, 38.5, 29.9, 23.3, 18.2, 14.6, 11.1; HRMS (ESI): exact mass calculated for C_24_H_37_O_3_Si [(M – H)^+^], 401.2506; found, 401.2507; [*α*]_D_^20^ = +192.84 (c 1.65, CH_2_Cl_2_).

### (((5*S*,8*S*)-2-Methoxy-3,8-dimethyl-5-vinyl-5,8-dihydronaphthalene-1,4-diyl)bis(oxy))bis(triisopropylsilane) (**9**)

A 50 mL Schlenk flask was charged with compound **20** (489 mg, 1.21 mmol, 1 equiv) in 10 mL of dry THF and cooled to −80 °C. After 5 min, *n*-BuLi (2.50 M, 0.59 mL, 1.46 mmol, 1.2 equiv) was added dropwise over 8 min. A change in color was observed from greenish to orange. The reaction was kept at −80 °C, and after 20 min, 10 mL of dry DMF was added dropwise over 9 min, causing a slight color change to stronger yellow. Then, TIPSCl (0.39 mL, 1.82 mmol, 1.5 equiv) was added dropwise and the reaction was allowed to slowly warm up. After 1 h and 40 min, TLC (petroleum ether/ethyl acetate, 20:1) at −35 °C confirmed full conversion. The reaction was quenched by addition of saturated NH_4_Cl solution, and the aqueous layer was extracted three times with Et_2_O. The combined organic layer was washed three times with H_2_O followed by brine. It was dried over MgSO_4_, filtered, and concentrated in vacuo. The crude material was flashed over silica, and the desired product was collected as greenish white solids in 97% yield (657 mg, 1.18 mmol). ^1^H-NMR (400 MHz, CDCl_3_): δ = 6.01 (ddd, *J* = 17.3, 10.2, 5.7 Hz, 1H), 5.80 (ddd, *J* = 10.1, 3.7, 1.6 Hz, 1H), 5.70 (ddd, *J* = 10.1, 4.2, 1.7 Hz, 1H), 4.86 (ddd, *J* = 10.2, 1.4 Hz, 1H), 4.74 (ddd, *J* = 17.3, 1.5 Hz, 1H), 4.20–4.14 (m, 1H), 3.63 (s, 3H), 3.55–3.47 (m, 1H), 2.18 (s, 3H), 1.37–1.25 (m, 6H), 1.23 (d, *J* = 6.8 Hz, 3H), 1.13–1.00 (m, 36H); ^13^C{^1^H}-NMR (101 MHz, CDCl_3_): δ = 148.4, 147.1, 142.2, 141.7, 130.1, 129.6, 124.4, 123.2, 120.2, 112.7, 60.5, 39.0, 31.0, 24.6, 18.3, 18.3, 18.2, 18.2, 18.2, 14.6, 14.1, 11.3; HRMS (ESI): exact mass calculated for C_33_H_59_O_3_Si_2_ [(M + H)^+^], 559.3997; found, 559.3996; [*α*]_D_^20^ = +152.91 (c 0.95, CH_2_Cl_2_).

### Methyl (*E*)-3-((1*R*,4*S*)-6-Methoxy-4,7-dimethyl-5,8-bis((triisopropylsilyl)oxy)-1,4-dihydronaphthalen-1-yl)acrylate (**21**)

The starting material was azeotropically dried three times with toluene prior to use. A 25 mL Schlenk flask was charged with compound **9** (400 mg, 0.716 mmol, 1 equiv) in 4 mL of dry degassed toluene. Freshly distilled methyl acrylate (0.39 mL, 4.29 mmol, 6 equiv) was added, and the flask was placed in an 80 °C oil bath. A Hoveyda–Grubbs second-generation catalyst (23 mg, 37 μmol, 5.1 mol %) dissolved in 2 mL of dry degassed toluene was added *via* a syringe pump over 10 h. After complete addition, the reaction mixture was heated for another 15 h. The next day, the flask was removed from heating and allowed to cool to room temperature. The reaction mixture was directly flashed over silica, and the desired product was collected as a pale greenish oil in 51% yield (223 mg, 0.361 mmol) accompanied by 41% (162 mg, 0.290 mmol) starting material. ^1^H-NMR (400 MHz, CDCl_3_): δ = 7.11 (dd, *J* = 15.8, 6.0 Hz, 1H), 5.86 (ddd, *J* = 10.1, 3.8, 1.8 Hz, 1H), 5.65 (ddd, *J* = 10.1, 4.1, 1.7 Hz, 1H), 5.48 (dd, *J* = 15.7, 1.4 Hz, 1H), 4.35–4.28 (m, 1H), 3.65 (s, 3H), 3.63 (s, 3H), 3.56–3.47 (m, 1H), 2.17 (s, 3H), 1.36–1.25 (m, 6H), 1.23 (d, *J* = 6.7 Hz, 3H), 1.13–1.03 (m, 36H); ^13^C{^1^H}-NMR (101 MHz, CDCl_3_): δ = 167.8, 152.2, 149.0, 147.2, 141.8, 131.6, 129.7, 122.4, 121.0, 120.5, 119.3, 60.5, 51.4, 38.2, 30.9, 24.4, 18.3, 18.2, 18.1, 14.6, 14.1, 11.3; HRMS (ESI): exact mass calculated for C_33_H_57_O_3_Si_2_ [(M – C_2_H_3_O_2_)^+^], 557.3841; found, 557.3812; [*α*]_D_^20^ = +213.59 (c 1.0, CH_2_Cl_2_).

### Methyl (*R*)-3-((1*S*,4*S*)-6-Methoxy-4,7-dimethyl-5,8-bis((triisopropylsilyl)oxy)-1,4-dihydronaphthalen-1-yl)butanoate (**(7epi)-8**)

A 25 mL Schlenk flask was charged with CuCI (37 mg, 0.38 mmol, 0.75 equiv), and compound **21** (310 mg, 0.50 mmol, 1equiv) in 8 mL of dry THF was added. The pale-yellow solution was cooled to −42 °C, and TMSCl (140 μL, 1.11 mmol, 2.2 equiv) was added. After 5 min, MeMgBr (3.0 M in Et_2_O, 0.67 mL, 2.01 mmol, 4 equiv) was added dropwise. The color changed from yellow to brown over green to red and was orange yellow after full addition. The reaction was allowed to slowly warm to −22 °C over 1 h at which point TLC (petroleum ether/ethyl acetate, 20:1) confirmed full conversion. It was quenched by addition of saturated NH_4_Cl solution and saturated NaHCO_3_ solution and stirred for 1 h and 30 min. The aqueous layer was extracted three times with ethyl acetate, and the combined organic layer was washed three times with H_2_O, followed by brine. It was dried over MgSO_4_ and concentrated in vacuo. The desired product was obtained as yellow solids in virtually quantitative yield (317 mg, 0.50 mmol). ^1^H-NMR (400 MHz, CDCl_3_): δ = 5.85 (ddd, *J* = 10.4, 3.6, 1.5 Hz, 1H), 5.71 (ddd, *J* = 10.4, 4.3, 1.8 Hz, 1H), 3.64–3.60 (m, 4H), 3.53 (s, 3H), 3.49–3.42 (m, 1H), 2.59 (m, 1H), 2.17 (s, 3H), 1.76 (dd, *J* = 15.5, 11.6 Hz, 1H), 1.65 (dd, *J* = 15.5, 3.5 Hz, 1H), 1.36–1.24 (m, 6H), 1.20 (d, *J* = 6.7 Hz, 3H), 1.13–1.02 (m, 36H). ^13^C{^1^H}-NMR (101 MHz, CDCl_3_): δ = 147.6, 148.5, 147.0, 141.3, 132.6, 130.6, 123.6, 121.0, 120.7, 60.5, 51.2, 40.9, 35.9, 34.2, 31.4, 24.5, 18.4, 18.3, 18.2, 18.1, 18.1, 14.6, 14.0, 11.5; HRMS (ESI): exact mass calculated for C_35_H_61_O_5_Si_2_ [(M – CH_3_)^−^], 617.4063; found, 617.4056; [*α*]_D_^20^ = +345.84 (c 0.42, CH_2_Cl_2_).

### 1-((1*S*,4*S*)-6-Methoxy-4,7-dimethyl-5,8-bis((triisopropylsilyl)oxy)-1,4-dihydronaphthalen-1-yl)ethan-1-one (**23**)

A 25 mL round-bottom flask was charged with compound **9** (152 mg, 0.271 mmol, 1 equiv) in 9 mL of dimethylacetamide, 3 mL of 1,2-dichlorethane, and 0.75 mL of H_2_O. Bis(acetonitrile)dichloropalladium(II) (25 mg, 95 μmol, 35 mol %) and 1,4-benzoquinone (323 mg, 2.99 mmol, 11 equiv) were added, and the mix was heated to 36 °C. After 5 h, TLC (petroleum ether/ethyl acetate, 20:1) confirmed full conversion. The mixture was diluted with Et_2_O, and the organic layer was washed with NaHCO_3_ (5×; at which point the aqueous layer lost all dark brown coloration) followed by H_2_O and brine. It was dried over MgSO_4_, filtered, and concentrated in vacuo. The crude material was flashed over silica, and the desired product was obtained as a pale pink oil in 92% yield (144 mg, 0.251 mmol). ^1^H-NMR (600 MHz, CDCl_3_): δ = 5.96 (ddd, *J* = 10.0, 4.3, 2.6 Hz, 1H), 5.55 (ddd, *J* = 10.0, 3.8, 1.4 Hz, 1H), 4.30–4.26 (m, 1H), 3.64 (s, 3H), 3.62–3.57 (m, 1H), 2.18 (s, 3H), 1.63 (s, 3H), 1.38–1.29 (m, 6H), 1.24 (d, *J* = 7.1 Hz, 3H), 1.12–0.99 (m, 36H); ^13^C{^1^H}-NMR (151 MHz, CDCl_3_): δ = 208.8, 149.5, 148.0, 141.8, 133.5, 129.9, 121.4, 120.5, 119.8, 60.5, 52.6, 30.8, 25.1, 24.4, 18.3, 18.2, 18.1, 14.5, 14.1, 11.3; [*α*]_D_^20^ = +57.62 (c 1.74, CH_2_Cl_2_).

### 1-((1*S*,4*S*)-6-Methoxy-4,7-dimethyl-5,8-bis((triisopropylsilyl)oxy)-1,2,3,4-tetrahydronaphthalen-1-yl)ethan-1-one (**24**)

A 50 mL Schlenk flask was charged with compound **23** (144 mg, 0.251 mmol, 1 equiv) in 15 mL of dry degassed DCM, and Crabtree’s catalyst (14 mg, 18 μmol, 7 mol %) was added. The flask was evacuated and backfilled eight times with H_2_, causing a color change from orange to yellow. The H_2_ valve was closed, and the reaction was stirred at room temperature. After 1 h, TLC (petroleum ether/ethyl acetate, 50:1) confirmed full conversion. The yellow solution was filtered through silica, and solvents were evaporated. The desired product was obtained as a colorless oil in 99% yield (143 mg, 0.248 mmol). ^1^H-NMR (400 MHz, CDCl_3_): δ = 3.72 (dd, *J* = 6.3, 2.0 Hz, 1H), 3.64 (s, 3H), 3.23–3.15 (m, 1H), 2.18 (m, 4H), 2.03–1.92 (m, 1H), 1.89 (s, 3H), 1.64–1.49 (m, 2H), 1.41–1.27 (m, 6H), 1.28–1.22 (m, 1H), 1.16 (d, *J* = 6.9 Hz, 3H), 1.13–1.00 (m, 36H); ^13^C{^1^H}-NMR (101 MHz, CDCl_3_): δ = 213.2, 149.0, 148.0, 142.0, 132.6, 121.7, 119.9, 60.6, 47.9, 29.6, 28.1, 26.7, 22.0, 21.6, 18.3, 18.2, 18.0, 18.0, 14.5, 14.1, 11.4; HRMS (ESI): exact mass calculated for C_33_H_61_O_4_Si_2_ [(M + H)^+^], 577.4108; found, 577.4105; [*α*]_D_^20^ = +17.9 (c 0.80, CH_2_Cl_2_).

### Methyl (*Z*)-3-((1*R*,4*S*)-6-Methoxy-4,7-dimethyl-5,8-bis((triisopropylsilyl)oxy)-1,4-dihydronaphthalen-1-yl)but-2-enoate (***Z*-22**) and Methyl (*E*)-3-((1*R*,4*S*)-6-Methoxy-4,7-dimethyl-5,8-bis((triisopropylsilyl)oxy)-1,4-dihydronaphthalen-1-yl)but-2-enoate (***E*-22**)

A 10 mL Schlenk flask was charged with CuCl (9.0 mg, 91 μmol, 0.5 equiv), and compound **21** (112 mg, 0.182 mmol, 1 equiv)/TMSCl (51 μL, 0.399 mmol, 2.2 equiv) in 1.5 mL of dry THF was added. The yellow solution with reddish copper salt was cooled to −50 °C, and after 5 min, MeMgBr (3.0 M, 0.30 mL, 0.91 mmol, 5 equiv) was added dropwise over 3 min. A color change was observed, from strong dark red after the first drop over green and pale green to orange. After full addition, the reaction mixture was yellow. The reaction mixture was allowed to slowly warm up to −20 °C, and after 45 min, TLC (petroleum ether/ethyl acetate, 20:1) confirmed full conversion. A small sample was taken for TLC reference, and then PhSeCl (80 mg, 0.417 mmol, 2.3 equiv) dissolved in 0.5 mL of dry THF was added, causing a color change to strong green. TLC (petroleum ether/ethyl acetate, 20:1) moments after addition already confirmed full conversion. The reaction was quenched by addition of saturated NH_4_Cl solution and NaHCO_3_ solution. The mix was stirred for 1 h before layers were separated. The aqueous layer was extracted three times with ethyl acetate. The combined organic layer was washed with H_2_O and brine and dried over MgSO_4_. Solvents were removed in vacuo, and the crude material was subjected to column chromatography. The desired product was collected as a yellow oil in 70% yield (100 mg, 0.127 mmol) as an inseparable 1:1 mixture of diastereomers. The material was directly subjected to the next reaction. A 50 mL round-bottom flask was charged with selenide (112 mg, 0.142 mmol, 1 equiv) in 2 mL of CHCl_3_, and pyridine (23 μL, 0.284 mmol, 2 equiv) was added followed by H_2_O_2_ (35% in H_2_O, 86 μL, 0.995 mmol, 7 equiv). The yellow solution was stirred at room temperature for 30 min, at which point TLC (petroleum ether/diethyl ether, 24:1) confirmed full conversion. The reaction was quenched by addition of saturated Na_2_S_2_O_3_ solution, and the aqueous layer was extracted three times with DCM. The combined organic layer was washed with saturated NaHCO_3_ solution and H_2_O. It was dried over MgSO_4_ and concentrated in vacuo. The crude material was purified by column chromatography, and two products were collected. A *Z*-isomer (***Z*-22**) (eluting first) was obtained in 17% yield (17 mg, 27 μmol), and an *E*-isomer (***E*-22**) (eluting second) was obtained in 29% yield (30 mg, 48 μmol). *Z*-isomer (***Z*-22**): ^1^H-NMR (600 MHz, CDCl_3_): δ = 5.81 (ddd, *J* = 10.0, 4.1, 2.3 Hz, 1H), 5.70–5.65 (m, 2H), 5.47 (dq, *J* = 4.1, 2.2, 1.7 Hz, 1H), 3.70 (s, 3H), 3.63 (s, 3H), 3.56–3.50 (m, 1H), 2.17 (s, 3H), 1.36–1.24 (m, 9H), 1.22 (d, *J* = 6.7 Hz, 3H), 1.11–1.04 (m, 27H), 1.00–0.94 (m, 9H); ^13^C{^1^H}-NMR (151 MHz, CDCl_3_): δ = 166.9, 165.4, 148.7, 147.9, 141.3, 130.8, 130.4, 124.8, 122.2, 120.0, 114.8, 60.5, 50.8, 39.4, 30.7, 24.9, 22.0, 18.4, 18.2, 18.1, 18.1, 14.4, 14.0, 11.5; *E*-isomer (***E*-22**): ^1^H-NMR (600 MHz, CDCl_3_): δ = 5.83 (ddd, *J* = 10.1, 3.9, 2.0 Hz, 1H), 5.58–5.57 (m, 1H), 5.55 (ddd, *J* = 10.0, 4.3, 1.7 Hz, 1H), 4.27 (ddt, *J* = 4.1, 2.8, 1.4 Hz, 1H), 3.63 (s, 3H), 3.63 (s, 3H), 3.56–3.53 (m, 1iH), 2.15 (s, 3H), 1.76 (d, *J* = 1.3 Hz, 3H), 1.37–1.27 (m, 6H), 1.24 (d, *J* = 6.7 Hz, 3H), 1.12–1.00 (m, 36H); ^13^C{^1^H}-NMR (151 MHz, CDCl_3_): δ = 167.8, 162.9, 149.1, 147.8, 141.5, 131.7, 130.6, 124.1, 120.8, 120.5, 115.7, 60.5, 50.8, 46.4, 31.1, 24.6, 18.4, 18.3, 18.1, 15.9, 14.7, 14.0, 11.4; HRMS (ESI): exact mass calculated for C_35_H_61_O_5_Si_2_ [(M–CH_2_ + H)^+^], 617.4052; found, 617.4033; [*α*]_D_^20^ = +155.91 (c 1.35, CH_2_Cl_2_).

### (5*S*,8*S*)-3-Methoxy-2,5-dimethyl-4-((triisopropylsilyl)oxy)-8-vinyl-5,8-dihydronaphthalen-1-ol (**25**)

Acetic acid (219.66 mg, 3.66 mmol) was added to a solution of compound **9** (838 mg, 1.50 mmol) in THF (20.0 mL), and the mixture was cooled to 0 °C. After addition of TBAF (1.0 M in THF, 1.50 mL, 1.50 mmol) over a period of 10 min, the solution turned yellow and was stirred at rt for 1 h and 50 min until TLC showed formation of the hydroquinone. The reaction was stopped by adding 5.0 mL of water and 100 mL of ethyl acetate. The organic phase was washed with water and brine. The solvents were removed in vacuo. The crude mixture was separated by column chromatography, giving 40% (338 mg, 605 μmol) of the desired product and 54% (324 mg, 807 μmol) starting material. ^1^H-NMR (400 MHz, CDCl_3_): δ = 5.86 (ddd, *J* = 10.0, 4.3, 2.3 Hz, 1H), 5.70 (ddd, *J* = 17.4, 9.6, 9.4 Hz, 1H), 5.52 (ddd, *J =* 10.1, 3.4, 1.4 Hz, 1H), 5.35 (d, *J* = 17.0 Hz, 1H), 5.22 (dd, *J* = 9.3, 1.9 Hz, 1H), 5.21 (s, 1H), 3.99–3.92 (1H, m), 3.67 (s, 3H), 3.59–3.49 (m, 1H), 2.15 (s, 3H), 1.18–1.28 (m, 3H), 1.26 (d, *J =* 6.8 Hz, 3H), 1.11–1.04 (m, 18H); ^13^C{^1^H}-NMR (101 MHz, CDCl_3_): δ = 149.2, 146.9, 142.5, 140.9, 130.8, 129.1124.5, 117.3, 116.8116.1, 60.8, 40.8, 30.5, 23.9, 18.3, 18.2, 14.0, 9.3. [*α*]_D_^20^ = +123.184° (c 0.90, CH_2_Cl_2_).

### (5*S*,8*S*)-3-Methoxy-2,5-dimethyl-4-((triisopropylsilyl)oxy)-8-vinyl-5,8-dihydronaphthalen-1-yl Acrylate (**26**)

A slurry of NaH (55% in mineral oil, 18 mg, 411 μmol) in THF (3.0 mL) was cooled to −23 °C. Then, compound **25** (83 mg, 205 μmol) in THF (1.0 mL) was added slowly over 20 min and the mixture was stirred for 6 min. Then, acryloyl chloride (37 mg, 411 μmol, 33 μL) was added dropwise over 5 min. The solution became brighter. After 1 h and 10 min at −20 °C, TLC confirmed full conversion of the starting material. The reaction mixture was quenched by adding a spatula of NaHCO_3_. Then, the solution was diluted with ethyl and water and the phases were separated. The aqueous layer was extracted with ethyl acetate. The combined organic phases were washed with water and brine. The solution was dried over Na_2_SO_4_, and the solvents evaporated, giving 95 mg of crude material. The crude material was purified over a plug of silica, and the desired product was isolated as a colorless oil in 83% yield (78 mg, 171 μmol). ^1^H-NMR (600 MHz, CDCl_3_): δ = 6.57 (broad d, *J =* 18.2 Hz, 1H), 6.29 (broad dd, *J =* 17.1, 9.7 Hz, 1H), 5.98 (broad d, *J =* 7.6 Hz, 1H), 5.77 (ddd, *J =* 10.1, 3.9, 1.9 Hz, 1H), 5.71–5.53 (broad m, 1H), 5.50 (ddd, *J =* 10.2, 3.6, 1.6 Hz, 2H), 4.91 (broad d, *J =* 16.9 Hz, 2H), 3.92–3.57 (m, 1H), 3.69 (s, 3H), 3.57–3.50 (m, 1H), 2.02 (broad s, 3H), 1.33 (m, *J =* 7.5 Hz, 3H), 1.28 (d, *J =* 7.3 Hz, 3H), 1.09 (d, *J =* 7.6 Hz, 9H), 1.06 (d, *J =* 7.6 Hz, 9H); ^13^C{^1^H}-NMR (151 MHz, CDCl_3_): δ = 164.1, 148.7, 148.6, 145.3, 141.9, 141.6, 132.6, 130.1, 129.6, 128.0, 125.3, 124.2, 122.6, 113.2, 60.9, 40.3, 30.5, 23.8, 18.2, 18.1, 14.0, 10.0; [*α*]_D_^20^ = +3.746° (c 0.37, CH_2_Cl_2_).

### (4a*R*,7*S*)-9-Methoxy-7,10-dimethyl-8-((triisopropylsilyl)oxy)-4a,7-dihydro-2*H*-naphtho[1,8-*bc*]oxepin-2-one (**27**)

A solution of compound **26** (225 mg, 0.497 mmol) in degassed (freeze–pump–thaw, three cycles) toluene (30 mL) was heated to 80 °C. Then, a solution of Hoveyda–Grubbs second-generation catalyst (6 mg, 11 μmol) in toluene (0.5 mL) was added dropwise over 3.5 h to the starting material (syringe pump). After complete addition, the reaction was stirred for 15 h at 80 °C. Then, another portion of the catalyst (1.5 mg) was added, and the solution was stirred for an additional 3 h. The reaction was quenched by bubbling air through the solution for 40 min. Then, toluene was evaporated to obtain 210 mg of the crude product, which was purified via chromatography using toluene and diethyl ether as eluents (very fast filtration to avoid decomposition and isomerization). The desired product was obtained in 98% yield (198 mg, 462 μmol). ^1^H-NMR (400 MHz, CDCl_3_): δ = 6.51 (dd, *J =* 4.5 Hz, 10.9 Hz, 1H), 6.51 (dd, *J =* 4.5 Hz, 10.9 Hz, 1H), 5.95 (ddd, *J =* 2.2 Hz, 4.2 Hz, 10.2 Hz, 1H), 5.80 (ddd, *J =* 1.1 Hz, 3.5 Hz, 10.1 Hz, 1H), 5.76 (dd, *J =* 2.4 Hz, 10.9 Hz, 1H), 4.38–4.32 (m, 1H), 3.67 (s, 3H), 3.56–3.48 (m, 1H), 2.26 (s, 3H), 1.38–1.27 (m, 3H), 1.26 (d, *J =* 6.6 Hz, 3H), 1.07 (d, *J =* 7.5 Hz, 9H), 1.04 (d, *J =* 7.5 Hz, 9H); ^13^C{^1^H}-NMR (101 MHz, CDCl_3_): δ = 164.1, 154.3, 148.6, 144.6, 143.7, 131.6, 128.4, 124.8, 122.7, 122.6, 119.4, 60.9, 34.5, 29.8, 23.6, 18.2, 18.1, 14.0, 10.3.

### 3-Methoxy-2-methyl-6-(penta-1,4-dien-3-yl)-4-((triisopropylsilyl)oxy)phenol (**34**)

A 100 mL Schlenk flask was charged with compound **15** (1.77 g, 4.7 mmol, 1 equiv) and azeotropically dried three times with toluene prior to use. Then, 40 mL of dry toluene was added, and the pale-yellow solution was degassed by applying the Schlenk technique. Subsequently, EuFOD (150 mg, 3 mol %) was added in one portion and the reaction mixture was degassed again. The flask was placed in a 110 °C oil bath and heated for 4 h, upon which TLC (petroleum ether/ethyl acetate, 10:1) confirmed full conversion. The reaction mixture was transferred into a separation funnel, washed with H_2_O (5×), followed by brine (5×), and dried over MgSO_4_. It was concentrated under reduced pressure, and the crude oil was purified by flash chromatography. Pure phenol was obtained as a yellow oil in 97% yield (1.72 g, 4.56 mmol). ^1^H-NMR (400 MHz, CDCl_3_): δ = 6.52 (s, 1H), 6.04 (ddd, *J* = 17.3, 10.3, 6.2 Hz, 2H), 5.24 (dt, *J* = 10.3, 1.5 Hz, 2H), 5.14 (dt, *J* = 17.3, 1.6 Hz, 2H), 4.75 (s, 1H), 4.22–4.14 (m, 1H), 3.77 (s, 4H), 2.18–2.12 (m, 3H), 1.29–1.19 (m, 3H), 1.10 (d, *J* = 7.4 Hz, 18H); ^13^C{^1^H}-NMR (101 MHz, CDCl_3_): δ = 148.4, 146.3, 142.9, 138.7, 122.1, 119.7, 117.6, 116.4, 60.3, 47.6, 18.1, 12.9, 9.4; IR [ATR]: ν = 3511, 2945, 2867, 1635, 1482, 1438, 1390, 1343, 1231, 1119, 1056, 997, 917, 881, 811, 760, 733 cm^–1^; HRMS (ESI): exact mass calculated for C_22_H_37_O_3_Si [(M + H)^+^], 377.2506; found, 377.2509.

### 3-Methoxy-2-methyl-6-(penta-1,4-dien-3-yl)-4-((triisopropylsilyl)oxy)phenyl acrylate (**33**)

A 250 mL Schlenk flask was charged with compound **34** (5.59 g, 15 mmol, 1 equiv) in 100 mL of dry DCM. The clear yellow solution was cooled in an ice bath, and Et_3_N (6.2 mL, 45 mmol, 3 equiv) was added, causing a color change to orange. Dropwise addition of acryloyl chloride (1.45 mL, 18 mmol, 1.2 equiv) caused a color change to strong orange. After 2 h, TLC (petroleum ether/ethyl acetate, 10:1) confirmed full consumption of the starting material. The reaction was quenched by addition of saturated NH_4_Cl solution, and layers were separated. The aqueous layer was extracted three times with DCM. The combined organic layer was washed with H_2_O and brine. It was dried over MgSO_4_ and concentrated in vacuo. The crude orange syrup was flashed over silica (petroleum ether/ethyl acetate, 10:1). The pure product was obtained as a colorless to pale yellow oil in 93% yield (5.94 g, 14 mmol). ^1^H-NMR (400 MHz, CDCl_3_): δ = 6.63–6.57 (m, 2H), 6.33 (dd, *J* = 17.3, 10.4 Hz, 1H), 6.01 (dd, *J* = 10.4, 1.3 Hz, 1H), 5.89 (ddd, *J* = 17.2, 10.3, 6.2 Hz, 2H), 5.12 (dt, *J* = 10.3, 1.5 Hz, 2H), 5.01 (dt, *J* = 17.3, 1.6 Hz, 2H), 4.06–4.00 (m, 1H), 3.79 (s, 3H), 2.04 (d, *J* = 0.5 Hz, 3H), 1.34–1.18 (m, 3H), 1.10 (d, *J* = 7.3 Hz, 18H); ^13^C{^1^H}-NMR (101 MHz, CDCl_3_): δ = 164.2, 148.0, 147.1, 140.9, 138.9, 132.6, 129.4, 127.8, 125.6, 117.3, 115.9, 60.3, 46.3, 18.1, 12.9, 10.2; IR [ATR]: ν = 2945, 2868, 1746, 1634, 1587, 1482, 1436, 1402, 1341, 1292, 1231, 1203, 1148, 1112, 1061, 1016, 997, 917, 905, 881, 803, 771, 734 cm^–1^; HRMS (ESI): exact mass calculated for C_25_H_39_O_4_Si [(M + H)^+^], 431.2612; found, 431.2600.

### 8-Methoxy-9-methyl-7-((triisopropylsilyl)oxy)-5-vinylbenzo[*b*]oxepin-2(5*H*)-one (**(rac)-35**)

Compound **33** (1.53 g, 3.55 mmol, 1 equiv) was azeotropically dried three times with toluene prior to use. It was dissolved in 360 mL of dry toluene, and two freeze–thaw cycles were carried out. Subsequently, a Grubbs–Hoveyda second-generation catalyst (67 mg, 0.11 mmol, 3 mol %) was added and the yellow mixture was heated to 80 °C for 15 h. The next day, TLC (petroleum ether/ethyl acetate, 10:1) confirmed full conversion. The reaction was removed from heating and allowed to cool to room temperature. Then, the flask was opened and stirred on air for approximately 1 h. The mixture was quickly flashed over a plug of silica to remove catalyst residues. Note that fast purification is necessary to prevent isomerization of the α,β-double bond into conjugation with the aromatic system and the methylene group. The desired product was subjected to the next reaction without further purification. ^1^H-NMR (400 MHz, CDCl_3_): δ = 6.85 (dd, *J* = 11.1, 6.8 Hz, 1H), 6.51 (s, 1H), 6.03 (ddd, *J* = 17.2, 10.3, 7.0 Hz, 1H), 5.90 (dd, *J* = 11.0, 1.1 Hz, 1H), 5.30 (dt, *J* = 10.2, 1.1 Hz, 1H), 5.23 (dt, *J* = 17.2, 1.2 Hz, 1H), 4.20–4.14 (m, 1H), 3.76 (s, 3H), 2.27 (s, 3H), 1.30–1.20 (m, 3H), 1.10 (d, *J* = 7.0 Hz, 18H); ^13^C{^1^H}-NMR (101 MHz, CDCl_3_): δ = 163.8, 149.8, 148.5, 146.5, 143.2, 134.6, 129.7, 125.6, 120.7, 117.9, 115.5, 60.4, 44.7, 18.0, 12.9, 10.3; IR [ATR]: ν = 2944, 2867, 1730, 1589, 1482, 1436, 1383, 1347, 1248, 1202, 1168, 1110, 1061, 1000, 919, 881, 808, 732 cm^–1^; HRMS (ESI): exact mass calculated for C_23_H_35_O_4_Si [(M + H)^+^], 403.2299; found, 403.2283.

### 8-Methoxy-4,9-dimethyl-7-((triisopropylsilyl)oxy)-5-vinyl-4,5-dihydrobenzo[*b*]oxepin-2(3*H*)-one (**(rac)-36**)

A 100 mL Schlenk flask was charged with CuCl (35 mg, 0.39 mmol, 5 mol %) and LiCl (30 mg, 0.68 mmol, 10 mol %), and compound **(rac)-35** (2.81 g, 6.98 mmol, 1 equiv) in 46 mL of dry THF was added followed by dropwise addition of TMSCl (0.98 mL, 7.68 mmol, 1.1 equiv). The orange mixture was cooled to −40 °C, and after 20 min, MeMgBr (3.0 M in ether, 2.79 mL, 8.38 mmol, 1.2 equiv) was added dropwise over 5 min. After 10 min, TLC (petroleum ether/ethyl acetate, 10:1) confirmed full conversion. The reaction was quenched by addition of saturated NaHCO_3_ solution and saturated NH_4_Cl solution. The blue solution was allowed to warm to room temperature and stirred for 15 min. The aqueous layer was extracted three times with ethyl acetate, and the combined organic layer was washed with H_2_O and brine. It was dried over MgSO_4_, filtered, and concentrated in vacuo. The crude orange oil was subjected to column chromatography (petroleum ether/ethyl acetate, 25:1), and the desired product was obtained as a yellow oil in 42% yield (1.24 g, 2.96 mmol) over two steps. The material crystallized in the freezer. ^1^H-NMR (400 MHz, CDCl_3_): δ = 6.58 (s, 1H), 5.92 (ddd, *J* = 17.2, 10.3, 7.9 Hz, 1H), 5.30 (dt, *J* = 10.4, 1.1 Hz, 1H), 5.17 (dt, *J* = 17.2, 1.3 Hz, 1H), 3.78 (s, 3H), 3.19–3.10 (m, 1H), 2.59 (dd, *J* = 11.9, 7.2 Hz, 1H), 2.30–2.12 (m, 6H), 1.32–1.20 (m, 3H), 1.17 (d, *J* = 6.7 Hz, 3H), 1.13–1.05 (m, 18H); ^13^C{^1^H}-NMR (101 MHz, CDCl_3_): δ = 171.0, 148.3, 146.4, 143.5, 137.2, 127.8, 123.6, 118.4, 116.2, 60.4, 49.1, 38.4, 38.2, 19.8, 18.0, 12.9, 10.0; IR [ATR]: ν = 2945, 2867, 1764, 1587, 1481, 1438, 1384, 1346, 1290, 1239, 1208, 1171, 1131, 1114, 1061, 1027, 999, 943, 917, 882, 858, 818, 797, 785, 762, 731 cm^–1^; HRMS (ESI): exact mass calculated for C_24_H_39_O_4_Si [(M + H)^+^], 419.2612; found, 419.2635.

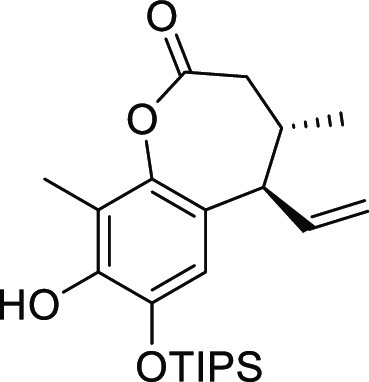



### 8-Hydroxy-4,9-dimethyl-7-((triisopropylsilyl)oxy)-5-vinyl-4,5-dihydrobenzo[*b*]oxepin-2(3*H*)-one (**(rac)-S6**)

A 25 mL Schlenk flask was charged with AlCl_3_ (283 mg, 2.12 mmol, 3.21 equiv) in 4 mL of DCM. To the mixture was added dimethyl sulfide (0.15 mL, 1.98 mmol, 3 equiv), causing the solid AlCl_3_ to dissolve. After 20 min, compound **(rac)-36** (277 mg, 0.66 mmol, 1 equiv) dissolved in 4 mL of dry DCM was added dropwise. After 30 min, TLC (petroleum ether/ethyl acetate, 12:1) confirmed full conversion. The reaction was quenched with saturated NaHCO_3_ solution and carefully brought to pH = 7. The color changed from strong dark brown to orange. The aqueous layer was extracted three times with DCM. The combined organic layer was washed with H_2_O and brine. It was dried over MgSO_4_, filtered, and concentrated in vacuo. The crude oil was flashed over a plug of silica, and the desired product was obtained as a red oil in 89% yield (238 mg, 0.59 mmol). ^1^H-NMR (400 MHz, CDCl_3_): δ = 6.55 (s, 1H), 5.91 (ddd, *J* = 17.2, 10.4, 7.8 Hz, 1H), 5.69 (s, 1H), 5.30 (dt, *J* = 10.4, 1.2 Hz, 1H), 5.16 (dt, *J* = 17.3, 1.3 Hz, 1H), 3.14 (dd, *J* = 9.3, 7.7 Hz, 1H), 2.59 (dd, *J* = 11.9, 7.3 Hz, 1H), 2.29–2.11 (m, 5H), 1.37–1.23 (m, 3H), 1.16 (d, *J* = 6.7 Hz, 3H), 1.11 (dd, *J* = 7.4, 3.1 Hz, 18H); ^13^C{^1^H}-NMR (101 MHz, CDCl_3_): δ = 171.1, 144.6, 143.9, 139.6, 137.4, 123.0, 118.2, 115.8, 113.1, 48.9, 38.6, 38.2, 19.8, 18.0, 12.8, 9.5; IR [ATR]: ν = 3531, 2944, 2867, 1762, 1611, 1488, 1462, 1440, 1383, 1351, 1316, 1245, 1203, 1171, 1131, 1113, 1053, 1025, 999, 946, 919, 881, 856, 803, 787, 743, 683, 647 cm^–1^; HRMS (ESI): exact mass calculated for C_23_H_37_O_4_Si [(M + H)^+^], 405.2456; found, 405.2476.

### 8-(Methoxymethoxy)-4,9-dimethyl-7-((triisopropylsilyl)oxy)-5-vinyl-4,5-dihydrobenzo[*b*]oxepin-2(3*H*)-one (**(rac)-32**)

A 50 mL Schlenk flask was charged with NaI (163 mg, 1.09 mmol, 20 mol %) and compound **(rac)-S6** (2.20 g, 5.44 mmol, 1 equiv) dissolved in 25 mL of dry THF. The red solution was cooled to −35 °C, and LiHMDS (1.0 M, 6.0 mL, 6.0 mmol, 1.1 equiv) was added followed by MOMCl (0.58 mL, 7.61 mmol, 1.4 equiv). The reaction was allowed to slowly warm up. After 25 min at −26 °C, TLC (petroleum ether/ethyl acetate, 10:1) confirmed full conversion. The reaction was quenched with saturated NaHCO_3_ solution and allowed to warm to room temperature. The aqueous layer was extracted four times with Et_2_O. The combined organic layer was washed with little H_2_O and brine. It was dried over MgSO_4_, filtered, and concentrated in vacuo. The crude orange-red oil was flashed over silica (25 g; petroleum ether/ethyl acetate, 20:1), and the desired product was collected as an orange oil in 94% yield (2.30 g, 5.13 mmol). ^1^H-NMR (400 MHz, CDCl_3_): δ = 6.58 (s, 1H), 5.91 (ddd, *J* = 17.2, 10.4, 8.0 Hz, 1H), 5.31 (dt, *J* = 10.5, 1.1 Hz, 1H), 5.20–5.08 (m, 3H), 3.57 (s, 3H), 3.19–3.10 (m, 1H), 2.60 (dd, *J* = 11.9, 7.2 Hz, 1H), 2.24 (s, 3H), 2.22 (m, 2H), 1.29–1.20 (m, 3H), 1.17 (d, *J* = 6.7 Hz, 3H), 1.08 (dd, *J* = 7.4, 3.0 Hz, 18H); ^13^C{^1^H}-NMR (101 MHz, CDCl_3_): δ = 170.9, 145.8, 145.7, 143.4, 137.1, 128.0, 124.1, 118.5, 115.9, 99.1, 57.7, 49.0, 38.4, 38.2, 19.8, 18.0, 13.0, 10.7; IR [ATR]: ν = 2944, 2868, 1754, 1645, 1589, 1482, 1416, 1383, 1342, 1235, 1203, 1162, 1130, 1057, 964, 923, 881, 854 cm^–1^; HRMS (ESI): exact mass calculated for C_25_H_41_O_5_Si [(M + H)^+^], 449.2718; found, 449.2738.

### 7-Hydroxy-8-(methoxymethoxy)-4,9-dimethyl-5-vinyl-4,5-dihydrobenzo[*b*]oxepin-2(3*H*)-one (**(rac)-37**)

A 50 mL round-bottom flask was charged with compound **(rac)-32** (2.30 g, 5.13 mmol, 1 equiv) dissolved in 20 mL of dry THF. To the orange solution was added TBAF (1.0 M in THF, 5.38 mL, 5.38 mmol, 1.05 equiv), causing a color change to black. After 3 min, TLC (petroleum ether/ethyl acetate, 5:1) confirmed full conversion. The reaction was quenched by addition of saturated NH_4_Cl solution, and the aqueous layer was extracted three times with Et_2_O. The combined organic layer was washed with small portions of H_2_O (12×) until the aqueous layer became clear and as good as colorless. The orange organic phase was washed with brine, dried over MgSO_4_, filtered, and concentrated in vacuo. The crude material was flashed over a plug of silica (gradient: petroleum ether/ethyl acetate, 5:1, to pure ethyl acetate). The purified material was dried on high vacuum overnight, and the desired product was obtained as a yellow-orange oil in 77% yield (1.16 g, 3.97 mmol). ^1^H-NMR (400 MHz, CDCl_3_): δ = 7.45–7.02 (broad s, 1H), 6.69 (s, 1H), 5.95 (ddd, *J* = 17.1, 10.3, 8.2 Hz, 1H), 5.32–5.25 (m, 1H), 5.21–5.12 (m, 1H), 5.03 (d, *J* = 6.1 Hz, 1H), 4.98 (d, *J* = 6.1 Hz, 1H), 3.63 (s, 3H), 3.14 (t, *J* = 8.8 Hz, 1H), 2.58 (dd, *J* = 11.9, 7.2 Hz, 1H), 2.27–2.12 (m, 5H), 1.16 (d, *J* = 6.7 Hz, 3H). ^13^C{^1^H}-NMR (101 MHz, CDCl_3_): δ = 171.0, 146.2, 143.4, 142.5, 137.0, 129.6, 122.6, 118.6, 112.8, 99.9, 57.4, 49.3, 38.2, 38.0, 19.8, 10.3; IR [ATR]: ν = 3355, 2977, 2871, 1757, 1642, 1593, 1482, 1437, 1345, 1291, 1242, 1200, 1170, 1157, 1132, 1108, 1048, 966, 913, 856, 803, 730, 647 cm^–1^; HRMS (ESI): exact mass calculated for C_16_H_21_O_5_ [(M + H)^+^], 293.1384; found, 293.1384.

### (4*S*,5*R*)-7-(((*S*,*E*)-1-(Allyloxy)pent-3-en-2-yl)oxy)-8-(methoxymethoxy)-4,9-dimethyl-5-vinyl-4,5-dihydrobenzo[*b*]oxepin-2(3*H*)-one (**38**) and (4*R*,5*S*)-7-(((*S*,*E*)-1-(Allyloxy)pent-3-en-2-yl)oxy)-8-(methoxymethoxy)-4,9-dimethyl-5-vinyl-4,5-dihydrobenzo[*b*]oxepin-2(3*H*)-one (**38′**)

A 100 mL Schlenk flask was charged with compound **(rac)*-*37** (539 mg, 1.84 mmol, 1 equiv) and alcohol **12** (367 mg, 2.58 mmol, 1.4 equiv) in 12 mL of dry toluene. The mixture was cooled to 0 °C, and PBu_3_ (0.50 mL, 2.03 mmol, 1.1 equiv) was added. After 5 min, ADDP (512 mg, 2.03 mmol, 1.1 equiv) dissolved in 8 mL of dry toluene was added. Immediate solidification was observed. After 2 h, TLC (petroleum ether/ethyl acetate, 5:1) confirmed full conversion. The reaction was diluted with Et_2_O, causing OPBu_3_ to precipitate. The mixture was filtered over silica gel and Celite. It was concentrated in vacuo, and the oily residue was subjected to column chromatography (petroleum ether/ethyl acetate, 13:1). The desired product **38** was obtained as a colorless to pale yellow oil in 36% yield (278 mg, 0.667 mmol). Diastereomer **38′** was collected as a pale-yellow oil in 36% yield (277 mg, 0.665 mmol). Desired **38**: ^1^H-NMR (400 MHz, CDCl_3_): δ = 6.65 (s, 1H), 5.97–5.82 (m, 2H), 5.81–5.67 (m, 1H), 5.48 (ddq, *J* = 15.4, 7.2, 1.6 Hz, 1H), 5.32–5.22 (m, 2H), 5.20–5.08 (m, 4H), 4.66 (td, *J* = 7.0, 4.0 Hz, 1H), 4.02 (dt, *J* = 5.6, 1.5 Hz, 2H), 3.65 (dd, *J* = 10.4, 6.9 Hz, 1H), 3.59–3.53 (m, 5H), 3.14 (t, *J* = 8.7 Hz, 1H), 2.59 (dd, *J* = 12.0, 7.2 Hz, 1H), 2.22 (s, 4H), 2.17–2.11 (m, 1H), 1.68 (ddd, *J* = 6.5, 1.7, 0.7 Hz, 3H), 1.15 (d, *J* = 6.7 Hz, 3H); ^13^C{^1^H}-NMR (101 MHz, CDCl_3_): δ = 170.8, 147.8, 145.2, 143.7, 137.1, 134.6, 130.5, 127.7, 127.6, 123.7, 118.4, 117.2, 113.4, 99.2, 79.9, 73.0, 72.4, 57.6, 49.3, 38.3, 38.1, 19.8, 18.0, 10.5; IR [ATR]: ν = 2979, 2932, 2870, 1761, 1673, 1644, 1591, 1481, 1435, 1396, 1380, 1346, 1290, 1231, 1157, 1131, 1108, 1062, 1028, 964, 920, 863, 800, 757, 738, 707, 672, 576, 508 cm^–1^; HRMS (ESI): exact mass calculated for C_24_H_33_O_6_ [(M + H)^+^], 417.2272; found, 417.2278; [*α*]_D_^20^ = +60.1 (c 0.90, CH_2_Cl_2_); Diastereomer **38′**: ^1^H-NMR (400 MHz, CDCl_3_): δ = 6.66 (s, 1H), 5.97–5.84 (m, 2H), 5.75 (dtd, *J* = 15.6, 6.5, 1.0 Hz, 1H), 5.44 (ddq, *J* = 15.5, 7.2, 1.6 Hz, 1H), 5.33–5.23 (m, 2H), 5.23–5.09 (m, 4H), 4.76–4.67 (m, 1H), 4.09–4.01 (m, 2H), 3.67 (dd, *J* = 10.4, 7.0 Hz, 1H), 3.63–3.55 (m, 4H), 3.20–3.11 (m, 1H), 2.59 (dd, *J* = 11.9, 7.1 Hz, 1H), 2.28–2.11 (m, 5H), 1.67 (dd, *J* = 6.6, 0.9 Hz, 3H), 1.16 (d, *J* = 6.6 Hz, 3H); ^13^C{^1^H}-NMR (101 MHz, CDCl_3_): δ = 170.9, 147.7, 144.8, 143.5, 137.2, 134.7, 130.9, 127.6, 127.4, 123.7, 118.4, 117.3, 112.7, 99.1, 79.5, 73.0, 72.5, 57.6, 49.2, 38.4, 38.1, 19.8, 17.9, 10.5; IR [ATR]: ν = 2870, 1759, 1643, 1592, 1482, 1434, 1379, 1346, 1231, 1157, 1130, 1109, 1062, 964, 919, 863, 787, 731, 669, 647 cm^–1^; HRMS (ESI): exact mass calculated for C_24_H_33_O_6_ [(M + H)^+^], 417.2272; found, 417.2243; [*α*]_D_^20^ = −37.4 (c 1.75, CH_2_Cl_2_).

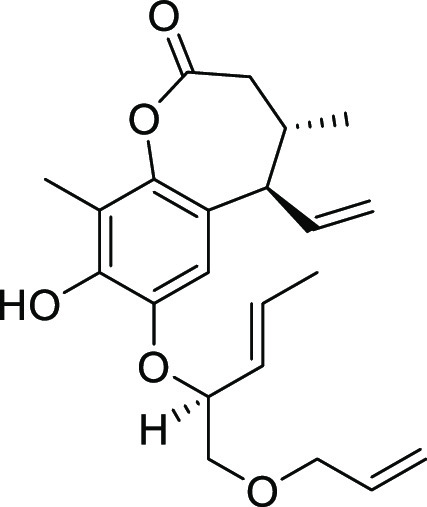



### (4*S*,5*R*)-7-(((*S*,*E*)-1-(Allyloxy)pent-3-en-2-yl)oxy)-8-hydroxy-4,9-dimethyl-5-vinyl-4,5-dihydrobenzo[*b*]oxepin-2(3*H*)-one (**S7**)

A 50 mL Schlenk flask was charged with MgBr_2_·Et_2_O (315 mg, 1.22 mmol, 1.07 equiv) and compound **38** (475 mg, 1.14 mmol, 1 equiv) in 15 mL of dry DCM. The mixture was cooled to 4 °C, and after 10 min, EtSH (0.18 mL, 2.39 mmol, 2.1 equiv) was added dropwise. After 30 min, the cooling bath was removed, and the reaction was stirred at room temperature. TLC (petroleum ether/ethyl acetate, 4:1) after an additional hour confirmed full conversion. The reaction was quenched by addition of saturated NaHCO_3_ solution, and layers were separated. The aqueous layer was extracted three times with DCM, and the combined organic layer was washed with H_2_O. It was dried over Na_2_SO_4_, filtered, and concentrated in vacuo. The crude residue was flashed over a plug of silica, and the desired product was obtained as an orange solid in 95% yield (404 mg, 1.08 mmol). ^1^H-NMR (400 MHz, CDCl_3_): δ = 7.65 (d, *J* = 1.1 Hz, 1H), 6.65 (s, 1H), 6.03–5.85 (m, 2H), 5.79–5.66 (m, 1H), 5.52 (ddq, *J* = 15.3, 7.8, 1.6 Hz, 1H), 5.34 (dq, *J* = 17.2, 1.5 Hz, 1H), 5.26 (m, 2H), 5.15 (dt, *J* = 17.2, 1.3 Hz, 1H), 4.20–4.12 (m, 3H), 3.65 (dd, *J* = 10.2, 9.3 Hz, 1H), 3.56–3.48 (m, 1H), 3.11 (t, *J* = 8.9 Hz, 1H), 2.59 (dd, *J* = 11.9, 7.3 Hz, 1H), 2.22–2.11 (m, 6H), 1.73 (dd, *J* = 6.5, 1.6 Hz, 3H), 1.15 (d, *J* = 6.6 Hz, 3H); ^13^C{^1^H}-NMR (101 MHz, CDCl_3_): δ = 171.0, 147.2, 146.0, 142.5, 137.4, 133.8, 131.9, 126.6, 122.7, 118.4, 118.1, 117.2, 116.7, 85.0, 72.6, 72.5, 48.9, 38.6, 38.1, 19.7, 18.0, 9.5; IR [ATR]: ν = 3297, 2976, 2932, 2870, 1746, 1667, 1639, 1609, 1482, 1445, 1419, 1375, 1345, 1317, 1243, 1226, 1196, 1175, 1144, 1131, 1108, 1090, 1056, 1024, 999, 972, 937, 919, 881, 867, 841, 801, 743, 680, 622 cm^–1^; HRMS (ESI): exact mass calculated for C_22_H_29_O_5_ [(M + H)^+^], 373.2010; found, 373.2016; [*α*]_D_^20^ = +107.1 (c 1.0, CH_2_Cl_2_).

### (4*S*,5*R*)-7-(((*S*,*E*)-1-(Allyloxy)pent-3-en-2-yl)oxy)-4,9-dimethyl-8-((triethylsilyl)oxy)-5-vinyl-4,5-dihydrobenzo[*b*]oxepin-2(3*H*)-one (**31**)

A 50 mL Schlenk flask was charged with compound **S7** (325 mg, 0.87 mmol, 1 equiv) in 5 mL of dry DCM. The yellow solution was cooled to −85 °C, and 2,6-lutidine (0.30 mL, 2.62 mmol, 3 equiv) was added followed by TESOTf (0.30 mL, 1.31 mmol, 1.5 equiv). After 10 min, the reaction mix was removed from the cooling bath and stirred at room temperature. TLC (petroleum ether/ethyl acetate, 5:1) after 15 min confirmed full conversion. The reaction was quenched with saturated NaHCO_3_ solution, and the aqueous layer was extracted five times with DCM. The combined organic layer was dried over MgSO_4_ and concentrated in vacuo. The material was used in the next reaction without further purification. ^1^H-NMR (400 MHz, CDCl_3_): δ = 6.58 (s, 1H), 5.98–5.84 (m, 2H), 5.82–5.72 (m, 1H), 5.48 (ddd, *J =* 15.6, 7.0, 1.6, 1H), 5.31–5.22 (m, 2H), 5.20–5.11 (m, 2H), 4.74 (m, 1H), 4.02 (d, *J =* 5.5, 2H), 3.68 (dd, *J =* 10.0, 6.4, 1H), 3.56 (dd, *J =* 10.1, 4.7, 1H), 3.14 (t, *J =* 8.6, 1H), 2.54 (dd, *J =* 11.9, 7.2, 1H), 2.23–2.08 (m, 6H), 2.13 (s, 3H), 1.70 (dd, *J =* 6.6, 0.8, 3H), 1.15 (d, *J =* 6.64, 3H), 0.96 (t, *J =* 8.0, 9H), 0.80–0.72 (m, 6H); ^13^C{^1^H}-NMR (101 MHz, CDCl_3_): δ = 171.2, 146.2, 143.3, 137.4, 134.6, 130.3, 128.2, 124.3, 121.3, 118.2, 117.3, 110.7, 78.5, 49.2, 38.6, 38.2, 19.8, 18.0, 10.7, 6.9, 5.6; IR [ATR]: ν = 2955, 2911, 2875, 1762, 1644, 1587, 1482, 1438, 1416, 1379, 1345, 1230, 1171, 1130, 1114, 1063, 1003, 966, 923, 884, 841, 801 cm^–1^; HRMS (ESI): exact mass calculated for C_28_H_43_O_5_Si [(M + H)^+^], 487.2874; found, 487.2875; [*α*]_D_^20^ = +70.7 (c 0.83, CH_2_Cl_2_).

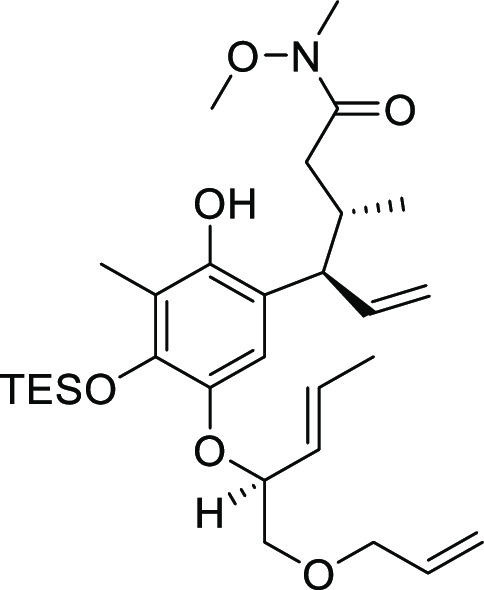



### (3*S*,4*R*)-4-(5-(((*S*,*E*)-1-(Allyloxy)pent-3-en-2-yl)oxy)-2-hydroxy-3-methyl-4-((triethylsilyl)oxy)phenyl)-*N*-methoxy-*N*,3-dimethylhex-5-enamide (**S8**)

A 25 mL Schlenk flask was charged with *N*,*O*-dimethyl hydroxylamine hydrochloride (411 mg, 4.21 mmol, 5 equiv) dissolved in 5 mL of dry toluene, and it was cooled to −20 °C. To the clear colorless solution was added AlMe_3_ (2.0 M, 2.11 mL, 4.21 mmol, 5 equiv) dropwise, and the mixture was stirred for 50 min. Then, compound **31** (410 mg, 0.84 mmol, 1 equiv) dissolved in 7 mL of dry toluene was added dropwise at −20 °C. After 45 min, TLC (petroleum ether/ethyl acetate, 4:1) at −15 °C confirmed full conversion. The reaction was quenched with saturated NH_4_Cl and saturated Seignette’s salt solution. It was diluted with ethyl acetate and stirred at room temperature for 2.5 h (until the aqueous layer was clear). Layers were separated, and the aqueous layer was extracted five times with ethyl acetate. The combined organic layer was washed with H_2_O, dried over MgSO_4_, and concentrated in vacuo. The desired product was obtained as a yellow amorphous solid in 93% yield (443 mg, 0.809 mmol) over two steps. ^1^H-NMR (400 MHz, CDCl_3_): δ = 8.08 (s, 1H), 6.49 (s, 1H), 6.08 (dt, *J* = 16.8, 9.9 Hz, 1H), 5.98–5.84 (m, 1H), 5.78–5.65 (m, 1H), 5.47 (ddd, *J* = 15.4, 7.2, 1.7 Hz, 1H), 5.32–5.22 (m, 1H), 5.21–5.13 (m, 2H), 5.08 (dd, *J* = 16.9, 2.1 Hz, 1H), 4.71–4.62 (m, 1H), 4.08–3.98 (m, 2H), 3.72–3.64 (m, 4H), 3.60–3.50 (m, 2H), 3.24 (s, 3H), 2.58 (dd, *J* = 17.5, 10.5 Hz, 1H), 2.30–2.10 (m, 5H), 1.69–1.61 (m, 3H), 0.95 (t, *J* = 7.8 Hz, 9H), 0.87 (d, *J* = 7.0 Hz, 3H), 0.75 (q, *J* = 8.6, 8.2 Hz, 6H); ^13^C{^1^H}-NMR (101 MHz, CDCl_3_): δ = 174.9, 147.0, 143.3, 141.4, 135.8, 134.9, 129.8, 129.1, 120.8, 118.2, 117.1, 111.9, 78.5, 72.9, 72.5, 61.3, 46.1, 35.6, 33.5, 32.3, 29.8, 18.0, 14.7, 10.4, 7.0, 5.6; HRMS (ESI): exact mass calculated for C_30_H_50_NO_6_Si [(M + H)^+^], 548.3402; found, 548.3408; [*α*]_D_^20^ = +22.2 (c 1.05, CH_2_Cl_2_).

### (3*S*,4*R*)-4-(5-(((*S*,*E*)-1-(Allyloxy)pent-3-en-2-yl)oxy)-2-((*tert*-butyldimethylsilyl)oxy)-3-methyl-4-((triethylsilyl)oxy)phenyl)-*N*-methoxy-*N*,3-dimethylhex-5-enamide (**39**)

A 50 mL Schlenk flask was charged with compound **S8** (446 mg, 0.814 mmol, 1 equiv) in 6 mL of dry DCM. It was cooled to −92 °C, and 2,6-lutidine (0.38 mL, 3.26 mmol, 4 equiv) was added followed by TBSOTf (0.26 mL, 1.22 mmol, 1.5 equiv). The reaction was allowed to warm to room temperature upon which TLC (petroleum ether/ethyl acetate, 4:1) confirmed full conversion. The reaction was quenched by addition of saturated NaHCO_3_ solution, and the aqueous layer was extracted three times with DCM. The combined organic layer was dried over MgSO_4_ and concentrated in vacuo. The crude mixture was subjected to column chromatography, and the desired product was obtained in 83% yield (445 mg, 0.672 mmol) accompanied by compound **31** in 11% yield (43 mg, 0.088 mmol). ^1^H-NMR (400 MHz, CDCl_3_): δ = 6.54 (s, 1H), 6.04–5.83 (m, 2H), 5.80–5.66 (m, 1H), 5.44 (ddq, *J* = 15.4, 7.3, 1.5 Hz, 1H), 5.32–5.21 (m, 1H), 5.21–5.02 (m, 3H), 4.74–4.66 (m, 1H), 4.07–3.96 (m, 2H), 3.67 (dd, *J* = 10.0, 6.4 Hz, 1H), 3.54 (dd, *J* = 10.0, 4.9 Hz, 1H), 3.50–3.46 (m, 4H), 3.08 (s, 3H), 2.23–2.03 (m, 6H), 1.65 (dd, *J* = 6.5, 1.6 Hz, 3H), 1.03 (s, 9H), 1.00–0.90 (m, 12H), 0.74 (q, *J* = 7.3 Hz, 6H), 0.13 (s, 6H); ^13^C{^1^H}-NMR (101 MHz, CDCl_3_): δ = 174.2 (HMBC), 145.5, 143.5, 142.8, 140.0, 134.9, 130.3, 128.4, 125.3, 121.1, 117.2, 115.7, 111.8, 78.0, 72.9, 72.4, 61.0, 47.2, 26.4, 18.8, 18.1, 18.0, 12.8, 7.0, 5.6, −2.8, −3.0; IR [ATR]: ν = 2955, 2933, 2875, 1669, 1591, 1482, 1415, 1381, 1333, 1302, 1239, 1128, 1077, 1004, 936, 903, 838, 822, 803, 778, 739, 692 cm^–1^; HRMS (ESI): exact mass calculated for C_30_H_50_NO_6_Si [(M–C_6_H_15_Si + H)^+^], 548.3402; found, 548.3400; [*α*]_D_^20^ = +33.4 (c 1.0, CH_2_Cl_2_).

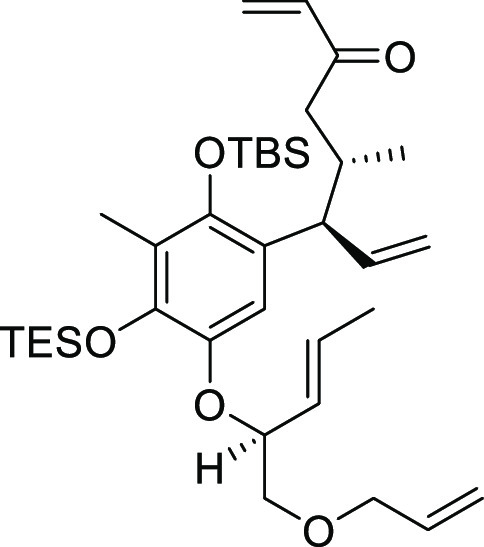



### (5*S*,6*R*)-6-(5-(((S,E)-1-(Allyloxy)pent-3-en-2-yl)oxy)-2-((*tert*-butyldimethylsilyl)oxy)-3-methyl-4-((triethylsilyl)oxy)phenyl)-5-methylocta-1,7-dien-3-one (**S9**)

A 25 mL Schlenk flask was charged with compound **39** (251 mg, 0.379 mmol, 1 equiv) dissolved in 5 mL of dry THF. The colorless mixture was cooled to 0 °C, and vinyl magnesium bromide (1.0 M, 0.80 mL, 0.80 mmol, 2.1 equiv) was added dropwise. After 1 h, TLC (petroleum ether/ethyl acetate, 5:1) confirmed full conversion. The reaction was quenched by addition of saturated NH_4_Cl solution, and the aqueous layer was extracted three times with ethyl acetate. The combined organic layer was washed with H_2_O and brine and dried over MgSO_4_. The crude material was flashed over a short plug of silica, and the desired product was collected as a pale-yellow oil in 95% yield (227 mg, 0.36 mmol). ^1^H-NMR (400 MHz, CDCl_3_): δ = 6.50 (s, 1H), 6.16 (dd, *J* = 17.7, 10.7 Hz, 1H), 6.04–5.84 (m, 3H), 5.77–5.60 (m, 2H), 5.50–5.39 (m, 1H), 5.27 (dq, *J* = 17.2, 1.7 Hz, 1H), 5.20–5.02 (m, 3H), 4.75–4.65 (m, 1H), 4.07–3.95 (m, 2H), 3.67 (dd, *J* = 9.9, 6.6 Hz, 1H), 3.59–3.45 (m, 2H), 2.39–2.28 (m, 1H), 2.24–2.01 (m, 5H), 1.71–1.57 (m, 3H), 1.03 (s, 9H), 0.94 (t, *J* = 7.9 Hz, 9H), 0.88 (d, *J* = 6.0 Hz, 3H), 0.75 (q, *J* = 7.5 Hz, 6H), 0.13 (s, 6H); ^13^C{^1^H}-NMR (101 MHz, CDCl_3_): δ = 201.0, 145.5, 143.6, 142.8, 139.7, 136.9, 134.8, 130.1, 128.4, 127.7, 125.1, 121.3, 117.2, 116.0, 111.4, 77.9, 73.0, 72.4, 47.1, 44.6, 35.0, 26.4, 18.8, 18.0, 18.0, 12.8, 7.0, 5.6, −2.8, −3.0.

### (3*S*,5*S*,6*R*)-6-(5-(((*S*,*E*)-1-(Allyloxy)pent-3-en-2-yl)oxy)-2-((*tert*-butyldimethylsilyl)oxy)-3-methyl-4-((triethylsilyl)oxy)phenyl)-5-methylocta-1,7-dien-3-ol (**40**)

A 25 mL Schlenk flask was charged with compound **S9** (162 mg, 0.258 mmol, 1 equiv) dissolved in 4 mL of dry DCM. The colorless solution was cooled to −90 °C and stirred for 5 min. Then, DIBAL (1.0 M in hexanes, 0.31 mL, 0.31 mmol, 1.2 equiv) was added dropwise. After 15 min, TLC (petroleum ether/ethyl acetate, 6:1) confirmed full conversion. The reaction was quenched by addition of saturated NH_4_Cl solution and saturated Seignette’s salt solution. The mixture was stirred for 1 h and 20 min before layers were separated. The aqueous layer was extracted three times with DCM, and the combined organic layer was dried over MgSO_4_. It was concentrated in vacuo, and the crude material was subjected to column chromatography. Pure **40** (eluting first) was obtained in 74% yield (120 mg, 0.19 mmol) accompanied by 7% 12 mg, 0.019 mmol) **40′** (eluting second). Allyl alcohol **40**: ^1^H-NMR (400 MHz, CDCl_3_): δ = 6.50 (s, 1zfH), 6.05–5.84 (m, 2H), 5.83–5.66 (m, 2H), 5.48 (ddq, *J* = 15.5, 6.9, 1.6 Hz, 1H), 5.27 (dq, *J* = 17.3, 1.6 Hz, 1H), 5.21–4.96 (m, 5H), 4.75–4.66 (m, 1H), 4.09–3.99 (m, 3H), 3.68 (dd, *J* = 10.0, 6.5 Hz, 1H), 3.59–3.49 (m, 2H), 2.08 (s, 4H), 1.96–1.84 (m, 1H), 1.66 (dd, *J* = 6.4, 1.6 Hz, 3H), 1.34 (ddd, *J* = 13.3, 10.0, 2.9 Hz, 1H), 1.14 (ddd, *J* = 14.1, 10.6, 3.8 Hz, 1H), 1.02 (s, 9H), 0.95 (t, *J* = 7.9 Hz, 9H), 0.88 (d, *J* = 6.7 Hz, 3H), 0.75 (q, *J* = 7.7 Hz, 6H), 0.12 (d, *J* = 4.2 Hz, 6H); ^13^C{^1^H}-NMR (101 MHz, CDCl_3_): δ = 145.6, 143.3, 142.7, 142.2, 139.9, 134.9, 129.9, 128.6, 125.6, 121.3, 117.2, 115.8, 113.6, 112.0, 77.9, 73.0, 72.4, 70.9, 47.4, 42.2, 34.0, 26.5, 18.9, 18.0, 16.7, 12.8, 7.0, 5.6, −2.8, −2.9; [*α*]_D_^20^ = +35.2 (c 1.0, CH_2_Cl_2_).

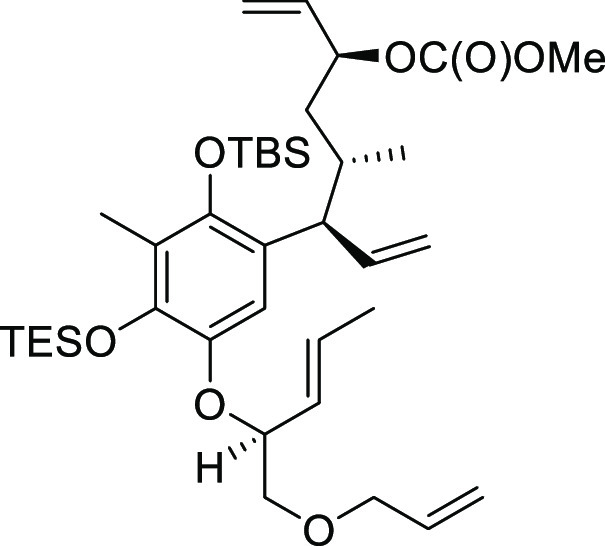



### (3*S*,5*S*,6*R*)-6-(5-(((*S*,*E*)-1-(Allyloxy)pent-3-en-2-yl)oxy)-2-((*tert*-butyldimethylsilyl)oxy)-3-methyl-4-((triethylsilyl)oxy)phenyl)-5-methylocta-1,7-dien-3-yl methyl carbonate (**S10**)

A 25 mL round-bottom flask was charged with compound **40** (59 mg, 93 μmol, 1 equiv) in 2 mL of dry DCM. The clear colorless solution was cooled to −20 °C, and pyridine (26 μL, 0.327 mmol, 3.5 equiv) was added followed by methyl chloroformate (25 μL, 0.327 mmol, 3.5 equiv). The solution turned orange, and the formation of a precipitate was observed. After 2 h, TLC (petroleum ether/ethyl acetate, 10:1) at −5 °C showed full conversion. The reaction was quenched by addition of brine, and the aqueous layer was extracted twice with DCM. The combined organic layer was dried over MgSO_4_, filtered, and concentrated in vacuo. The crude mixture was flashed over a short plug of silica, and the desired product was obtained as a colorless oil in 99% yield (64 mg, 93 μmol). ^1^H-NMR (400 MHz, CDCl_3_): δ = 6.49 (s, 1H), 6.03–5.84 (m, 2H), 5.71 (dddd, *J* = 16.9, 12.3, 8.7, 6.2 Hz, 2H), 5.47 (ddq, *J* = 15.4, 7.1, 1.6 Hz, 1H), 5.27 (dq, *J* = 17.3, 1.7 Hz, 1H), 5.23–5.00 (m, 6H), 4.76–4.67 (m, 1H), 4.08–3.99 (m, 2H), 3.74 (s, 3H), 3.68 (dd, *J* = 10.0, 6.5 Hz, 1H), 3.59–3.49 (m, 2H), 2.07 (s, 3H), 1.88–1.74 (m, 1H), 1.70–1.56 (m, 4H), 1.16 (ddd, *J* = 14.2, 10.9, 3.0 Hz, 1H), 1.01 (s, 9H), 0.95 (t, *J* = 8.3 Hz, 9H), 0.86 (d, *J* = 6.5 Hz, 3H), 0.75 (q, *J* = 7.7 Hz, 6H), 0.13 (s, 3H), 0.11 (s, 3H); ^13^C{^1^H}-NMR (101 MHz, CDCl_3_): δ = 155.6, 145.6, 143.2, 142.8, 139.6, 137.0, 134.9, 129.9, 128.5, 125.1, 121.2, 117.2, 116.5, 116.0, 112.2, 78.0, 77.1, 73.0, 72.4, 54.6, 47.1, 39.0, 33.5, 26.5, 18.8, 18.0, 16.3, 12.8, 7.0, 5.6, −2.9; HRMS (ESI): exact mass calculated for C_36_H_62_O_5_Si_2_Na [(M–C_2_H_2_O_2_ + Na)^+^], 653.4028; found, 653.4031; [*α*]_D_^20^ = +35.7 (c 1.1, CH_2_Cl_2_).

### (3*S*,5*S*,6*R*)-6-(5-(((*S*,*E*)-1-(Allyloxy)pent-3-en-2-yl)oxy)-2-((*tert*-butyldimethylsilyl)oxy)-4-hydroxy-3-methylphenyl)-5-methylocta-1,7-dien-3-yl methyl carbonate (**30**)

A 25 mL round-bottom flask was charged with compound **S10** (64 mg, 93 μmol, 1 equiv) in 4 mL of THF and 0.3 mL of H_2_O. TFA (10% in H_2_O, 0.7 mL, 0.93 mmol, 10 equiv) was added at room temperature, and the mixture was stirred for 1.5 h. After TLC (petroleum ether/ethyl acetate, 10:1) showed full conversion, the reaction was quenched by addition of saturated NaHCO_3_ solution. The aqueous layer was extracted three times with ethyl acetate, and the combined organic layer was washed with H_2_O and brine. It was dried over MgSO_4_, filtered, and concentrated in vacuo. The crude material was flashed over a plug of silica, and the desired product was obtained as a colorless oil in virtually quantitative yield (53 mg, 92 μmol). ^1^H-NMR (400 MHz, CDCl_3_): δ = 6.55 (s, 1H), 6.03–5.84 (m, 2H), 5.77–5.64 (m, 2H), 5.51 (ddq, *J* = 15.3, 7.7, 1.6 Hz, 1H), 5.37–4.97 (m, 7H), 4.25–4.16 (m, 1H), 4.16–4.11 (m, 2H), 3.74 (s, 3H), 3.63 (dd, *J* = 10.1, 8.9 Hz, 1H), 3.58–3.49 (m, *J* = 4.7 Hz, 2H), 2.10 (s, 3H), 1.87–1.77 (m, 1H), 1.75–1.61 (m, 4H), 1.22–1.14 (m, 1H), 1.03 (s, 9H), 0.89 (d, *J* = 6.6 Hz, 3H), 0.16 (s, 3H), 0.15 (s, 3H); ^13^C{^1^H}-NMR (101 MHz, CDCl_3_): δ = 155.6, 148.2, 146.1, 140.2, 139.5, 137.0, 134.1, 131.3, 127.0, 123.2, 118.2, 117.9, 116.6, 116.1, 115.5, 84.1, 77.2, 72.7, 72.7, 54.6, 46.9, 38.9, 33.5, 26.4, 18.8, 18.0, 16.7, 11.6, −2.6, −2.8; HRMS (ESI): exact mass calculated for C_32_H_51_O_7_Si [(M + H)^+^], 575.3399; found, 575.3369.

### (1*R*,2*S*,4*S*,5*R*)-9-(((*S*,*E*)-1-(Allyloxy)pent-3-en-2-yl)oxy)-6-((*tert*-butyldimethylsilyl)oxy)-2,7-dimethyl-1,4-divinylspiro[4.5]deca-6,9-dien-8-one (**29a**)

A 10 mL Schlenk flask was charged with PPh_3_ (11.4 mg, 44 μmol, 50 mol %) and Pd(dba)_2_ (10.0 mg, 17 μmol, 20 mol %), and compound **30** (50 mg, 87 μmol, 1 equiv) dissolved in 2 mL of dry DCM was added. The yellow reaction mixture was stirred for 30 min, at which point TLC (petroleum ether/ethyl acetate, 10:1) confirmed full conversion. The solvent was removed in vacuo, and the residue was purified by column chromatography. The desired product was obtained as a colorless oil in 21% yield (9 mg, 18 μmol). ^1^H-NMR (600 MHz, CDCl_3_): δ = 5.90 (ddt, *J* = 17.3, 10.4, 5.7 Hz, 1H), 5.75 (dqd, *J* = 15.6, 6.5, 1.0 Hz, 1H), 5.60 (ddd, *J* = 17.0, 10.3, 8.7 Hz, 1H), 5.52 (ddd, *J* = 17.0, 10.1, 8.4 Hz, 1H), 5.44 (ddq, *J* = 15.6, 7.0, 1.6 Hz, 1H), 5.27 (dq, *J* = 17.2, 1.6 Hz, 1H), 5.19 (dq, *J* = 10.4, 1.4 Hz, 1H), 5.14 (s, 1H), 4.93 (ddd, *J* = 10.3, 2.0, 0.7 Hz, 1H), 4.89 (ddd, *J* = 17.0, 2.0, 1.0 Hz, 1H), 4.81 (ddd, *J* = 16.9, 2.1, 1.0 Hz, 1H), 4.77 (ddd, *J* = 10.1, 2.1, 0.7 Hz, 1H), 4.59–4.54 (m, 1H), 4.04–3.97 (m, 2H), 3.64 (dd, *J* = 10.1, 6.4 Hz, 1H), 3.51 (dd, *J* = 10.1, 4.7 Hz, 1H), 2.82–2.76 (m, 1H), 2.53 (ddd, *J* = 11.5, 8.4, 6.0 Hz, 1H), 1.94 (ddt, *J* = 12.2, 10.6, 6.1 Hz, 1H), 1.84 (dt, *J* = 11.9, 6.0 Hz, 1H), 1.75–1.73 (m, 7H), 1.06 (d, *J* = 6.4 Hz, 3H), 0.96 (s, 9H), 0.21 (s, 3H), 0.19 (s, 3H); ^13^C{^1^H}-NMR (151 MHz, CDCl_3_): δ = 203.5, 159.2, 144.3, 138.5, 138.2, 134.6, 130.3, 127.8, 118.4, 117.5, 115.7, 114.7, 113.1, 72.5, 72.3, 63.3, 62.0, 58.0, 39.8, 39.2, 25.9, 18.9, 18.1, 17.8, 9.5, −3.6, −3.7; HRMS (ESI): exact mass calculated for C_30_H_47_O_4_Si [(M + H)^+^], 499.3238; found, 499.3207.

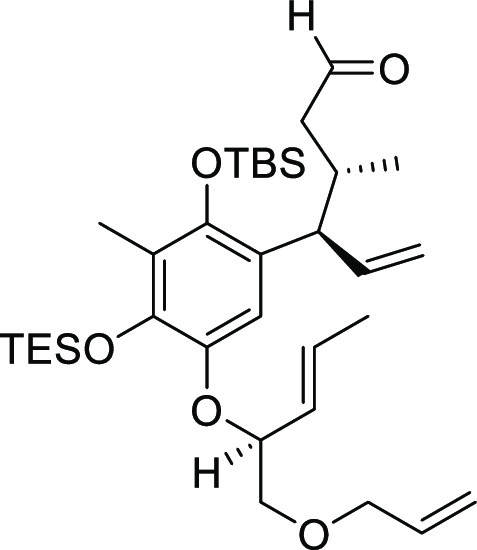



### (3*S*,4*R*)-4-(5-(((*S*,*E*)-1-(Allyloxy)pent-3-en-2-yl)oxy)-2-((*tert*-butyldimethylsilyl)oxy)-3-methyl-4-((triethylsilyl)oxy)phenyl)-3-methylhex-5-enal (**S11**)

A 25 mL Schlenk flask was charged with compound **39** (310 mg, 0.468 mmol, 1 equiv) in 5 mL of dry toluene. The pale-yellow solution was cooled to −91 °C, and DIBAL (1.0 M, 0.56 mL, 0.56 mmol, 1.2 equiv) was added dropwise. After 2 min, TLC (petroleum ether/ethyl acetate, 4:1) showed full conversion. The reaction was quenched with saturated Seignette’s salt solution, and the flask was removed from the cooling bath. The mix was allowed to warm to room temperature and stirred for another 1.5 h. The aqueous layer was extracted four times with Et_2_O, and the combined organic layer was washed with H_2_O and brine. It was dried over MgSO_4_, filtered, and concentrated in vacuo. The crude material was flashed over a short plug of silica, and the desired product was obtained as a very pale-yellow oil in 98% yield (278 mg, 0.461 mmol). ^1^H-NMR (400 MHz, CDCl_3_): δ = 9.47 (dd, *J* = 3.0, 1.2 Hz, 1H), 6.48 (s, 1H), 6.03–5.85 (m, 2H), 5.78–5.65 (m, 1H), 5.44 (ddq, *J* = 15.5, 7.0, 1.5 Hz, 1H), 5.32–5.22 (m, 1H), 5.22–5.11 (m, 2H), 5.11–5.03 (m, 1H), 4.71 (q, *J* = 6.3 Hz, 1H), 4.09–3.97 (m, 2H), 3.68 (dd, *J* = 10.0, 6.6 Hz, 1H), 3.59–3.46 (m, 2H), 2.25–1.97 (m, 6H), 1.70–1.61 (m, 3H), 1.02 (s, 9H), 0.99–0.89 (m, 12H), 0.80–0.69 (m, 6H), 0.12 (s, 6H); ^13^C{^1^H}-NMR (101 MHz, CDCl_3_): δ = 202.9, 145.5, 143.5, 143.0, 139.2, 134.8, 130.1, 128.5, 124.7, 121.5, 117.2, 116.4, 111.5, 77.9, 73.0, 72.5, 49.0, 46.7, 33.5, 26.4, 18.8, 18.2, 18.0, 12.8, 7.0, 5.6, −2.9, −3.0; IR [ATR]: ν = 2954, 2931.04, 2875, 1726, 1590, 1482, 1417, 1378, 1360, 1301, 1239, 1128, 1078, 1004, 964, 937, 894, 838, 822, 802, 778, 739, 691 cm^–1^; HRMS (ESI): exact mass calculated for C_34_H_58_O_5_Si_2_Na [(M + Na)^+^], 625.3715; found, 625.3719; [*α*]_D_^20^ = +53.4 (c 1.0, CH_2_Cl_2_).

### (3*R*,5*S*,6*R*)-6-(5-(((*S*,*E*)-1-(Allyloxy)pent-3-en-2-yl)oxy)-2-((*tert*-butyldimethylsilyl)oxy)-3-methyl-4-((triethylsilyl)oxy)phenyl)-5-methylocta-1,7-dien-3-ol (**40′**)

A 25 mL Schlenk flask was charged with compound **S11** (258 mg, 0.427 mmol, 1 equiv) in 5 mL of dry THF. The colorless solution was cooled by an ice bath, and vinyl magnesium bromide (1.0 M, 0.51 mL, 0.51 mmol, 1.2 equiv) was added dropwise over 5 min. TLC (petroleum ether/ethyl acetate, 10:1) after 20 min showed full conversion. The reaction was quenched by addition of saturated NH_4_Cl solution, and the aqueous layer was extracted four times with Et_2_O. The combined organic layer was dried over MgSO_4_, filtered, and concentrated in vacuo. The crude residue was purified by column chromatography. Allyl alcohol **40** (eluted first) was obtained in 24% yield (65 mg, 0.103 mmol), and allyl alcohol **40′** (eluted second) was obtained in 46% yield (124 mg, 0.197 mmol). Allyl alcohol **40′**: ^1^H-NMR (400 MHz, CDCl_3_): δ = 6.49 (s, 1H), 6.04–5.84 (m, 2H), 5.80–5.57 (m, 2H), 5.54–5.43 (m, 1H), 5.27 (dq, *J* = 17.2, 1.7 Hz, 1H), 5.21–4.98 (m, 5H), 4.75–4.63 (m, 1H), 4.08–3.98 (m, 2H), 3.95–3.88 (m, 1H), 3.68 (dd, *J* = 10.0, 6.5 Hz, 1H), 3.58–3.49 (m, 2H), 2.07 (s, 3H), 1.71–1.56 (m, 4H), 1.41 (ddd, *J* = 13.3, 7.8, 3.9 Hz, 1H), 1.32–1.25 (m, 1H), 1.02 (s, 9H), 0.95 (t, *J* = 7.9 Hz, 9H), 0.87 (d, *J* = 6.7 Hz, 3H), 0.75 (q, *J* = 7.8 Hz, 6H), 0.13 (s, 3H), 0.11 (s, 3H); ^13^C{^1^H}-NMR (101 MHz, CDCl_3_): δ = 145.4, 143.3, 142.7, 141.3, 139.6, 134.8, 129.8, 128.8, 125.8, 121.3, 117.2, 116.0, 114.9, 111.9, 77.9, 73.0, 72.4, 72.4, 47.3, 42.2, 34.9, 26.5, 18.8, 18.0, 17.4, 12.9, 7.0, 5.6, −2.9, −2.9; HRMS (ESI): exact mass calculated for C_30_H_48_O_5_SiNa [(M–C_6_H_15_Si + Na)^+^], 539.3163; found, 539.3187; [*α*]_D_^20^ = +38.6 (c 1.0, CH_2_Cl_2_).

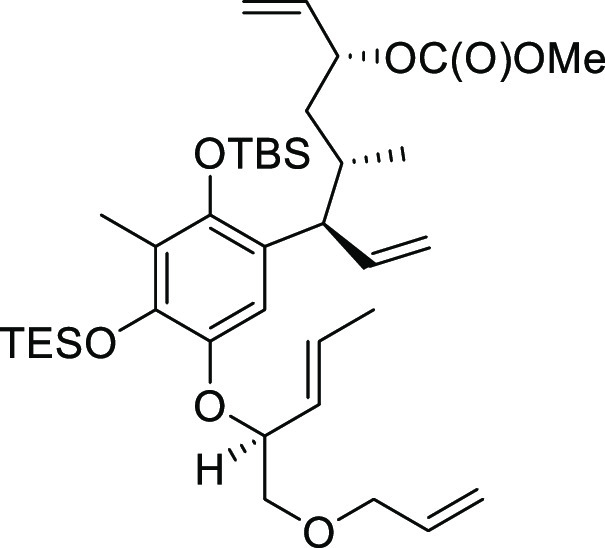



### (3*R*,5*S*,6*R*)-6-(5-(((*S*,*E*)-1-(Allyloxy)pent-3-en-2-yl)oxy)-2-((*tert*-butyldimethylsilyl)oxy)-3-methyl-4-((triethylsilyl)oxy)phenyl)-5-methylocta-1,7-dien-3-yl methyl carbonate (**S12**)

A 10 mL round-bottom flask was charged with compound **40′** (63 mg, 0.10 mmol, 1 equiv) in 1.5 mL of dry DCM. The clear colorless solution was cooled to −20 °C, and pyridine (28 μL, 0.35 mmol, 3.5 equiv) was added followed by methyl chloroformate (27 μL, 0.35 mmol, 3.5 equiv). The solution turned orange, and the formation of a precipitate was observed. After 2 h, TLC (petroleum ether/ethyl acetate, 10:1) at 10 °C showed full conversion. The reaction was quenched by addition of brine, and the aqueous layer was extracted three times with DCM. The combined organic layer was dried over MgSO_4_, filtered, and concentrated in vacuo. The crude mixture was flashed over a short plug of silica, and the desired product was obtained as a colorless oil in 94% yield (65 mg, 94 μmol). ^1^H-NMR (400 MHz, CDCl_3_): δ = 6.46 (s, 1H), 6.01–5.83 (m, 2H), 5.79–5.66 (m, 1H), 5.63–5.42 (m, 2H), 5.31–5.00 (m, 6H), 4.98–4.90 (m, 1H), 4.73–4.64 (m, 1H), 4.07–3.96 (m, 2H), 3.73 (s, 3H), 3.67 (dd, *J* = 10.0, 6.5 Hz, 1H), 3.59–3.48 (m, 2H), 2.07 (s, 3H), 1.66 (dd, *J* = 6.4, 1.6 Hz, 3H), 1.62–1.37 (m, 3H), 1.01 (s, 9H), 0.95 (t, *J* = 7.9 Hz, 9H), 0.85 (d, *J* = 6.5 Hz, 3H), 0.75 (q, *J* = 8.5, 8.0 Hz, 6H), 0.12 (s, 3H), 0.11 (s, 3H); ^13^C{^1^H}-NMR (101 MHz, CDCl_3_): δ = 155.2, 145.5, 143.3, 142.8, 139.1, 135.8, 134.8, 129.7, 128.8, 125.3, 121.3, 118.6, 117.2, 116.2, 111.8, 79.1, 78.0, 73.0, 72.5, 54.6, 47.5, 38.8, 34.4, 26.5, 18.8, 18.0, 16.4, 12.9, 7.0, 5.6, −2.8, −2.9; HRMS (ESI): exact mass calculated for C_38_H_65_O_7_Si_2_ [(M + H)^+^], 689.4263; found, 689.4244; [*α*]_D_^20^ = +29.2 (c 1.0, CH_2_Cl_2_).

### (3*R*,5*S*,6*R*)-6-(5-(((*S*,*E*)-1-(Allyloxy)pent-3-en-2-yl)oxy)-2-((*tert*-butyldimethylsilyl)oxy)-4-hydroxy-3-methylphenyl)-5-methylocta-1,7-dien-3-yl methyl carbonate (**30′**)

A 25 mL round-bottom flask was charged with compound **S12** (94 mg, 0.136 mmol, 1 equiv) in 6 mL of THF and 0.5 mL of H_2_O. TFA (10% in H_2_O, 1.0 mL, 1.36 mmol, 10 equiv) was added at room temperature, and the mixture was stirred for 2 h and 50 min. After TLC (petroleum ether/ethyl acetate, 10:1) showed full conversion, the reaction was quenched by addition of saturated NaHCO_3_ solution. The aqueous layer was extracted three times with ethyl acetate, and the combined organic layer was washed with H_2_O and brine. It was dried over MgSO_4_, filtered, and concentrated in vacuo. The crude material was flashed over a plug of silica, and the desired product was obtained as a colorless oil in 98% yield (77 mg, 0.134 mmol). ^1^H-NMR (400 MHz, CD_2_Cl_2_): δ = 7.26 (s, 1H), 6.54 (s, 1H), 6.04–5.84 (m, 2H), 5.80–5.67 (m, 1H), 5.66–5.47 (m, 2H), 5.39–5.29 (m, 3H), 5.29–5.12 (m, 1H), 5.07 (ddd, *J* = 10.4, 2.0, 1.0 Hz, 1H), 5.04–4.97 (m, 1H), 4.91–4.83 (m, 1H), 4.21–4.11 (m, 3H), 3.69 (s, 3H), 3.62 (dd, *J* = 10.2, 9.1 Hz, 1H), 3.58–3.45 (m, 2H), 2.08 (s, 3H), 1.73 (dd, *J* = 6.5, 1.6 Hz, 3H), 1.64–1.49 (m, 2H), 1.39 (ddd, *J* = 13.4, 10.6, 5.4 Hz, 1H), 1.04 (s, 9H), 0.90 (d, *J* = 6.6 Hz, 3H), 0.17 (s, 3H), 0.15 (s, 3H); ^13^C{^1^H}-NMR (101 MHz, CD_2_Cl_2_): δ = 155.3, 146.5, 140.4, 140.1, 136.3, 134.5, 131.4, 127.4, 123.7, 118.3, 118.0, 117.9, 116.3, 115.7, 84.6, 79.2, 73.1, 72.8, 54.8, 47.6, 39.0, 34.6, 26.5, 19.0, 18.0, 17.2, 11.7, −2.5, −2.8; HRMS (ESI): exact mass calculated for C_32_H_51_O_7_Si [(M + H)^+^], 575.3399; found, 575.3372.

### (3*R*,5*S*,6*R*)-6-(5-(((*S*,*E*)-1-(Allyloxy)pent-3-en-2-yl)oxy)-2-((*tert*-butyldimethylsilyl)oxy)-3-methyl-4-((triethylsilyl)oxy)phenyl)-5-methylocta-1,7-dien-3-yl 4-nitrobenzoate (**41**)

A 10 mL Schlenk flask was charged with compound **40** (50 mg, 79 μmol, 1 equiv) dissolved in 2 mL of dry toluene, and PPh_3_ (88 μL, 0.367 mmol, 4.5 equiv) was added. After 5 min, *p*-nitrobenzoic acid (112 mg, 0.555 mmol, 7 equiv) was added and the mixture was stirred for 5 min before DIAD (46 mg, 0.277 mmol, 3.5 equiv) dissolved in 0.9 mL of dry toluene was added. After 40 min, TLC (petroleum ether/ethyl acetate, 5:1) confirmed full conversion and the reaction was quenched by addition of saturated NaHCO_3_ solution. The aqueous layer was extracted twice with ethyl acetate, and the combined organic layer was washed with NaHCO_3_ followed by H_2_O and brine. It was dried over MgSO_4_ and concentrated in vacuo. The crude material was purified by column chromatography, and the desired product was obtained in 63% yield (39 mg, 50 μmol). ^1^H-NMR (400 MHz, CDCl_3_): δ = 8.32–8.22 (m, 2H), 8.20–8.12 (m, 2H), 6.49 (s, 1H), 6.06–5.84 (m, 2H), 5.80–5.60 (m, 2H), 5.49 (ddq, *J* = 15.5, 6.9, 1.6 Hz, 1H), 5.44–5.35 (m, 1H), 5.32–5.28 (m, 1H), 5.26–5.24 (m, 1H), 5.22–5.02 (m, 4H), 4.73–4.66 (m, 1H), 4.06–4.00 (m, 2H), 3.67 (ddd, *J* = 12.0, 10.0, 6.3 Hz, 1H), 3.59–3.52 (m, 2H), 2.08 (s, 3H), 1.70–1.60 (m, 4H), 1.55–1.39 (m, 1H), 1.01 (s, 9H), 0.98–0.89 (m, 12H), 0.76 (q, *J* = 8.0 Hz, 6H), 0.13 (s, 3H), 0.12 (s, 3H); ^13^C{^1^H}-NMR (101 MHz, CDCl_3_): δ = 163.9, 150.6, 145.5, 143.4, 142.8, 139.1, 136.3, 135.7, 134.8, 130.8, 129.6, 128.8, 125.3, 123.6, 121.3, 118.6, 117.2, 116.3, 111.8, 78.0, 76.7, 73.0, 72.5, 47.6, 38.8, 34.7, 26.5, 18.8, 18.0, 16.6, 12.9, 7.0, 5.6, −2.8, −2.8; [*α*]_D_^20^ = +13.2 (c 0.95, CH_2_Cl_2_).

### 6-(((*S*,*E*)-1-(Allyloxy)pent-3-en-2-yl)oxy)-3-((*tert*-butyldimethylsilyl)oxy)-4-((3*R*,4*S*,6*R*)-6-hydroxy-4-methylocta-1,7-dien-3-yl)-2-methylphenol (**42**)

A 25 mL round-bottom flask was charged with compound **41** (20 mg, 26 μmol, 1 equiv) in 3.5 mL of EtOH/iPrOH (2.5:1), and K_2_CO_3_ (35 mg, 0.256 mmol, 10 equiv) was added. The colorless mixture was stirred for 2 days at which point TLC (petroleum ether/ethyl acetate, 5:1) confirmed full conversion. The reaction was quenched by addition of solid NH_4_Cl, and solvents were evaporated. The residue was taken up in ethyl acetate and little H_2_O. The aqueous layer was extracted three times with ethyl acetate, and the combined organic layer was washed with H_2_O and Brine. It was dried over MgSO_4_ and concentrated in vacuo. The crude material was subjected to column chromatography and desired product was collected in 76% yield (10 mg, 19 μmol). ^1^H-NMR (600 MHz, CDCl_3_): δ = 7.33 (s, 1H), 6.55 (s, 1H), 6.00–5.87 (m, 2H), 5.74–5.62 (m, 2H), 5.51 (ddq, *J* = 15.4, 7.8, 1.6 Hz, 1H), 5.34 (dq, *J* = 17.3, 1.6 Hz, 1H), 5.25 (dq, *J* = 10.3, 1.3 Hz, 1H), 5.11 (dt, *J* = 17.1, 1.3 Hz, 1H), 5.07–5.03 (m, 2H), 5.00 (dt, *J* = 17.3, 1.5 Hz, 1H), 4.20–4.09 (m, 3H), 3.97–3.90 (m, 1H), 3.62 (dd, *J* = 10.1, 9.2 Hz, 1H), 3.55–3.49 (m, 2H), 2.10 (s, 3H), 1.73 (dd, *J* = 6.5, 1.7 Hz, 3H), 1.65–1.59 (m, 1H), 1.44 (ddd, *J* = 13.4, 8.3, 3.6 Hz, 1H), 1.33–1.27 (m, 1H), 1.03 (s, 9H), 0.90 (d, *J* = 6.6 Hz, 3H), 0.16 (s, 3H), 0.15 (s, 3H); ^13^C{^1^H}-NMR (151 MHz, CDCl_3_): δ = 148.1, 146.1, 141.2, 140.2, 139.6, 134.0, 131.3, 127.0, 123.8, 118.3, 118.0, 116.2, 115.5, 115.3, 84.3, 72.7, 72.7, 47.1, 41.9, 34.7, 26.5, 18.9, 18.0, 17.6, 11.6, −2.6, −2.8; [α]_D_^20^ = +61.6 (c 0.5, CH_2_Cl_2_).

### (1*R*,2*S*,4*S*,5*R*)-9-(((*S*,*E*)-1-(Allyloxy)pent-3-en-2-yl)oxy)-8-hydroxy-2,7-dimethyl-1,4-divinylspiro[4.5]deca-7,9-dien-6-one (**43**)

A 10 mL Schlenk was charged with compound **42** (10 mg, 19 μmol, 1 equiv) in 0.5 mL of dry toluene. The colorless solution was cooled in an ice bath, PBu_3_ (22 μL, 87 μmol, 4.5 equiv) was added, and the mixture was stirred for 15 min. Then, DIAD (27 mg, 0.135 mmol, 7 equiv) dissolved in 0.3 mL of dry toluene was added dropwise and the mixture was allowed to slowly warm up. After 2 h, TLC (petroleum ether/ethyl acetate, 5:1) confirmed full conversion. Volatiles were removed in vacuo, and the crude residue was subjected to column chromatography. The desired product was collected in 94% yield (7 mg, 18 μmol). ^1^H-NMR (600 MHz, CDCl_3_): δ = 8.18 (s, 1H), 5.92 (ddt, *J* = 17.3, 10.4, 5.8 Hz, 1H), 5.80–5.74 (m, 1H), 5.58 (s, 1H), 5.58–5.51 (m, 2H), 5.43 (ddq, *J* = 15.3, 8.1, 1.7 Hz, 1H), 5.32 (dq, *J* = 17.2, 1.5 Hz, 1H), 5.26 (dq, *J* = 10.4, 1.3 Hz, 1H), 4.93–4.91 (m, 1H), 4.90 (ddd, *J* = 11.7, 1.9, 0.8 Hz, 1H), 4.84 (ddd, *J* = 17.0, 2.0, 1.1 Hz, 1H), 4.79 (ddd, *J* = 10.1, 2.0, 0.8 Hz, 1H), 4.17–4.13 (m, 1H), 4.11 (ddt, *J* = 5.7, 4.2, 1.4 Hz, 2H), 3.61 (dd, *J* = 10.2, 9.1 Hz, 1H), 3.52 (dd, *J* = 10.2, 3.0 Hz, 1H), 2.83 (dd, *J* = 10.3, 9.0 Hz, 1H), 2.61–2.55 (m, 1H), 1.95–1.88 (m, 1H), 1.87–1.78 (m, 2H), 1.77 (d, *J* = 1.6 Hz, 3H), 1.76 (s, 3H), 1.05 (d, *J* = 6.4 Hz, 3H); ^13^C{^1^H}-NMR (151 MHz, CDCl_3_): δ = 201.8, 159.6, 142.4, 138.3, 137.8, 133.7, 132.8, 125.9, 124.3, 118.6, 115.8, 115.1, 111.4, 83.1, 72.7, 72.2, 63.6, 62.2, 56.7, 40.1, 39.3, 18.0, 17.6, 7.7; HRMS (ESI): exact mass calculated for C_24_H_33_O_4_ [(M + H)^+^], 385.2373; found, 385.2379.

## Notes

Supporting crystallographic data for this paper are provided free of charge by the joint Cambridge Crystallographic Data Centre and Fachinformationszentrum Karlsruhe Access Structures service at https://www.ccdc.cam.ac.uk/structures/?.

## Supporting Information Available

General information, NMR spectra (^1^H and ^13^C) of all prepared compounds, and discussion with respect to compounds **29** and **29a** (PDF)
