# Supplementary material for: Efforts toward the Total Synthesis of Elisabethin A
Source: J Org Chem. 2022 Oct 25;87(22):15333–49. doi: 10.1021/acs.joc.2c01914 (PMC9680034; doi:10.1021/acs.joc.2c01914)

Supporting Information

Efforts towards the total synthesis of elisabethin A

Maximilian Kaiser, David Schönbauer, Katharina Schragl, Matthias Weil[a], Peter Gaertner, Valentin S. Enev

Institute of Applied Synthetic Chemistry, TU Wien, Getreidemarkt 9/163, 1060 Wien, Austria

[a]Institute of Chemical Technologies and Analytics, TU Wien, Getreidemarkt 9/164, 1060 Wien, Austria

Table of Contents

General Information SI-2

Compound **29** vs **29a** SI-3

NMR spectra SI-4

1. 3-Methoxy-4-((triisopropylsilyl)oxy)benzaldehyde (**S1**) SI-4
2. 3-Methoxy-4-((triisopropylsilyl)oxy)phenyl formate (**S2**) SI-5
3. 3-Methoxy-4-((triisopropylsilyl)oxy)phenol (**S3**) SI-6
4. Triisopropyl(2-methoxy-4-(methoxymethoxy)phenoxy)silane (**S4**) SI-7
5. Triisopropyl(2-methoxy-4-(methoxymethoxy)-3-methylphenoxy)silane (**13**) SI-8
6. 3-Methoxy-2-methyl-4-((triisopropylsilyl)oxy)phenol (**14**) SI-9
7. (*E*)-Triisopropyl(2-methoxy-3-methyl-4-(penta-2,4-dien-1-yloxy)phenoxy)silane (**15**) SI-10
8. (*E*)-2-methoxy-3-methyl-4-(penta-2,4-dien-1-yloxy)phenol (**S5**) SI-11
9. (*E*)-Triethyl(2-methoxy-3-methyl-4-(penta-2,4-dien-1-yloxy)phenoxy)silane (**16**) SI-12
10. 3-Methoxy-2-methyl-6-(penta-1,4-dien-3-yl)-4-((triethylsilyl)oxy)phenol (**17**) SI-13
11. Triethyl(2-methoxy-3-methyl-5-(penta-1,4-dien-3-yl)-4-((triisopropylsilyl)oxy)phenoxy)silane (**18**) SI-14
12. 2-Methoxy-3-methyl-5-(penta-1,4-dien-3-yl)-4-((triisopropylsilyl)oxy)phenol (**11**) SI-15
13. (*S,E*)-(4-((1-(Allyloxy)pent-3-en-2-yl)oxy)-3-methoxy-2-methyl-6-(penta-1,4-dien-3-yl)phenoxy)triisopropylsilane (**19**) SI-16
14. (*S,E*)-2-(5-(Allyloxy)pent-3-en-2-yl)-6-methoxy-5-methyl-3-(penta-1,4-dien-3-yl)-4-((triisopropylsilyl)oxy)phenol (**10**) SI-17
15. (5*S*,8*S*)-2-Methoxy-3,8-dimethyl-4-((triisopropylsilyl)oxy)-5-vinyl-5,8-dihydronaphthalen-1-ol (**20**) SI-18
16. (((5*S*,8*S*)-2-Methoxy-3,8-dimethyl-5-vinyl-5,8-dihydronaphthalene-1,4-diyl)bis(oxy))bis(triisopropylsilane) (**9**) SI-19
17. Methyl (*E*)-3-((1*R*,4*S*)-6-methoxy-4,7-dimethyl-5,8-bis((triisopropylsilyl)oxy)-1,4-dihydronaphthalen-1-yl)acrylate (**21**) SI-20
18. Methyl (*R*)-3-((1S,4S)-6-methoxy-4,7-dimethyl-5,8-bis((triisopropylsilyl)oxy)-1,4-dihydronaphthalen-1-yl)butanoate (**7-epi-8**) SI-21
19. 1-((1*S*,4*S*)-6-Methoxy-4,7-dimethyl-5,8-bis((triisopropylsilyl)oxy)-1,4-dihydronaphthalen-1-yl)ethan-1-one (**23**) SI-22
20. 1-((1*S*,4*S*)-6-Methoxy-4,7-dimethyl-5,8-bis((triisopropylsilyl)oxy)-1,2,3,4-tetrahydronaphthalen-1-yl)ethan-1-one (**24**) SI-23
21. Methyl (*Z*)-3-((1*R*,4*S*)-6-methoxy-4,7-dimethyl-5,8-bis((triisopropylsilyl)oxy)-1,4-dihydronaphthalen-1-yl)but-2-enoate (***Z*-22**) SI-24
22. Methyl (*E*)-3-((1*R*,4*S*)-6-methoxy-4,7-dimethyl-5,8-bis((triisopropylsilyl)oxy)-1,4-dihydronaphthalen-1-yl)but-2-enoate (***E*-22**) SI-25
23. (5*S*,8*S*)-3-Methoxy-2,5-dimethyl-4-((triisopropylsilyl)oxy)-8-vinyl-5,8-dihydronaphthalen-1-ol (**25**) SI-26
24. (5*S*,8*S*)-3-Methoxy-2,5-dimethyl-4-((triisopropylsilyl)oxy)-8-vinyl-5,8-dihydronaphthalen-1-yl acrylate (**26**) SI-27
25. (4a*R*,7*S*)-9-Methoxy-7,10-dimethyl-8-((triisopropylsilyl)oxy)-4a,7-dihydro-2H-naphtho[1,8-bc]oxepin-2-one (**27**) SI-28
26. 3-Methoxy-2-methyl-6-(penta-1,4-dien-3-yl)-4-((triisopropylsilyl)oxy)phenol (**34**) SI-29
27. 3-Methoxy-2-methyl-6-(penta-1,4-dien-3-yl)-4-((triisopropylsilyl)oxy)phenyl acrylate (**33**) SI-30
28. 8-Methoxy-9-methyl-7-((triisopropylsilyl)oxy)-5-vinylbenzo[b]oxepin-2(5H)-one (**rac-35**) SI-31
29. 8-Methoxy-4,9-dimethyl-7-((triisopropylsilyl)oxy)-5-vinyl-4,5-dihydrobenzo[b]oxepin-2(3H)-one (**rac-36**) . SI-32
30. 8-Hydroxy-4,9-dimethyl-7-((triisopropylsilyl)oxy)-5-vinyl-4,5-dihydrobenzo[b]oxepin-2(3H)-one (**rac-S6**) . SI-33
31. 8-(Methoxymethoxy)-4,9-dimethyl-7-((triisopropylsilyl)oxy)-5-vinyl-4,5-dihydrobenzo[b]oxepin-2(3H)-one (**rac-32**) SI-34
32. 7-Hydroxy-8-(methoxymethoxy)-4,9-dimethyl-5-vinyl-4,5-dihydrobenzo[b]oxepin-2(3H)-one (**rac-37**) SI-35
33. (4*S*,5*R*)-7-(((*S*,*E*)-1-(allyloxy)pent-3-en-2-yl)oxy)-8-(methoxymethoxy)-4,9-dimethyl-5-vinyl-4,5-dihydrobenzo[b]oxepin-2(3H)-one (**38**) SI-36

34. (4*R*,5*S*)-7-(((*S*,*E*)-1-(allyloxy)pent-3-en-2-yl)oxy)-8-(methoxymethoxy)-4,9-dimethyl-5-vinyl-4,5-dihydrobenzo[b]oxepin-2(3H)-one (**38'**) SI-37
35. (4*S*,5*R*)-7-(((*S*,*E*)-1-(Allyloxy)pent-3-en-2-yl)oxy)-8-hydroxy-4,9-dimethyl-5-vinyl-4,5-dihydrobenzo[b]oxepin-2(3H)-one (**S7**) SI-38
36. (4*S*,5*R*)-7-(((*S*,*E*)-1-(Allyloxy)pent-3-en-2-yl)oxy)-4,9-dimethyl-8-((triethylsilyl)oxy)-5-vinyl-4,5-dihydrobenzo[b]oxepin-2(3H)-one (**31**) SI-39
37. (3*S*,4*R*)-4-(5-(((*S*,*E*)-1-(Allyloxy)pent-3-en-2-yl)oxy)-2-hydroxy-3-methyl-4-((triethylsilyl)oxy)phenyl)-N-methoxy-N,3-dimethylhex-5-enamide (**S8**) SI-40
38. (3*S*,4*R*)-4-(5-(((*S*,*E*)-1-(Allyloxy)pent-3-en-2-yl)oxy)-2-((tert-butyldimethylsilyl)oxy)-3-methyl-4-((triethylsilyl)oxy)phenyl)-N-methoxy-N,3-dimethylhex-5-enamide (**39**) SI-41
39. (5*S*,6*R*)-6-(5-(((*S*,*E*)-1-(allyloxy)pent-3-en-2-yl)oxy)-2-((tert-butyldimethylsilyl)oxy)-3-methyl-4-((triethylsilyl)oxy)phenyl)-5-methylocta-1,7-dien-3-one (**S9**) SI-42
40. (3*S*,5*S*,6*R*)-6-(5-(((*S*,*E*)-1-(Allyloxy)pent-3-en-2-yl)oxy)-2-((tert-butyldimethylsilyl)oxy)-3-methyl-4-((triethylsilyl)oxy)phenyl)-5-methylocta-1,7-dien-3-ol (**40**) SI-43
41. (3*S*,5*S*,6*R*)-6-(5-(((*S*,*E*)-1-(Allyloxy)pent-3-en-2-yl)oxy)-2-((tert-butyldimethylsilyl)oxy)-3-methyl-4-((triethylsilyl)oxy)phenyl)-5-methylocta-1,7-dien-3-yl methyl carbonate (**S10**) SI-44
42. (3*S*,5*S*,6*R*)-6-(5-(((*S*,*E*)-1-(Allyloxy)pent-3-en-2-yl)oxy)-2-((tert-butyldimethylsilyl)oxy)-4-hydroxy-3-methylphenyl)-5-methylocta-1,7-dien-3-yl methyl carbonate (**30**) SI-45
43. (3*S*,4*R*)-4-(5-(((*S*,*E*)-1-(Allyloxy)pent-3-en-2-yl)oxy)-2-((tert-butyldimethylsilyl)oxy)-3-methyl-4-((triethylsilyl)oxy)phenyl)-3-methylhex-5-enal (**S11**) SI-46
44. (3*R*,5*S*,6*R*)-6-(5-(((*S*,*E*)-1-(allyloxy)pent-3-en-2-yl)oxy)-2-((tert-butyldimethylsilyl)oxy)-3-methyl-4-((triethylsilyl)oxy)phenyl)-5-methylocta-1,7-dien-3-ol (**40'**) SI-47
45. (3*R*,5*S*,6*R*)-6-(5-(((*S*,*E*)-1-(Allyloxy)pent-3-en-2-yl)oxy)-2-((tert-butyldimethylsilyl)oxy)-3-methyl-4-((triethylsilyl)oxy)phenyl)-5-methylocta-1,7-dien-3-yl methyl carbonate (**S12**) SI-48
46. (3*R*,5*S*,6*R*)-6-(5-(((*S*,*E*)-1-(Allyloxy)pent-3-en-2-yl)oxy)-2-((tert-butyldimethylsilyl)oxy)-4-hydroxy-3-methylphenyl)-5-methylocta-1,7-dien-3-yl methyl carbonate (**30'**) SI-49
47. (1*R*,2*S*,4*S*,5*R*)-9-(((*S*,*E*)-1-(Allyloxy)pent-3-en-2-yl)oxy)-6-((tert-butyldimethylsilyl)oxy)-2,7-dimethyl-1,4-divinylspiro[4.5]deca-6,9-dien-8-one (**29a**) SI-50
48. (3*R*,5*S*,6*R*)-6-(5-(((*S*,*E*)-1-(allyloxy)pent-3-en-2-yl)oxy)-2-((tert-butyldimethylsilyl)oxy)-3-methyl-4-((triethylsilyl)oxy)phenyl)-5-methylocta-1,7-dien-3-yl 4-nitrobenzoate (**41**) SI-51
49. 6-(((*S*,*E*)-1-(allyloxy)pent-3-en-2-yl)oxy)-3-((tert-butyldimethylsilyl)oxy)-4-((3*R*,4*S*,6*R*)-6-hydroxy-4-methylocta-1,7-dien-3-yl)-2-methylphenol (**42**) SI-52
50. (1*R*,2*S*,4*S*,5*R*)-9-(((*S*,*E*)-1-(allyloxy)pent-3-en-2-yl)oxy)-8-hydroxy-2,7-dimethyl-1,4-divinylspiro[4.5]deca-7,9-dien-6-one (**43**) SI-53

Structure determination by single-crystal X-ray diffraction SI-56

Experimental SI-56
Table 1 ..... SI-57
Figure. 1. Molecular structure of **S7** with displacement ellipsoids drawn at the 50% probability level. SI-58
Figure 2. Molecular structures of the two independent molecules of **9** with displacement ellipsoids drawn at the 50% probability level. SI-59
Figure 3. Molecular structure of **7-epi-8** with displacement ellipsoids drawn at the 50% probability level. SI-60
Figure 4. Molecular structure of **36** with displacement ellipsoids drawn at the 50% probability level. SI-61

General Information

The following general procedures were used in all reactions unless otherwise stated. Glassware was oven-dried at 115°C. Schlenk flasks were flame-dried. Oxygen- and moisture sensitive reactions were carried out under a slight argon overpressure using Schlenk techniques and in dry solvents. Sensitive liquids and solutions were transferred *via* double tipped cannula or syringes through rubber septa. All reactions were stirred magnetically unless otherwise stated. The solvents used were purified and dried according to common procedures as follows. Dry methylene chloride and diethyl ether were retrieved from an Innovative Technologies PureSolv system. Dry tetrahydrofuran was pre-dried using an Innovative Technologies PureSolv system, refluxed over sodium/benzophenone and freshly distilled. Dry toluene, hexane, ethyl acetate and acetonitrile were p.a. and HPLC grade, respectively, refluxed over sodium and freshly distilled. Dry DMF and DMSO were used as purchased. Ethyl acetate, petroleum ether and diethyl ether (technical grade) were distilled prior to use. Methylene chloride (technical grade) was distilled from potassium carbonate prior to use. All other solvents used were p.a. or HPLC grade. All reagents were used as received. ^{1}H and ^{13}C NMR spectra were recorded on a Bruker AC 400 at 400 and 101 MHz; AC 600 at 600 and 151 MHz or AC 200 at 200 MHz using the solvent peak as reference. ^{13}C NMR spectra were run in proton-decoupled mode. Multiplicities of ^{1}H signals were referred to as s (singlet), d (doublet), t (triplet), q (quartet) and more complex patterns or m (multiplet). IR spectra were recorded on a Perkin Elmer Spectrum 65 FT IR Spectrometer equipped with a specac MK II Golden Gate Single Reflection ATR unit. TLC-analysis was done with precoated aluminium-backed plates (Silica gel 60 F254, Merck). Compounds were visualized by submerging in: an acidic phosphomolybdic acid / Cerium sulphate solution, $KMnO_4$, Vanillin or Anisaldehyde and dried with a heat gun. Column chromatography was carried out with silica gel Merck 60. Eluent

systems refer to volumetric ratios, e.g., 4:1 =80 %: 20 %. Specific rotations were measured on an Anton Parr MCP 500 polarimeter in at 20°C and 589 nm. HR-MS measurements were carried out in acetonitrile, methanol, water or a mixture on an Agilent 1100/1200 HPLC with binary pumps, a degassed and a column thermostat and an Agilent 6230 AJS ESI-TOF mass spectrometer. Data analysis was carried out using MassHunter Qualitative Analysis software (Agilent). Single crystal X-ray diffraction (SCXRD) revealed the determination of the relative and absolute configurations for compounds **9**, **S7**, **7-epi-8** and **36**. For compound **40**, single crystals diffracted very poorly (significantly below the recommended resolution limit) but allowed to determine the stereochemistry.

Compound **29** vs **29a**

When carrying out the reaction the TLC of the reaction mixture and the one after work up (evaporation) showed significant differences. While in the TLC of the reaction mixture (left) the formed product is more polar than the starting material. After evaporation of CH_2Cl_2 the majority of the newly formed entity is significantly less polar on TLC (right) than the starting material. As it was not possible to isolate the initially formed product(s) we conducted the above-mentioned reaction in CD_2Cl_2 to measure NMR spectra without the necessity of solvent evaporation. Careful analysis of 2D NMR experiments of the reaction mixture allowed to extract the following data which was attributed to compound **29**:

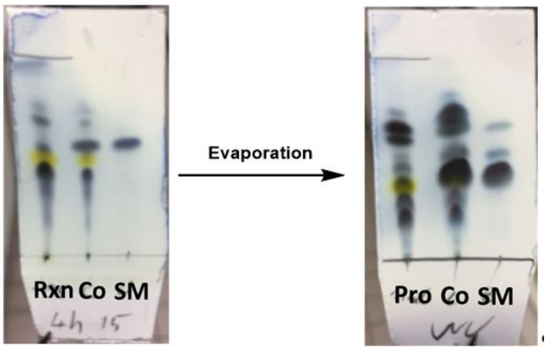

^{1}H-NMR (600 MHz, CD_2Cl_2): δ= 5.95 – 5.88 (m, 1H), 5.76 – 5.67 (m, 2H), 5.65 (s, 1H), 5.59 (ddd, *J* = 17.1, 10.3, 8.5 Hz, 1H), 5.45 – 5.39 (m, 2H), 5.30 – 5.26 (m, 1H), 5.17 (dq, *J* = 10.4, 1.5 Hz, 1H), 5.02 (ddd, *J* = 10.4, 1.8, 0.7 Hz, 1H), 4.90 – 4.79 (m, 2H), 4.58 – 4.53 (m, 1H), 4.06 – 3.97 (m, 2H), 3.67 – 3.59 (m, 1H), 3.56 – 3.51 (m, 1H), 2.79 (dd, *J* = 11.0, 8.5 Hz, 1H), 2.71 (m, 1H), 2.04 – 1.99 (m, 2H), 1.77 (s, 3H), 1.71 – 1.69 (m, 3H), 1.57 – 1.51 (m, 1H)., 1.05-1.01 (m, 12H), 0.34 (s, 3H), 0.31 (s, 3H); ^{13}C-NMR (151 MHz, CD_2Cl_2): δ= 184.1, 167.4, 145.9, 138.0, 137.4, 135.3, 130.3, 128.0, 124.6, 118.7, 117.0, 116.8, 115.7, 77.9, 72.9, 72.6, 58.0, 56.1, 41.7, 38.6, 30.1, 26.9, 18.0, 17.0, 11.1, -0.4, -1.6.

Comparison of the HMBC spectra of compounds **29**, **29a** and **43** allowed the following conclusion:

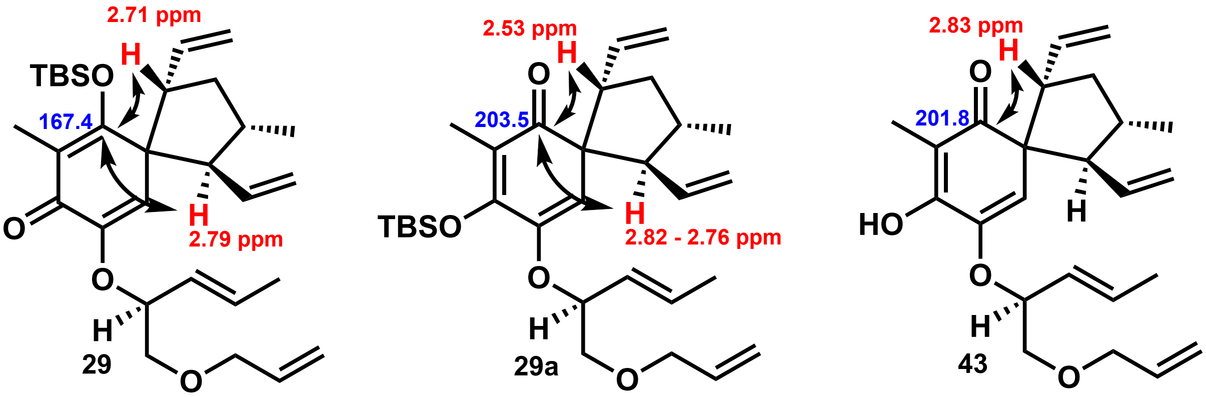

In **29**, the hydrogens at 2.71 and 2.79 ppm, respectively (in red) both correlate to a quaternary carbon at 167.4 belonging to the silyl-enolate (in blue). In compound **29a**, the same hydrogens at 2.53 and 2.82-2.76 ppm respectively correlate to a carbon at 203.5 ppm represented by the ketone (in blue). A similar observation was made in the spectra of compound **43**. Here, the hydrogen at 2.83 ppm (in red) correlates to the ketone functionality (in blue) at 201.8 ppm.
Although we are confident about the structural elucidation of the above-mentioned materials, unfortunately, these observations can neither explain the discrepancy with respect to behavior on silica, nor absolutely confirm the structure of compound **29**.

NMR spectra

1. 3-Methoxy-4-((triisopropylsilyl)oxy)benzaldehyde (S1)

^{1}H NMR (400 MHz, CDCl3)

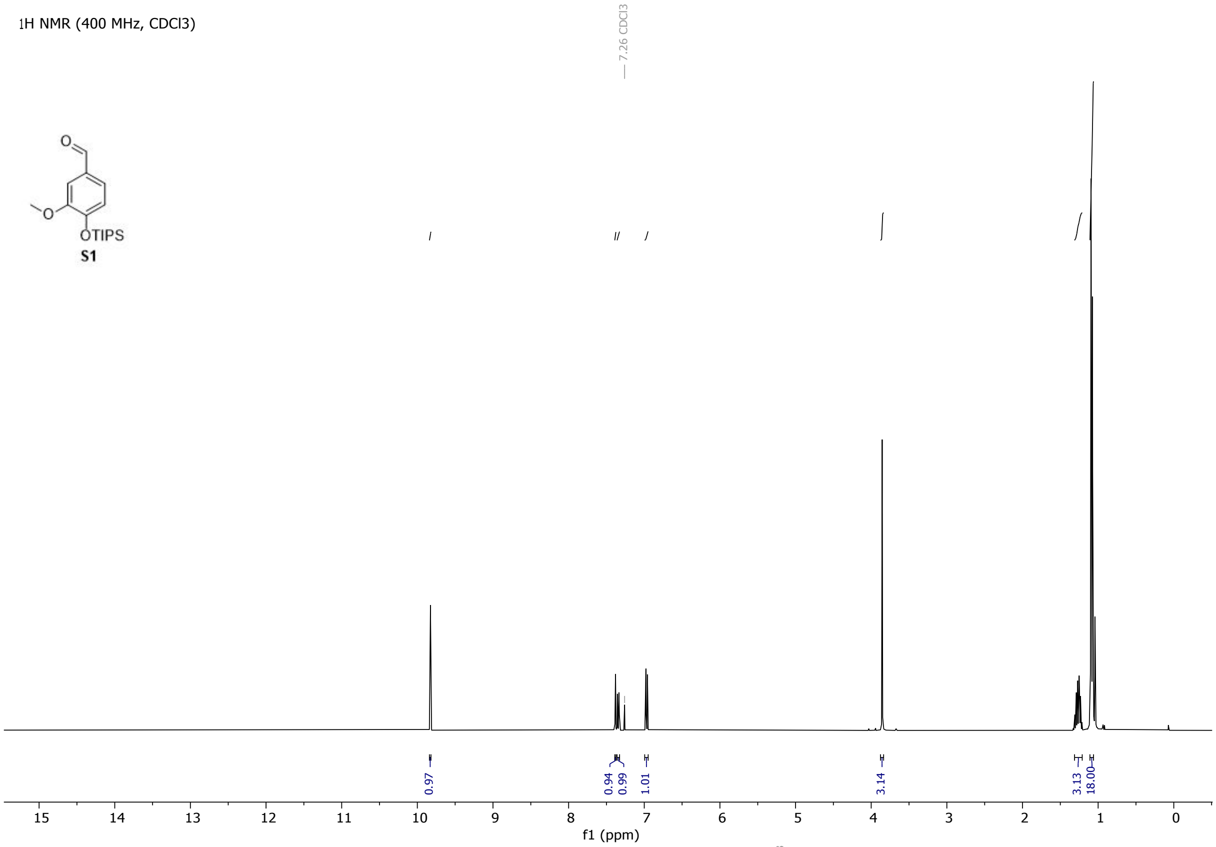

^{13}C{^{1}H} NMR (101 MHz, CDCl3)

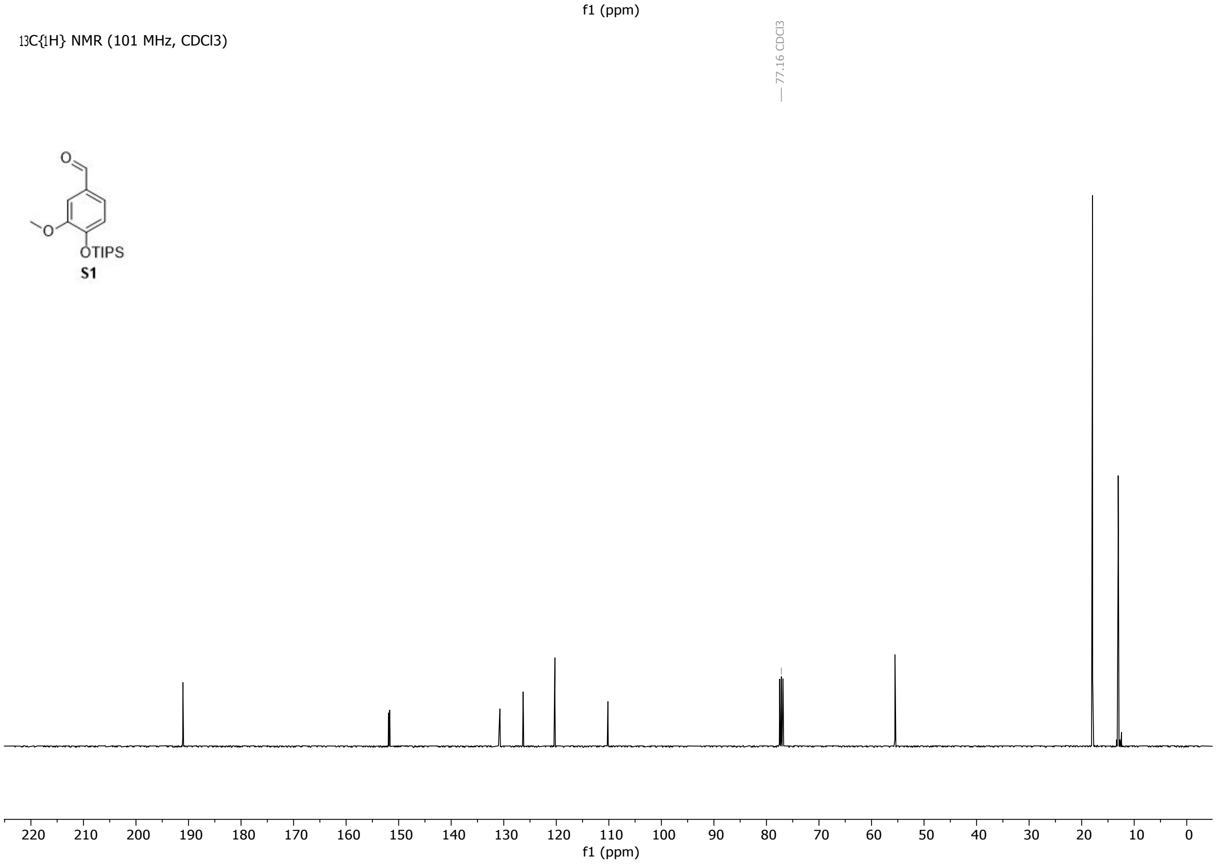

2. 3-Methoxy-4-((triisopropylsilyl)oxy)phenyl formate (S2)

1H NMR (400 MHz, CDCl3)

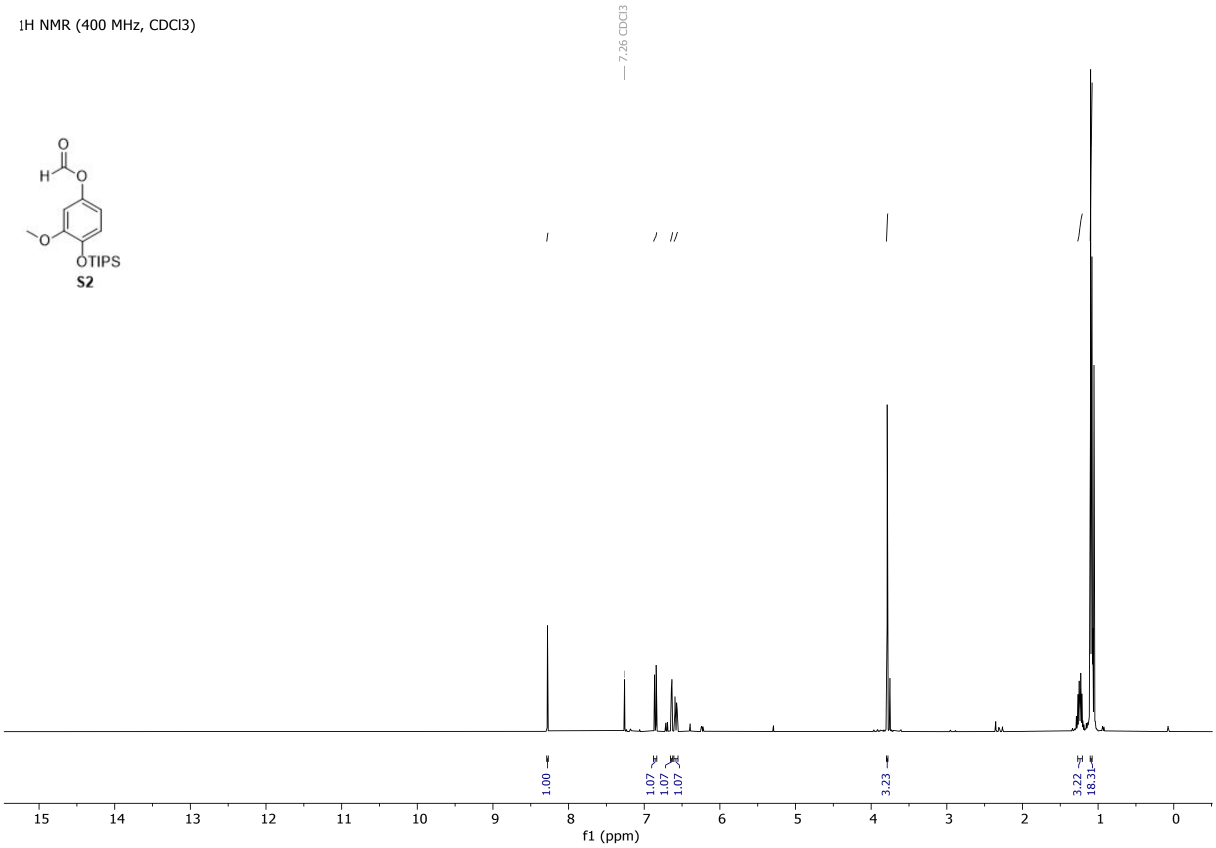

13C{1H} NMR (101 MHz, CDCl3)

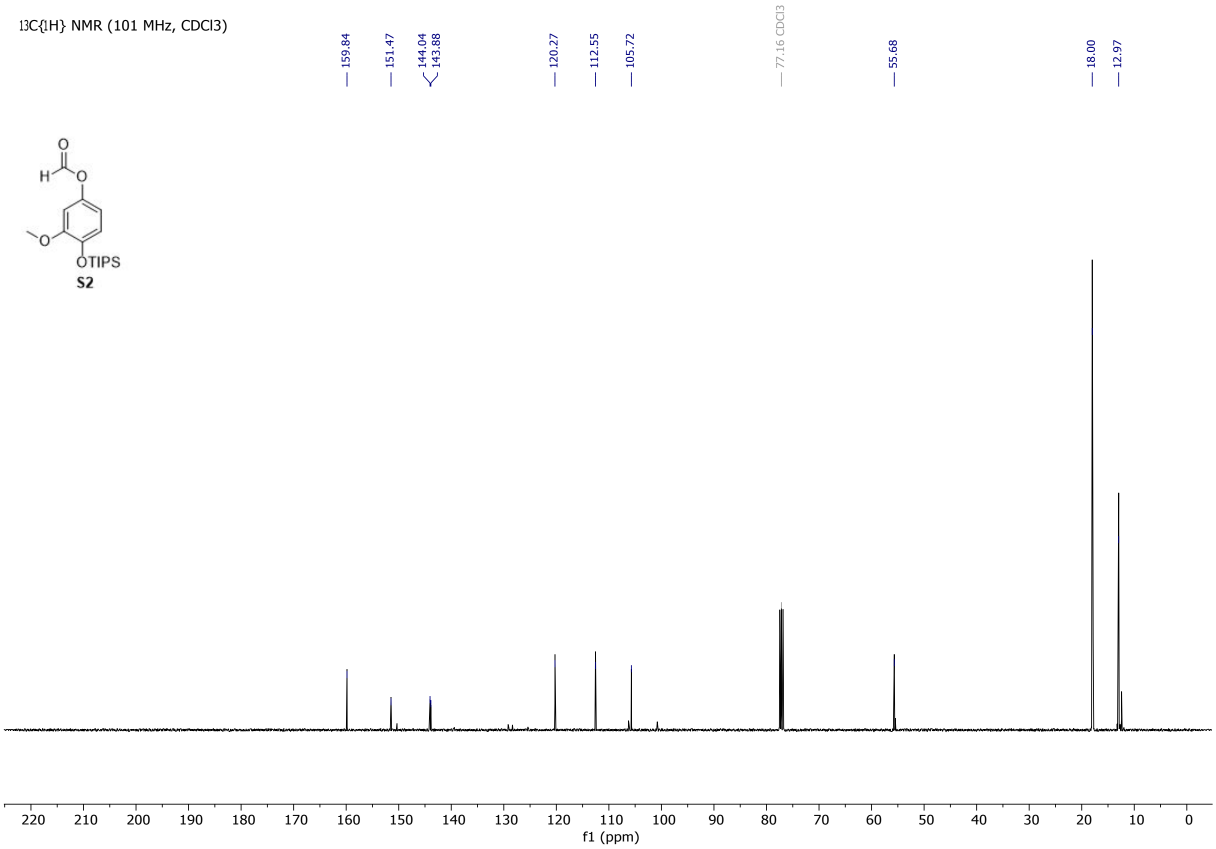

3. 3-Methoxy-4-((triisopropylsilyl)oxy)phenol (S3)

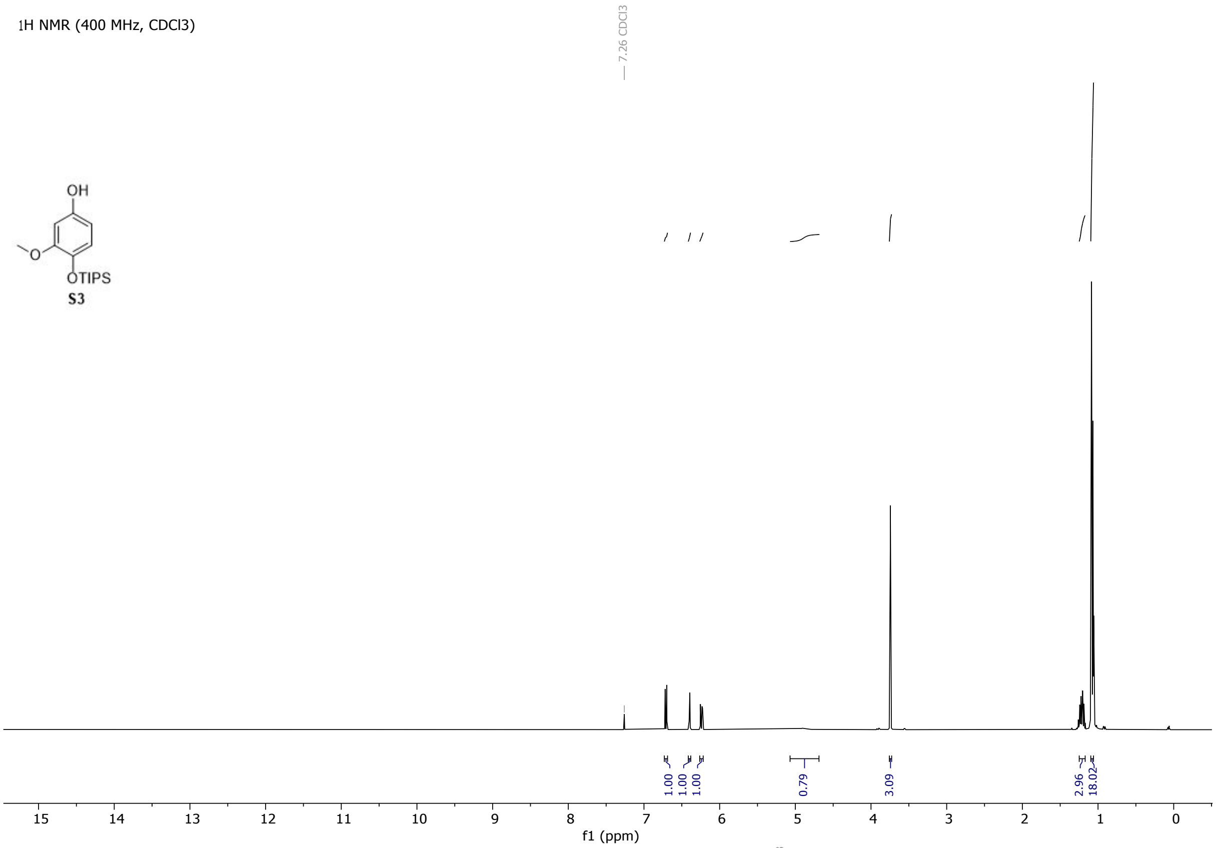

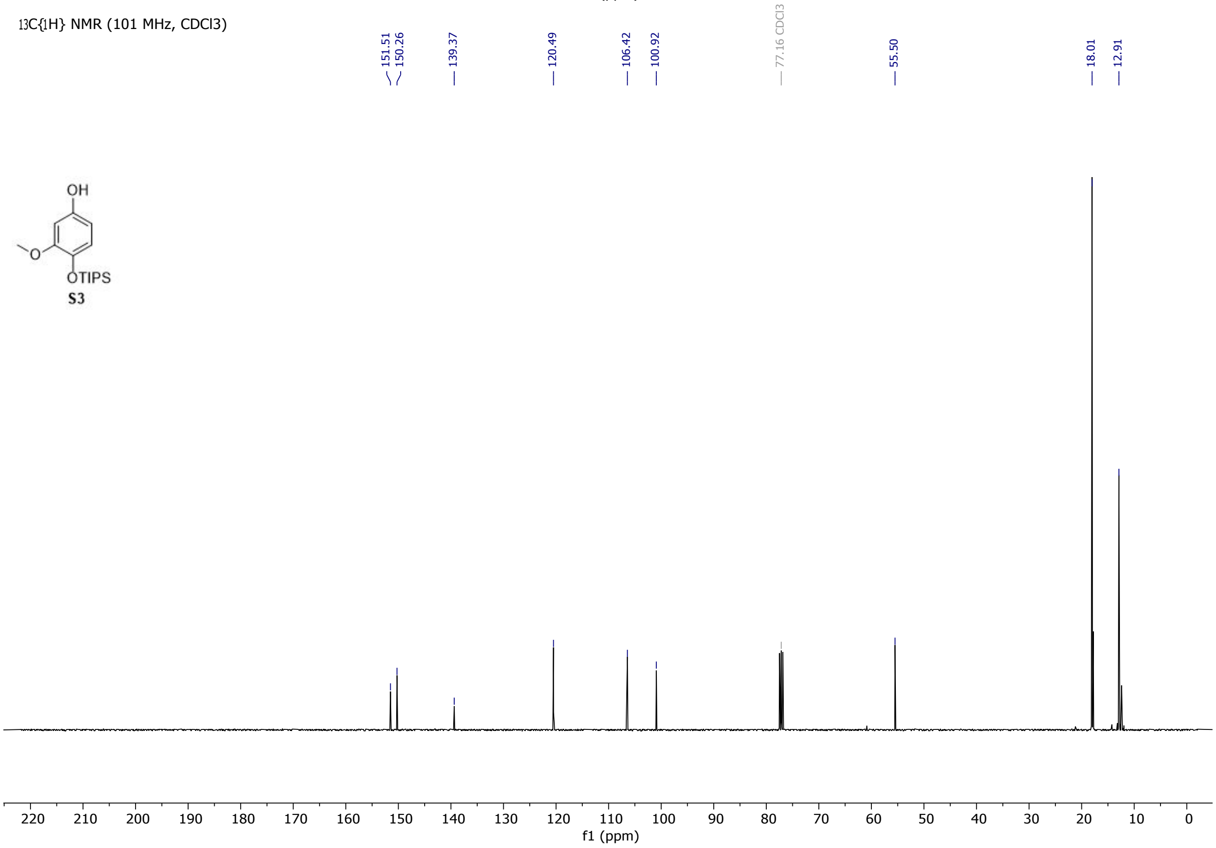

4. Triisopropyl(2-methoxy-4-(methoxymethoxy)phenoxy)silane (S4)

1H NMR (400 MHz, CDCl3)

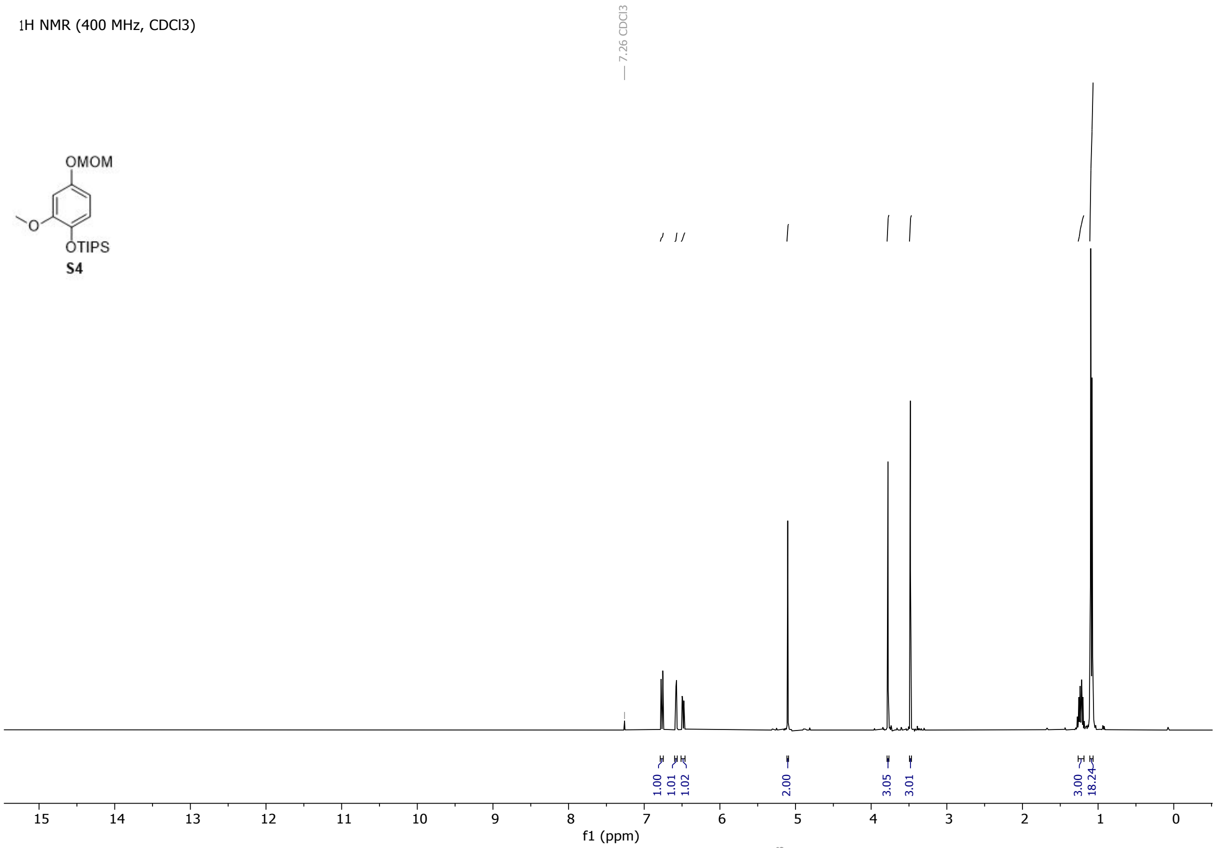

13C{1H} NMR (101 MHz, CDCl3)

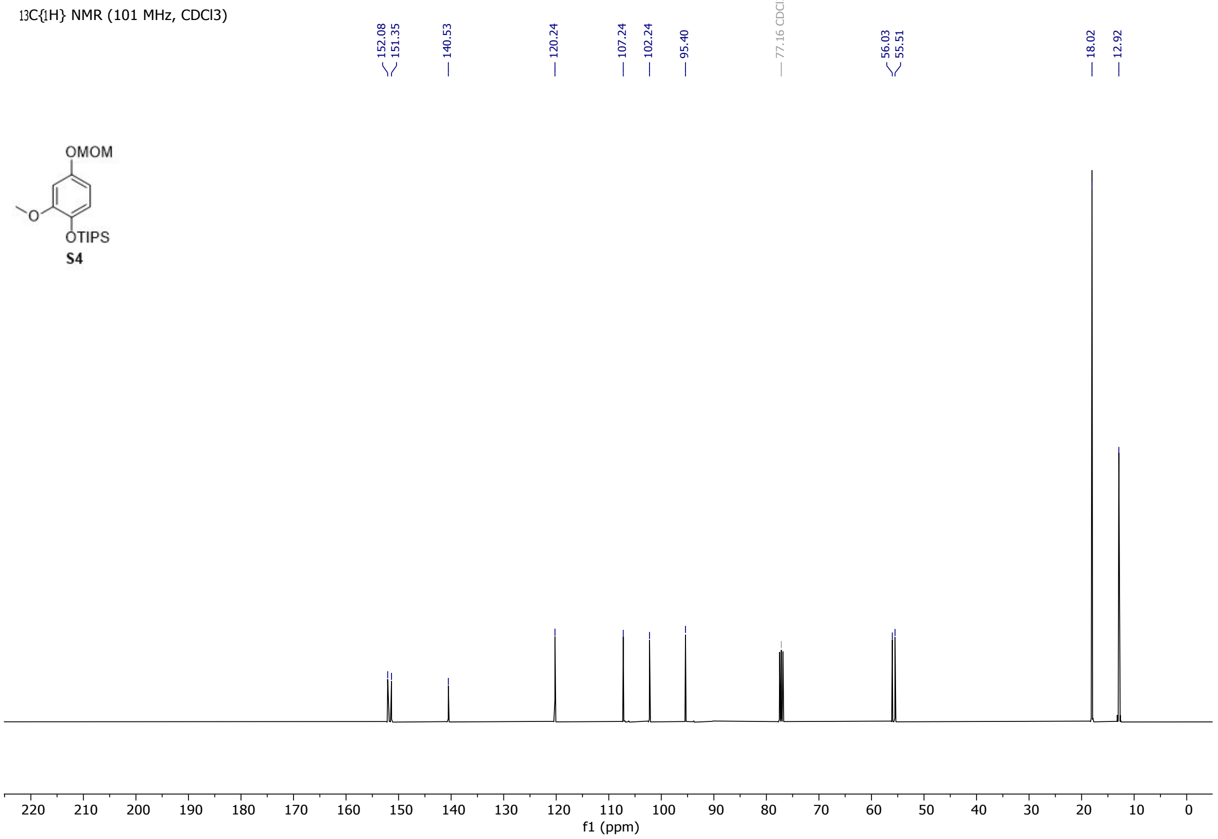

5. Triisopropyl(2-methoxy-4-(methoxymethoxy)-3-methylphenoxy)silane (13)

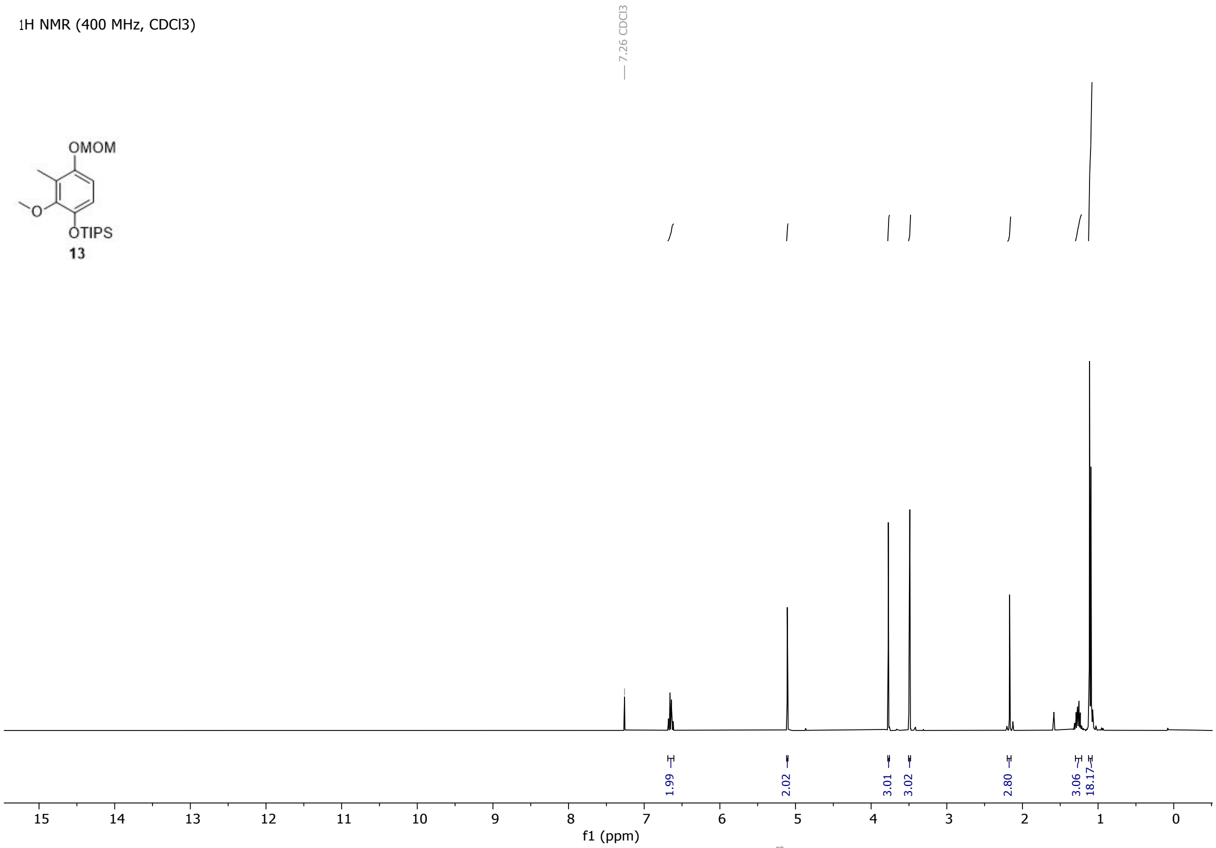

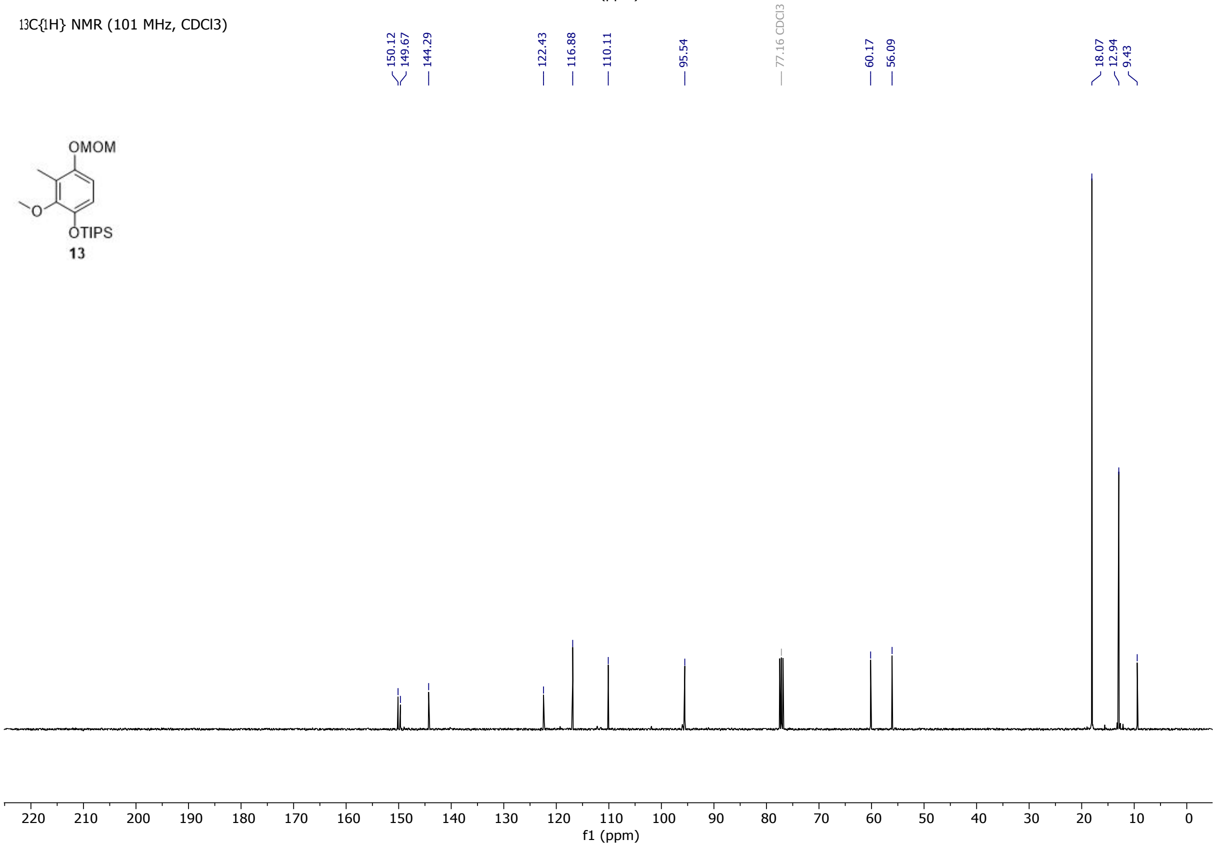

6. 3-Methoxy-2-methyl-4-((triisopropylsilyl)oxy)phenol (14)

1H NMR (400 MHz, CDCl3)

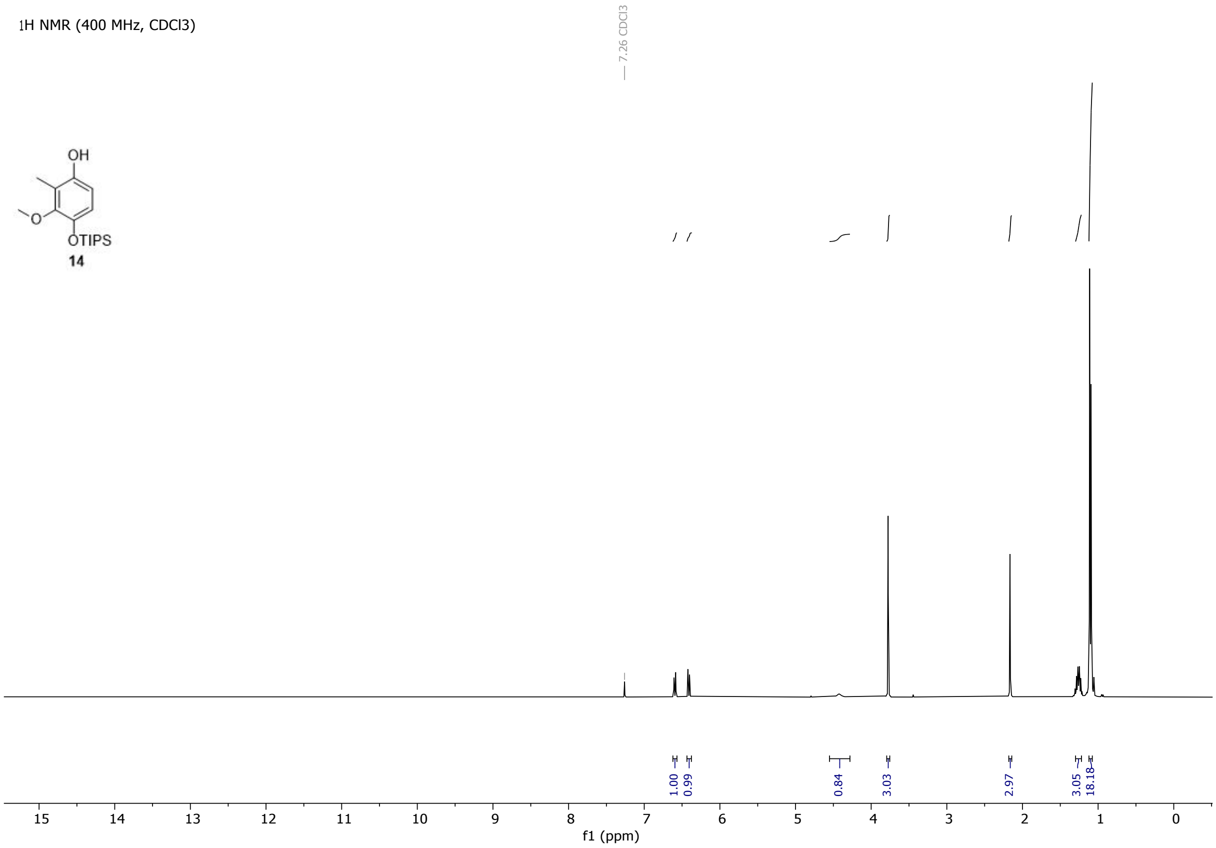

13C{1H} NMR (101 MHz, CDCl3)

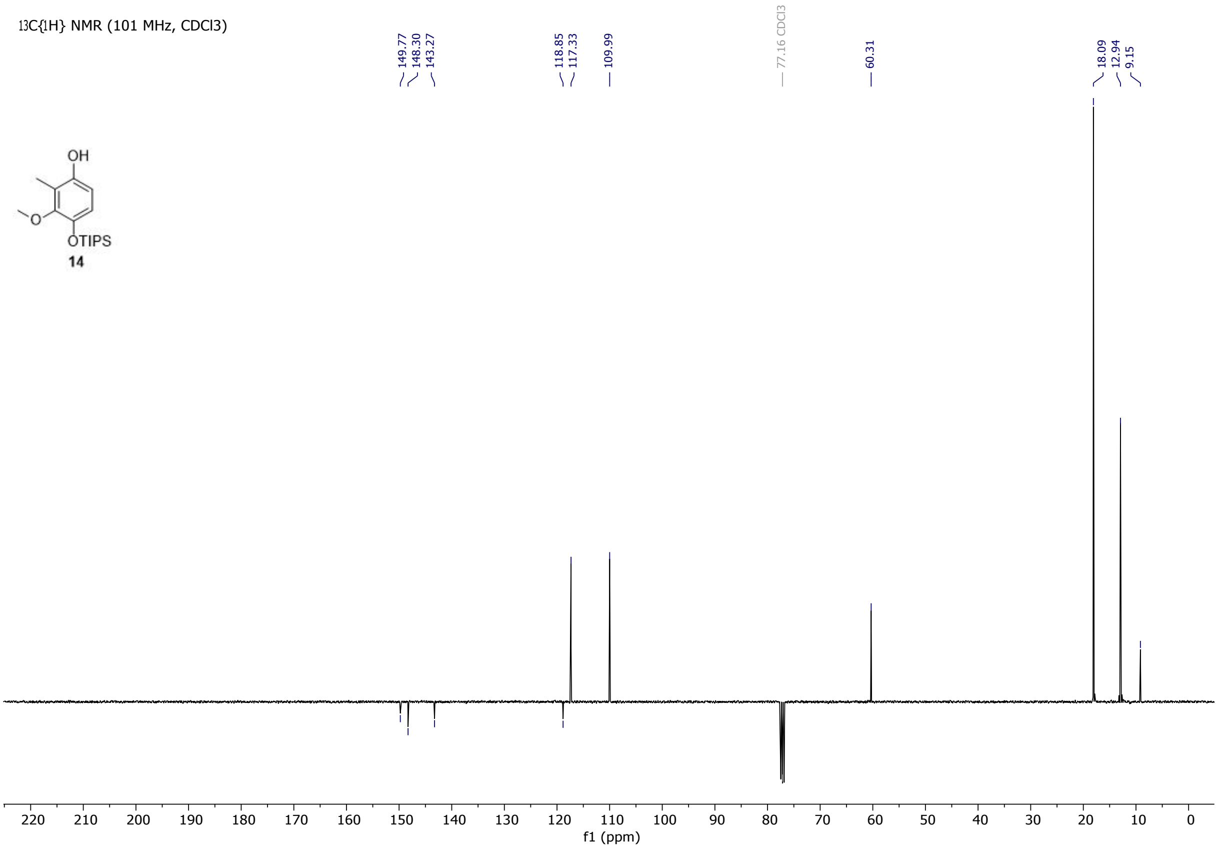

7. (*E*)-Triisopropyl(2-methoxy-3-methyl-4-(penta-2,4-dien-1-yloxy)phenoxy)silane (15)

^{1}H NMR (400 MHz, CDCl$_3$)

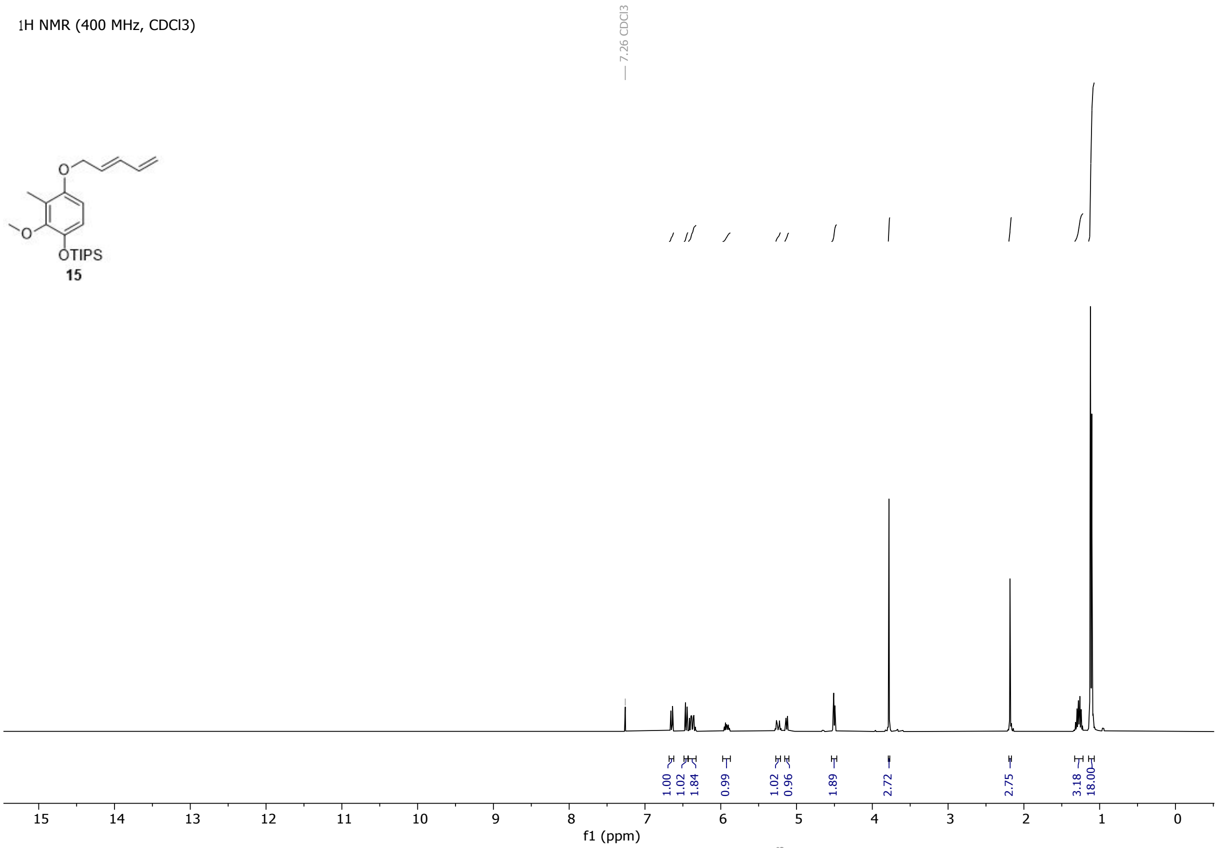

^{13}C{^{1}H} NMR (101 MHz, CDCl$_3$)

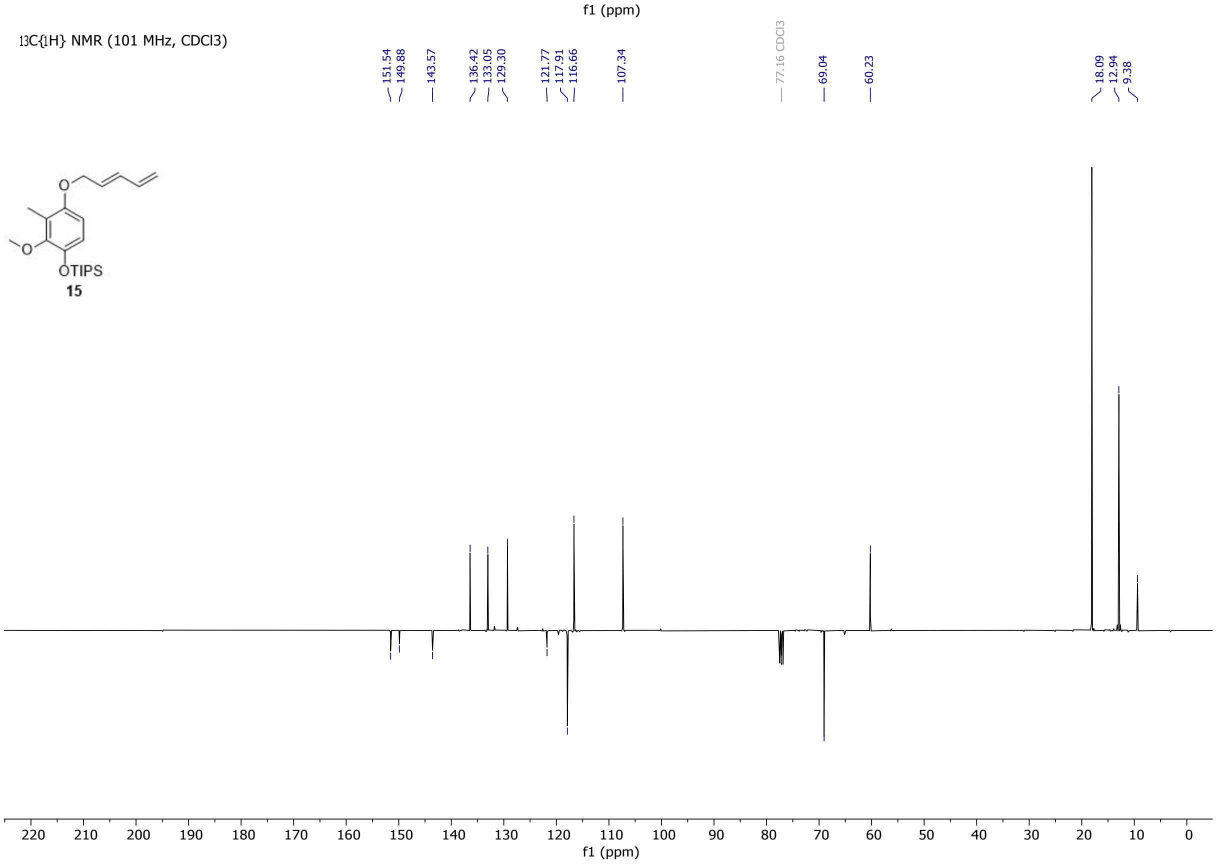

8. (*E*)-2-methoxy-3-methyl-4-(penta-2,4-dien-1-yloxy)phenol (S5)

1H NMR (400 MHz, CDCl3)

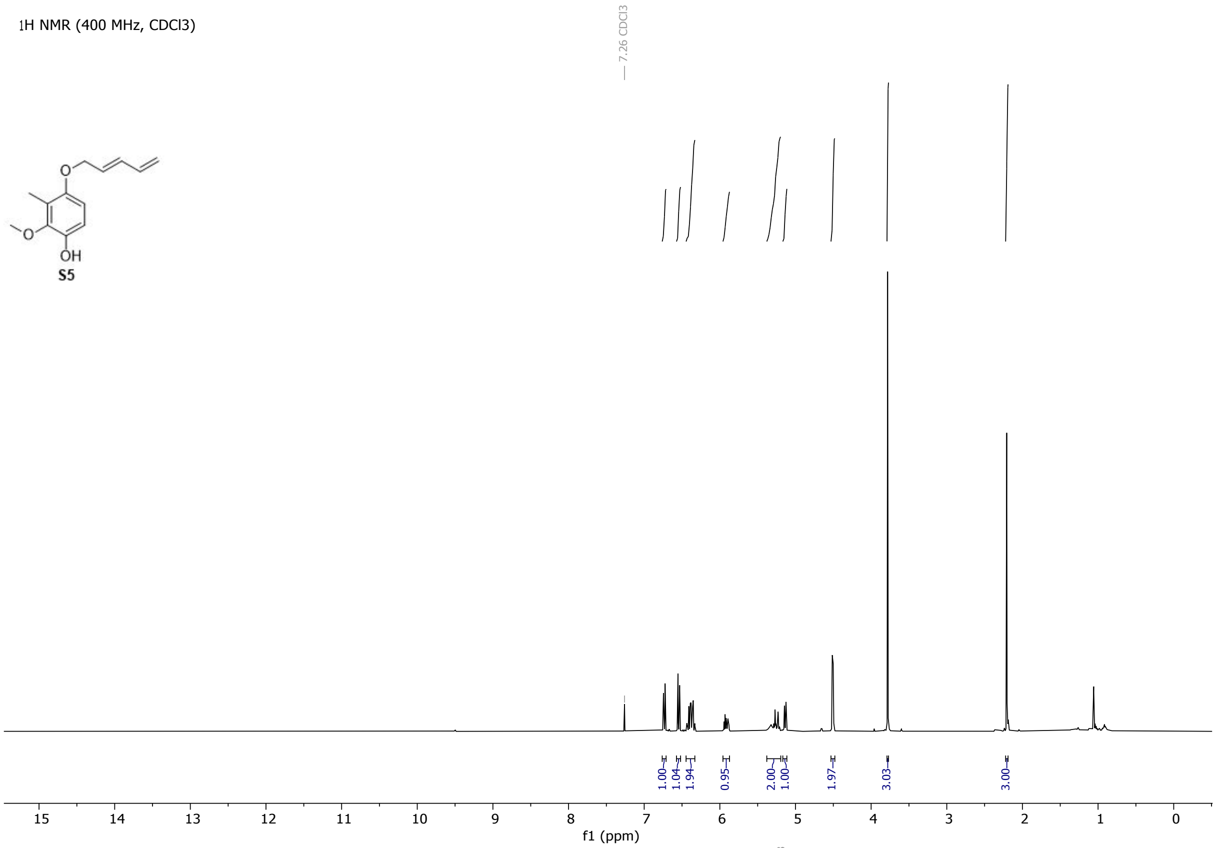

13C{1H} NMR (101 MHz, CDCl3)

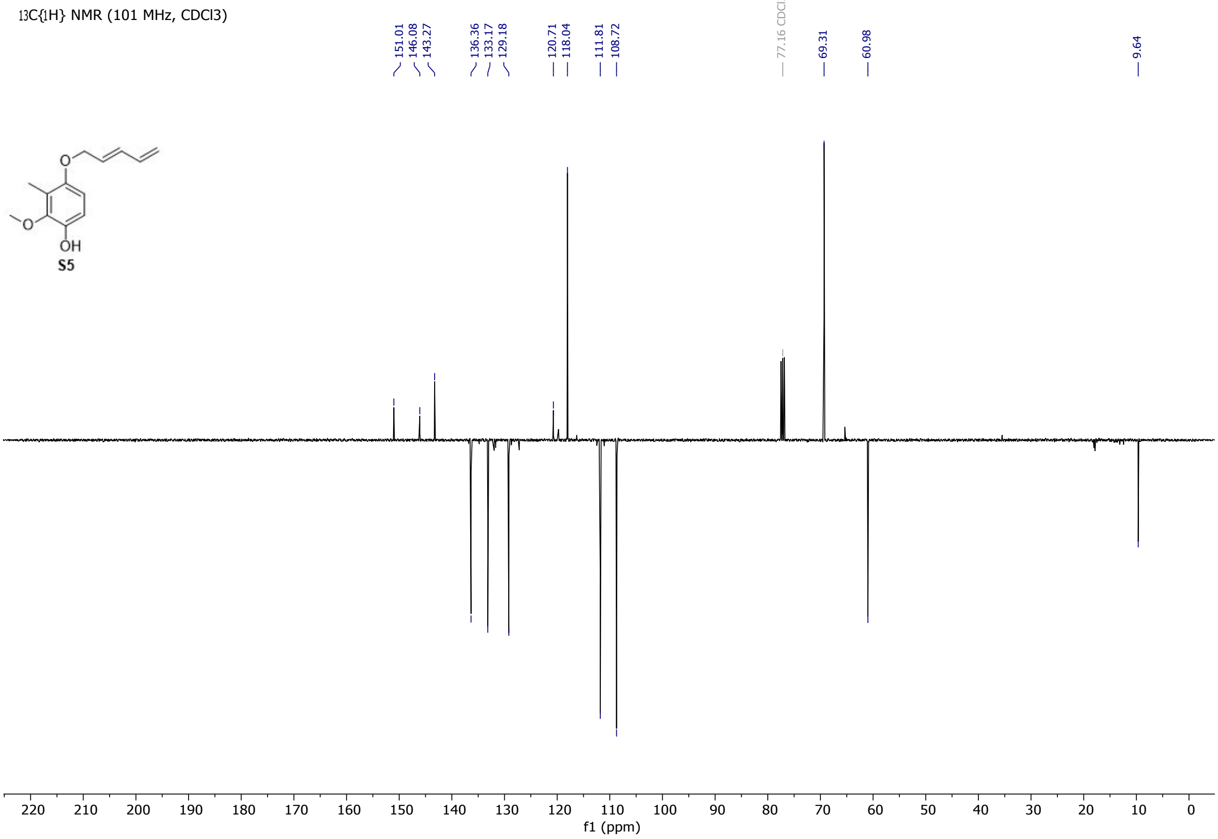

9. (*E*)-Triethyl(2-methoxy-3-methyl-4-(penta-2,4-dien-1-yloxy)phenoxy)silane (16)

1H NMR (400 MHz, CDCl3)

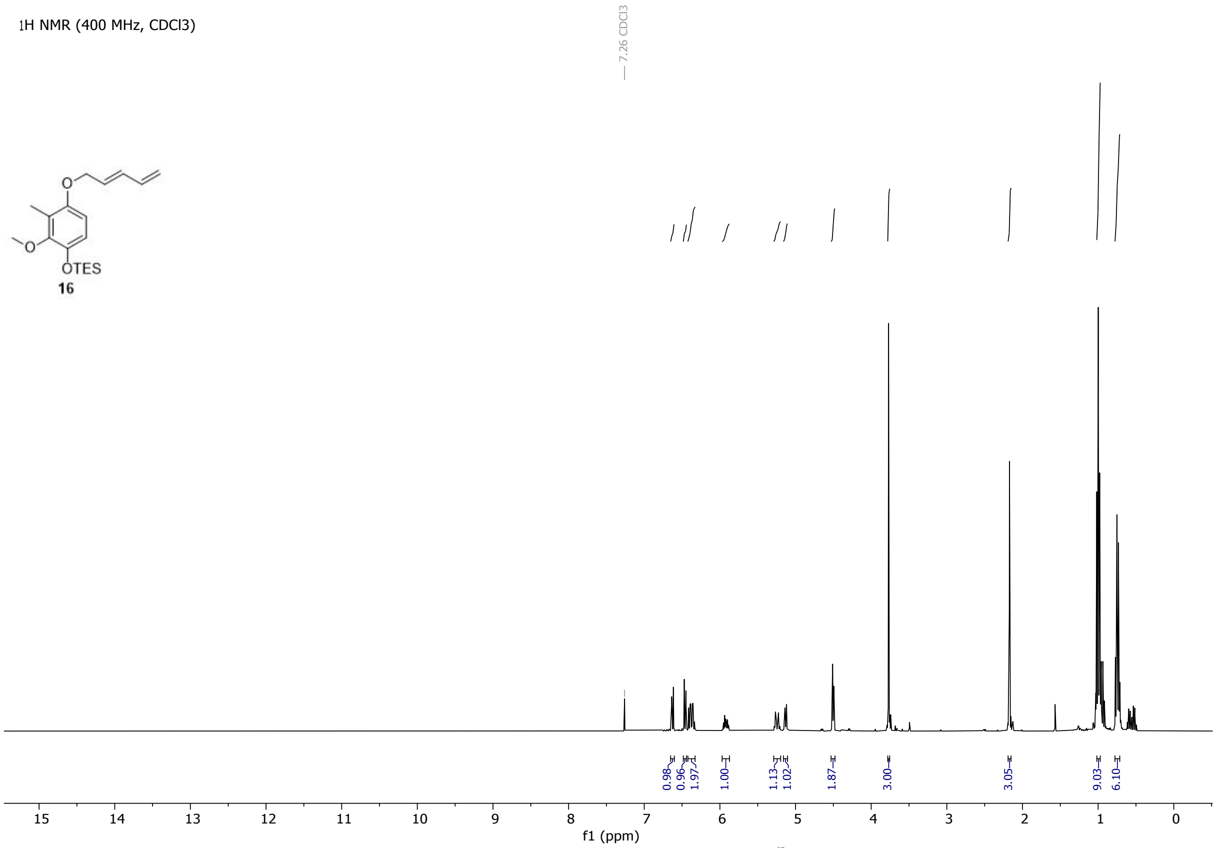

13C{1H} NMR (101 MHz, CDCl3)

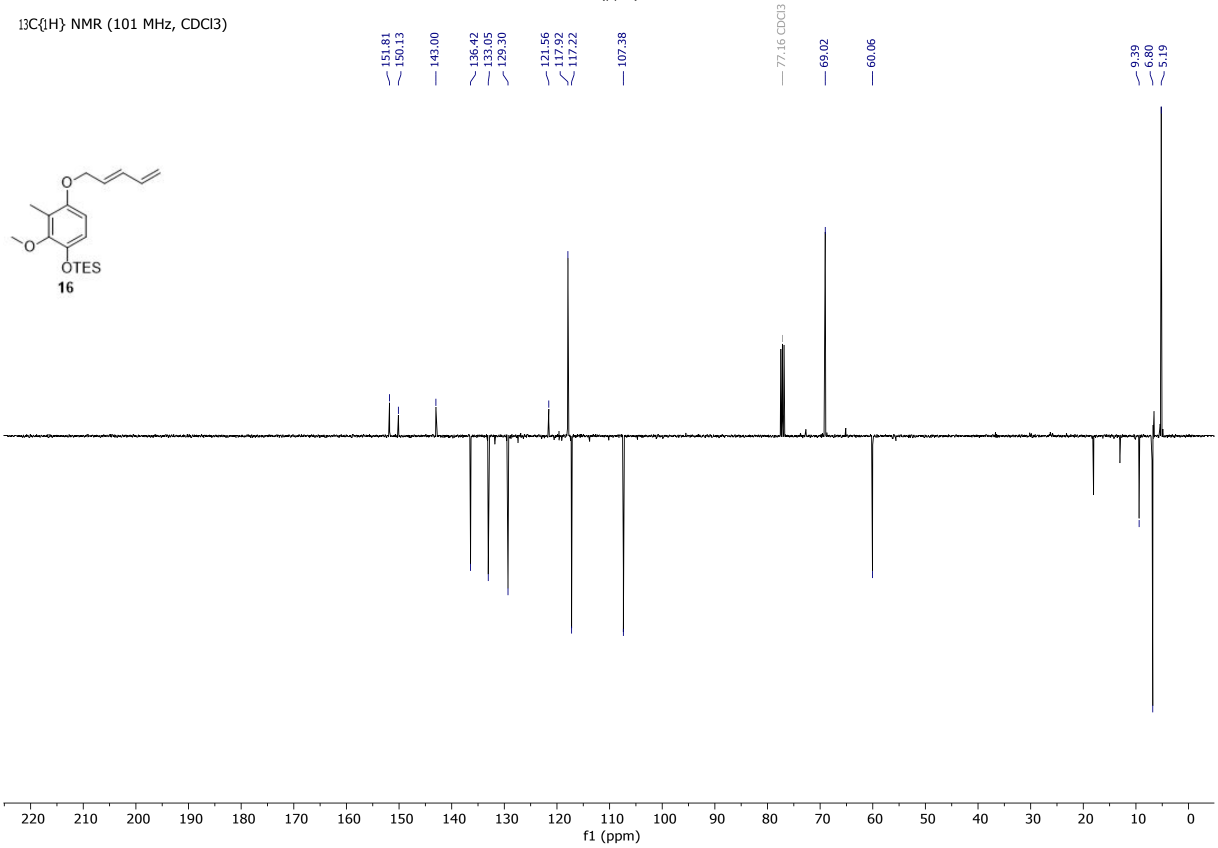

10. 3-Methoxy-2-methyl-6-(penta-1,4-dien-3-yl)-4-((triethylsilyl)oxy)phenol (17)

1H NMR (400 MHz, CDCl3)

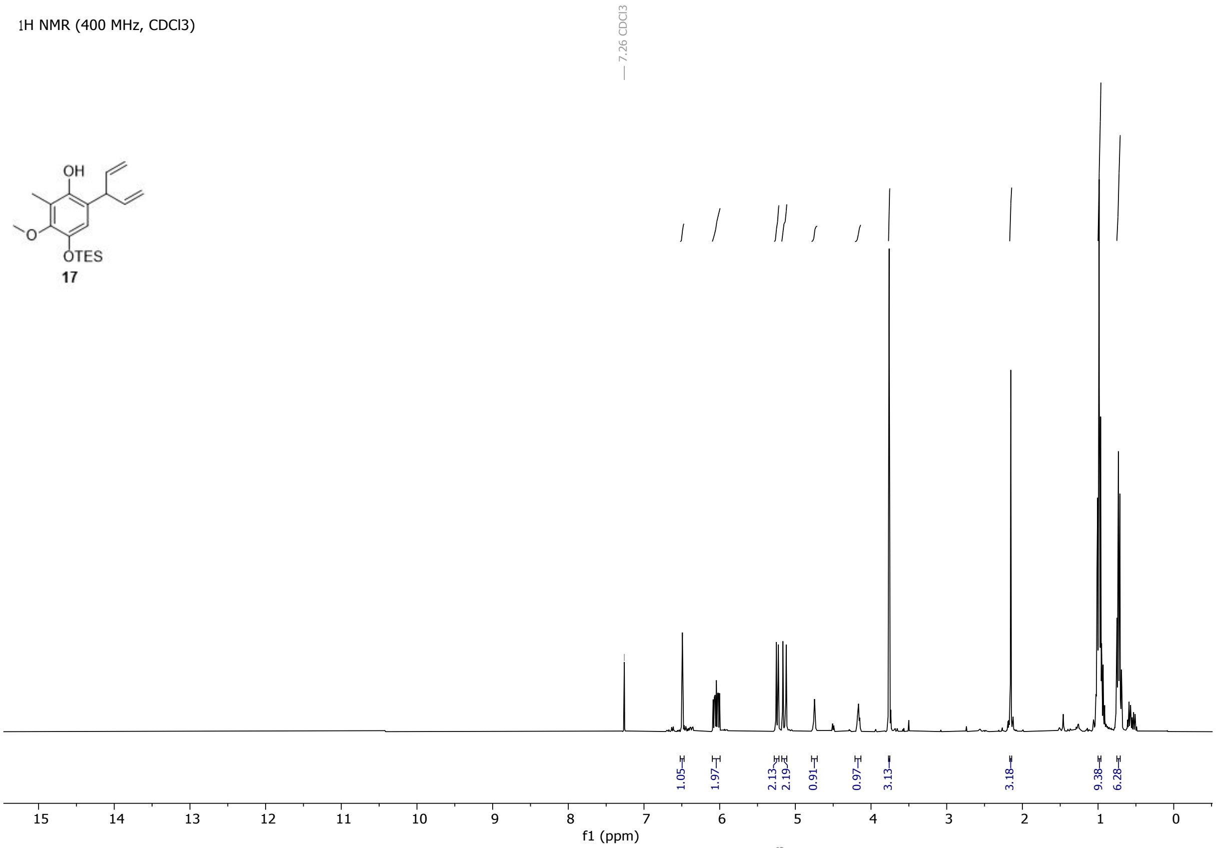

13C{1H} NMR (101 MHz, CDCl3)

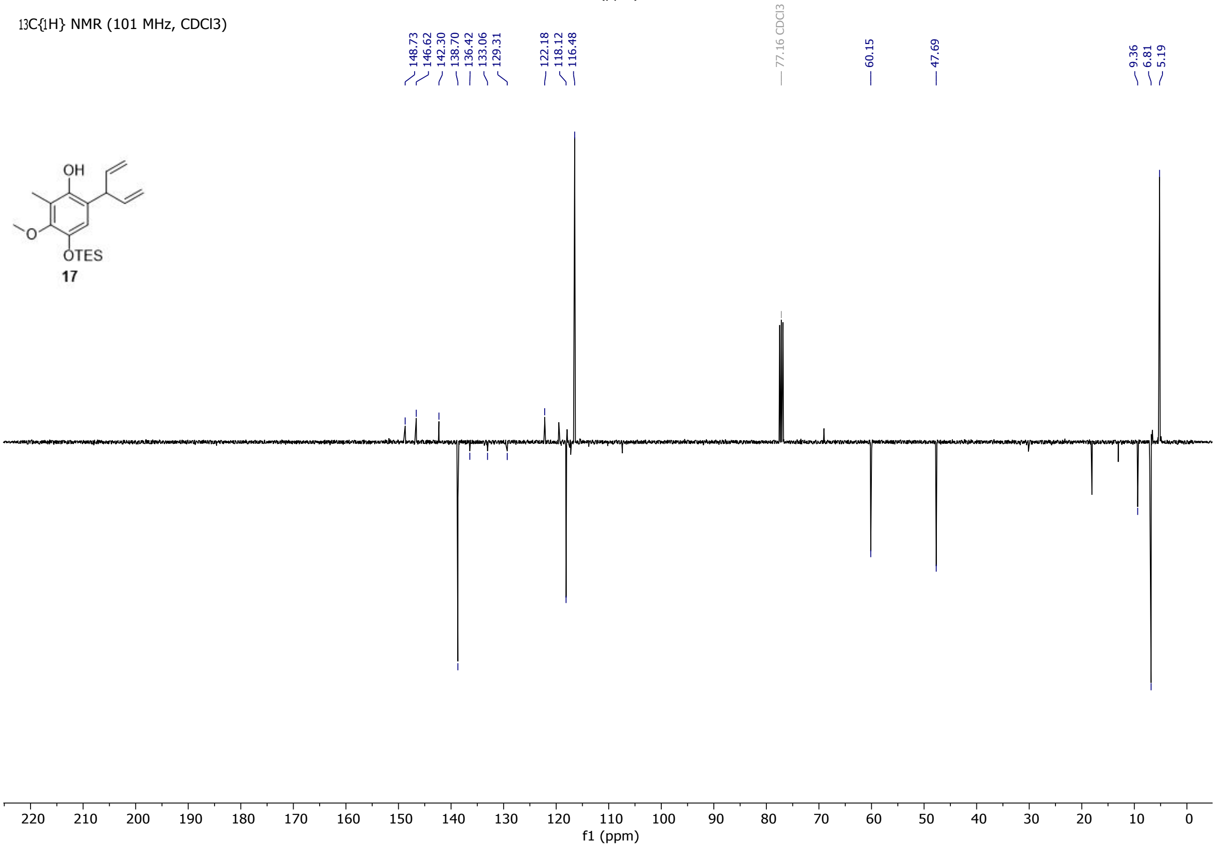

11. Triethyl(2-methoxy-3-methyl-5-(penta-1,4-dien-3-yl)-4-((triisopropylsilyl)oxy)phenoxy)silane (18)

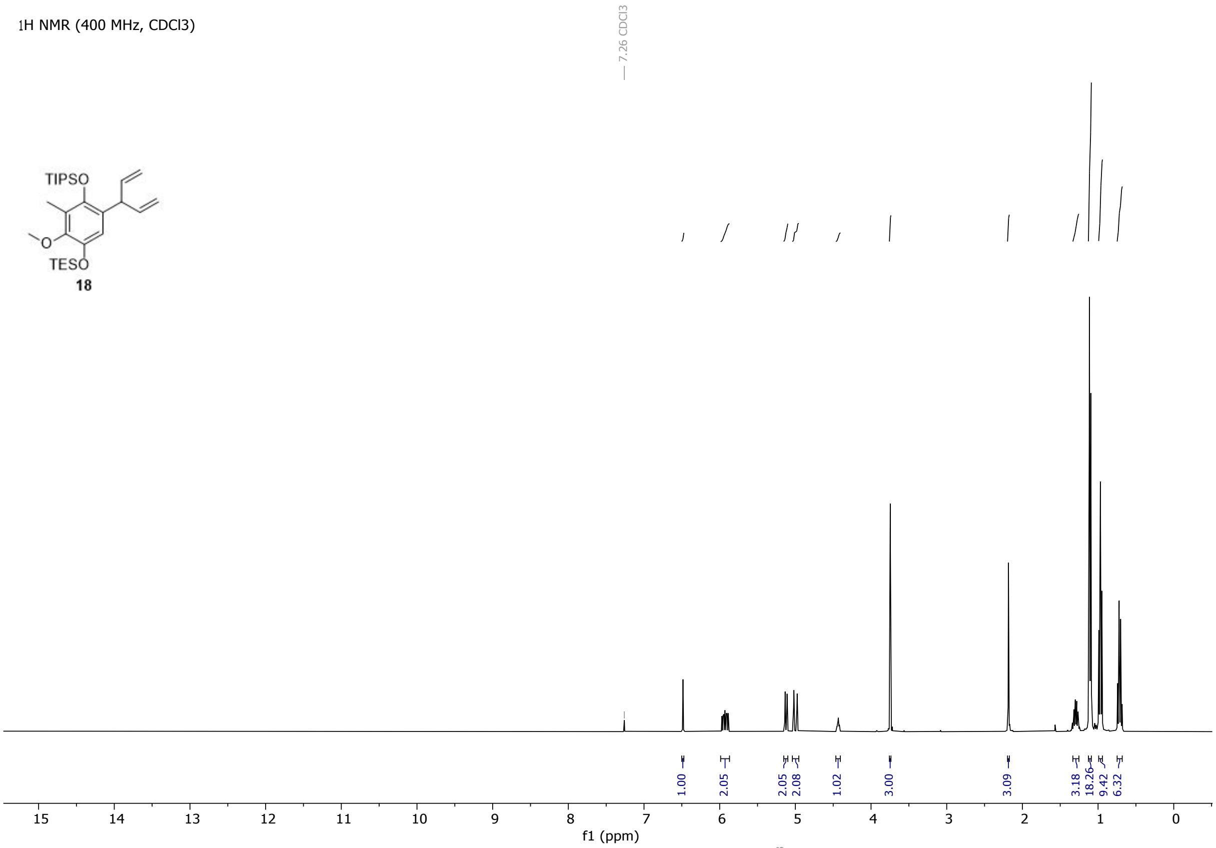

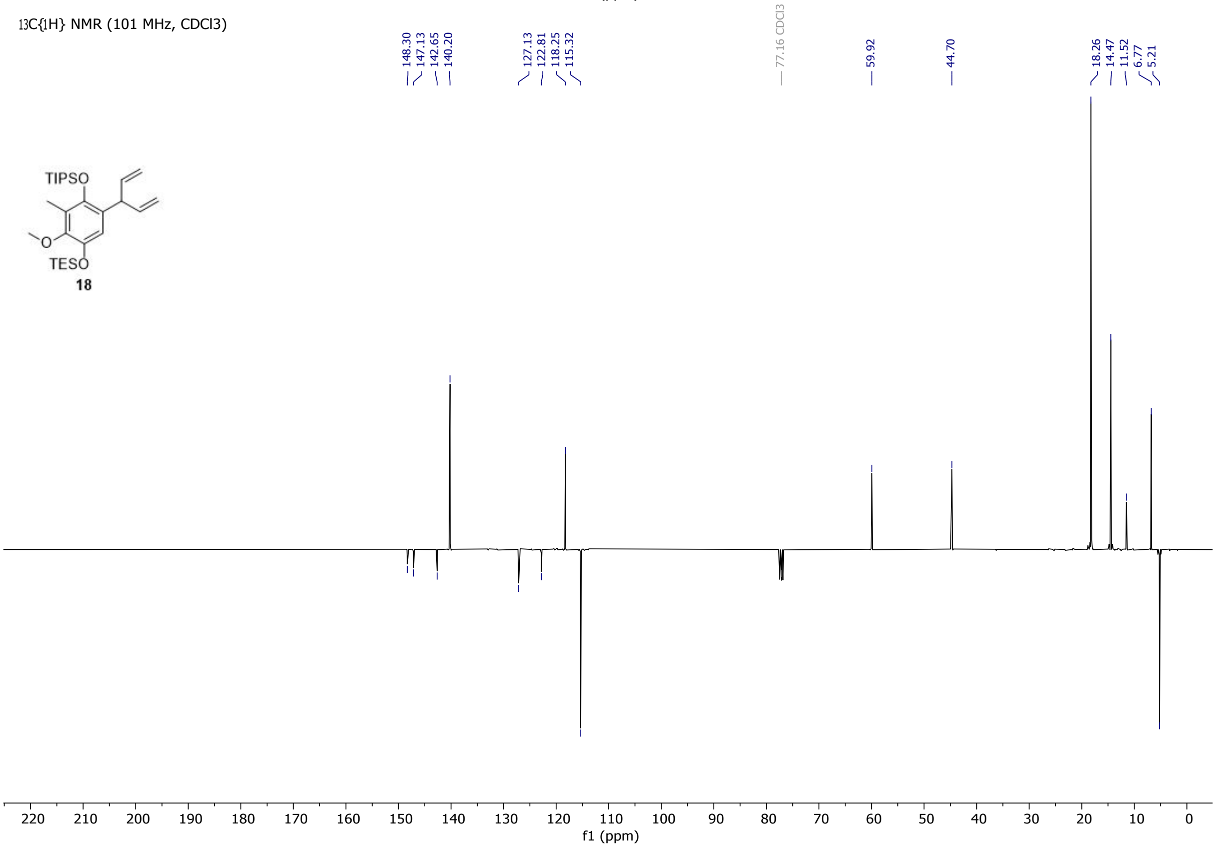

12. 2-Methoxy-3-methyl-5-(penta-1,4-dien-3-yl)-4-((triisopropylsilyl)oxy)phenol (11)

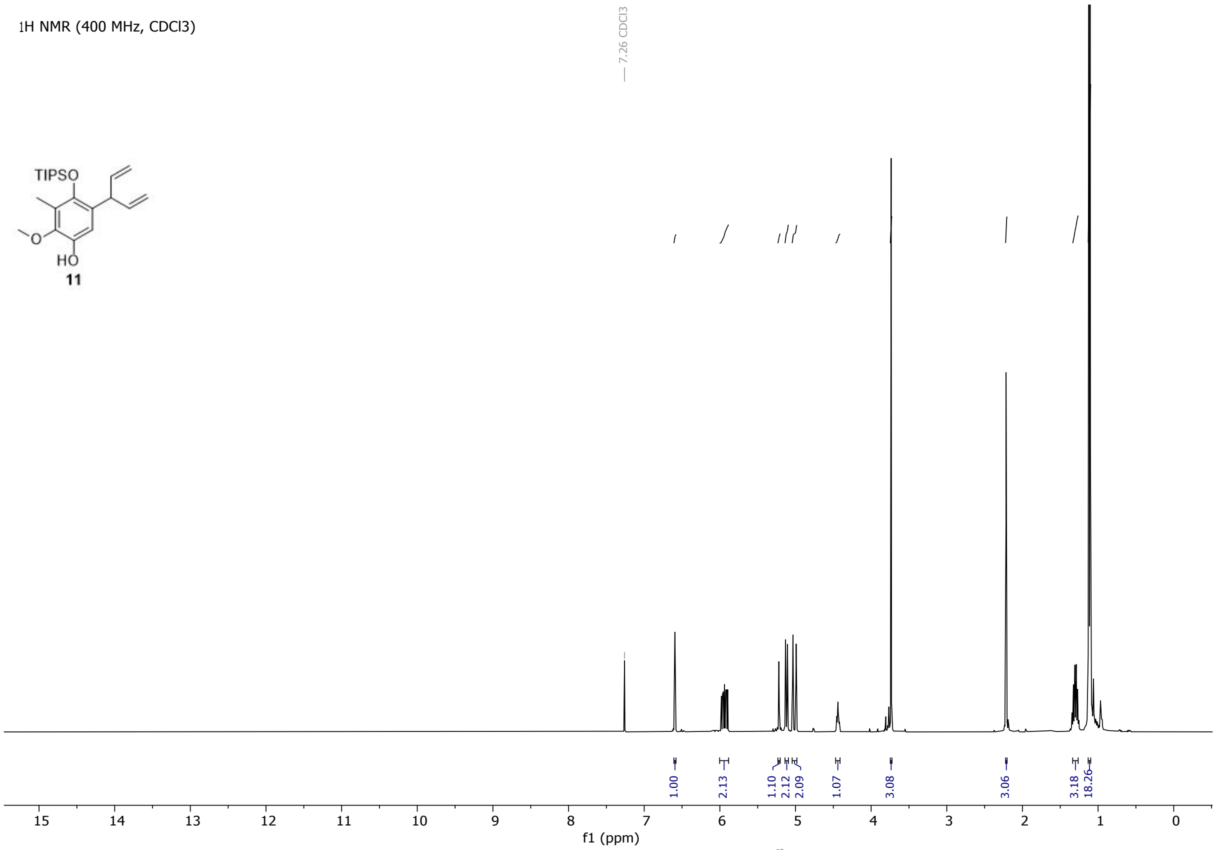

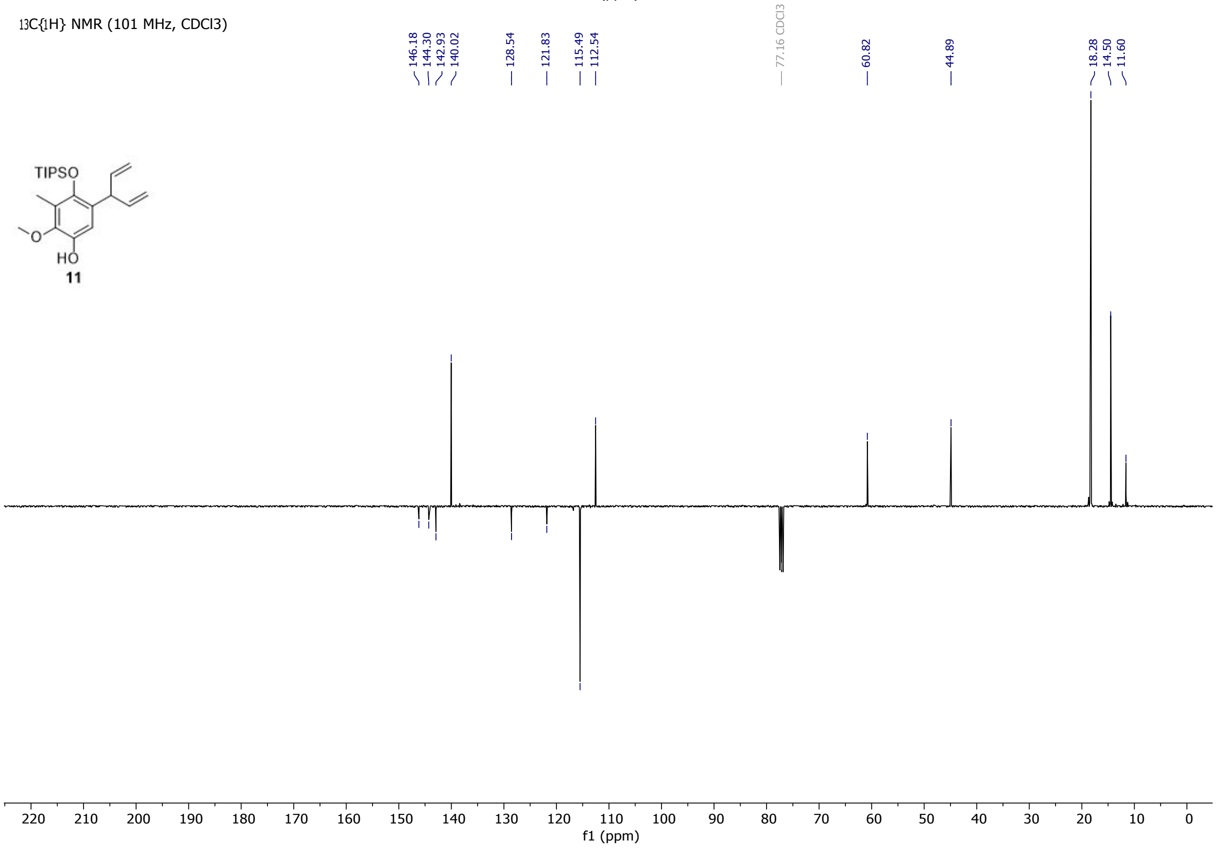

13. (*S,E*)-(4-((1-(Allyloxy)pent-3-en-2-yl)oxy)-3-methoxy-2-methyl-6-(penta-1,4-dien-3-yl)phenoxy)triisopropylsilane (19)

1H NMR (400 MHz, CDCl3)

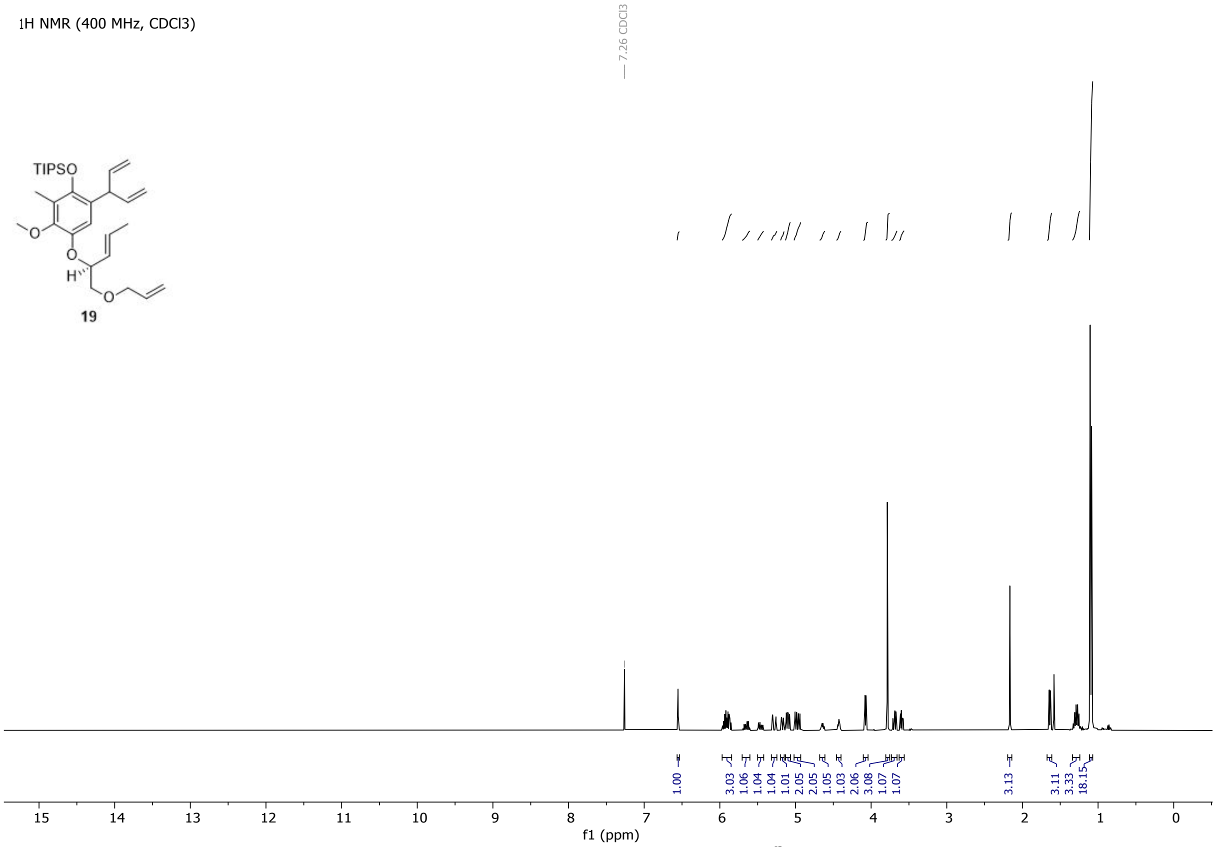

13C{1H} NMR (101 MHz, CDCl3)

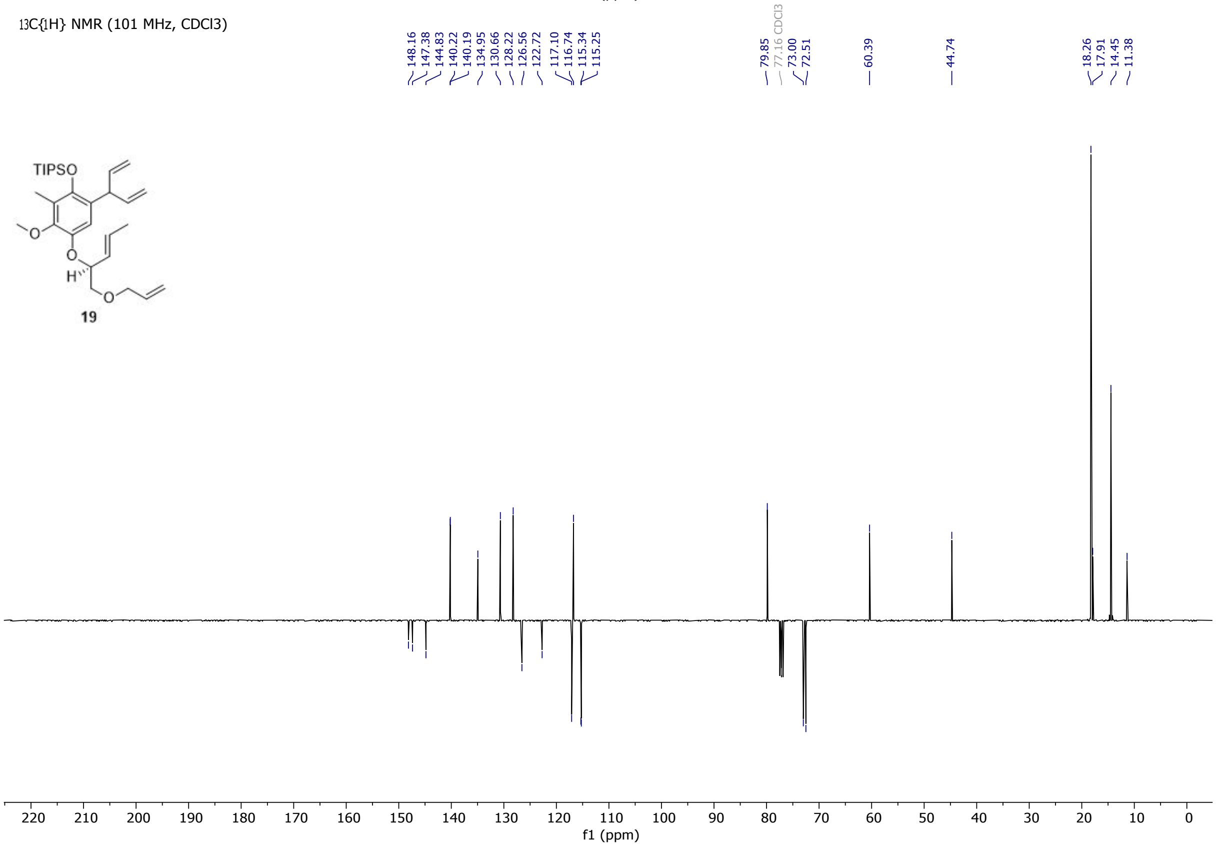

14. (*S*,*E*)-2-(5-(Allyloxy)pent-3-en-2-yl)-6-methoxy-5-methyl-3-(penta-1,4-dien-3-yl)-4-((triisopropylsilyl)oxy)phenol (10)

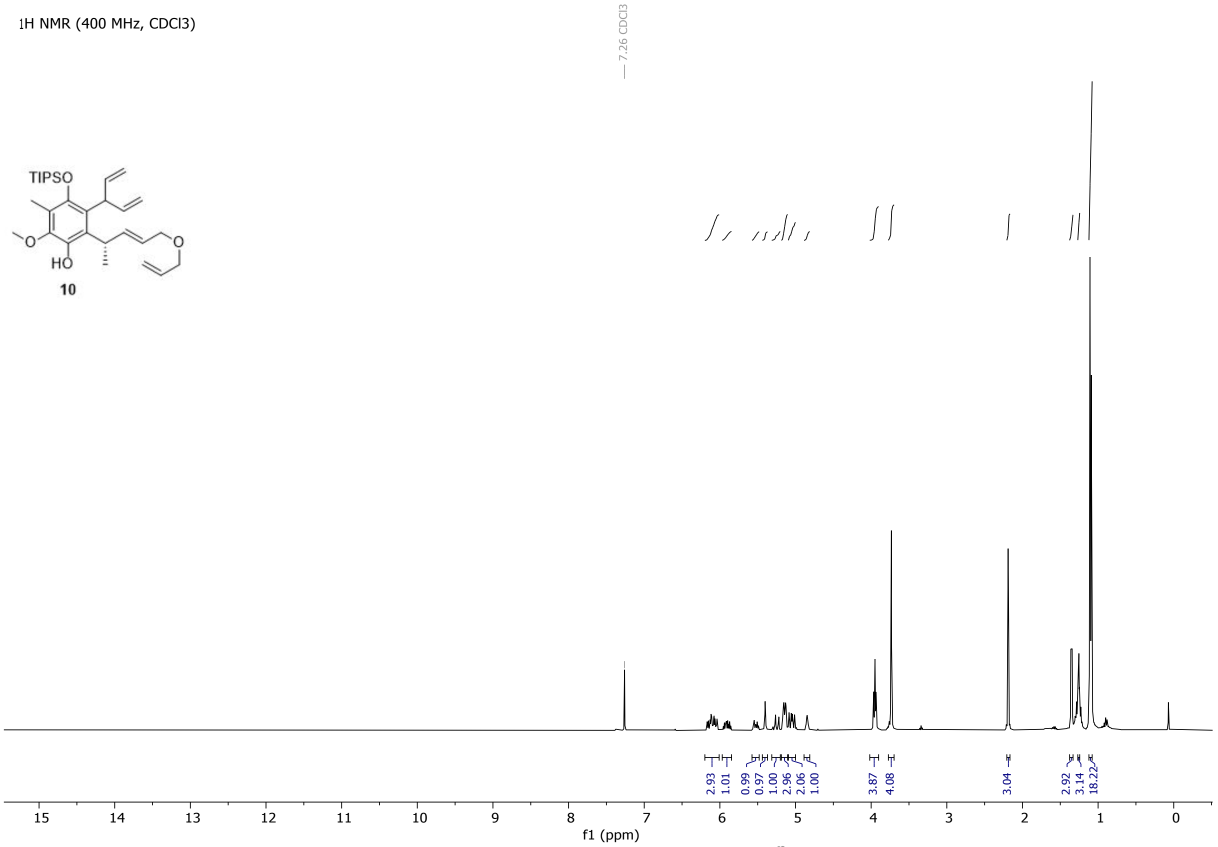

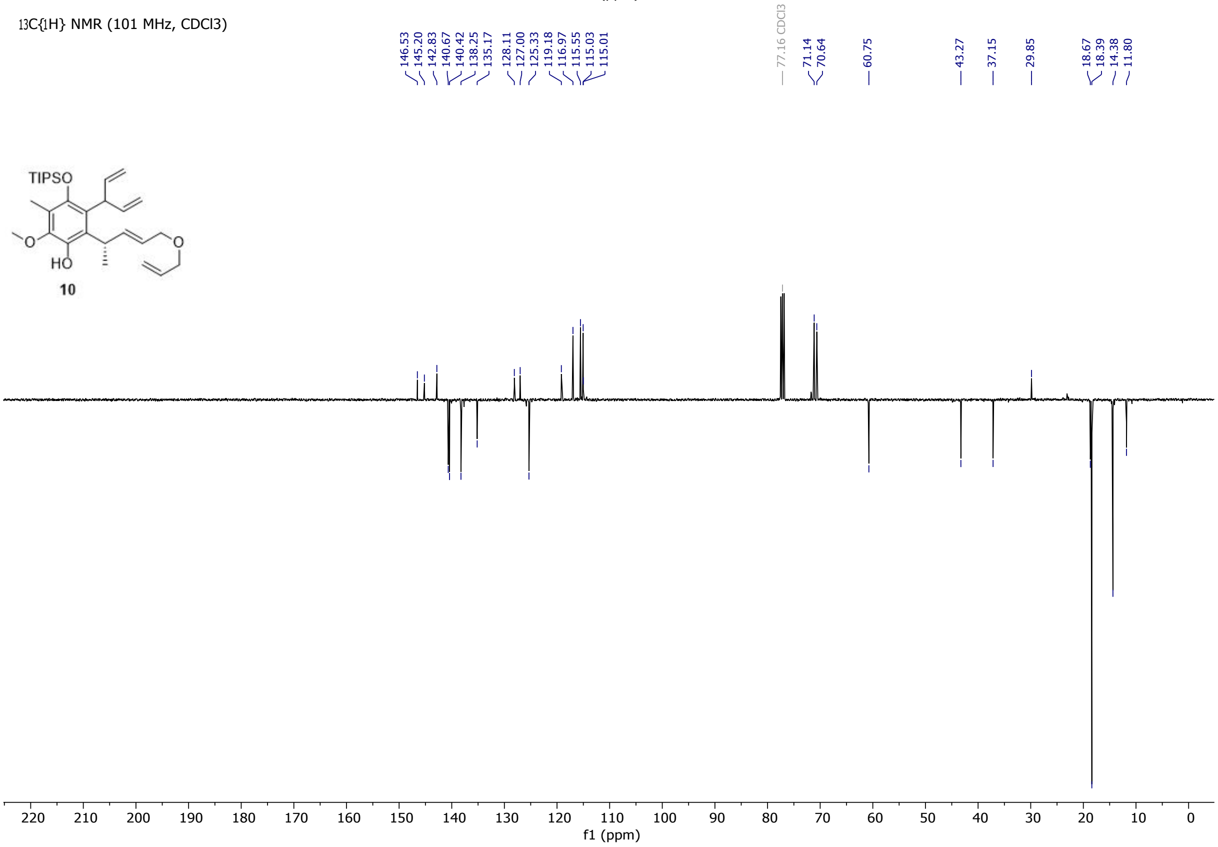

15. (5*S*,8*S*)-2-Methoxy-3,8-dimethyl-4-((triisopropylsilyl)oxy)-5-vinyl-5,8-dihydronaphthalen-1-ol (20)

1H NMR (400 MHz, CDCl3)

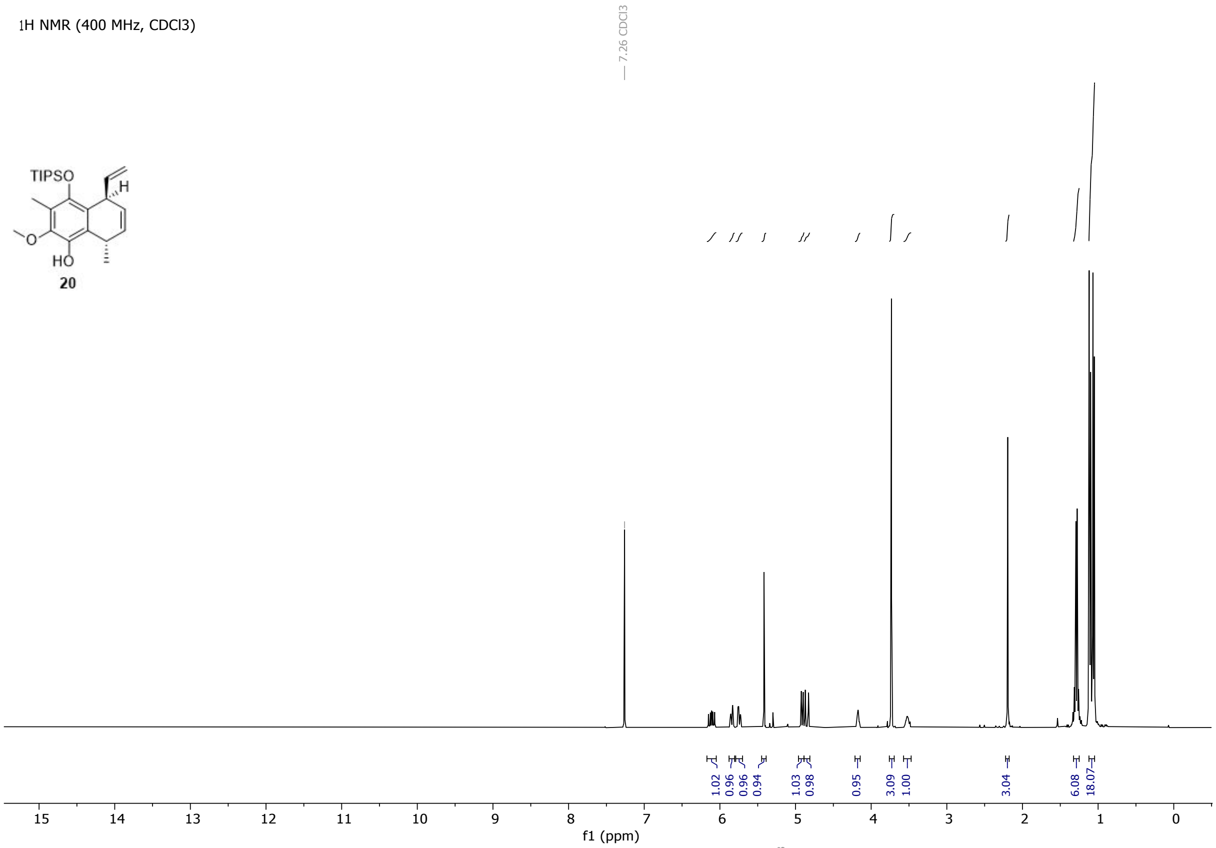

13C{1H} NMR (101 MHz, CDCl3)

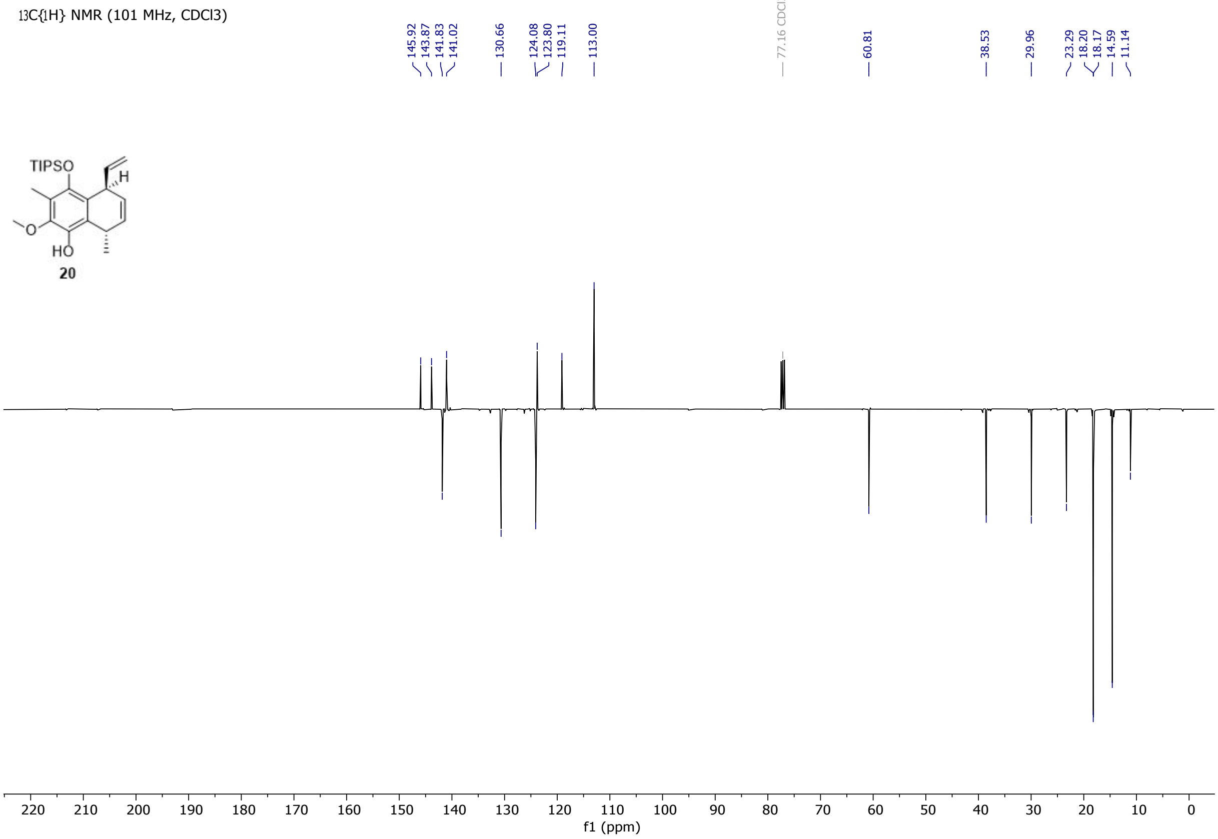

16. (((5*S*,8*S*)-2-Methoxy-3,8-dimethyl-5-vinyl-5,8-dihydronaphthalene-1,4-diyl)bis(oxy))bis(triisopropylsilane) (9)

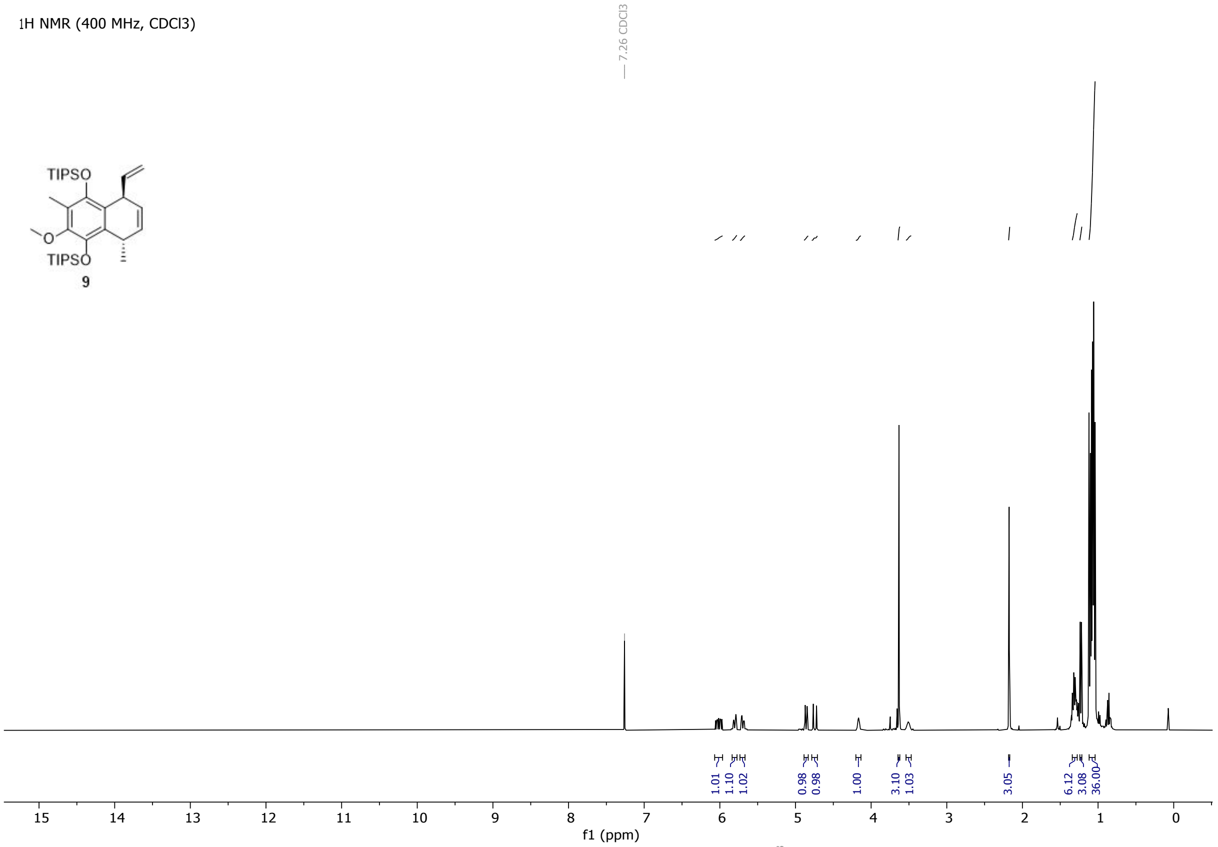

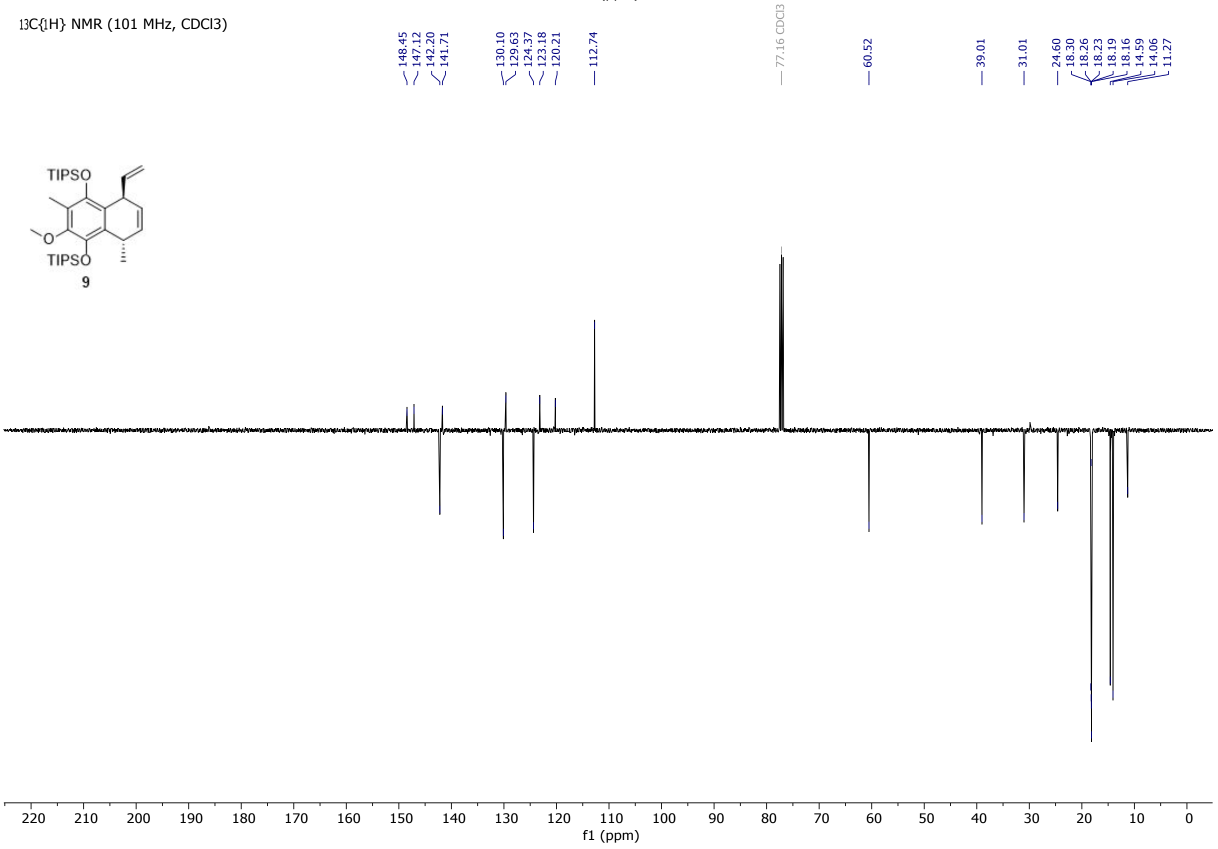

17. Methyl (*E*)-3-((1*R*,4*S*)-6-methoxy-4,7-dimethyl-5,8-bis((triisopropylsilyl)oxy)-1,4-dihydronaphthalen-1-yl)acrylate (21)

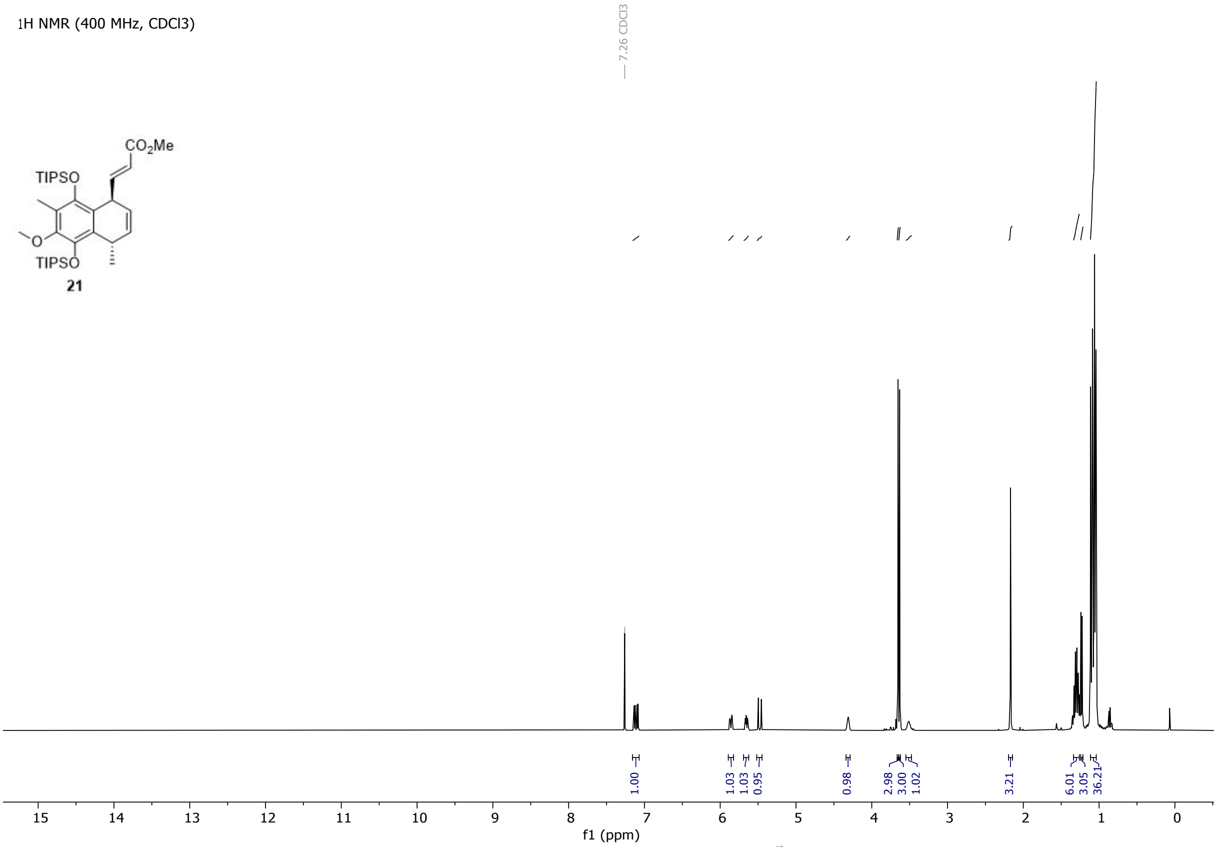

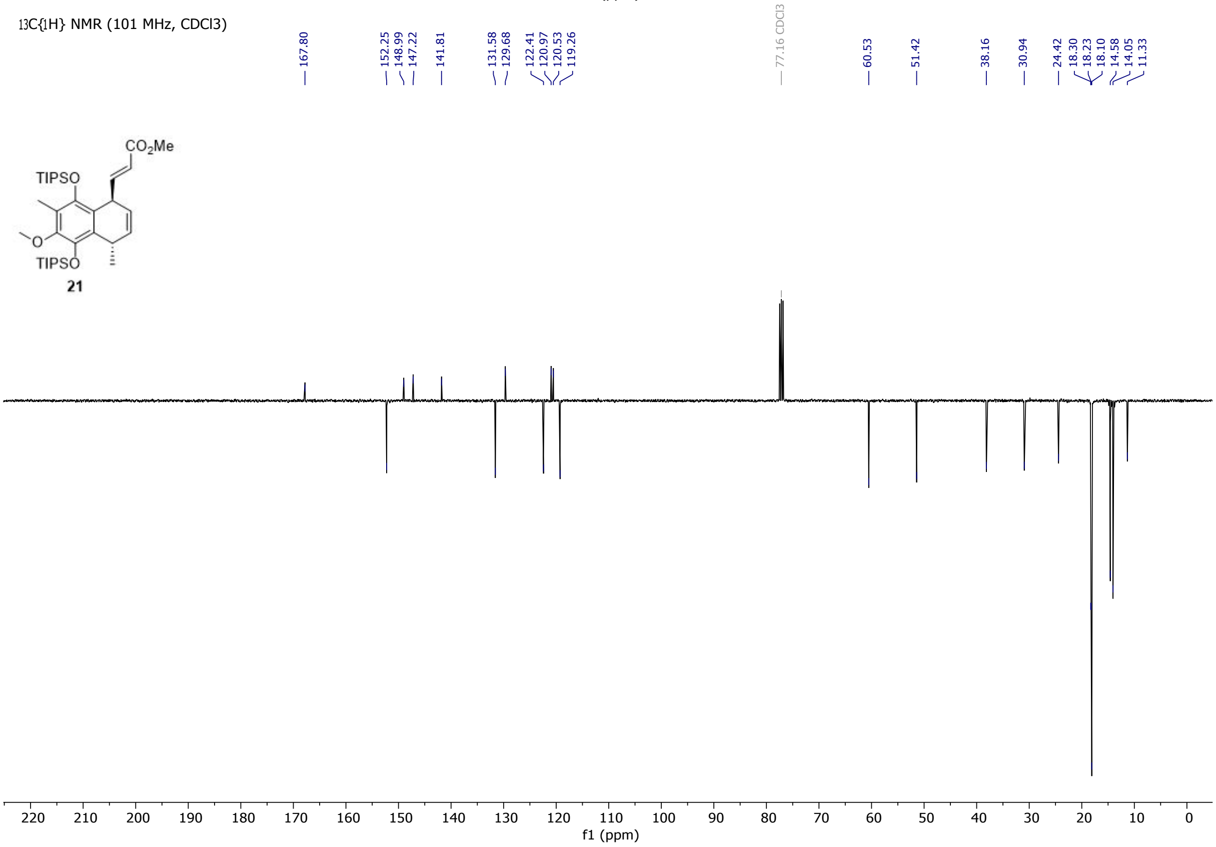

18. Methyl (*R*)-3-((1S,4S)-6-methoxy-4,7-dimethyl-5,8-bis((triisopropylsilyl)oxy)-1,4-dihydronaphthalen-1-yl)butanoate (7-epi-8)

1H NMR (400 MHz, CDCl3)

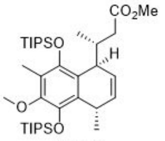

7-epi-8

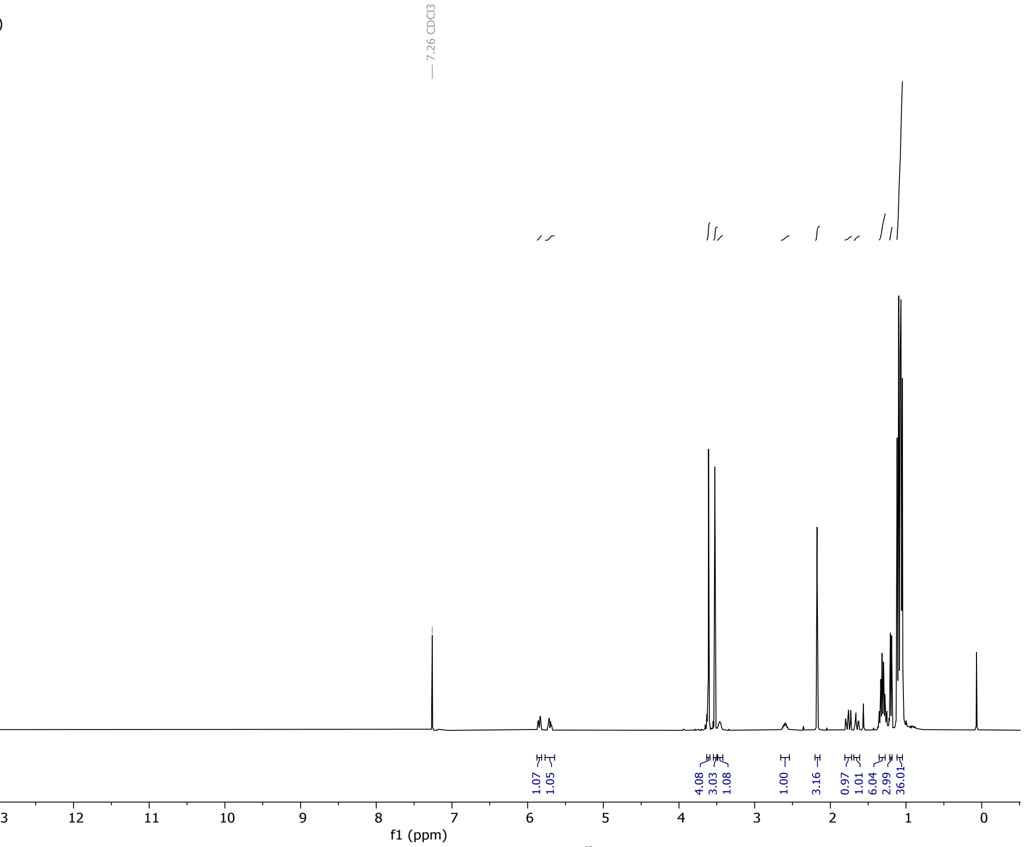

13C{1H} NMR (101 MHz, CDCl3)

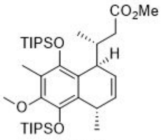

7-epi-8

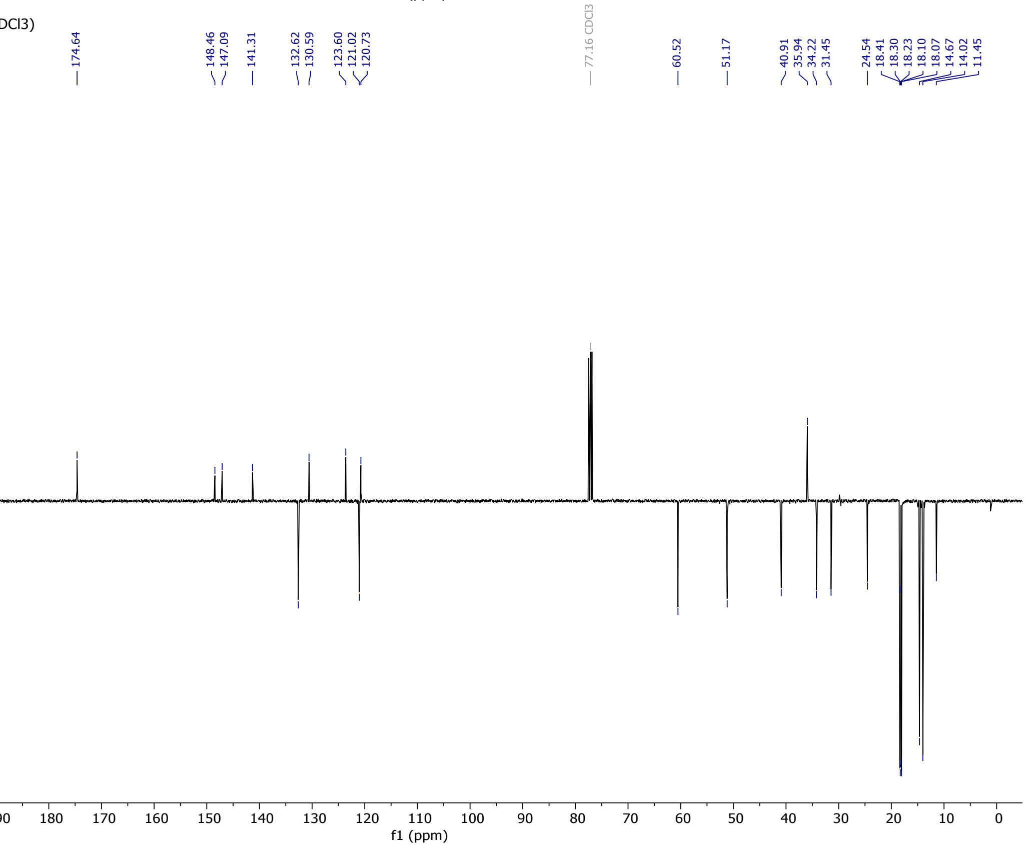

19. 1-((1*S*,4*S*)-6-Methoxy-4,7-dimethyl-5,8-bis((triisopropylsilyl)oxy)-1,4-dihydronaphthalen-1-yl)ethan-1-one (23)

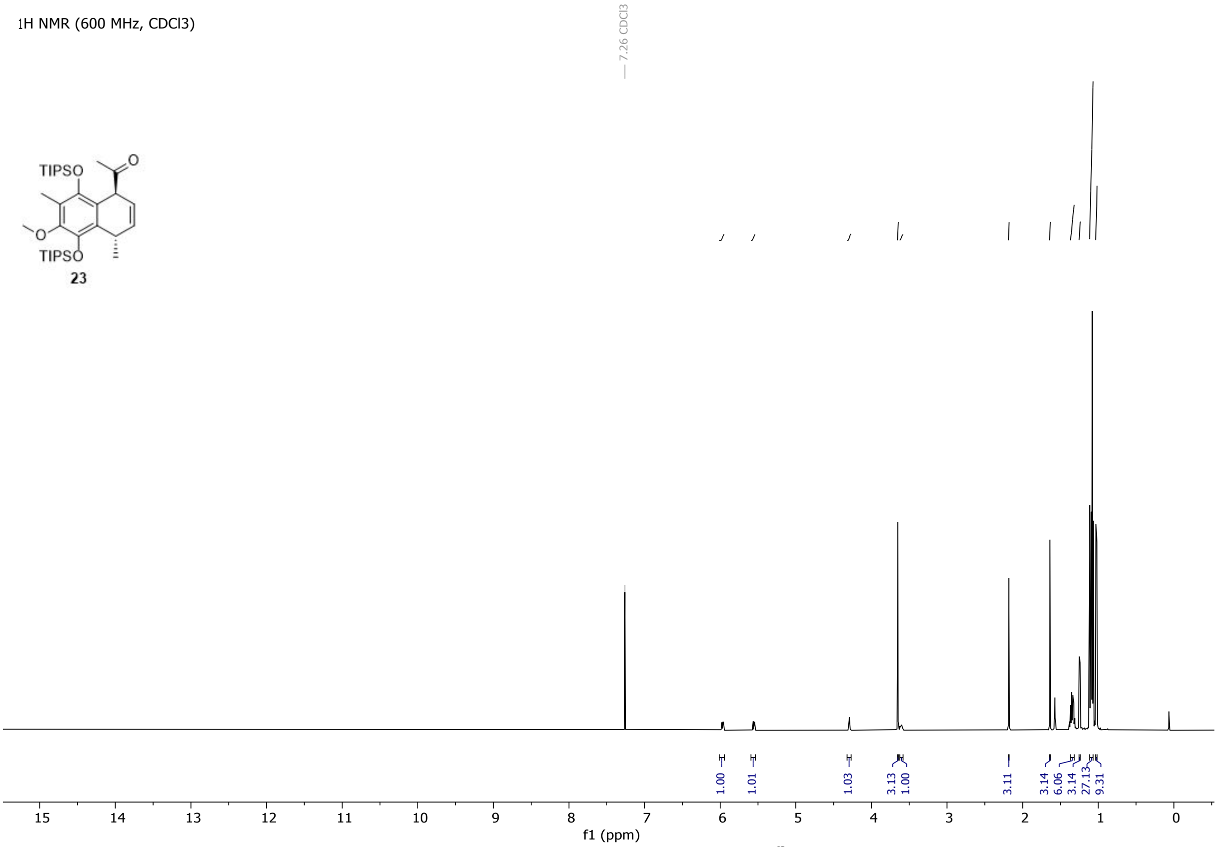

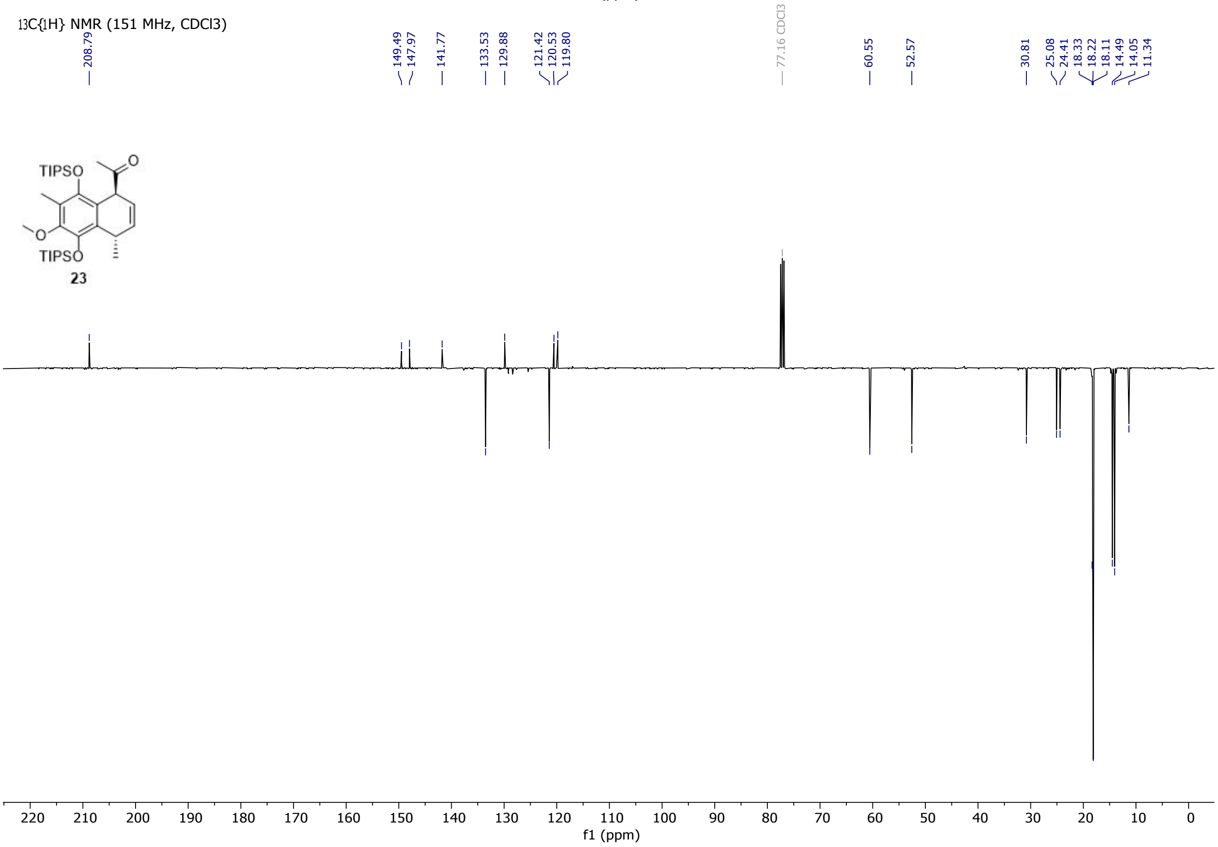

20. 1-((1*S*,4*S*)-6-Methoxy-4,7-dimethyl-5,8-bis((triisopropylsilyl)oxy)-1,2,3,4-tetrahydronaphthalen-1-yl)ethan-1-one (24)

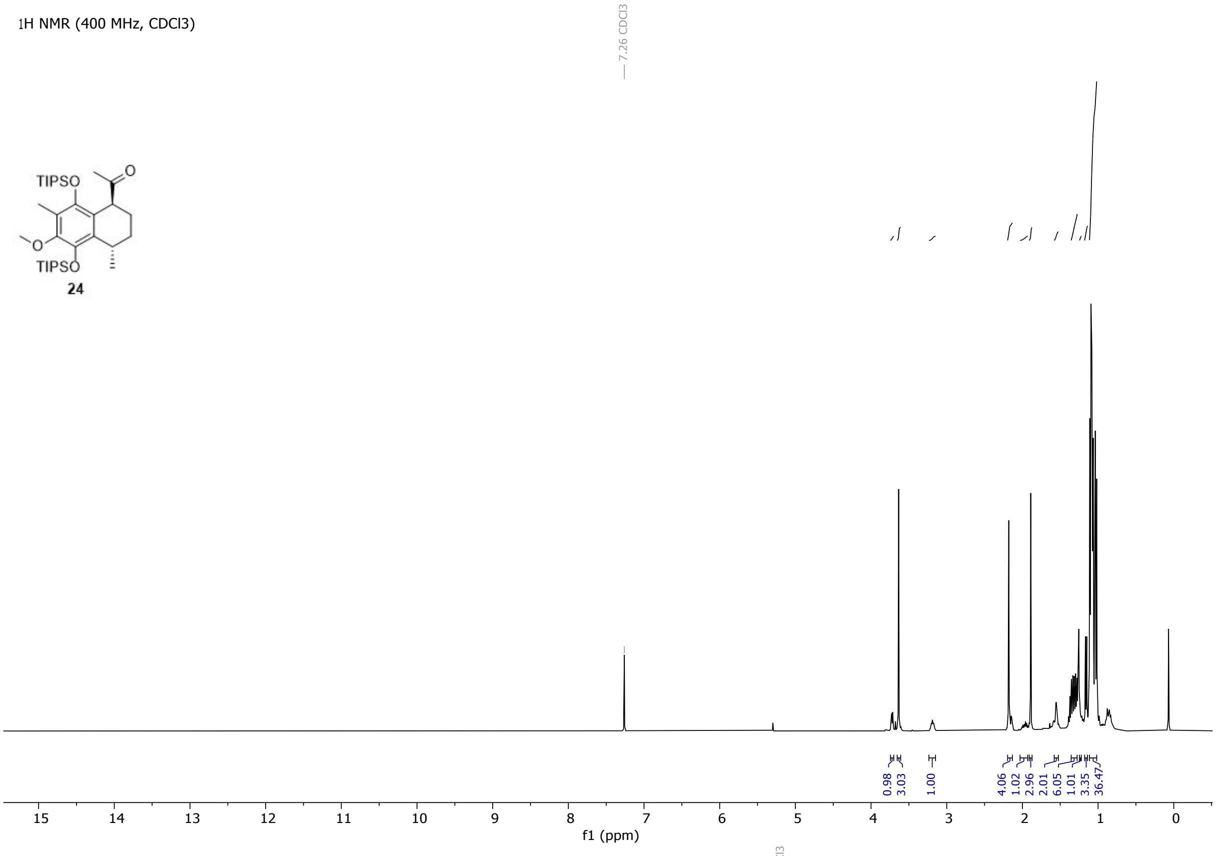

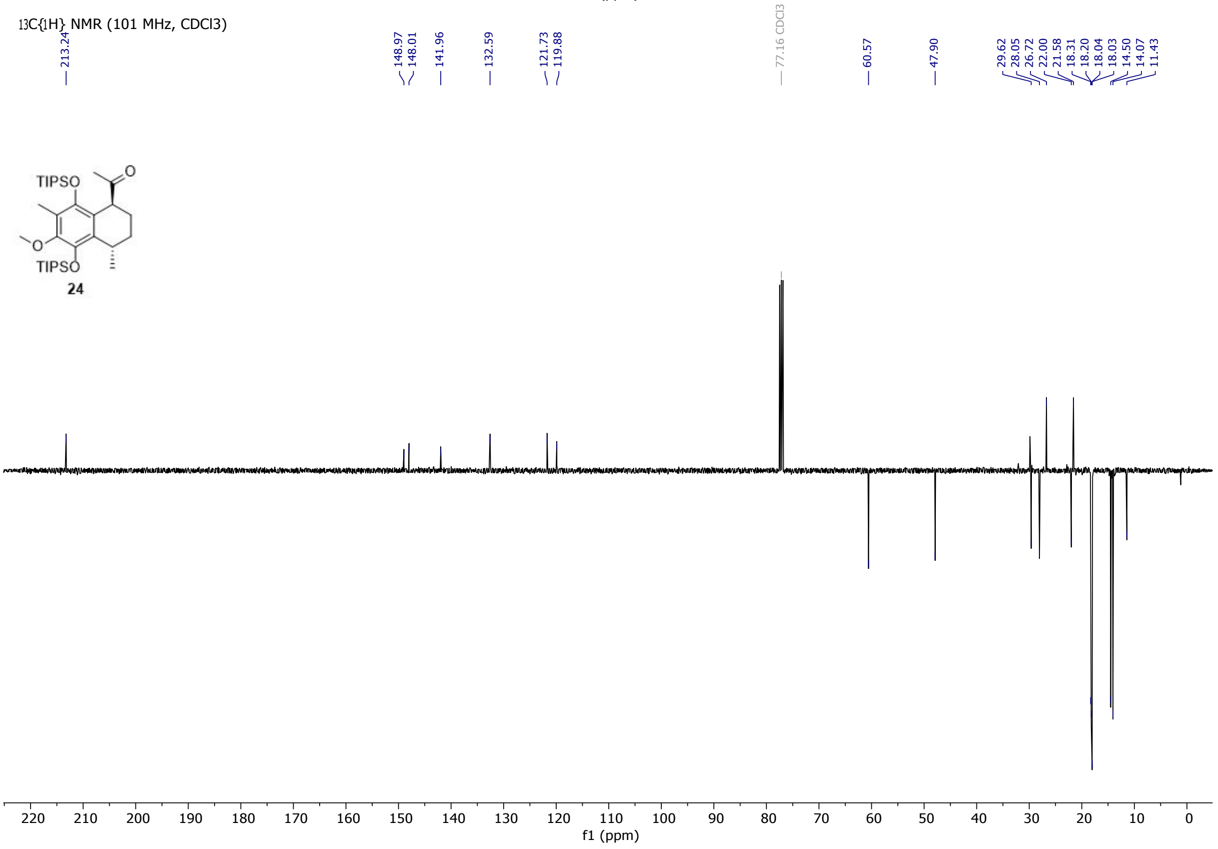

21. Methyl (*Z*)-3-((1*R*,4*S*)-6-methoxy-4,7-dimethyl-5,8-bis((triisopropylsilyl)oxy)-1,4-dihydronaphthalen-1-yl)but-2-enoate (*Z*-22)

^{1}H NMR (600 MHz, CDCl3)

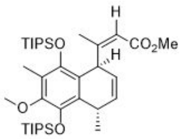

(*Z*)-22

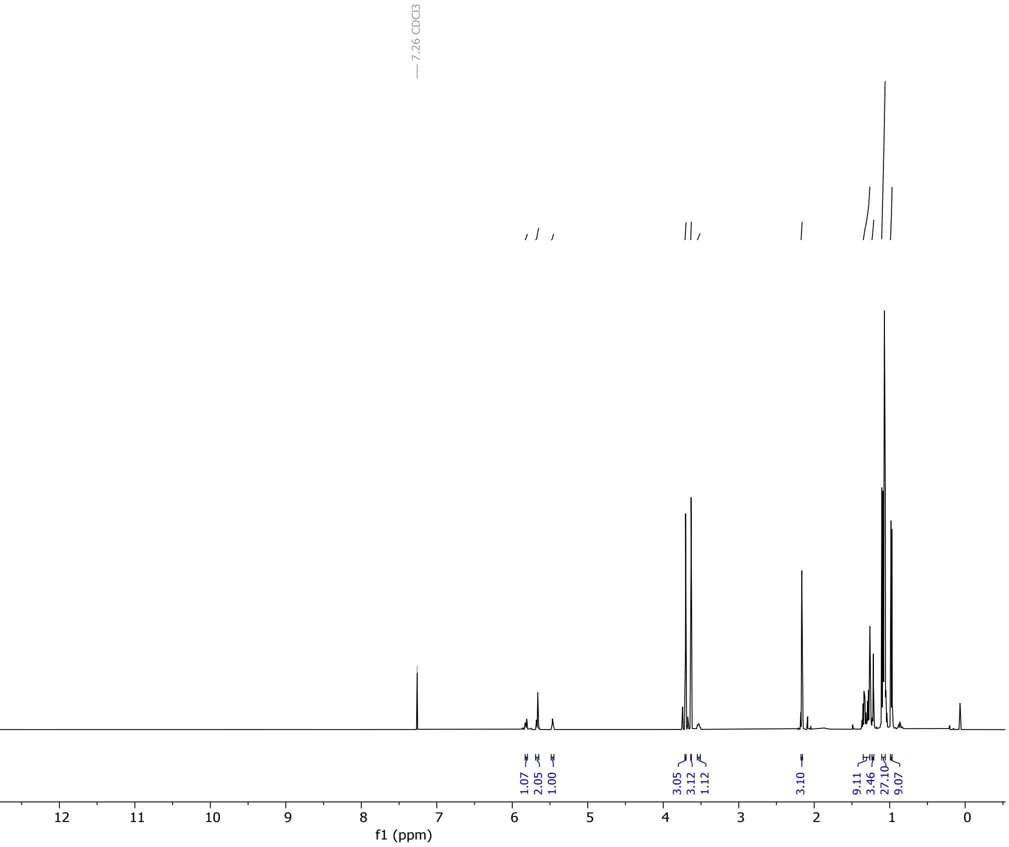

^{13}C{^{1}H} NMR (151 MHz, CDCl3)

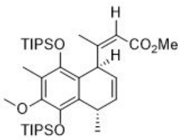

(*Z*)-22

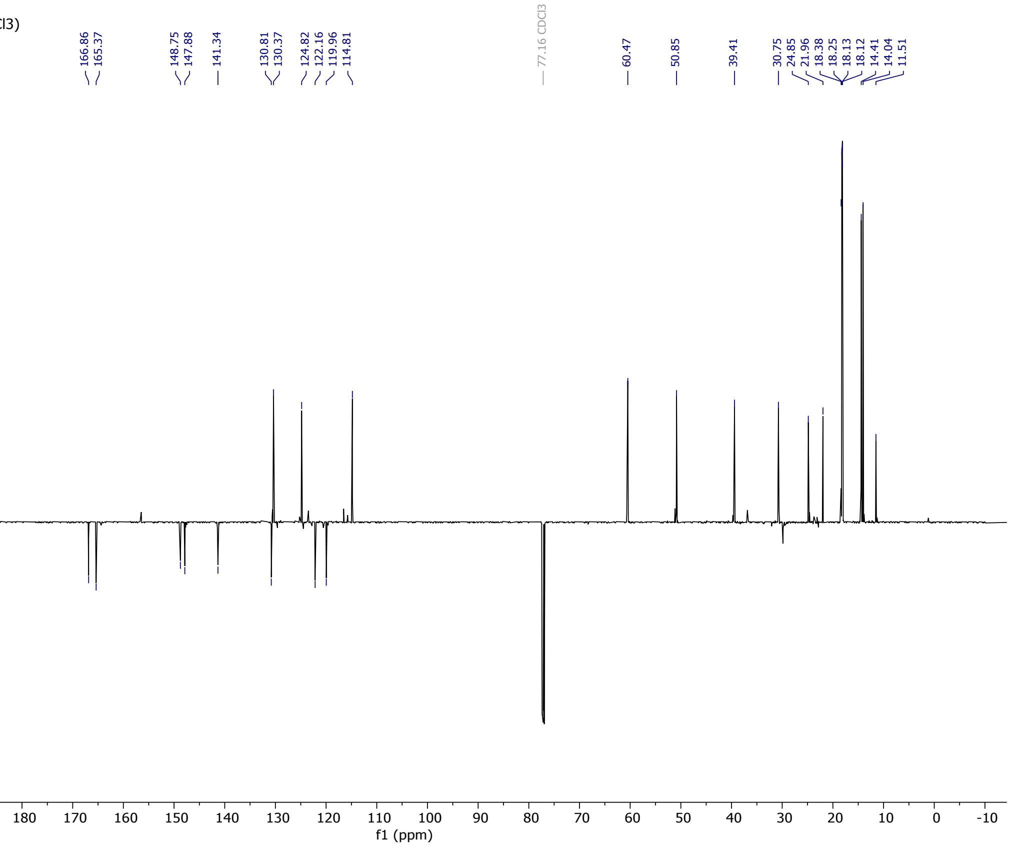

22. Methyl (*E*)-3-((1*R*,4*S*)-6-methoxy-4,7-dimethyl-5,8-bis((triisopropylsilyl)oxy)-1,4-dihydronaphthalen-1-yl)but-2-enoate (*E*-22)

1H NMR (600 MHz, CDCl3)

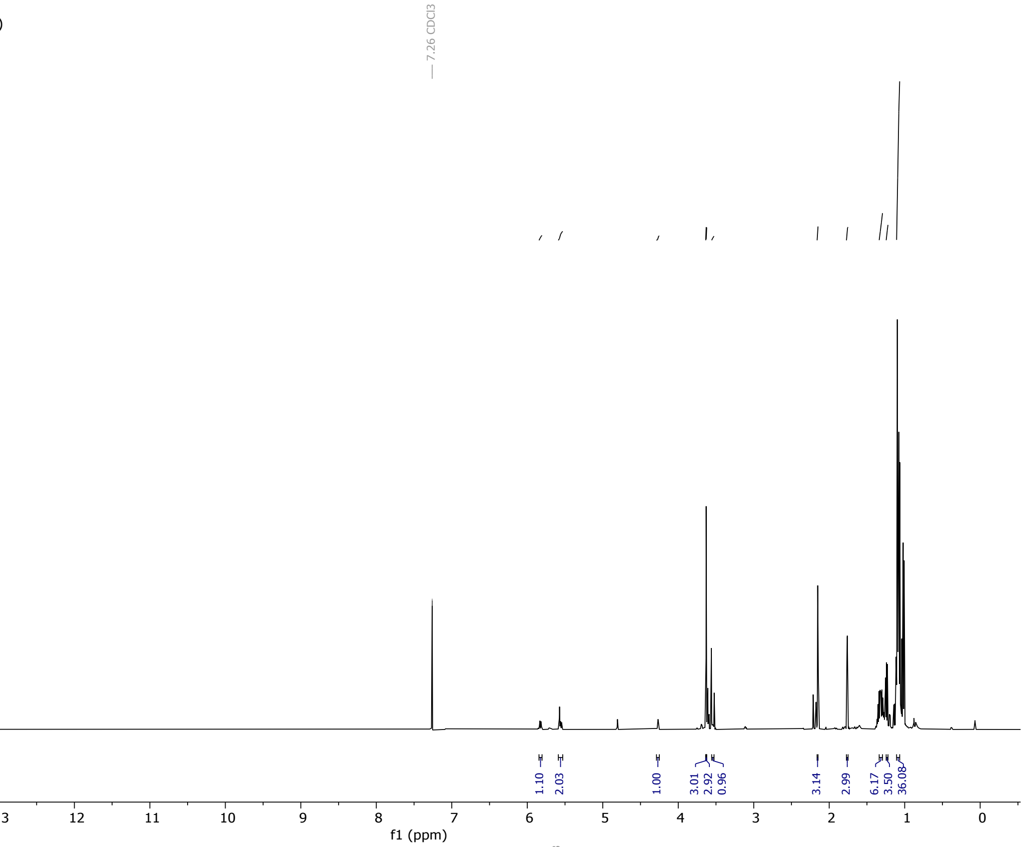

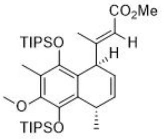

(*E*)-22

13C{1H} NMR (151 MHz, CDCl3)

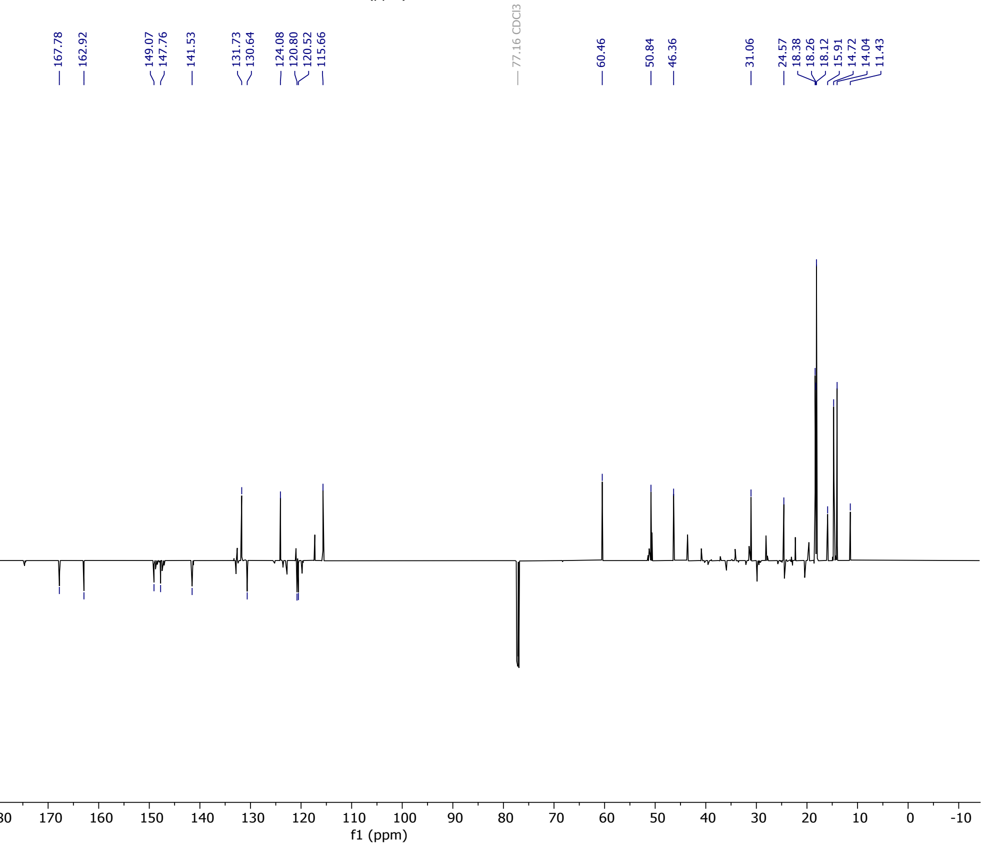

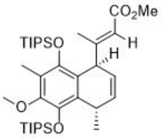

(*E*)-22

23. (5*S*,8*S*)-3-Methoxy-2,5-dimethyl-4-((triisopropylsilyl)oxy)-8-vinyl-5,8-dihydronaphthalen-1-ol (25)

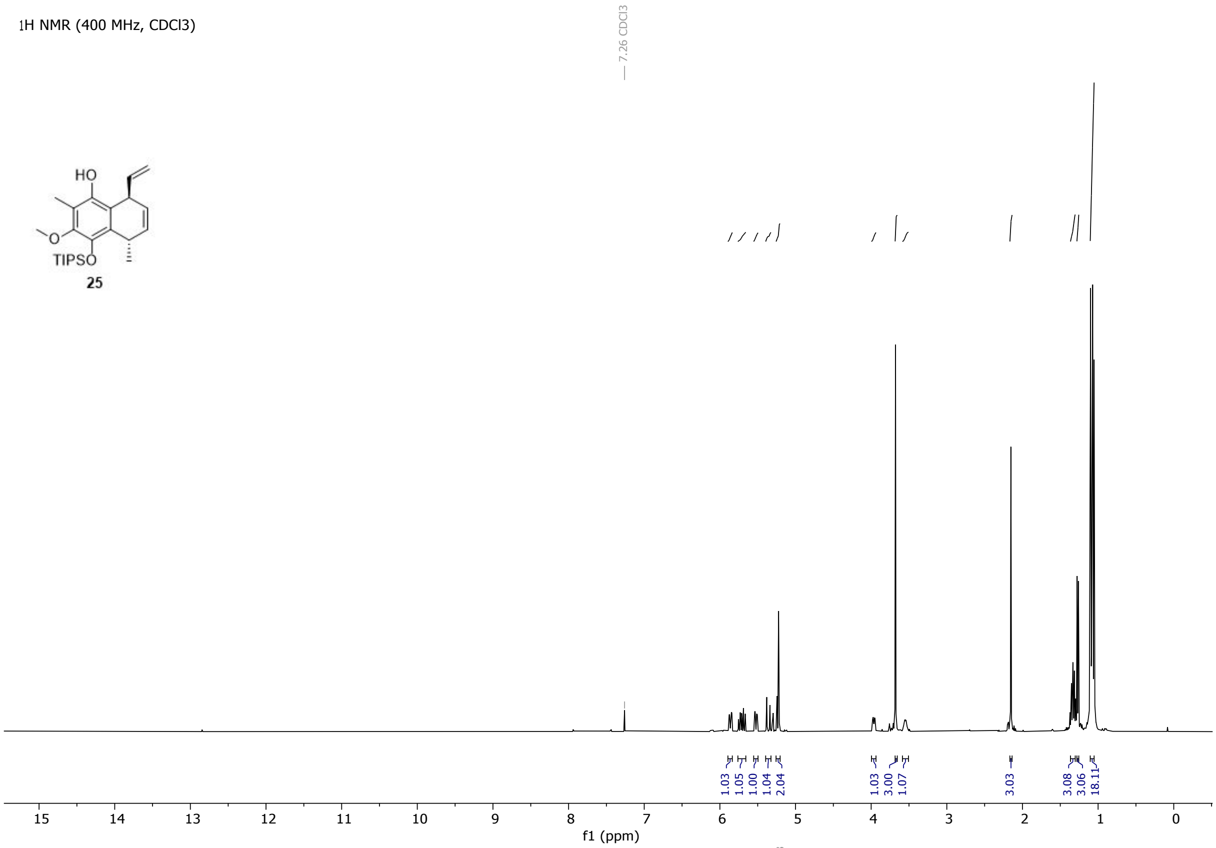

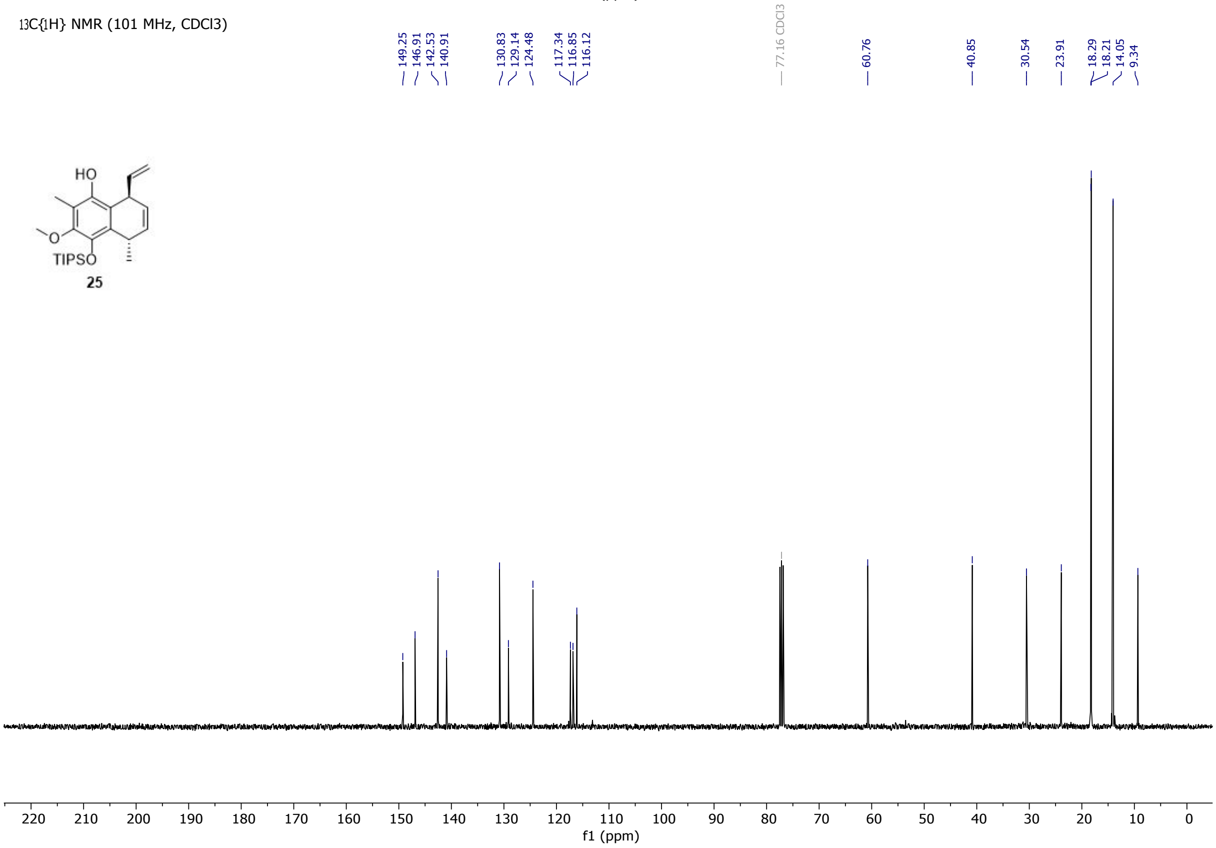

24. (5*S*,8*S*)-3-Methoxy-2,5-dimethyl-4-((triisopropylsilyl)oxy)-8-vinyl-5,8-dihydronaphthalen-1-yl acrylate (26)

^1H NMR (600 MHz, CDCl3)

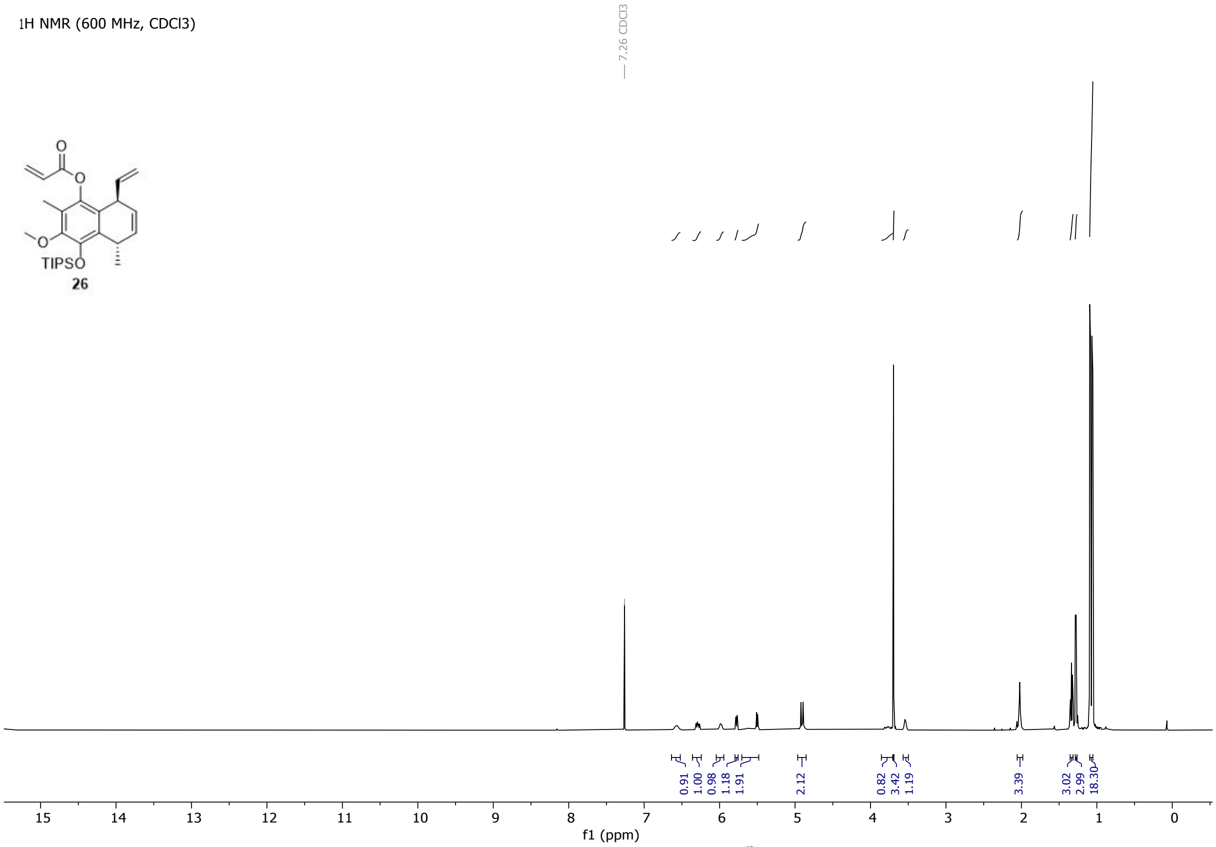

$^{13}C\{^1H\}$ NMR (151 MHz, CDCl3)

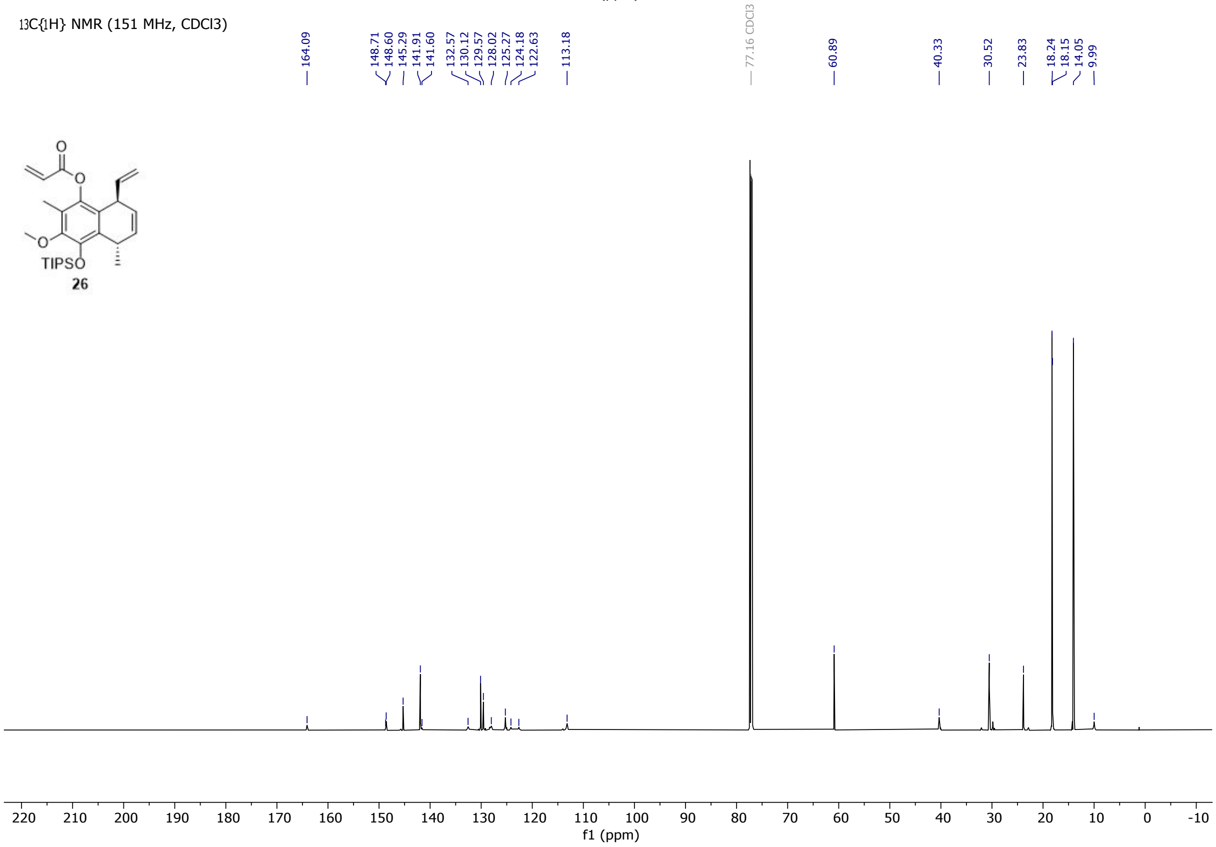

25. (4a*R*,7*S*)-9-Methoxy-7,10-dimethyl-8-((triisopropylsilyl)oxy)-4a,7-dihydro-2H-naphtho[1,8-bc]oxepin-2-one (27)

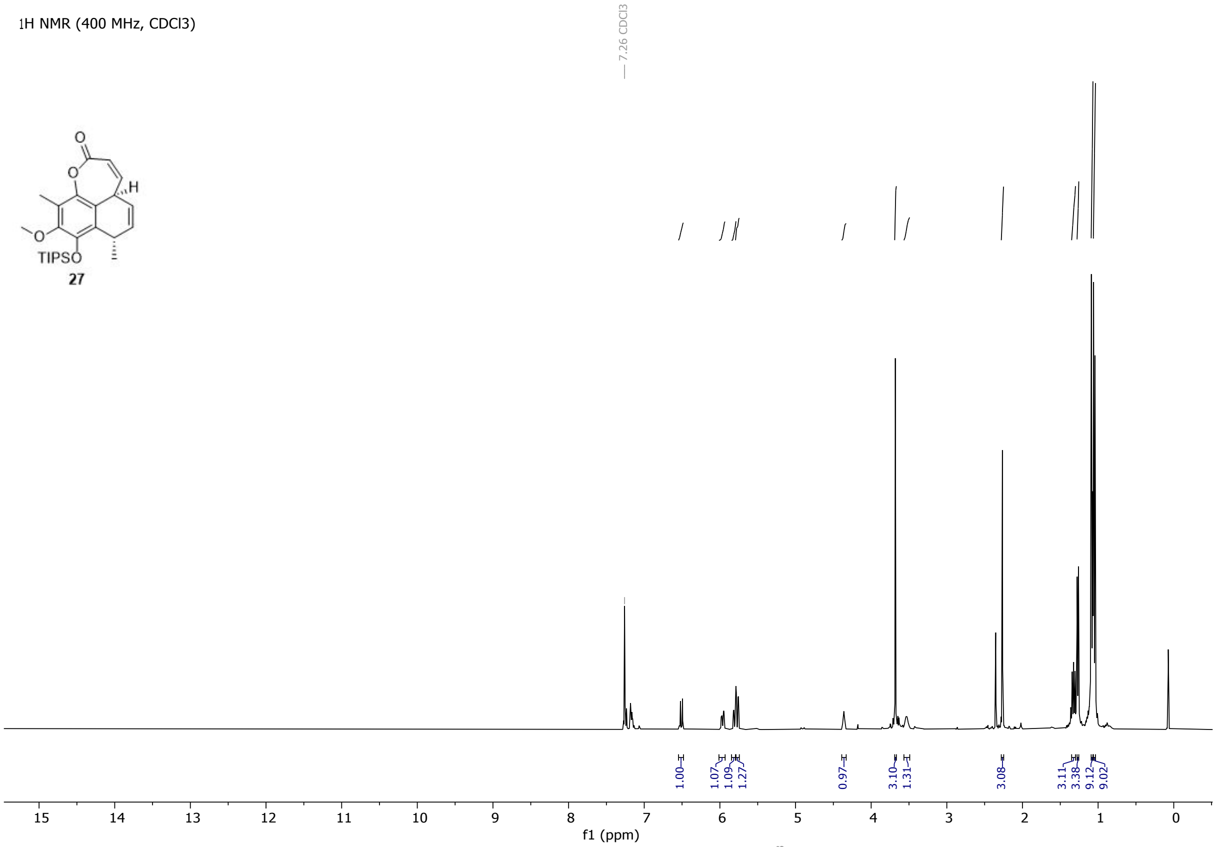

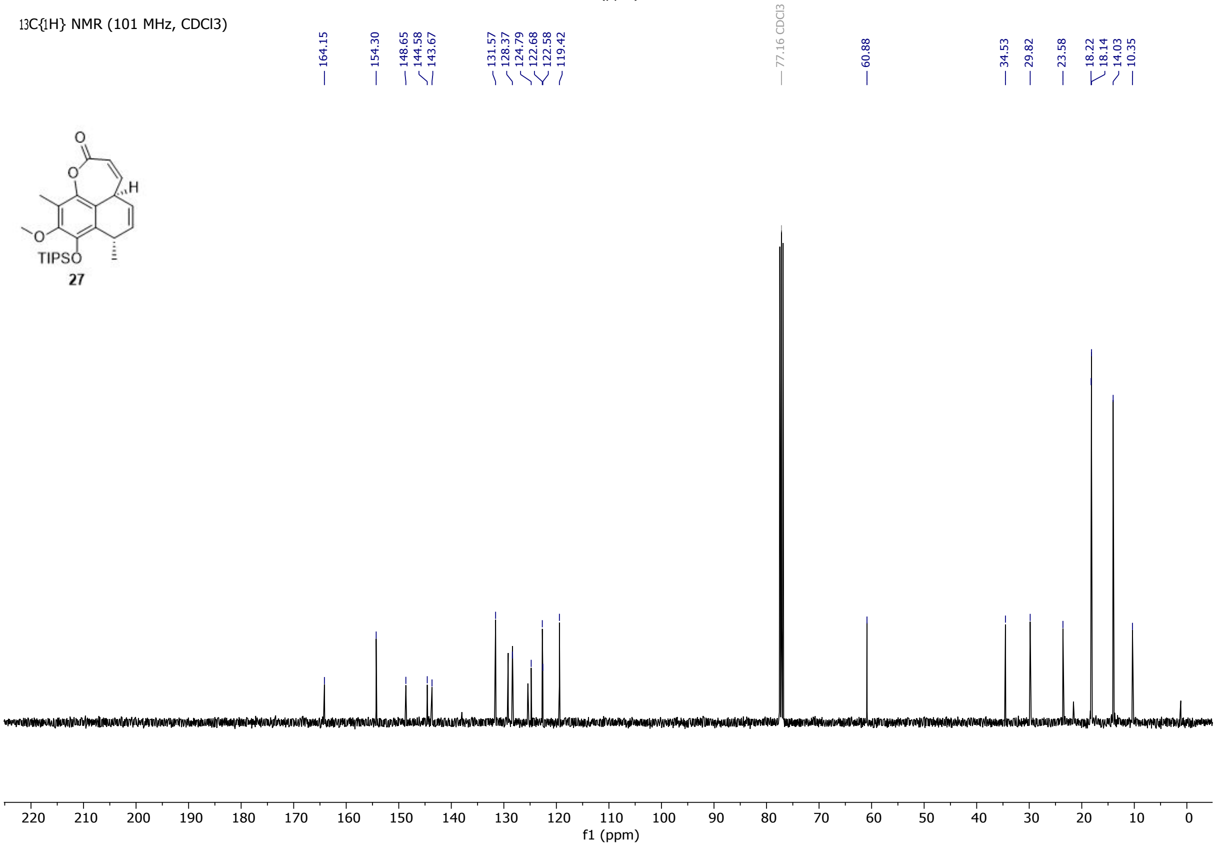

26. 3-Methoxy-2-methyl-6-(penta-1,4-dien-3-yl)-4-((triisopropylsilyl)oxy)phenol (34)

1H NMR (400 MHz, CDCl3)

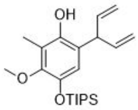

34

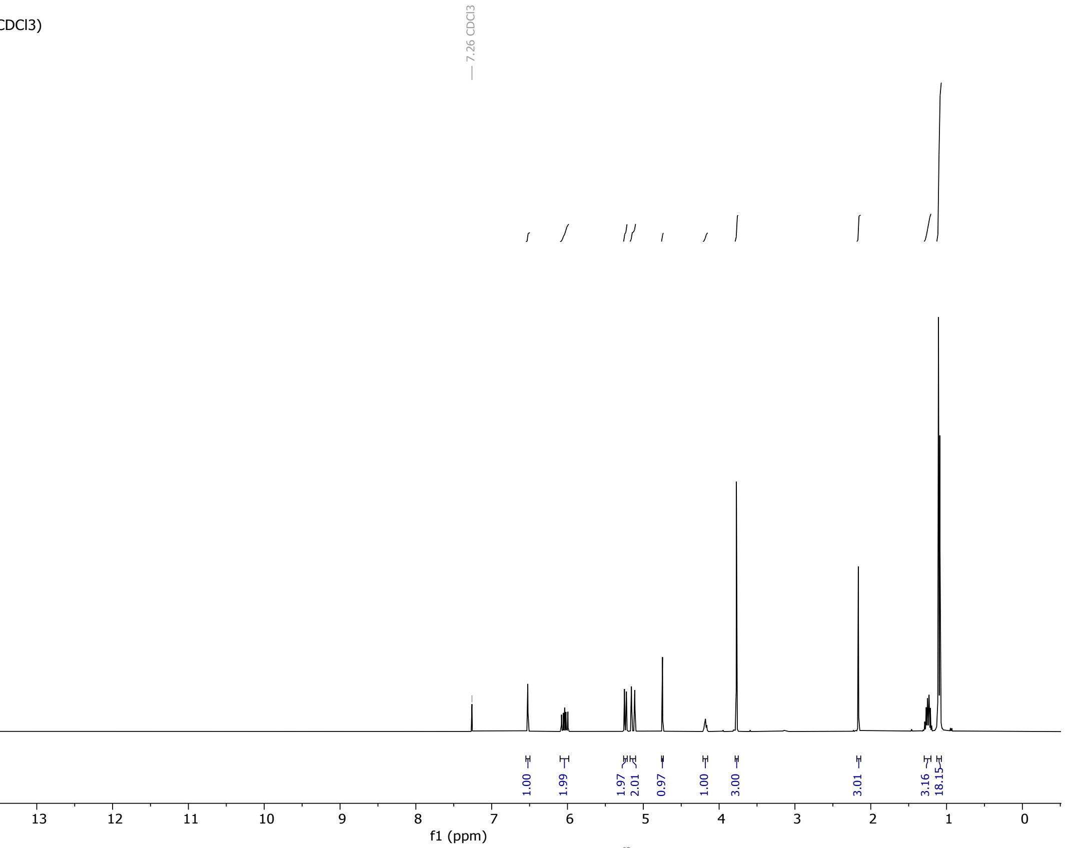

13C{1H} NMR (101 MHz, CDCl3)

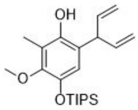

34

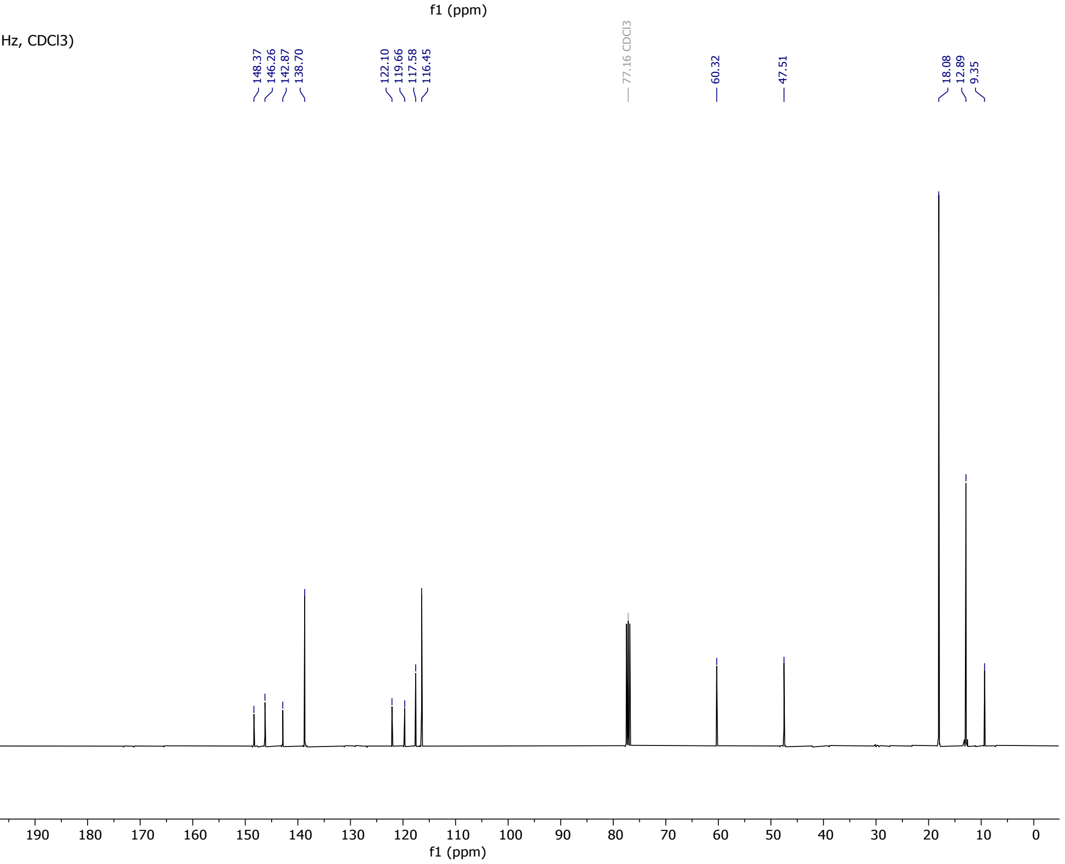

27. 3-Methoxy-2-methyl-6-(penta-1,4-dien-3-yl)-4-((triisopropylsilyl)oxy)phenyl acrylate (33)

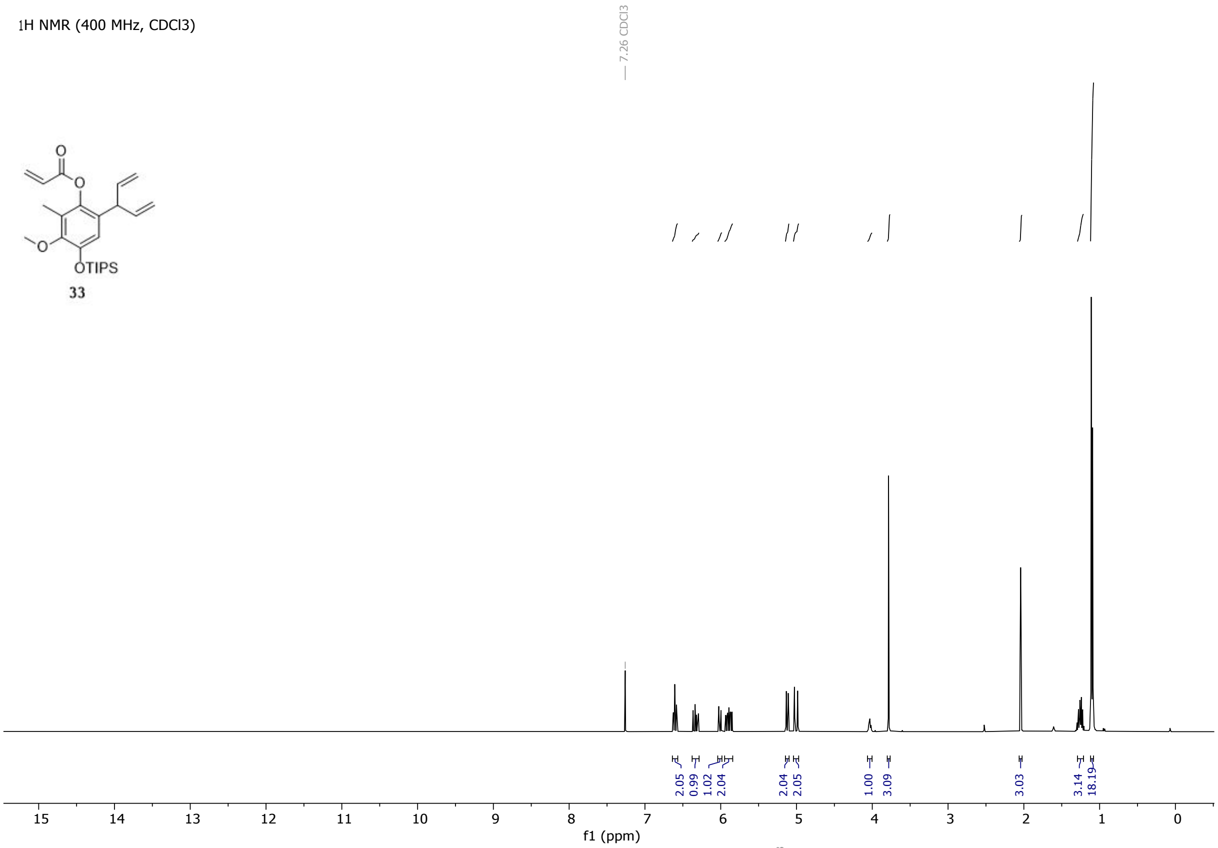

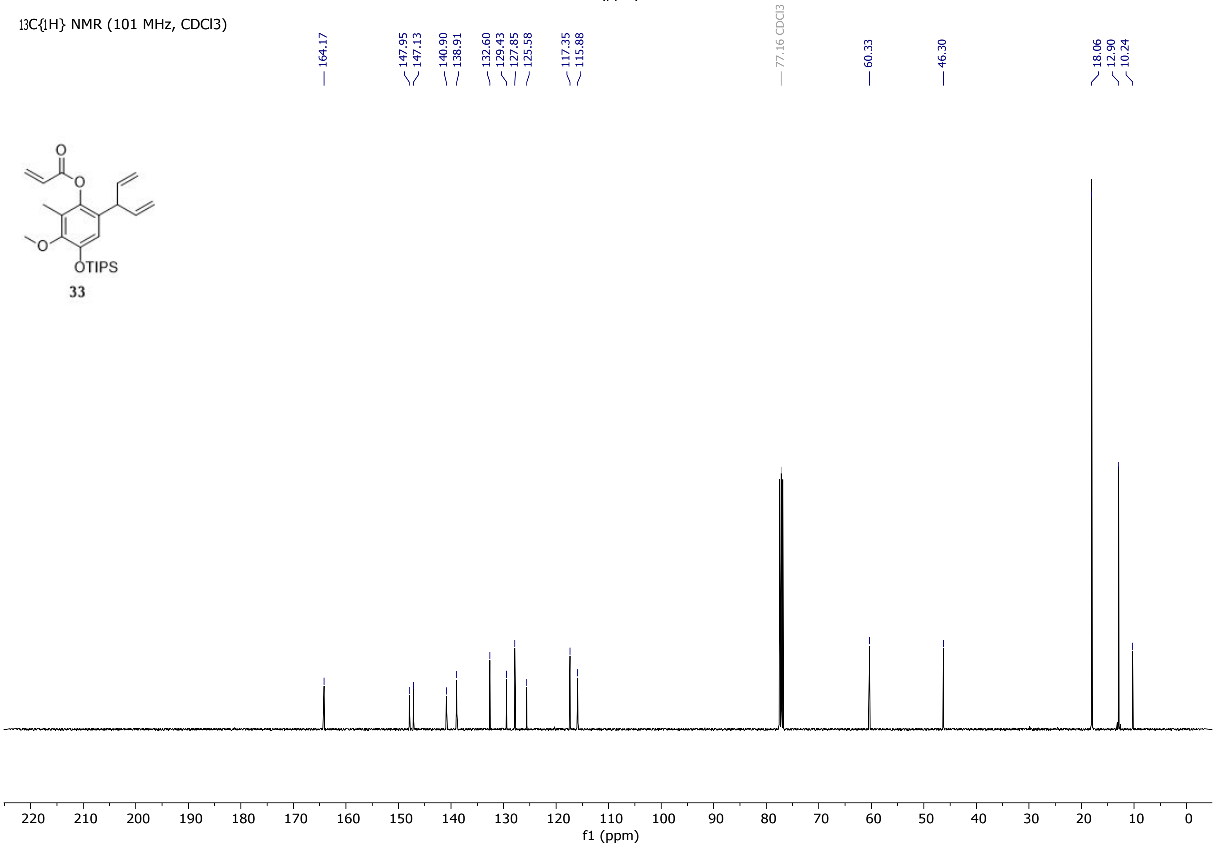

28. 8-Methoxy-9-methyl-7-((triisopropylsilyl)oxy)-5-vinylbenzo[b]oxepin-2(5H)-one (rac-35)

1H NMR (400 MHz, CDCl3)

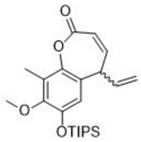

(rac)-35

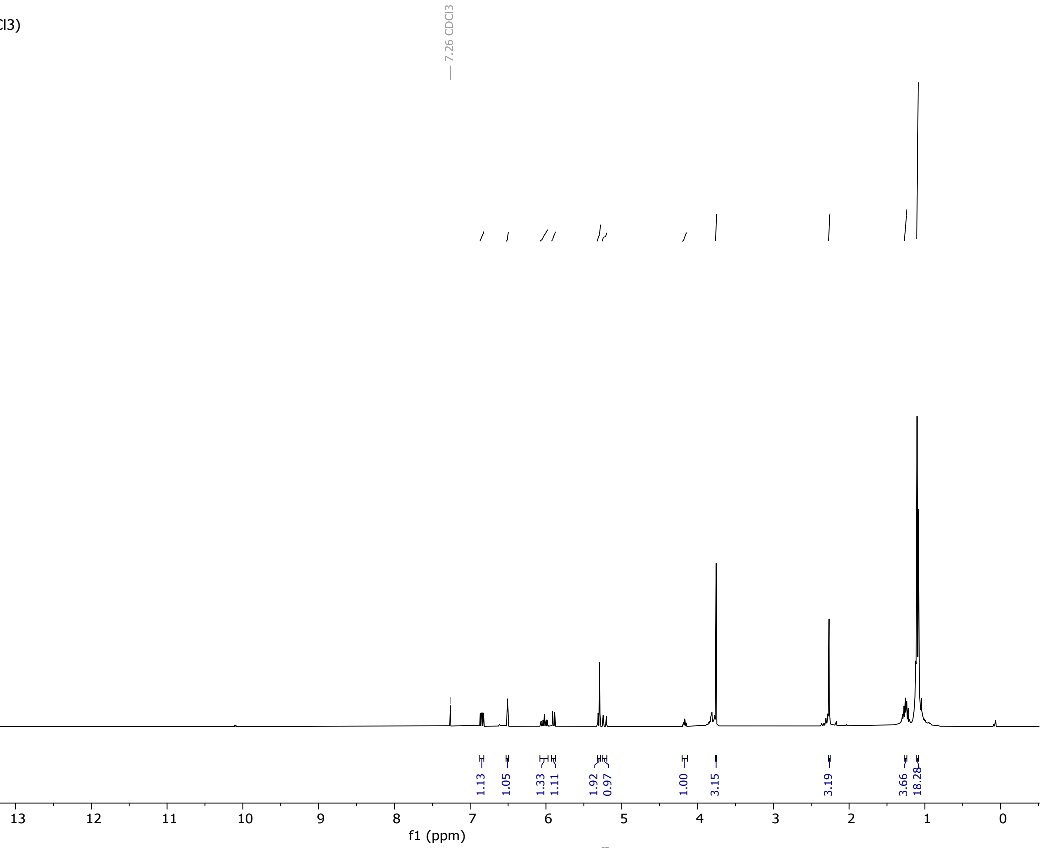

13C{1H} NMR (101 MHz, CDCl3)

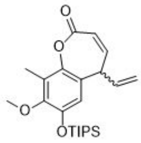

(rac)-35

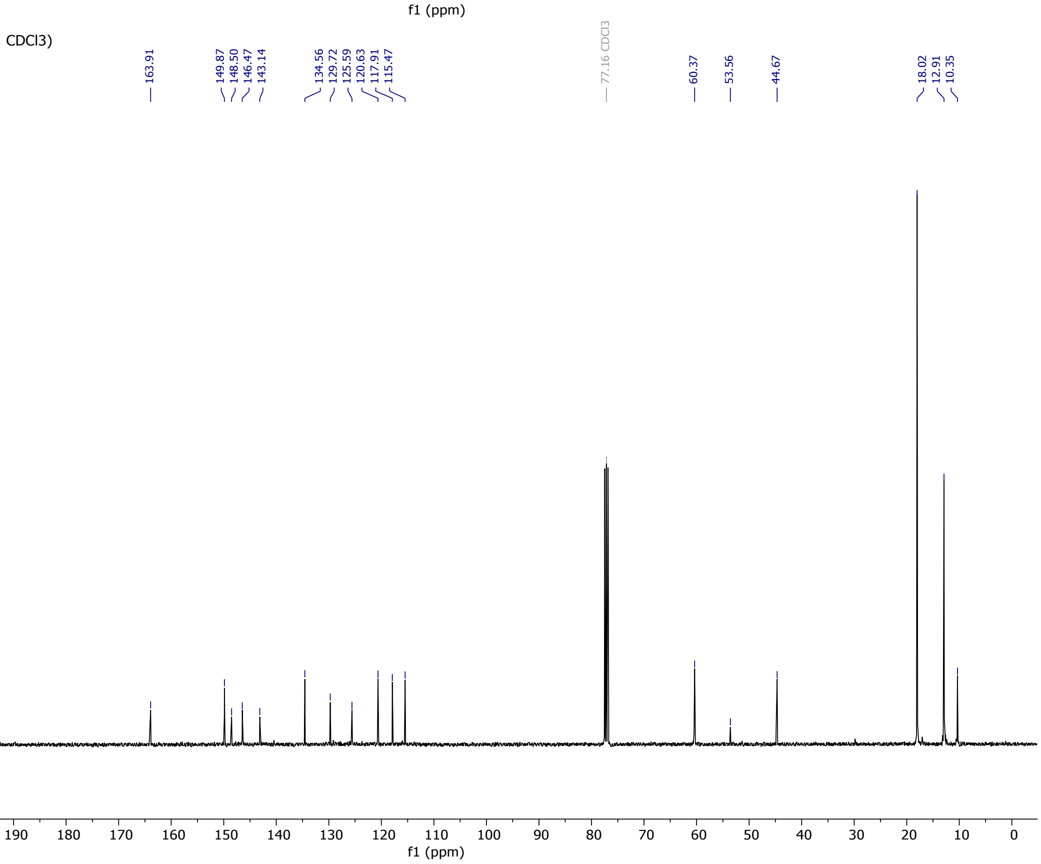

29. 8-Methoxy-4,9-dimethyl-7-((triisopropylsilyl)oxy)-5-vinyl-4,5-dihydrobenzo[b]oxepin-2(3H)-one (rac-36)

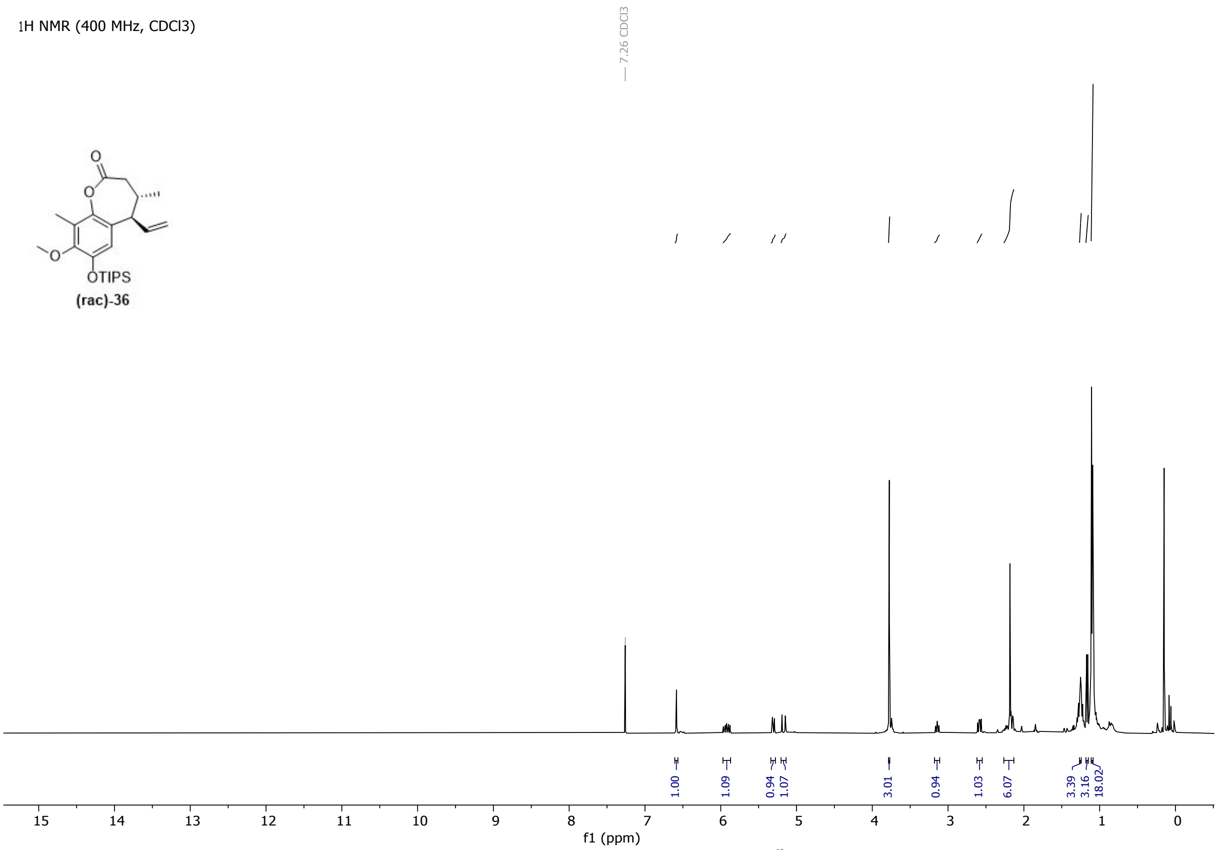

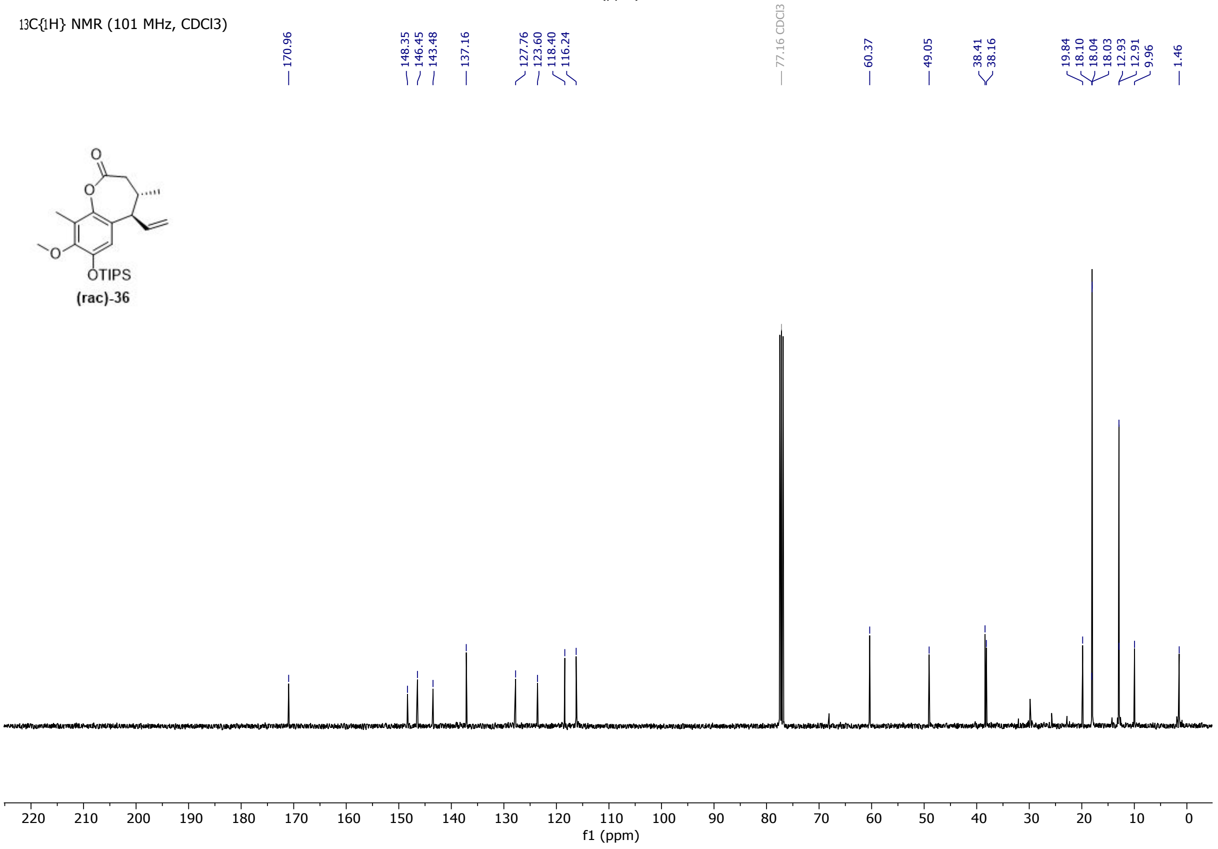

30. 8-Hydroxy-4,9-dimethyl-7-((triisopropylsilyl)oxy)-5-vinyl-4,5-dihydrobenzo[b]oxepin-2(3H)-one (rac-S6)

1H NMR (400 MHz, CDCl3)

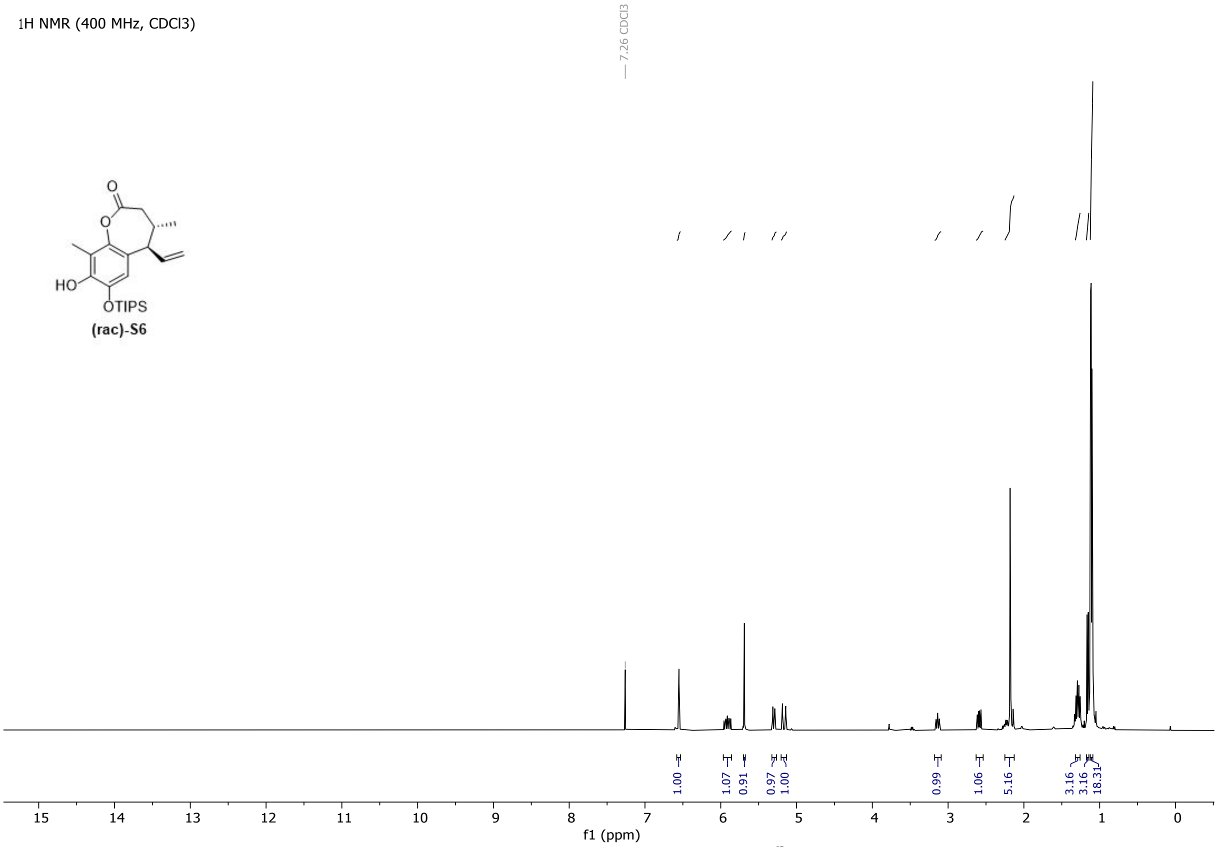

13C{1H} NMR (101 MHz, CDCl3)

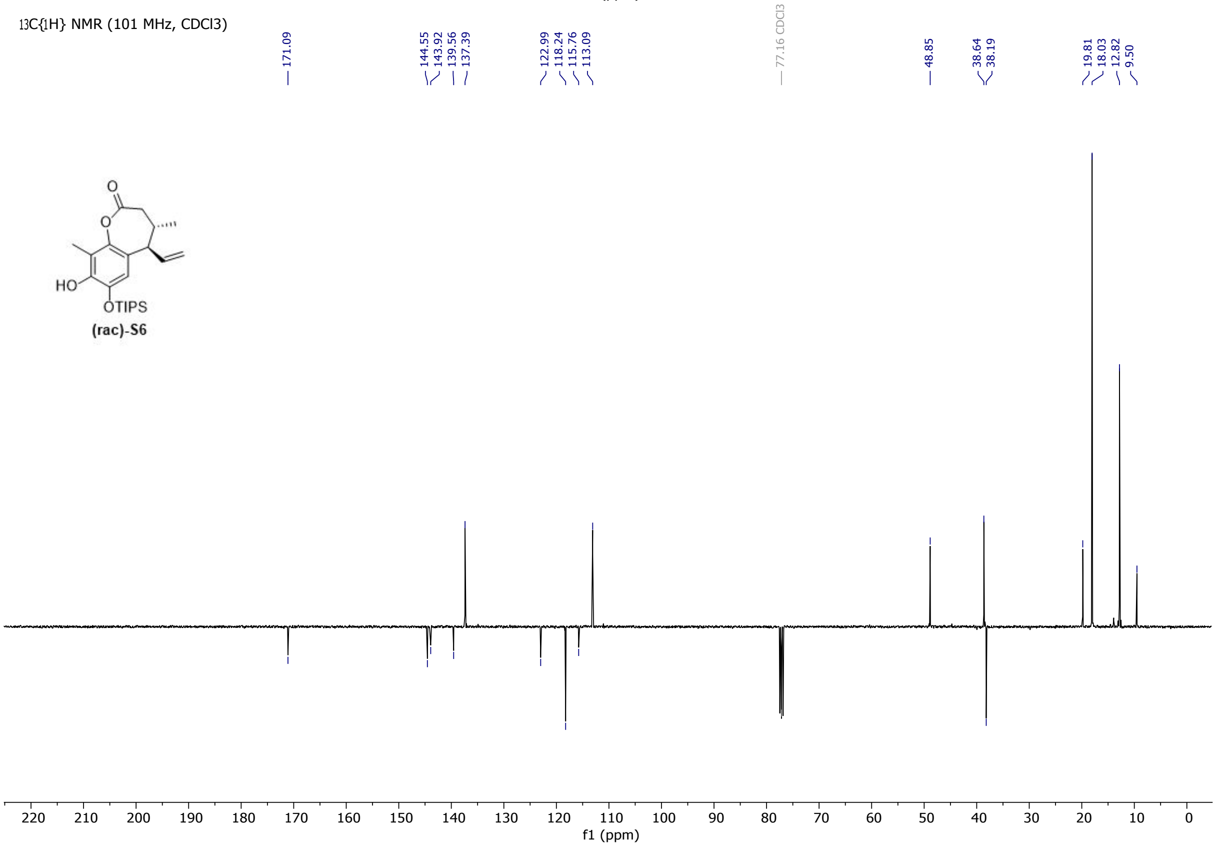

31. 8-(Methoxymethoxy)-4,9-dimethyl-7-((triisopropylsilyl)oxy)-5-vinyl-4,5-dihydrobenzo[b]oxepin-2(3H)-one (rac-32)

1H NMR (400 MHz, CDCl3)

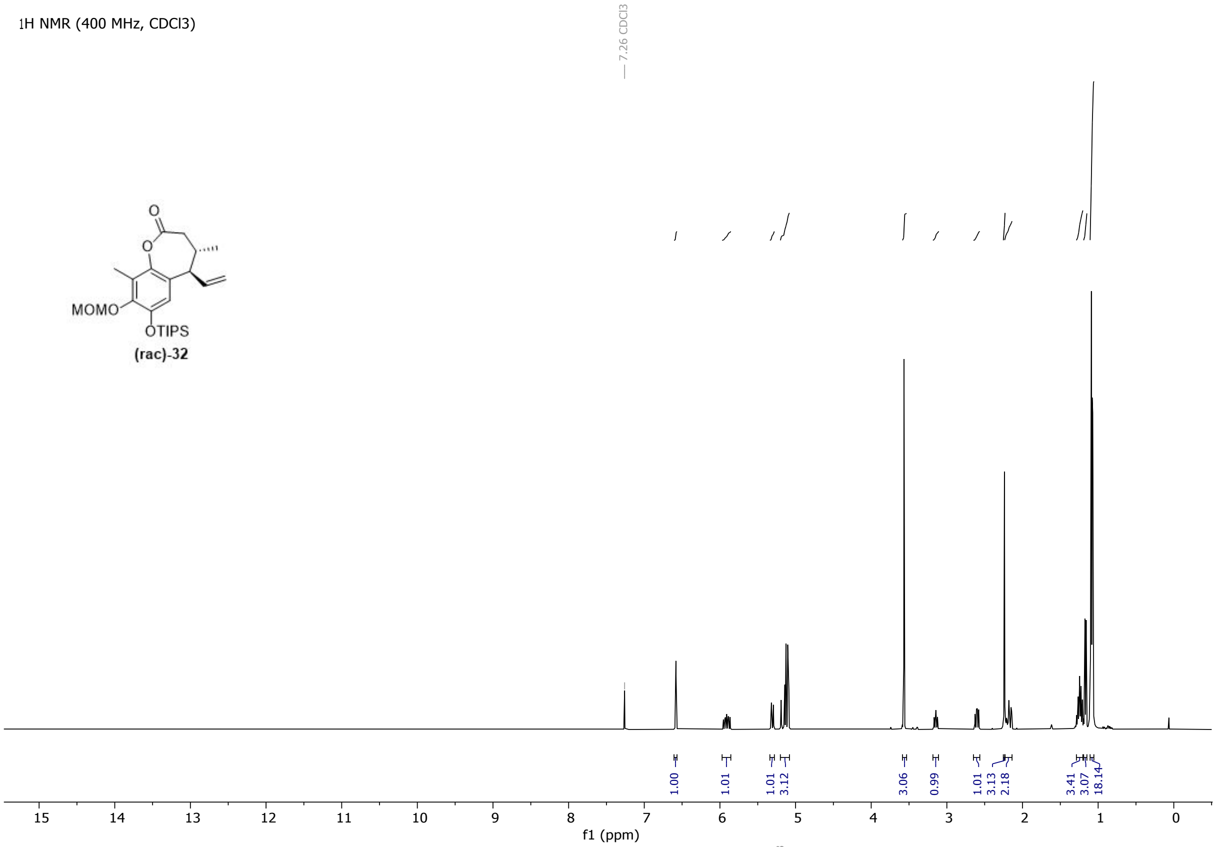

13C{1H} NMR (101 MHz, CDCl3)

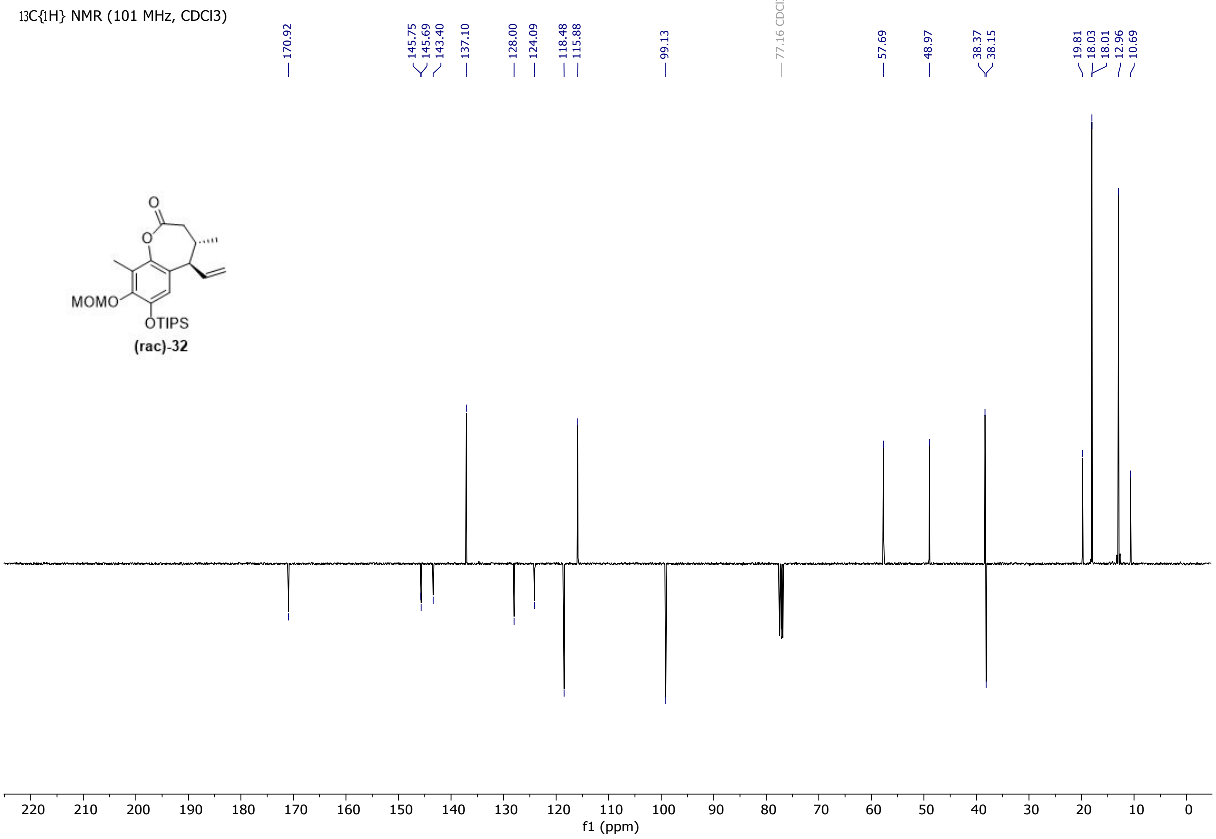

32. 7-Hydroxy-8-(methoxymethoxy)-4,9-dimethyl-5-vinyl-4,5-dihydrobenzo[b]oxepin-2(3H)-one (rac-37)

1H NMR (400 MHz, CDCl3)

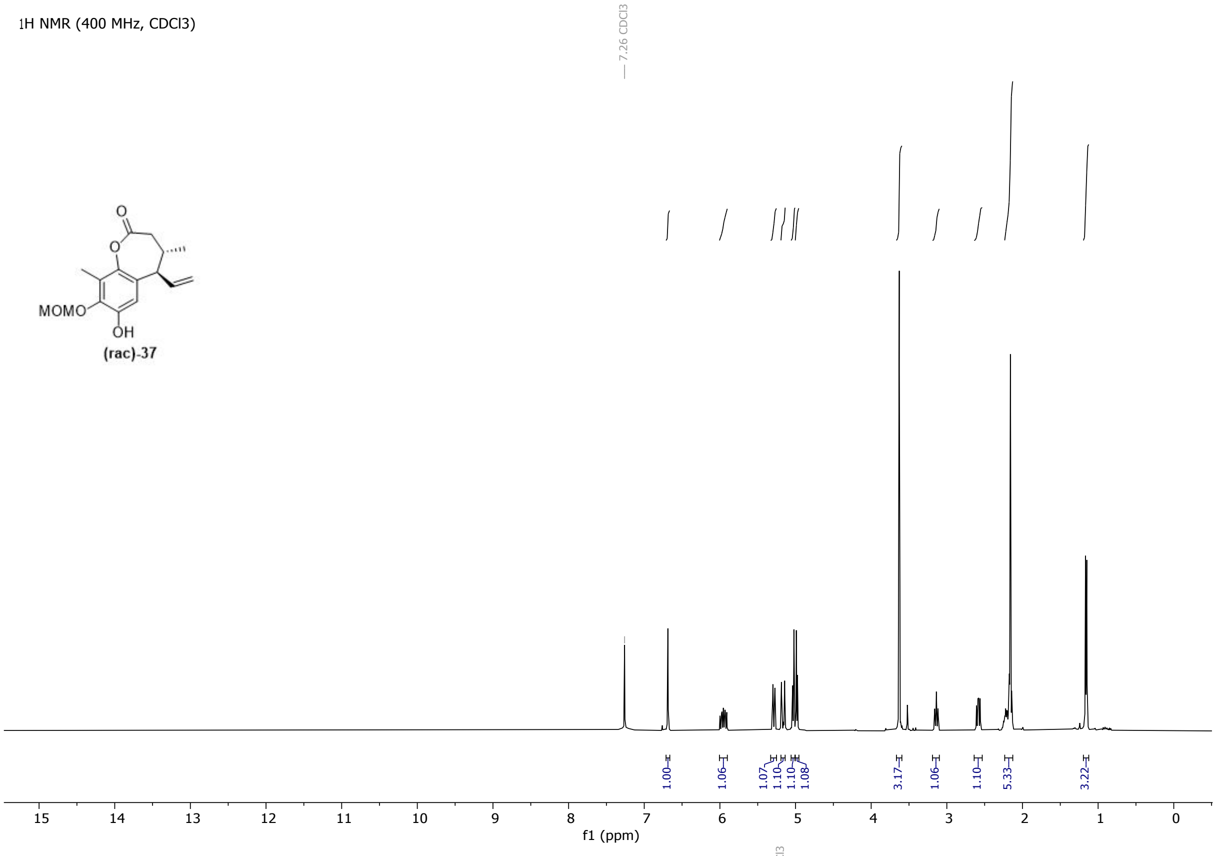

13C{1H} NMR (101 MHz, CDCl3)

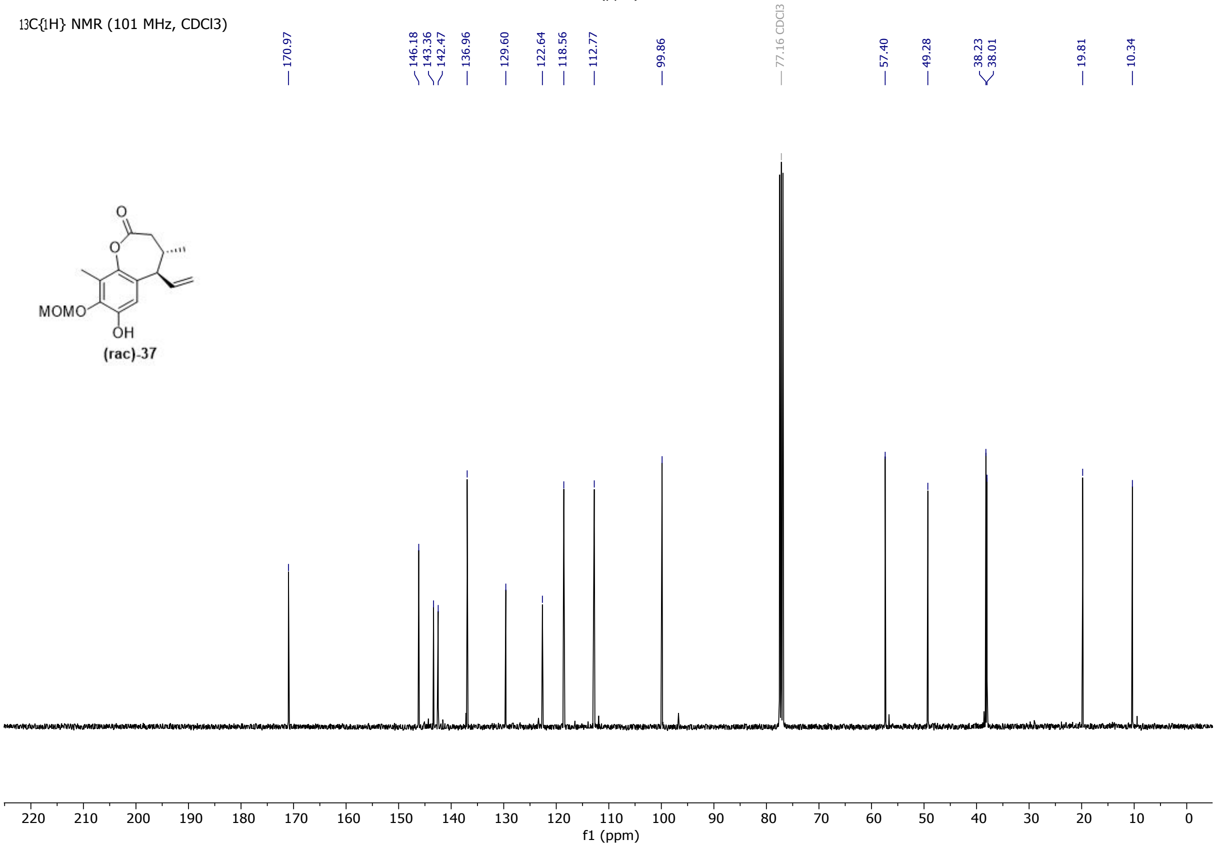

33. (4*S*,5*R*)-7-(((*S*,*E*)-1-(allyloxy)pent-3-en-2-yl)oxy)-8-(methoxymethoxy)-4,9-dimethyl-5-vinyl-4,5-dihydrobenzo[b]oxepin-2(3H)-one (38)

1H NMR (400 MHz, CDCl3)

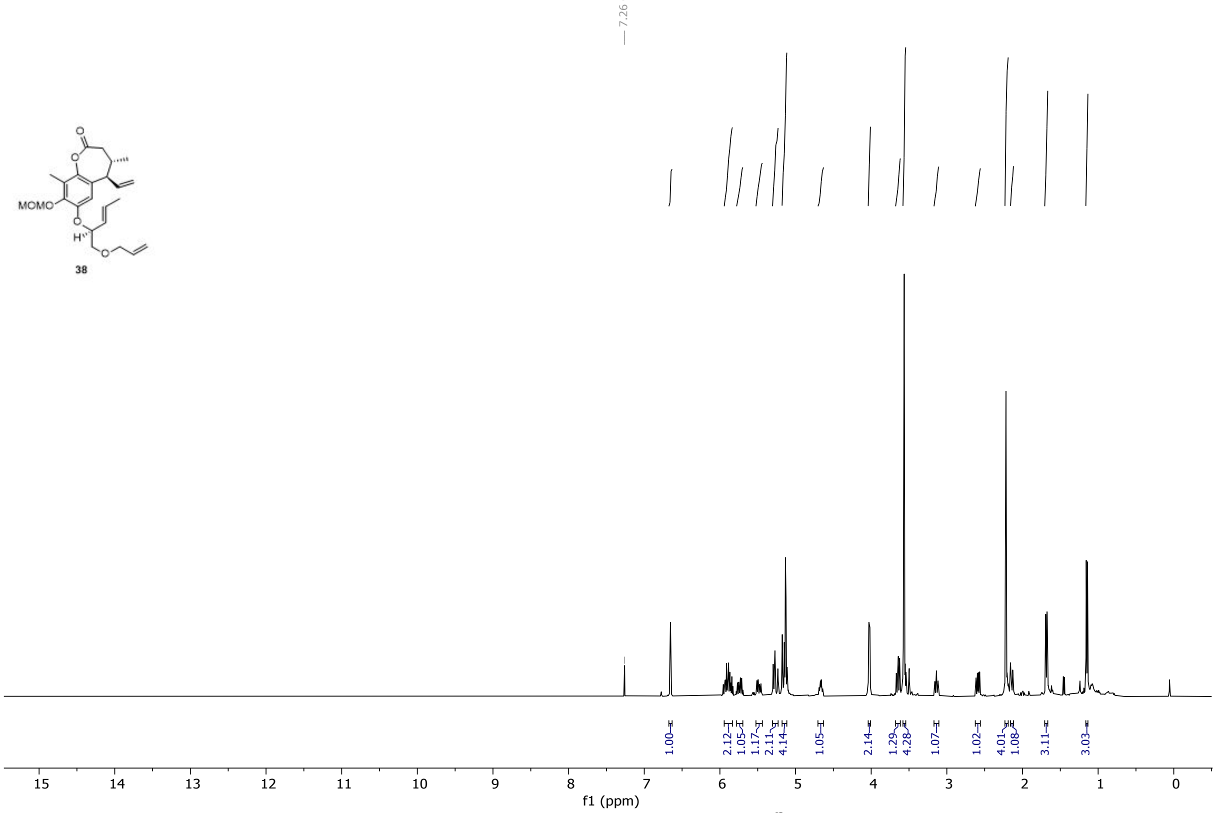

13C{1H} NMR (101 MHz, CDCl3)

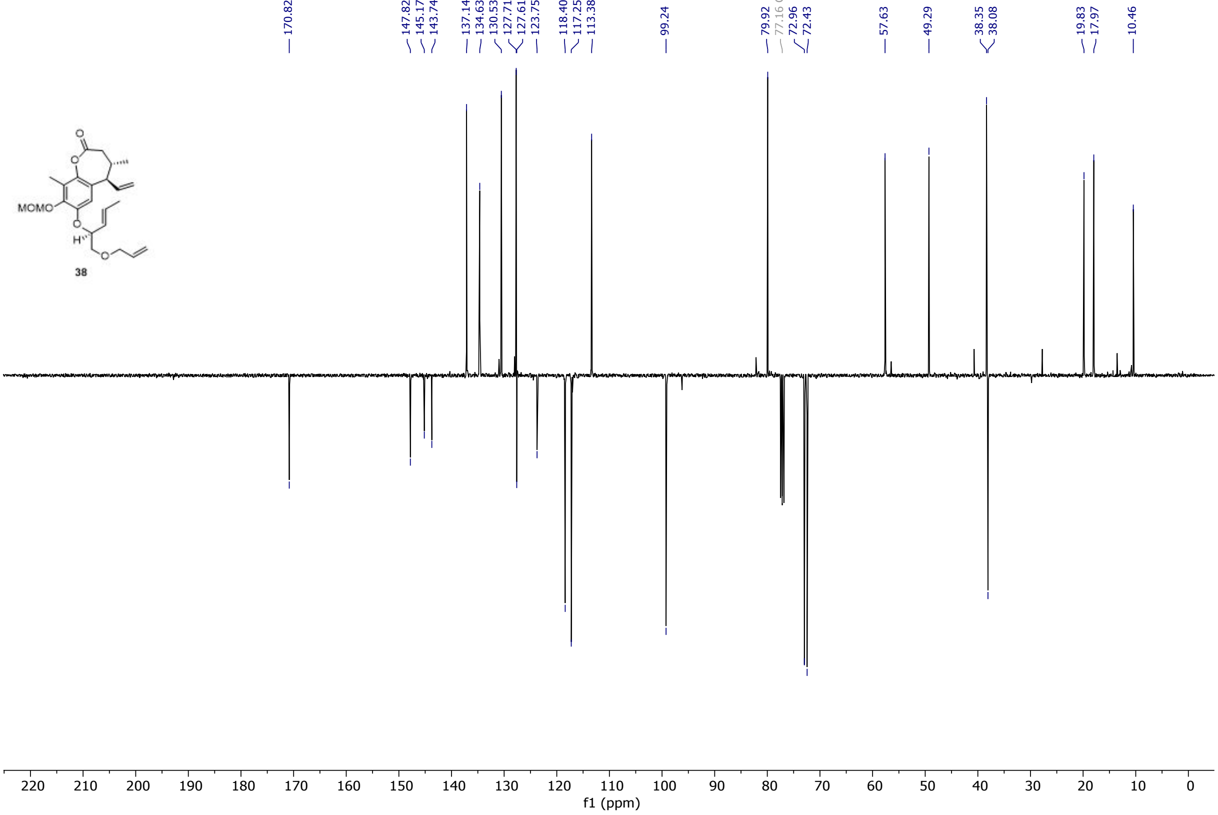

34. (4*R*,5*S*)-7-(((*S*,*E*)-1-(allyloxy)pent-3-en-2-yl)oxy)-8-(methoxymethoxy)-4,9-dimethyl-5-vinyl-4,5-dihydrobenzo[b]oxepin-2(3H)-one (38')

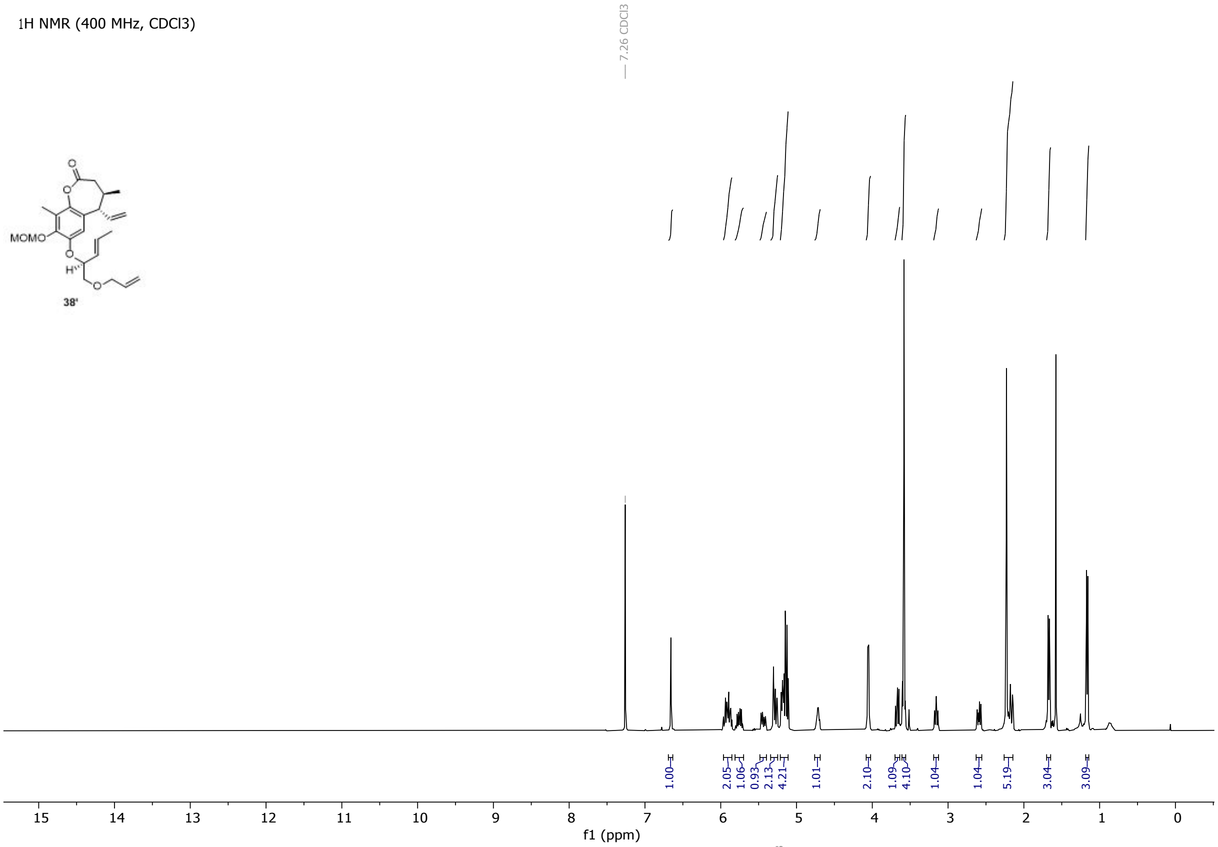

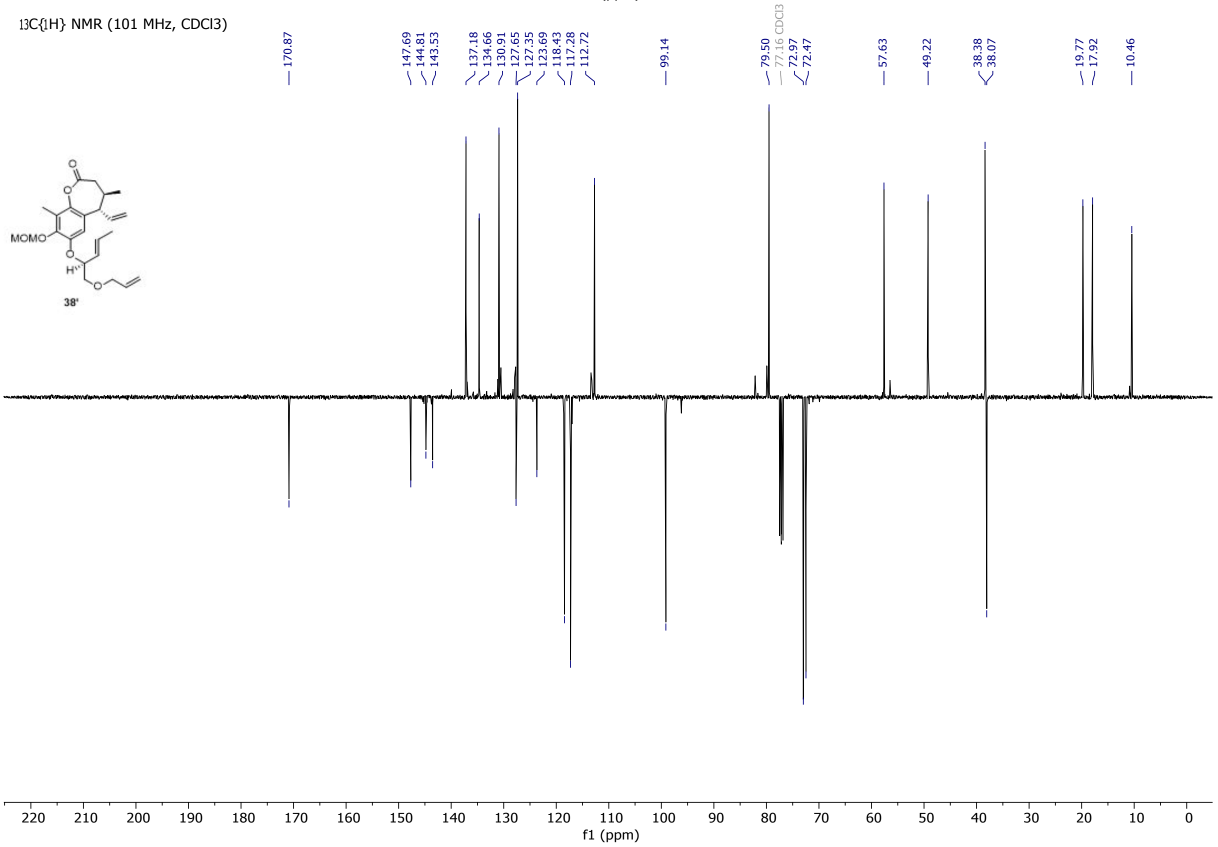

35. (4*S*,5*R*)-7-(((*S*,*E*)-1-(Allyloxy)pent-3-en-2-yl)oxy)-8-hydroxy-4,9-dimethyl-5-vinyl-4,5-dihydrobenzo[b]oxepin-2(3H)-one (S7)

1H NMR (400 MHz, CDCl3)

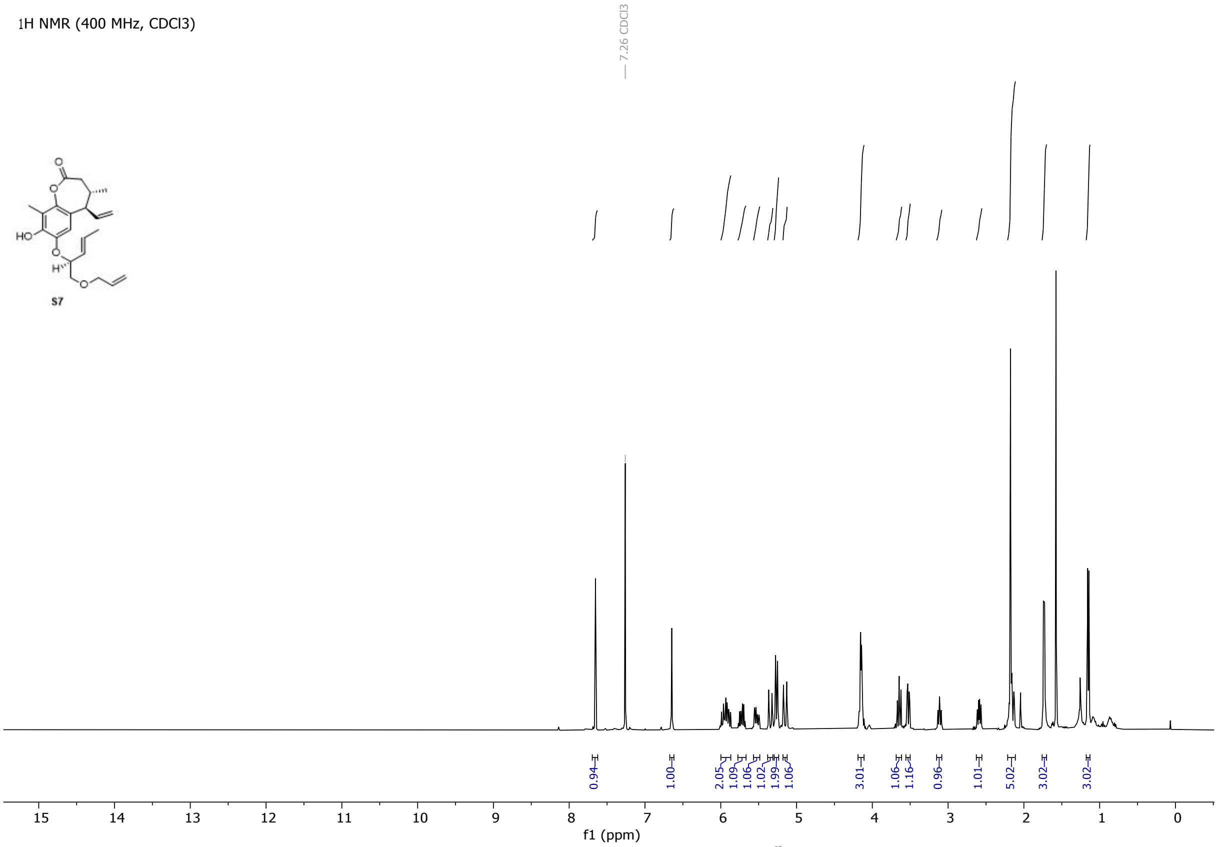

13C{1H} NMR (101 MHz, CDCl3)

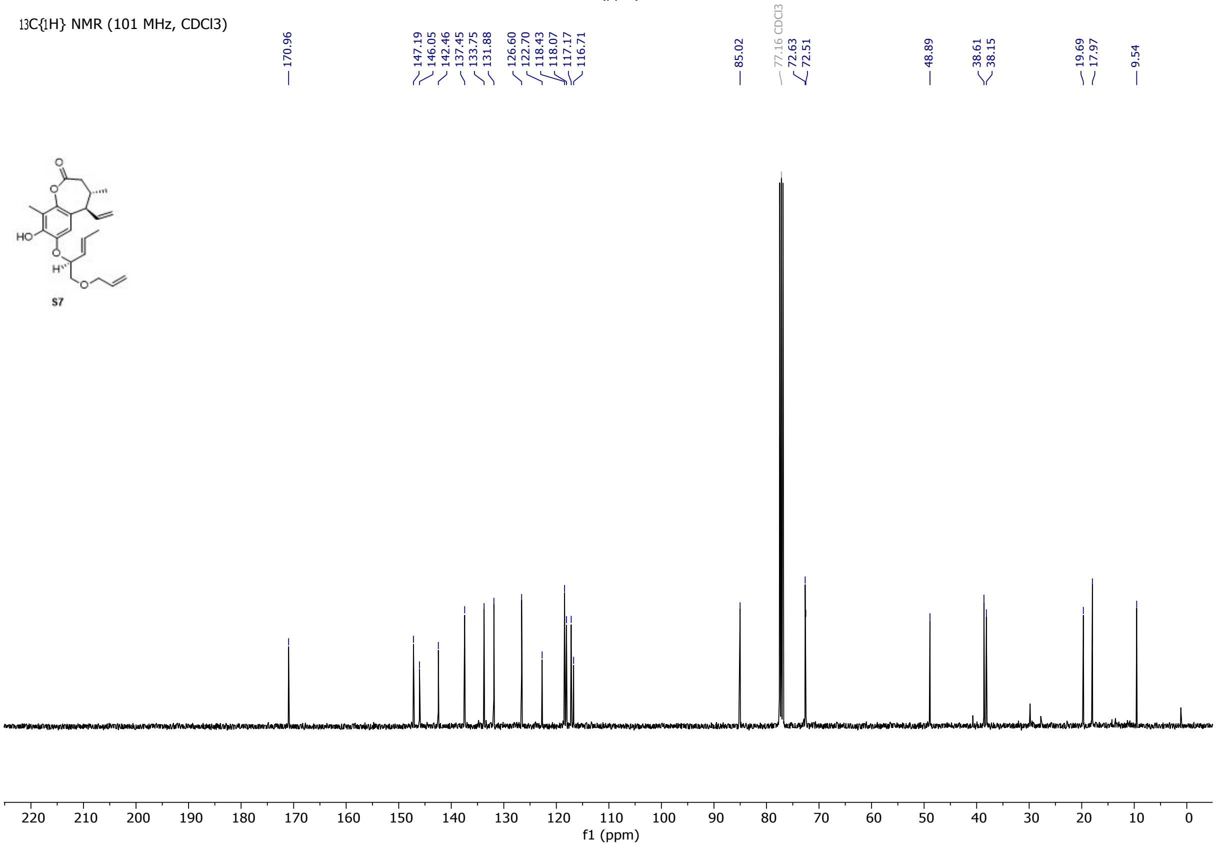

36. (4*S*,5*R*)-7-(((*S*,*E*)-1-(Allyloxy)pent-3-en-2-yl)oxy)-4,9-dimethyl-8-((triethylsilyl)oxy)-5-vinyl-4,5-dihydrobenzo[b]oxepin-2(3H)-one (31)

1H NMR (400 MHz, CDCl3)

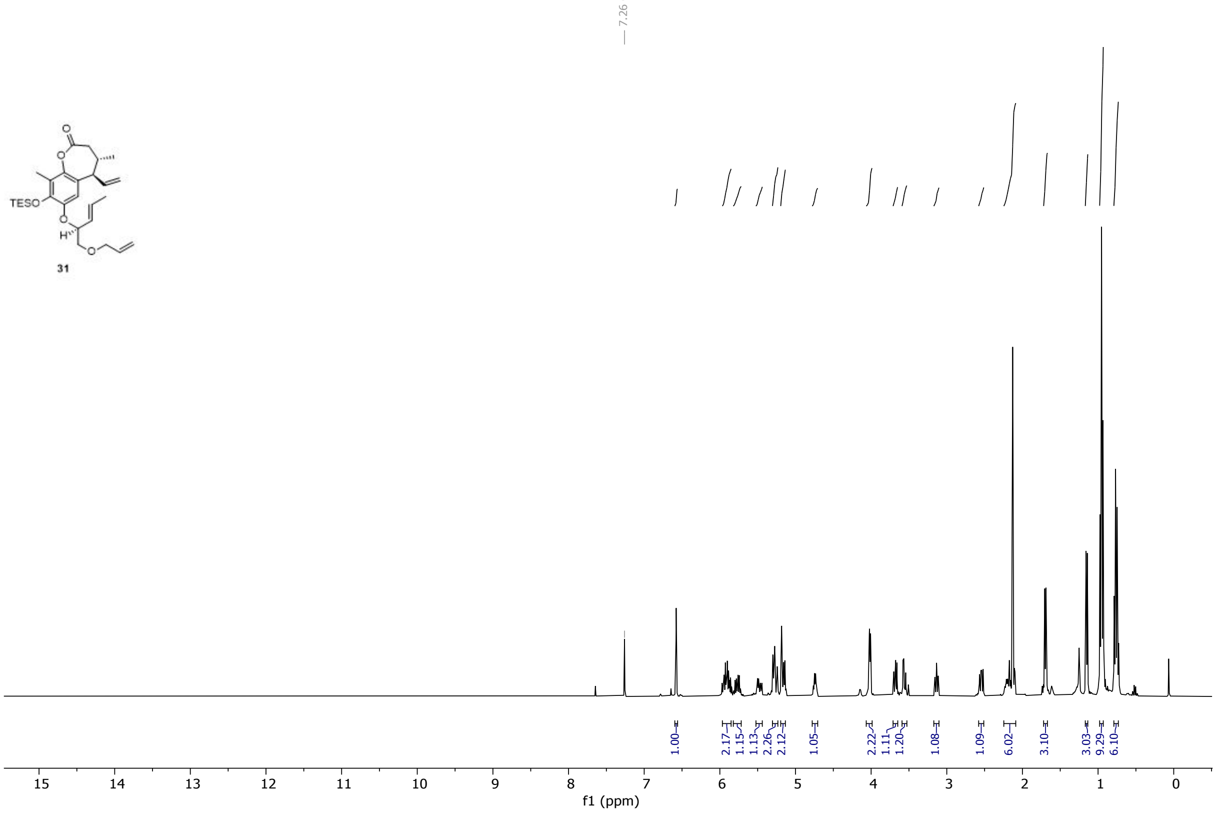

13C{1H} NMR (101 MHz, CDCl3)

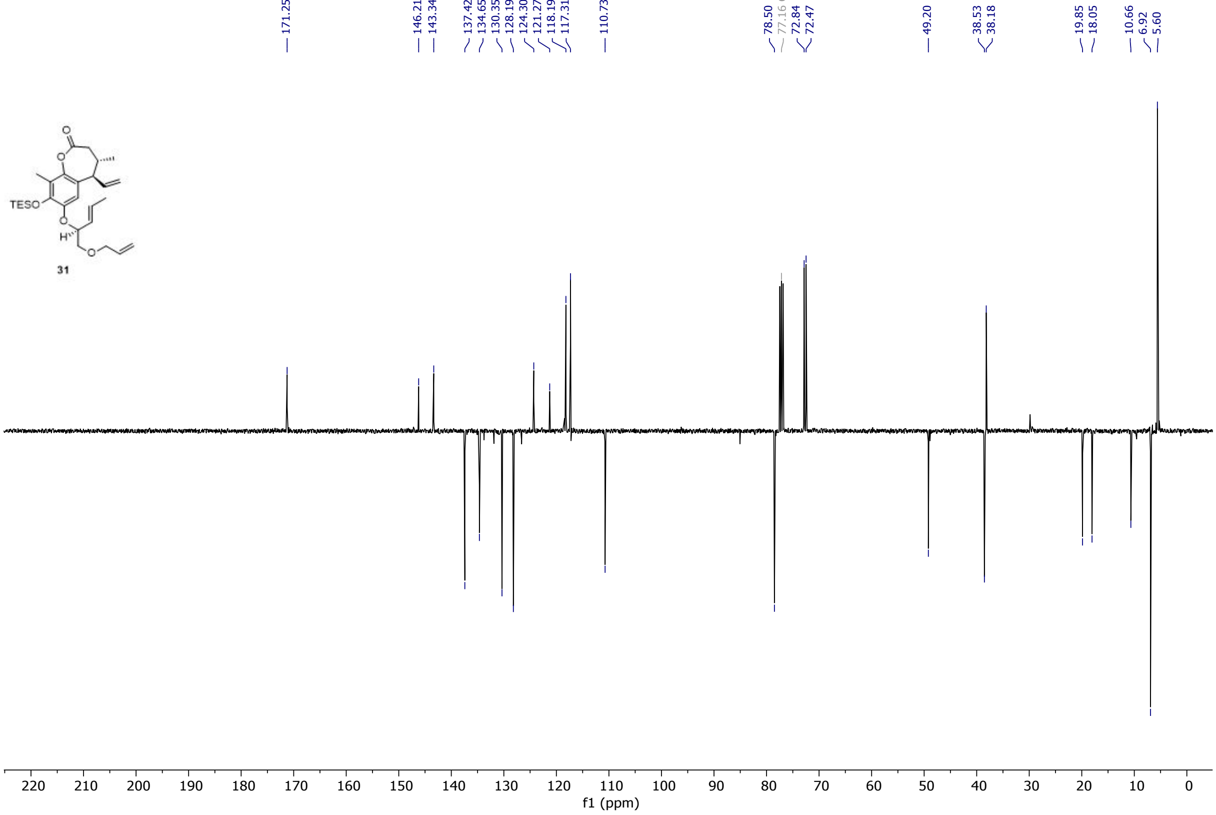

37. (3*S*,4*R*)-4-(5-(((*S*,*E*)-1-(Allyloxy)pent-3-en-2-yl)oxy)-2-hydroxy-3-methyl-4-((triethylsilyl)oxy)phenyl)-N-methoxy-N,3-dimethylhex-5-enamide (S8)

1H NMR (400 MHz, CDCl3)

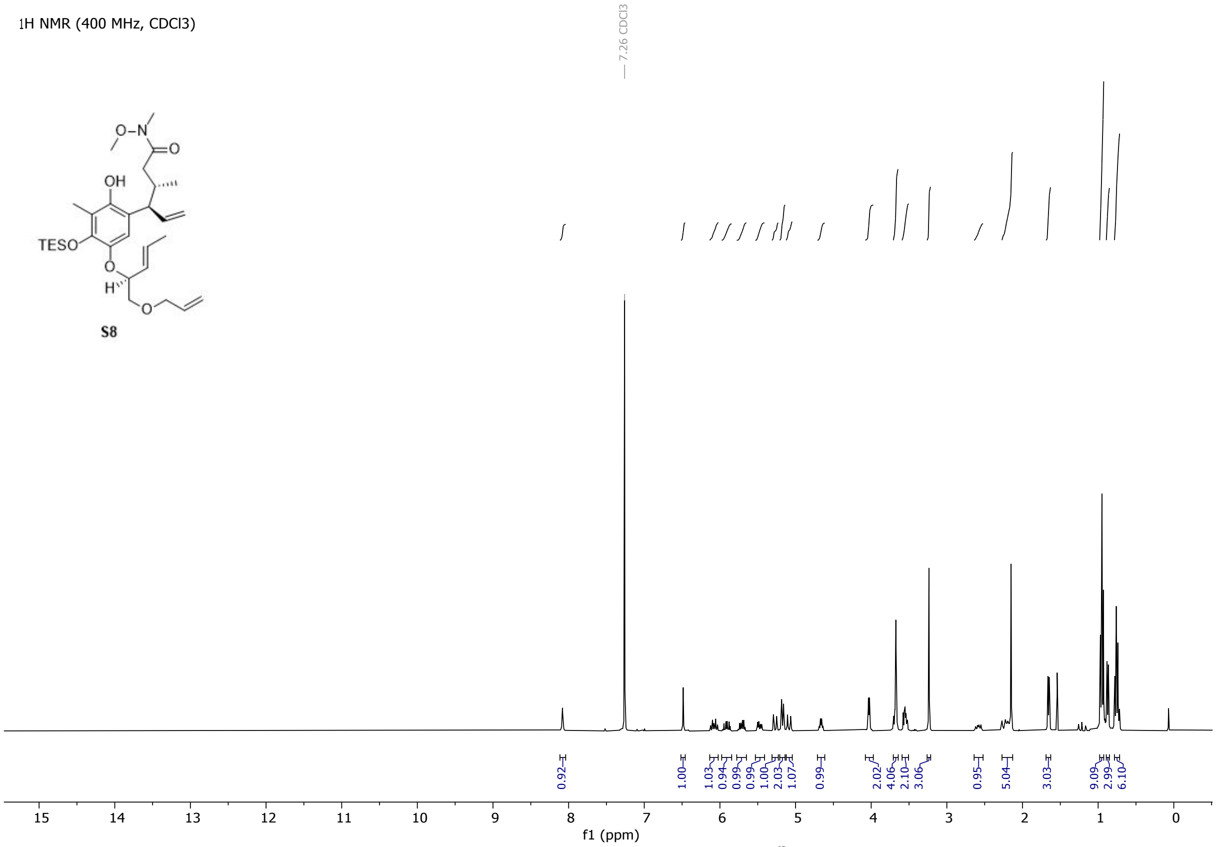

13C{1H} NMR (101 MHz, CDCl3)

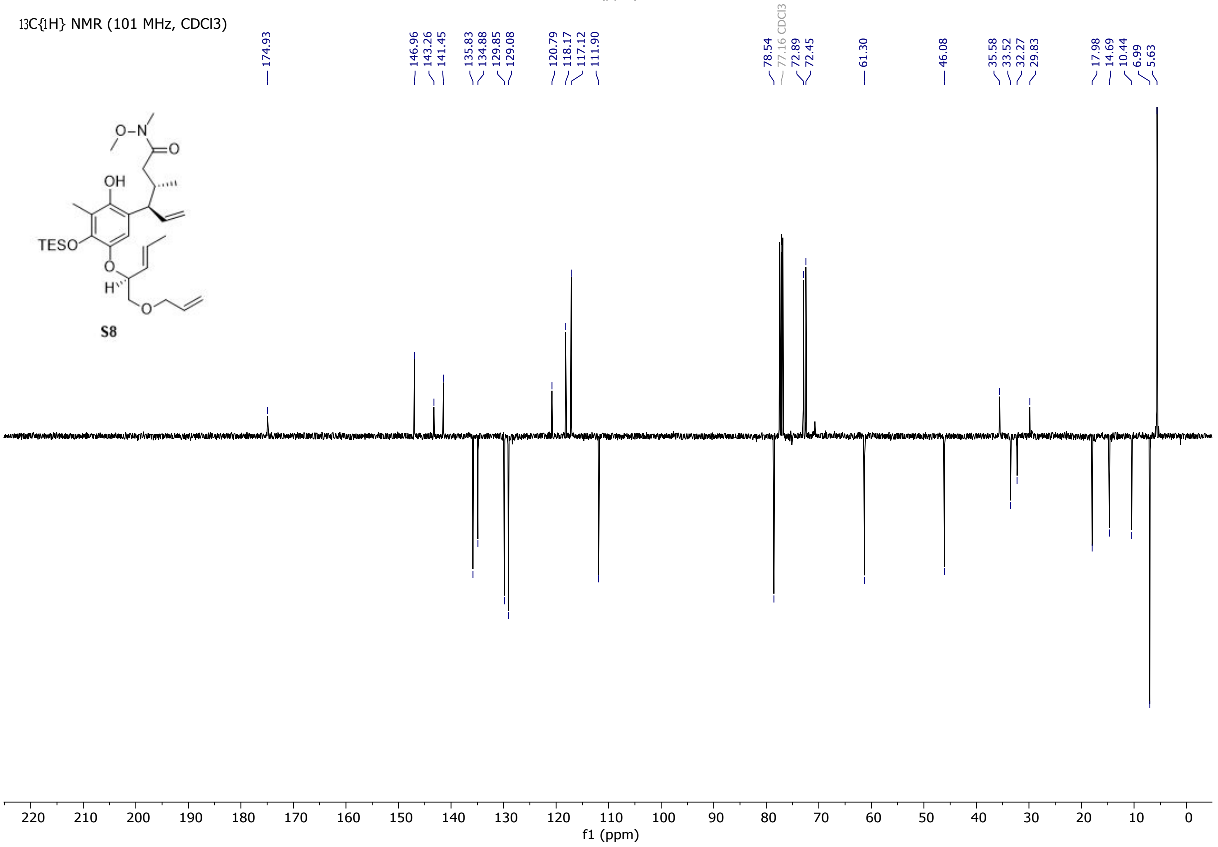

38. (3*S*,4*R*)-4-(5-(((*S*,*E*)-1-(Allyloxy)pent-3-en-2-yl)oxy)-2-((tert-butyldimethylsilyl)oxy)-3-methyl-4-((triethylsilyl)oxy)phenyl)-N-methoxy-N,3-dimethylhex-5-enamide (39)

1H NMR (400 MHz, CDCl3)

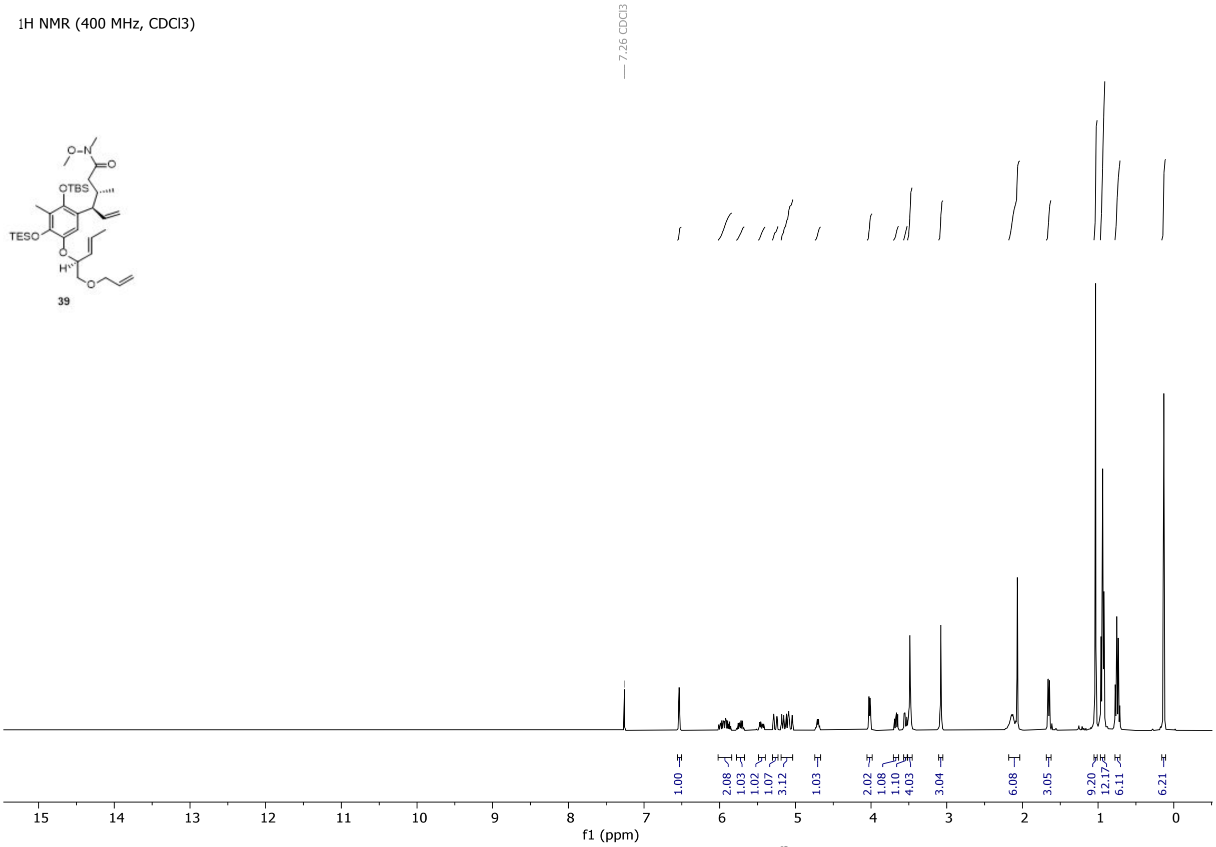

13C{1H} NMR (101 MHz, CDCl3)

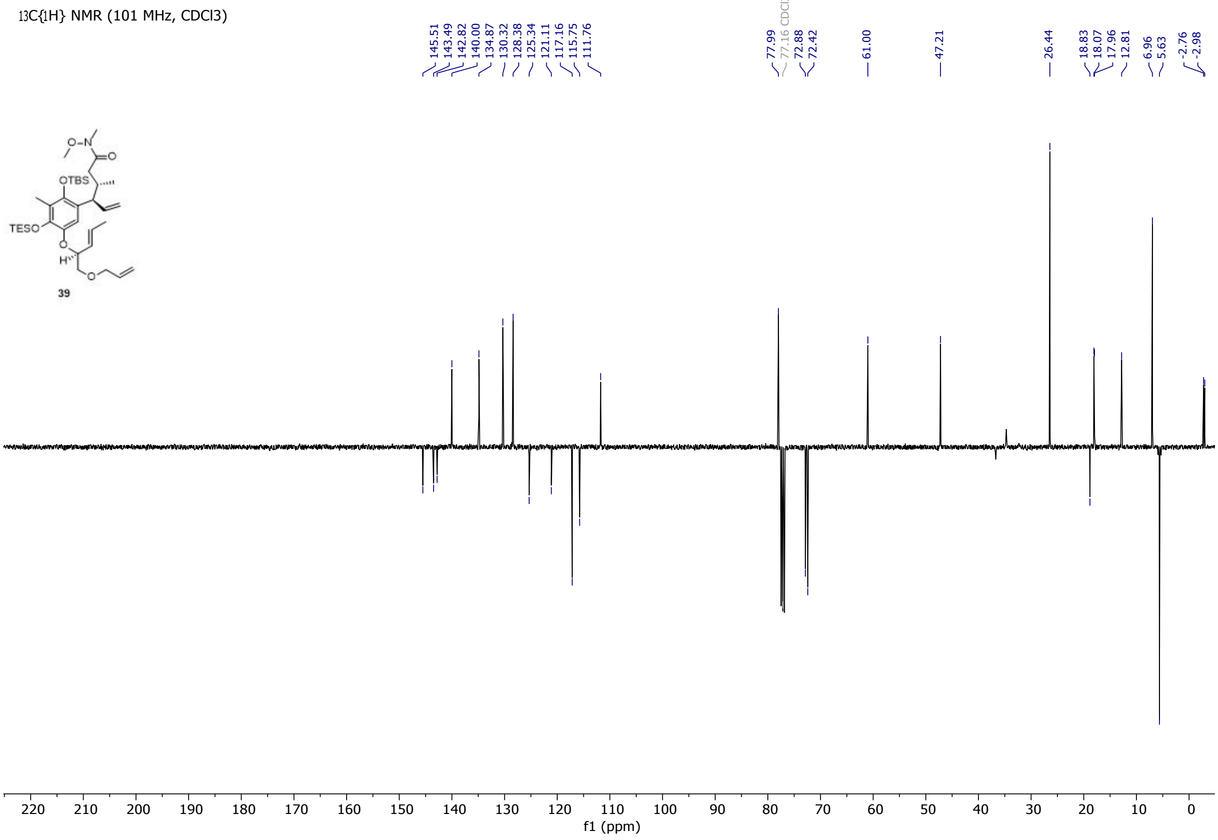

39. (5*S*,6*R*)-6-(5-(((*S*,*E*)-1-(allyloxy)pent-3-en-2-yl)oxy)-2-((tert-butyldimethylsilyl)oxy)-3-methyl-4-((triethylsilyl)oxy)phenyl)-5-methylocta-1,7-dien-3-one (S9)

1H NMR (400 MHz, CDCl3)

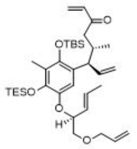

S9

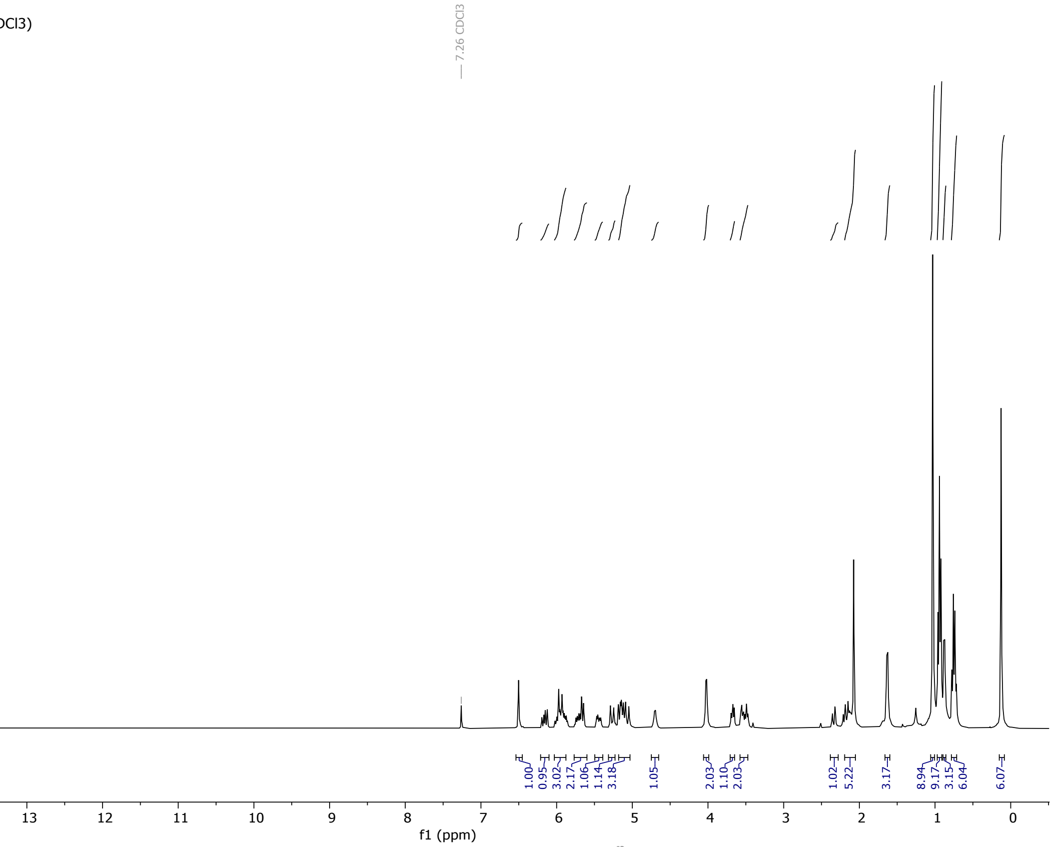

13C{1H} NMR (101 MHz, CDCl3)

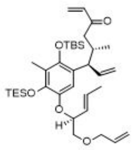

S9

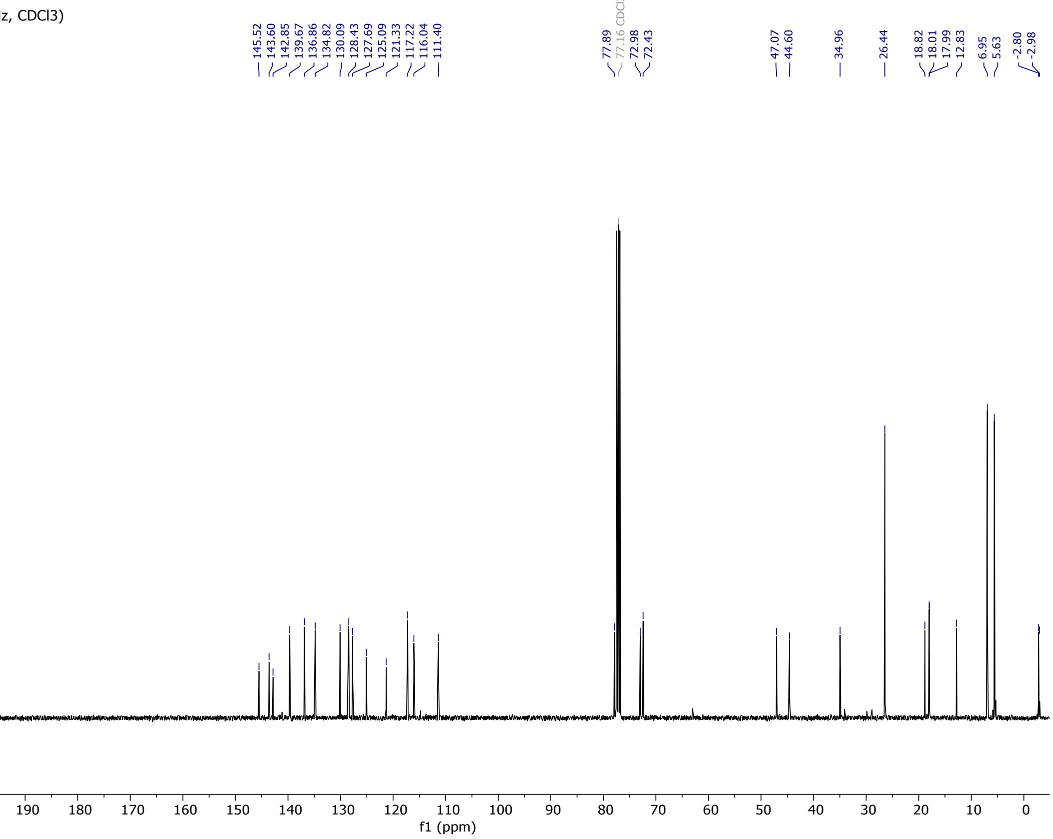

40. (3*S*,5*S*,6*R*)-6-(5-(((*S*,*E*)-1-(Allyloxy)pent-3-en-2-yl)oxy)-2-((tert-butyldimethylsilyl)oxy)-3-methyl-4-((triethylsilyl)oxy)phenyl)-5-methylocta-1,7-dien-3-ol (40)

1H NMR (400 MHz, CDCl3)

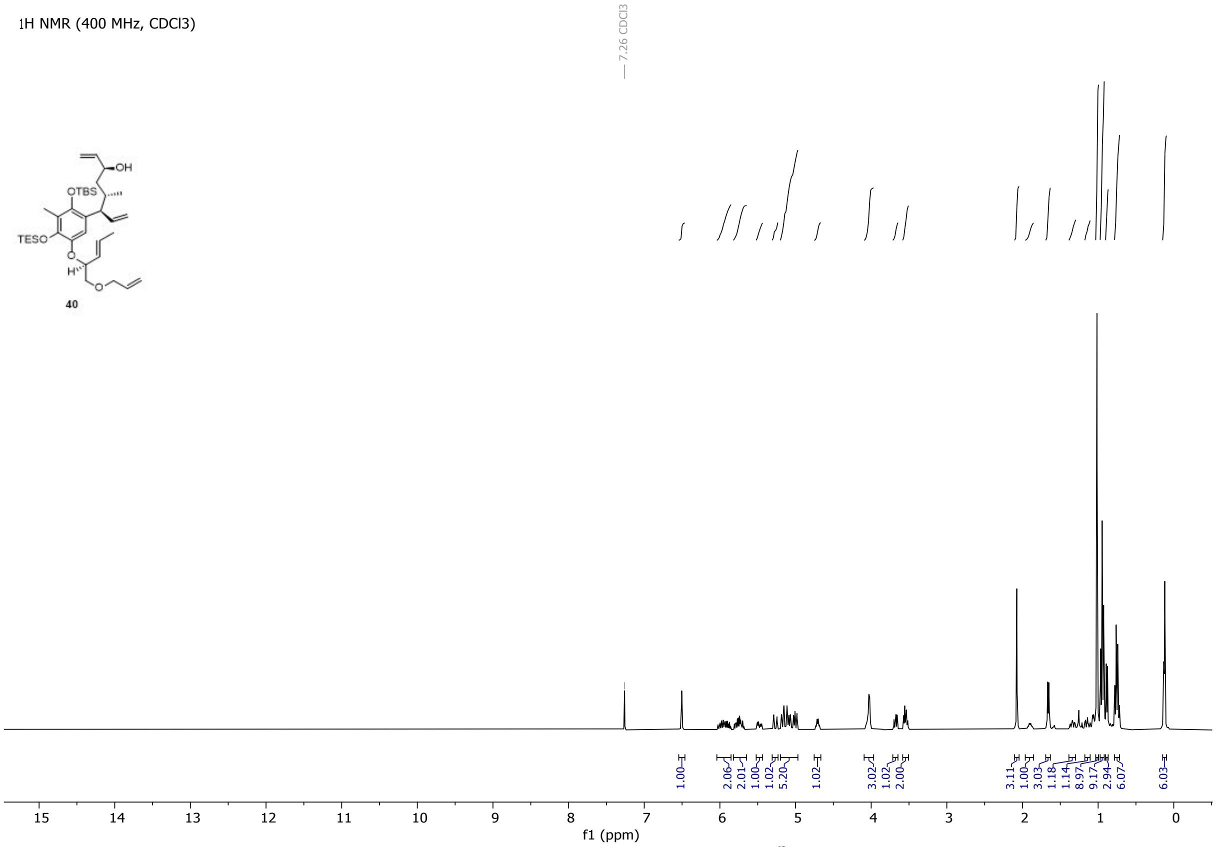

13C{1H} NMR (101 MHz, CDCl3)

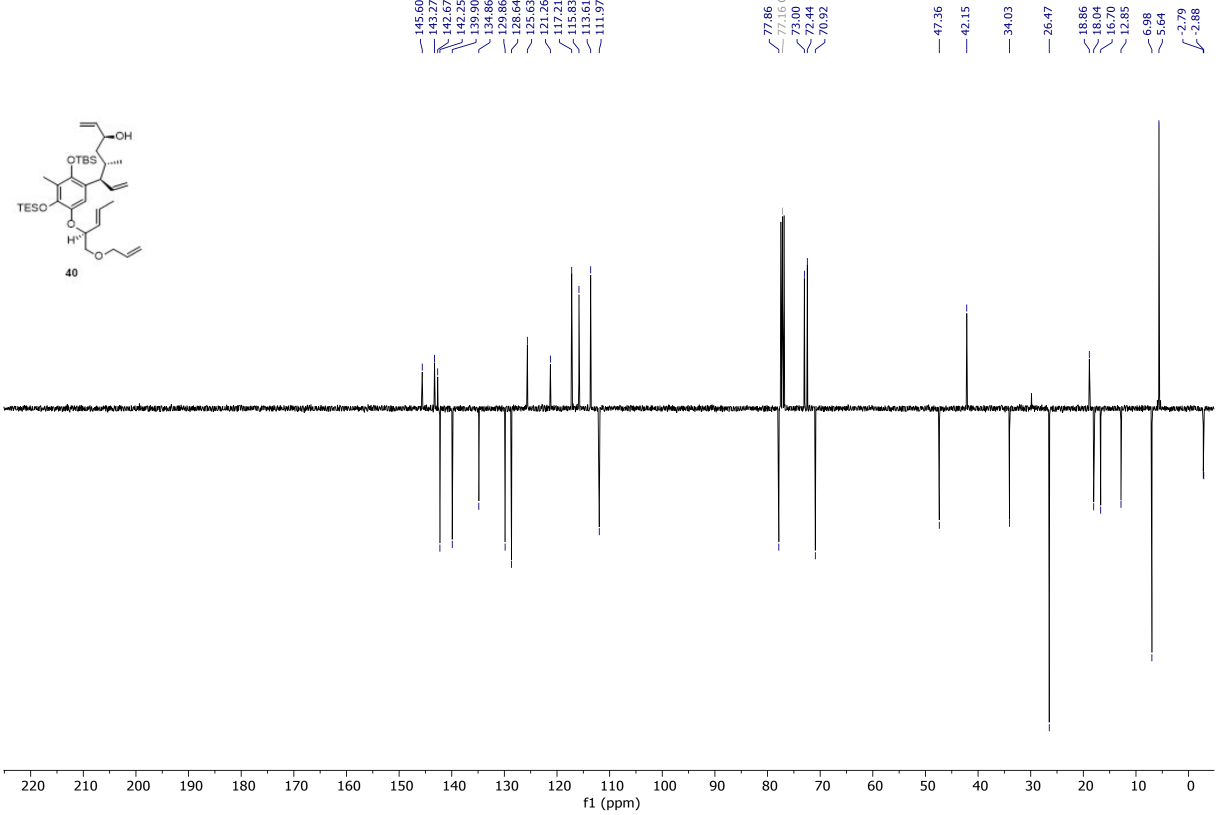

41. (3*S*,5*S*,6*R*)-6-(5-(((*S*,*E*)-1-(Allyloxy)pent-3-en-2-yl)oxy)-2-((tert-butyldimethylsilyl)oxy)-3-methyl-4-((triethylsilyl)oxy)phenyl)-5-methylocta-1,7-dien-3-yl methyl carbonate (S10)

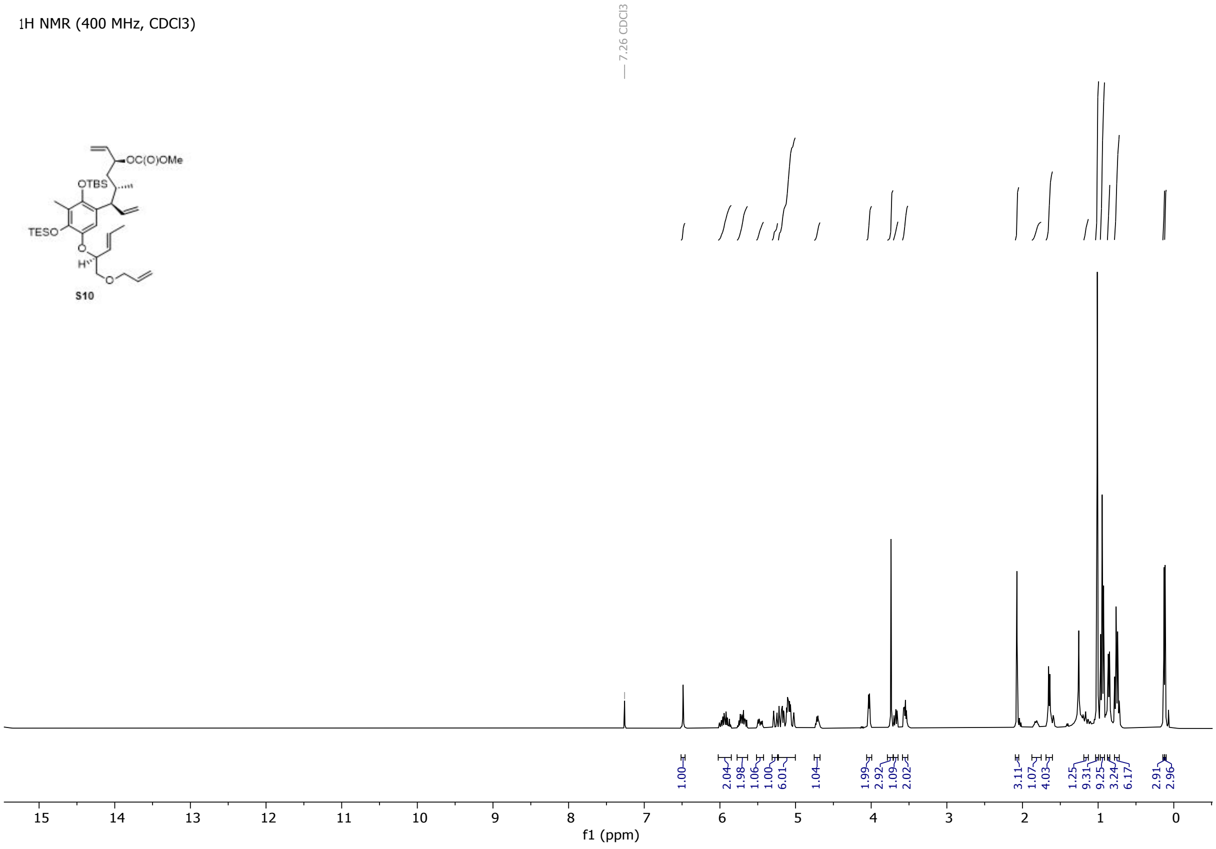

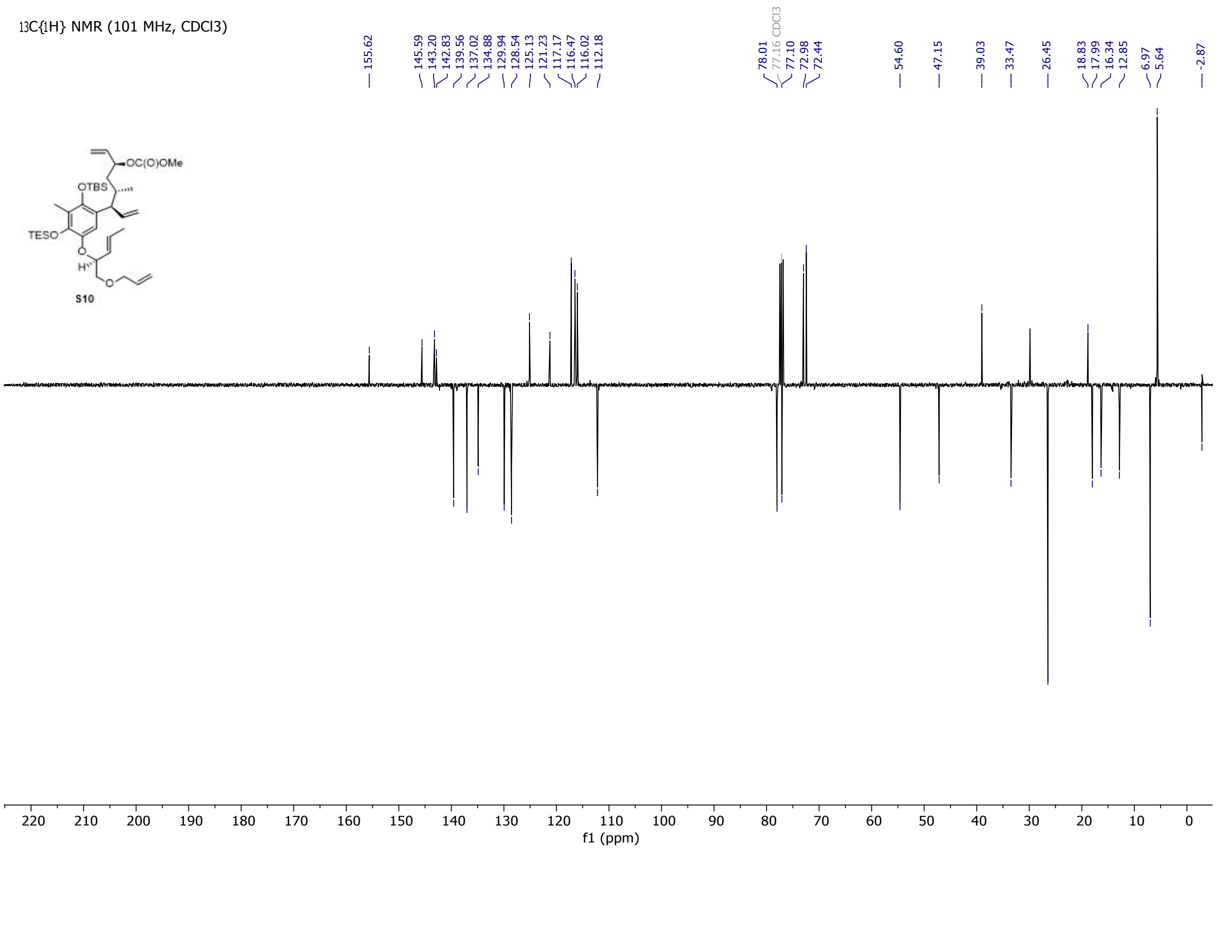

42. (3*S*,5*S*,6*R*)-6-(5-(((*S*,*E*)-1-(Allyloxy)pent-3-en-2-yl)oxy)-2-((tert-butyldimethylsilyl)oxy)-4-hydroxy-3-methylphenyl)-5-methylocta-1,7-dien-3-yl methyl carbonate (30)

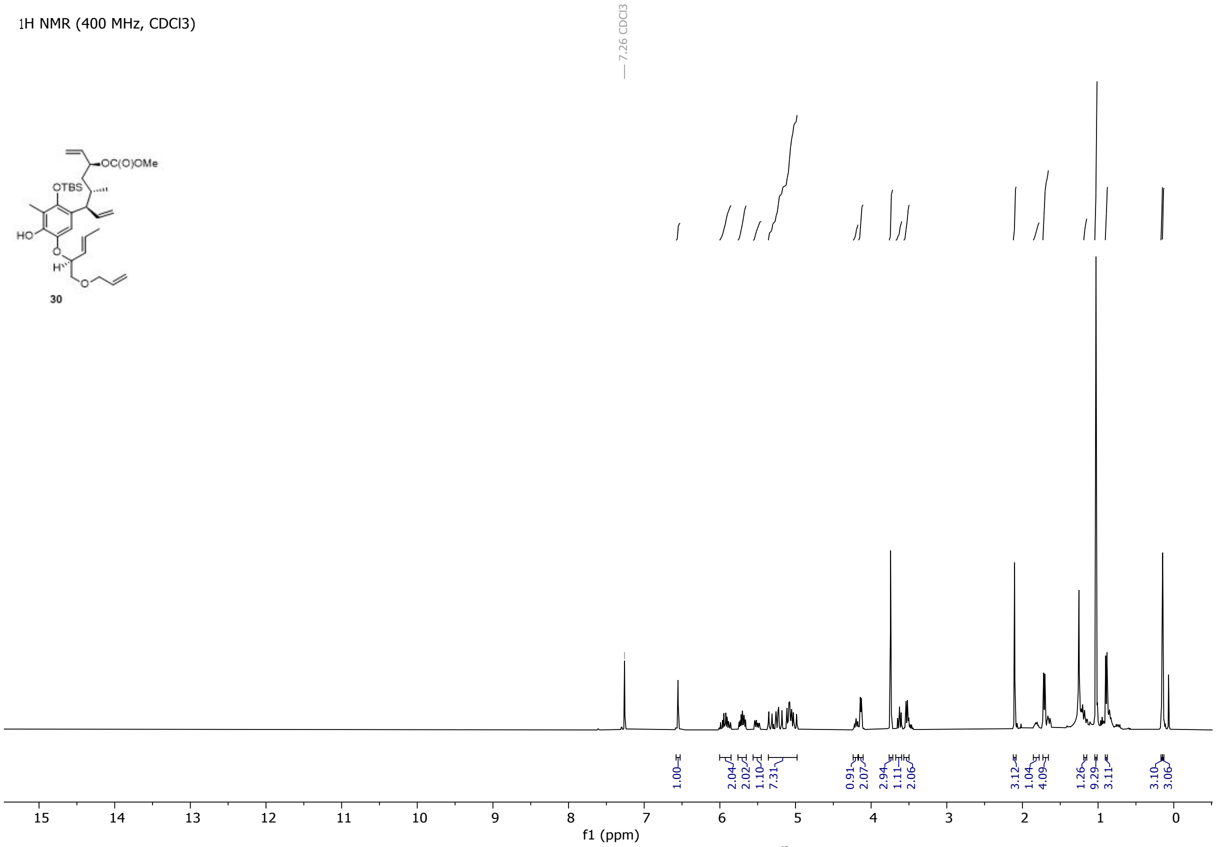

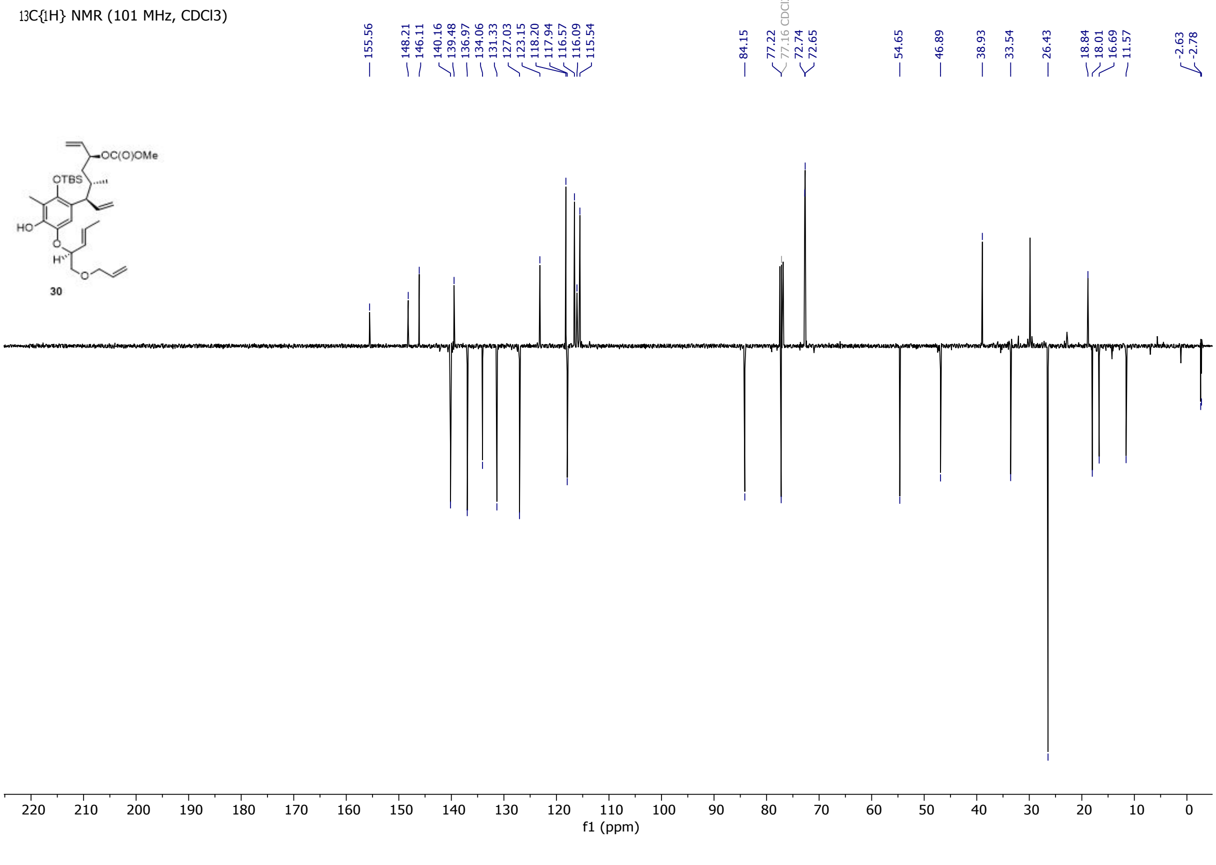

43. (3*S*,4*R*)-4-(5-(((*S*,*E*)-1-(Allyloxy)pent-3-en-2-yl)oxy)-2-((tert-butyldimethylsilyl)oxy)-3-methyl-4-((triethylsilyl)oxy)phenyl)-3-methylhex-5-enal (S11)

^{1}H NMR (400 MHz, CDCl3)

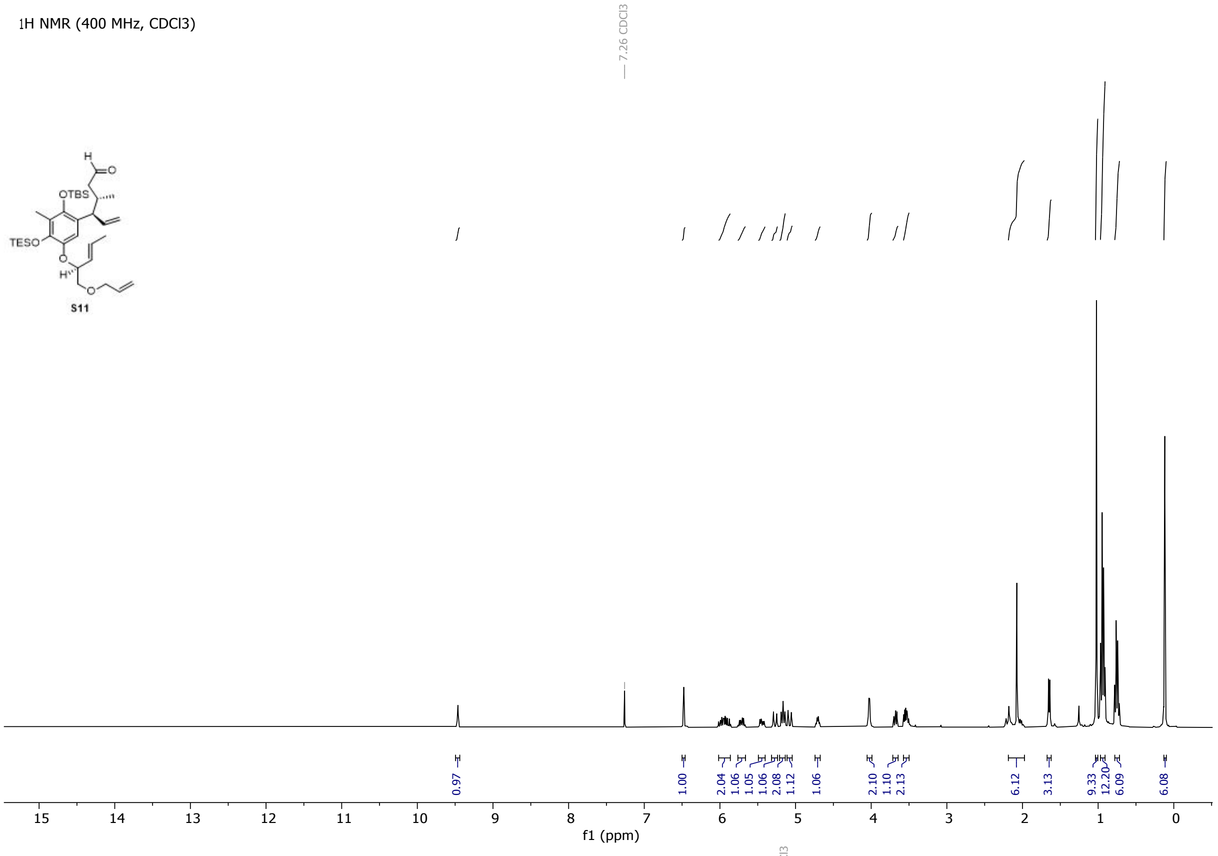

^{13}C{^{1}H} NMR (101 MHz, CDCl3)

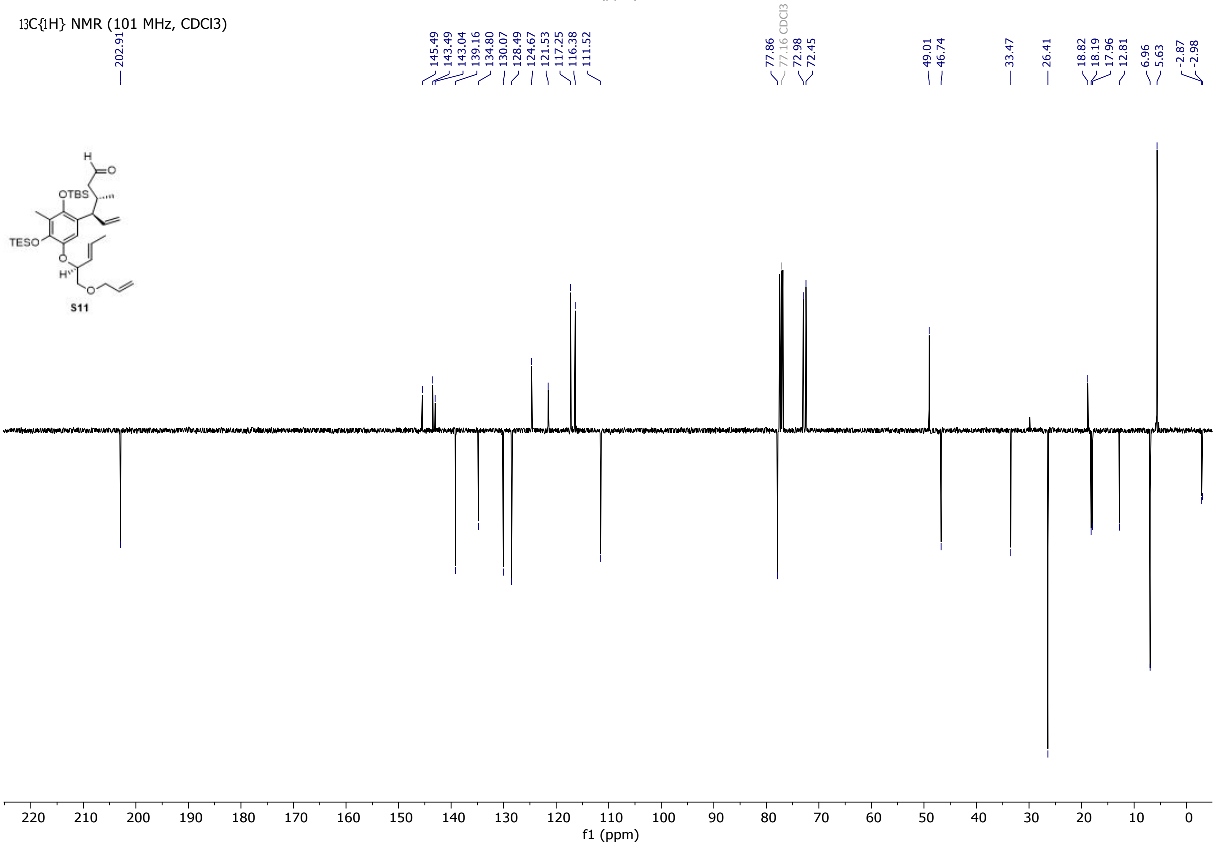

44. (3*R*,5*S*,6*R*)-6-(5-(((*S*,*E*)-1-(allyloxy)pent-3-en-2-yl)oxy)-2-((tert-butyldimethylsilyl)oxy)-3-methyl-4-((triethylsilyl)oxy)phenyl)-5-methylocta-1,7-dien-3-ol (40')

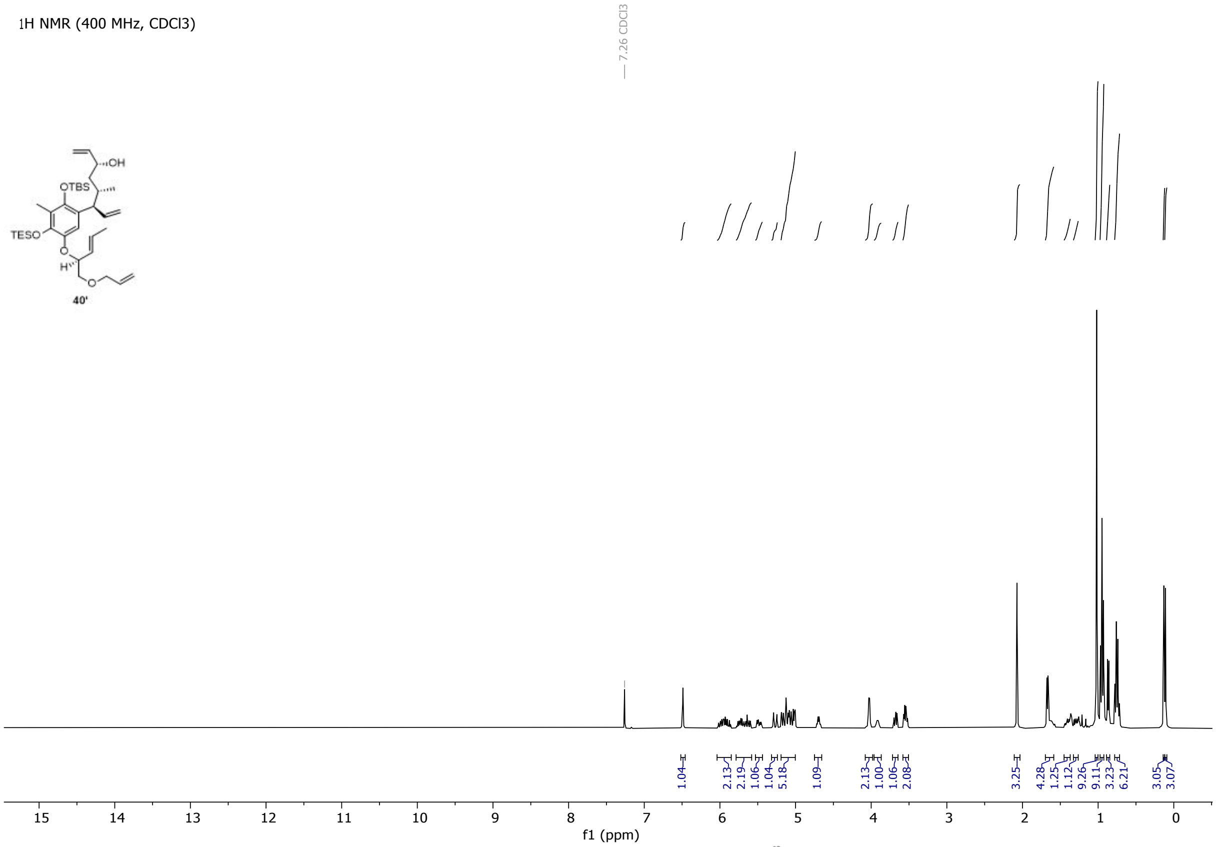

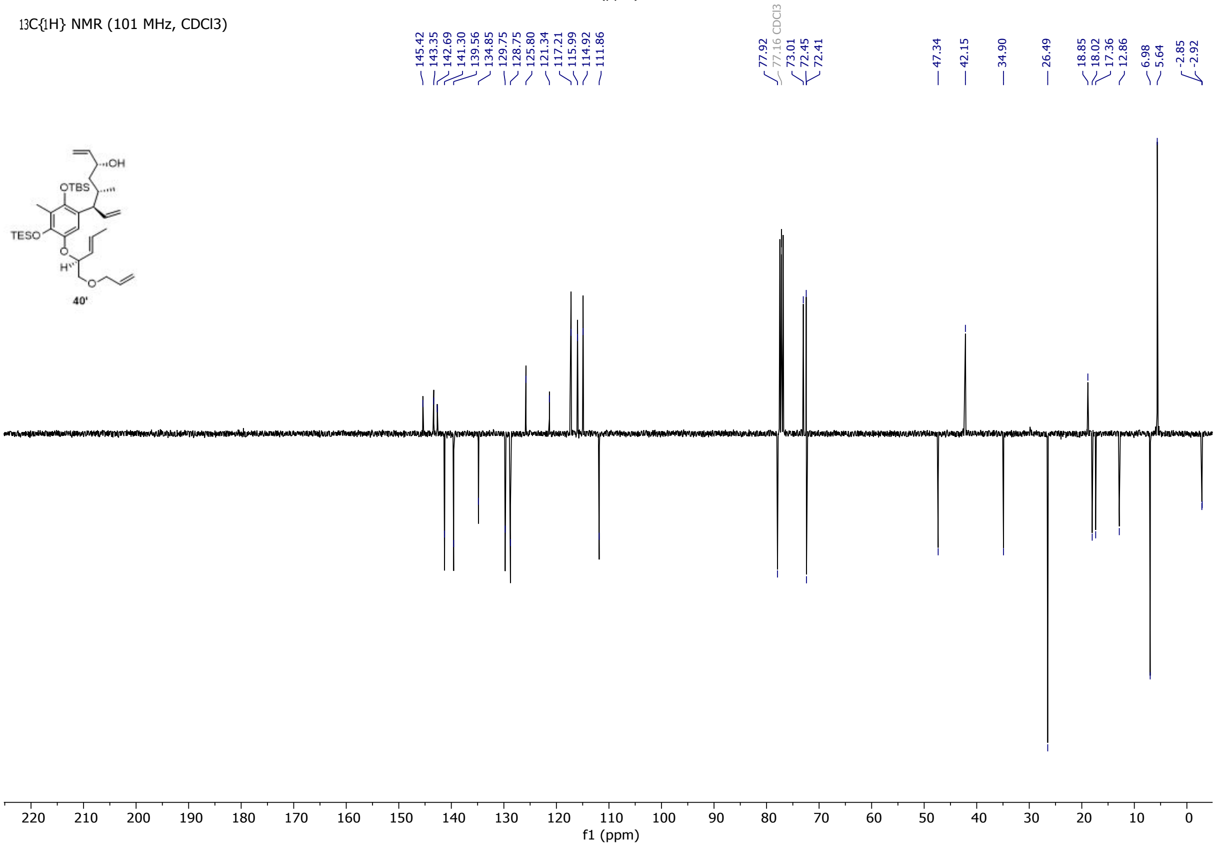

45. (3*R*,5*S*,6*R*)-6-(5-(((*S*,*E*)-1-(Allyloxy)pent-3-en-2-yl)oxy)-2-((tert-butyldimethylsilyl)oxy)-3-methyl-4-((triethylsilyl)oxy)phenyl)-5-methylocta-1,7-dien-3-yl methyl carbonate (S12)

1H NMR (400 MHz, CDCl3)

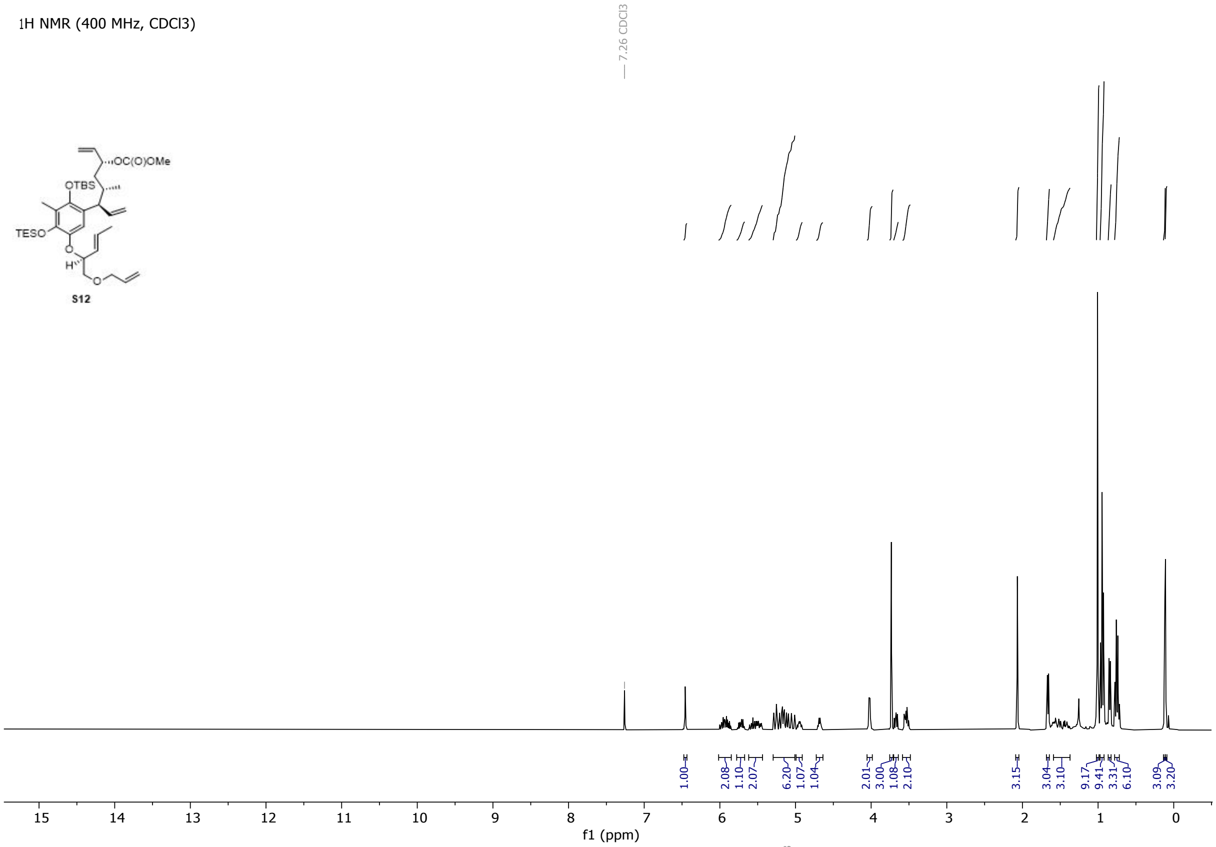

13C{1H} NMR (101 MHz, CDCl3)

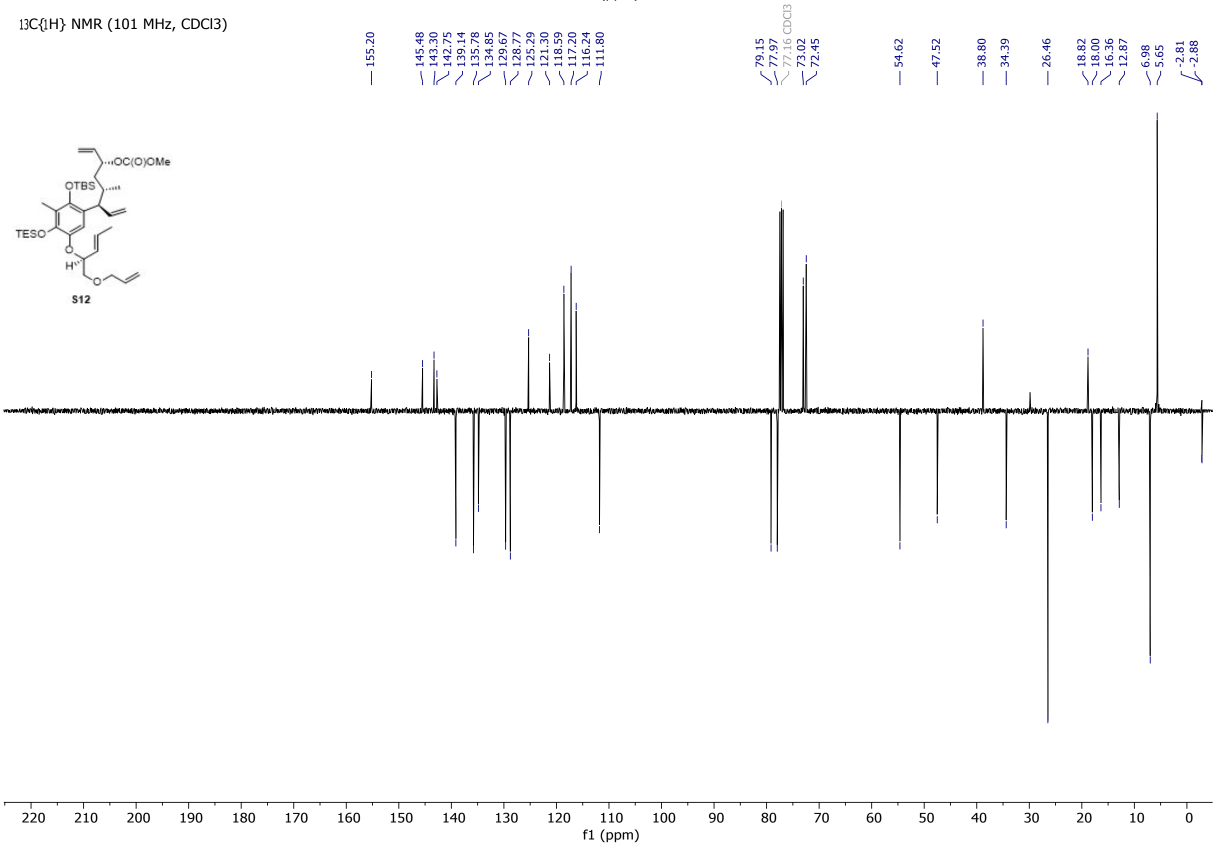

46. (3*R*,5*S*,6*R*)-6-(5-(((*S*,*E*)-1-(Allyloxy)pent-3-en-2-yl)oxy)-2-((tert-butyldimethylsilyl)oxy)-4-hydroxy-3-methylphenyl)-5-methylocta-1,7-dien-3-yl methyl carbonate (30')

1H NMR (400 MHz, CD2Cl2)

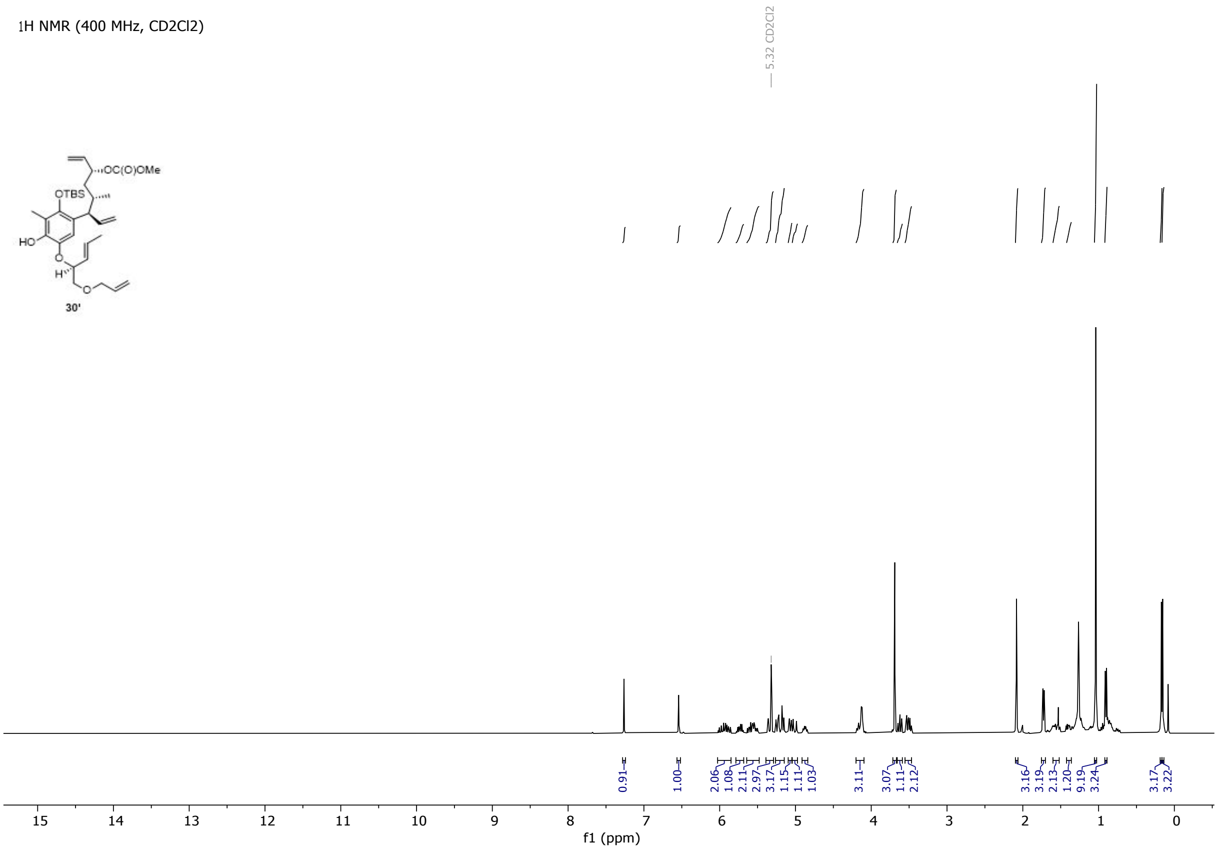

13C{1H} NMR (101 MHz, CD2Cl2)

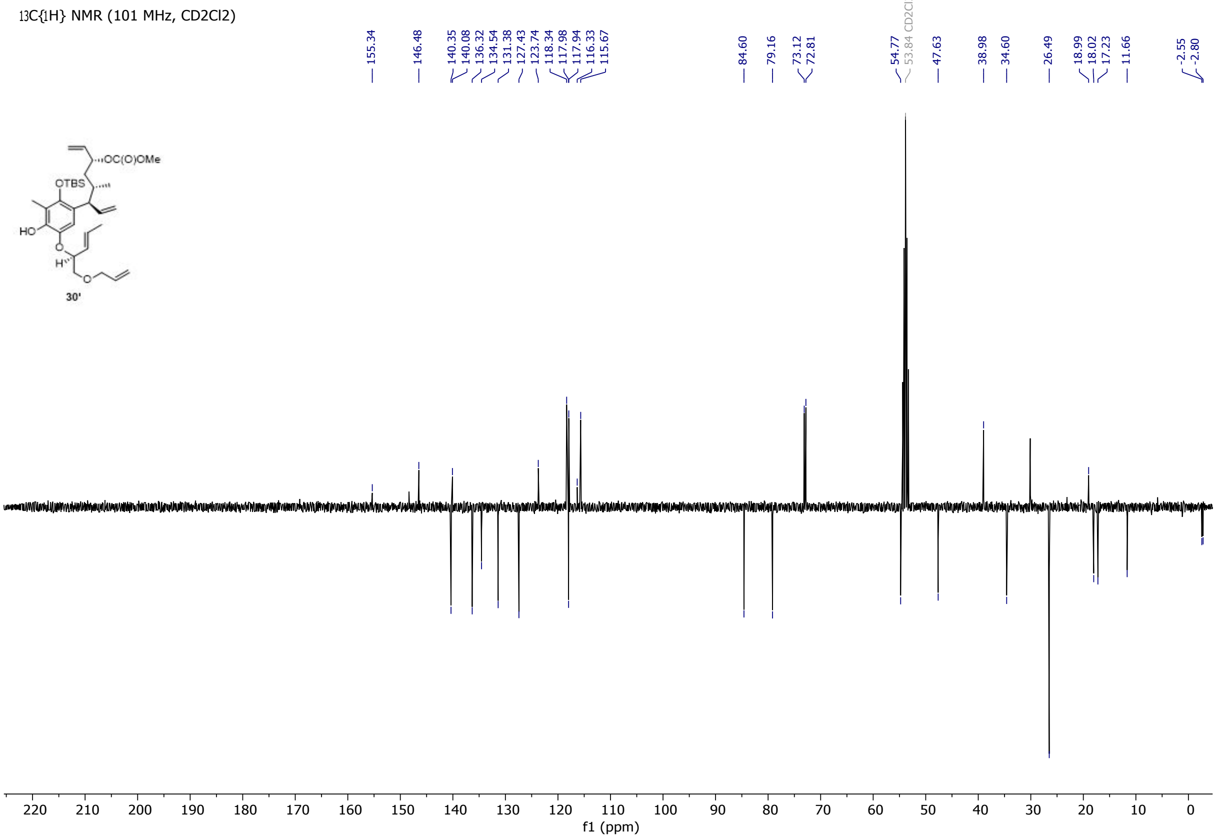

47. (1*R*,2*S*,4*S*,5*R*)-9-(((*S*,*E*)-1-(Allyloxy)pent-3-en-2-yl)oxy)-6-((tert-butyldimethylsilyl)oxy)-2,7-dimethyl-1,4-divinylspiro[4.5]deca-6,9-dien-8-one (29a)

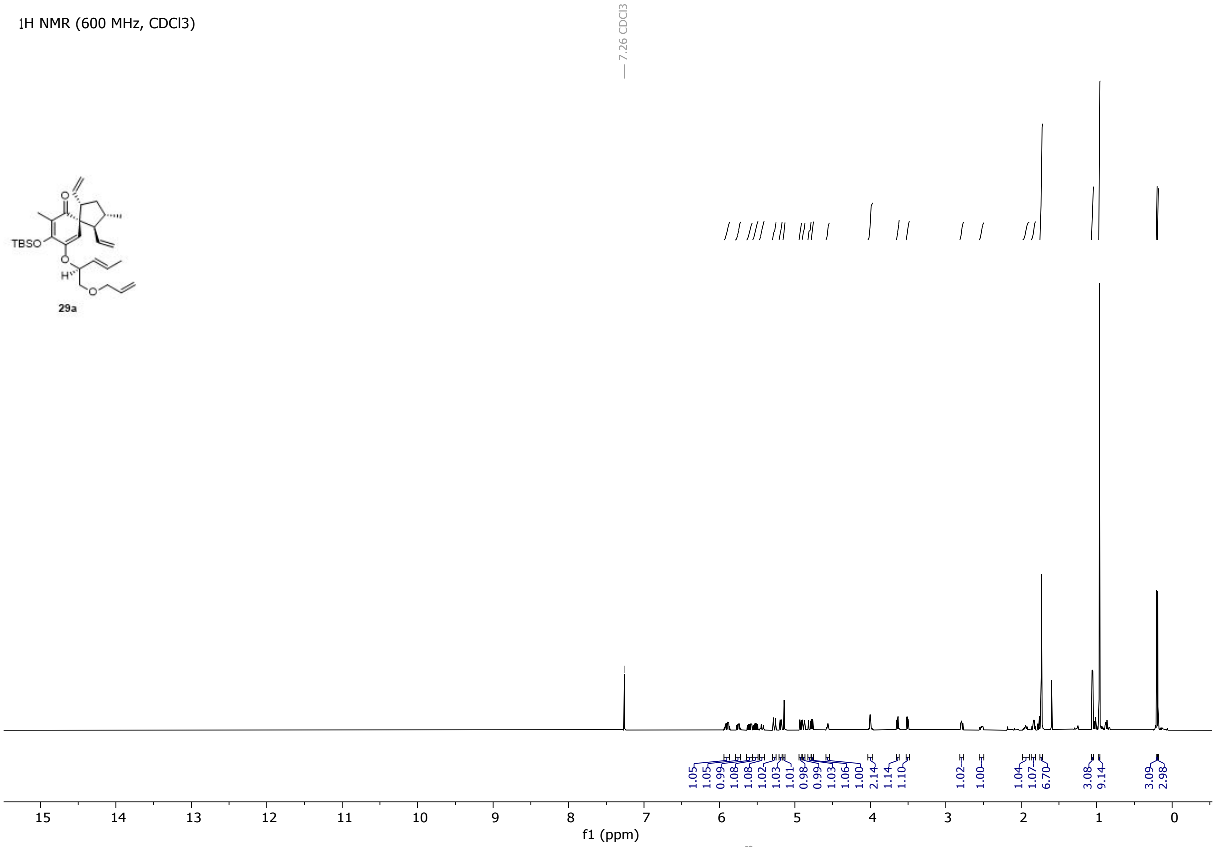

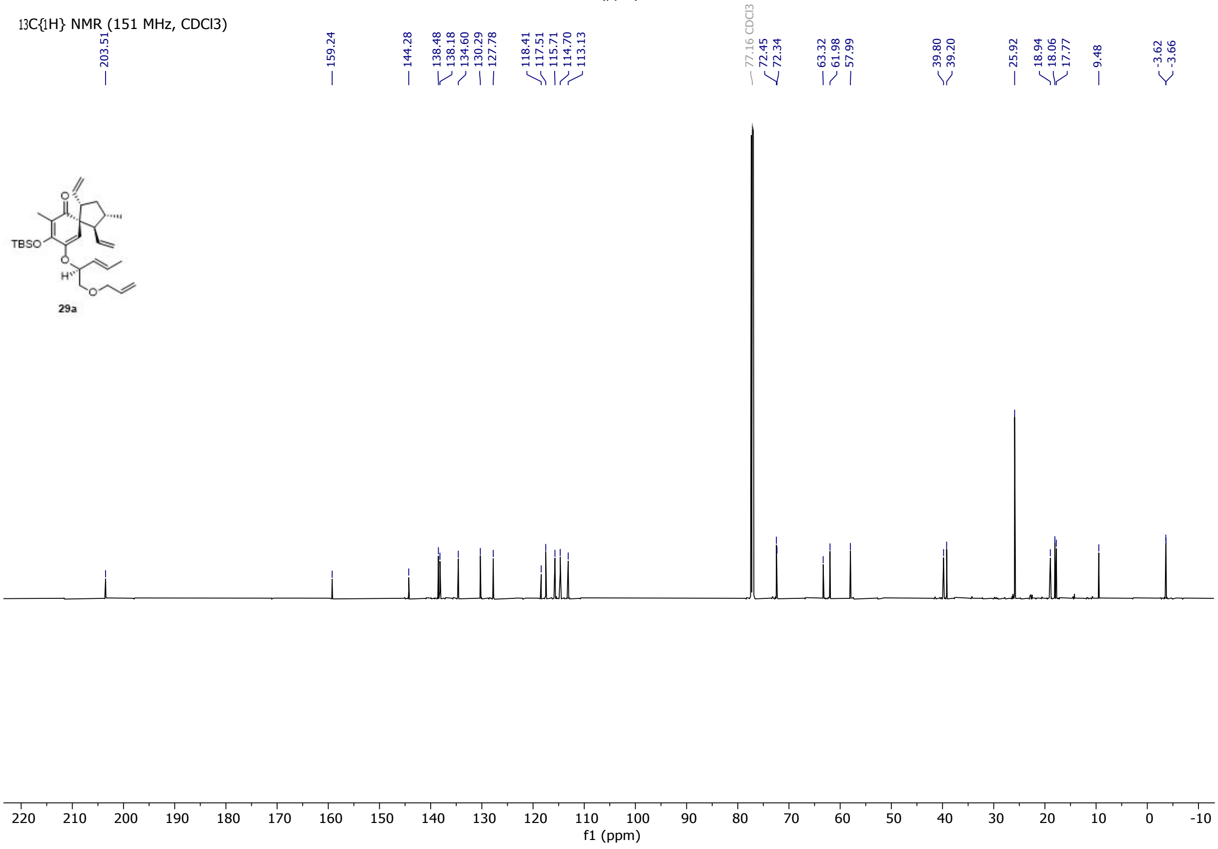

48. (3*R*,5*S*,6*R*)-6-(5-(((*S*,*E*)-1-(allyloxy)pent-3-en-2-yl)oxy)-2-((tert-butyldimethylsilyl)oxy)-3-methyl-4-((triethylsilyl)oxy)phenyl)-5-methylocta-1,7-dien-3-yl 4-nitrobenzoate (41)

1H NMR (400 MHz, CDCl3)

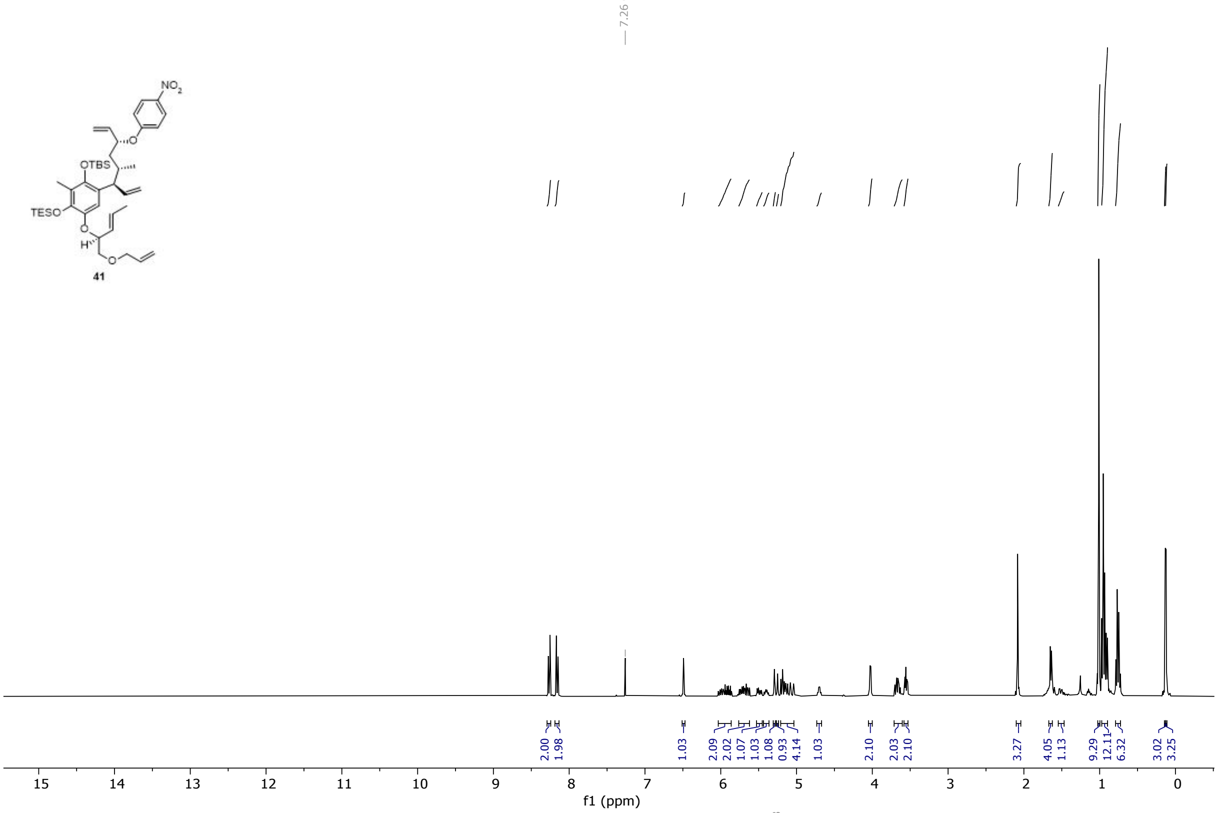

13C{1H} NMR (101 MHz, CDCl3)

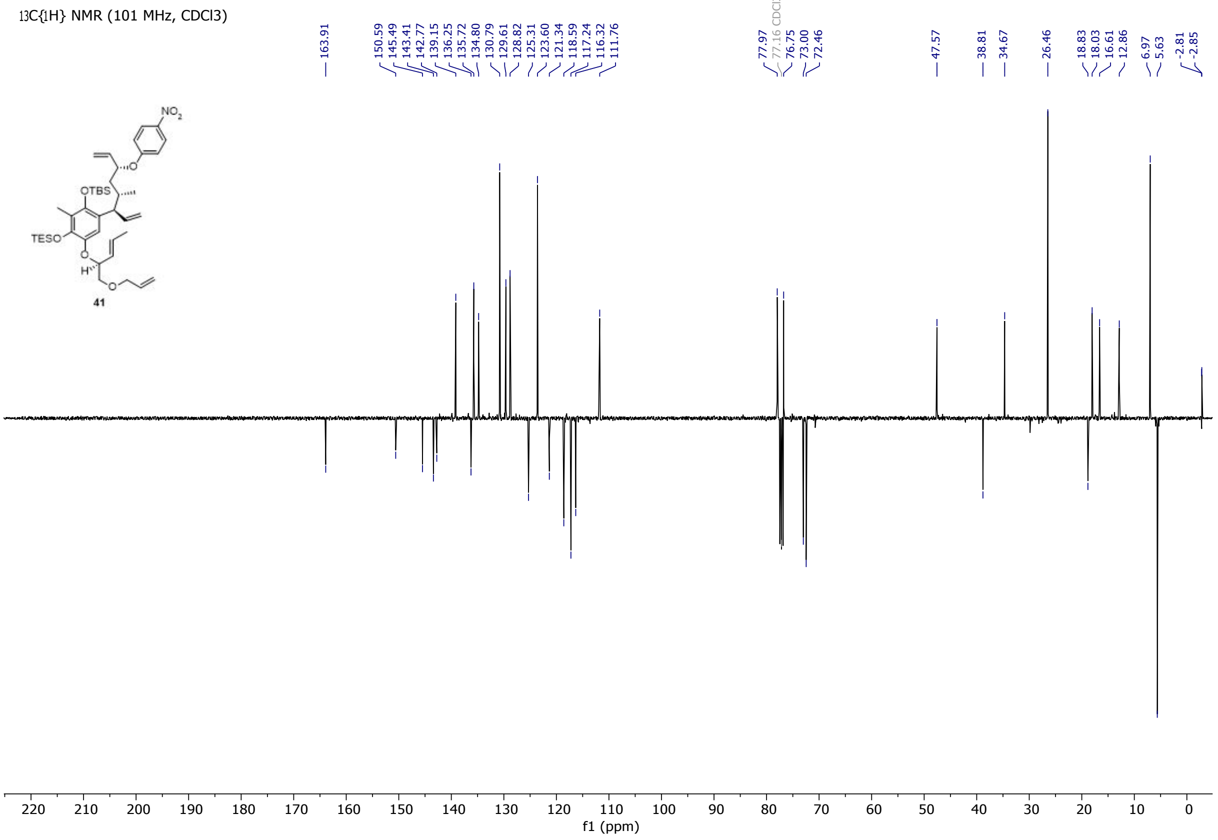

49. 6-(((*S*,*E*)-1-(allyloxy)pent-3-en-2-yl)oxy)-3-((tert-butyldimethylsilyl)oxy)-4-((3*R*,4*S*,6*R*)-6-hydroxy-4-methylocta-1,7-dien-3-yl)-2-methylphenol (42)

1H NMR (600 MHz, CDCl3)

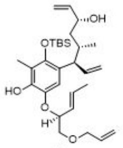

42

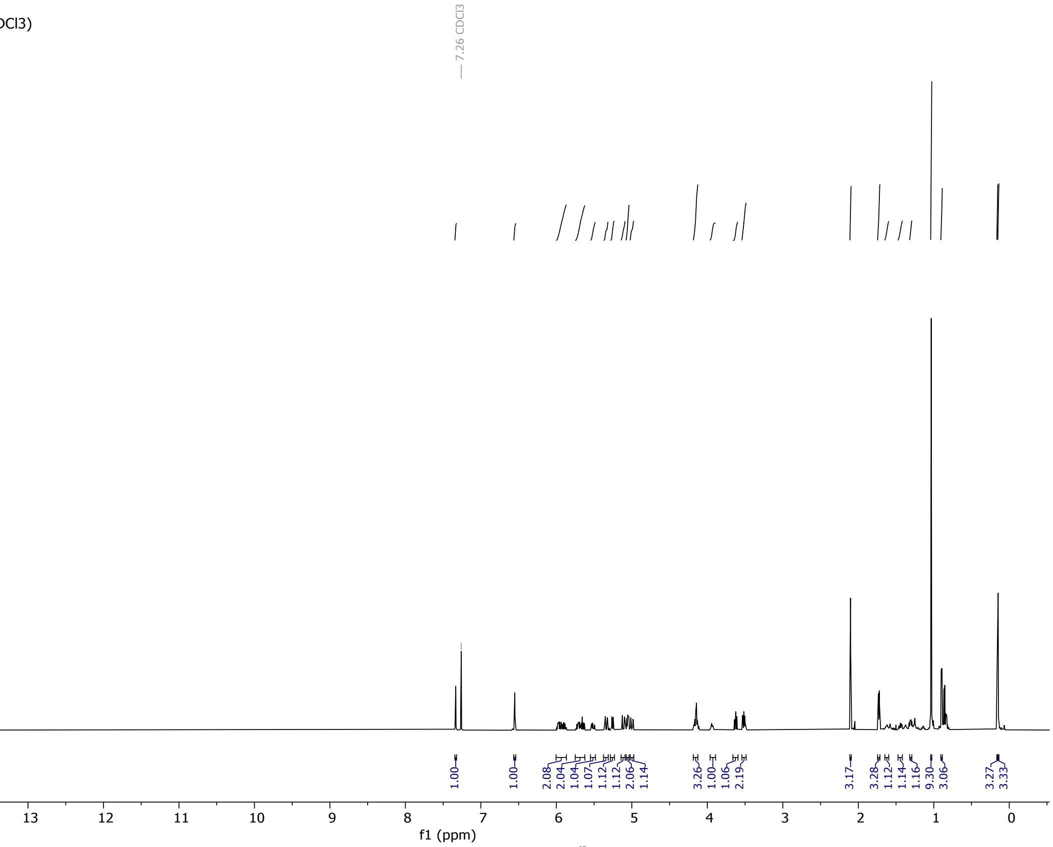

13C{1H} NMR (151 MHz, CDCl3)

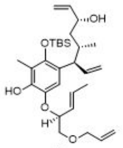

42

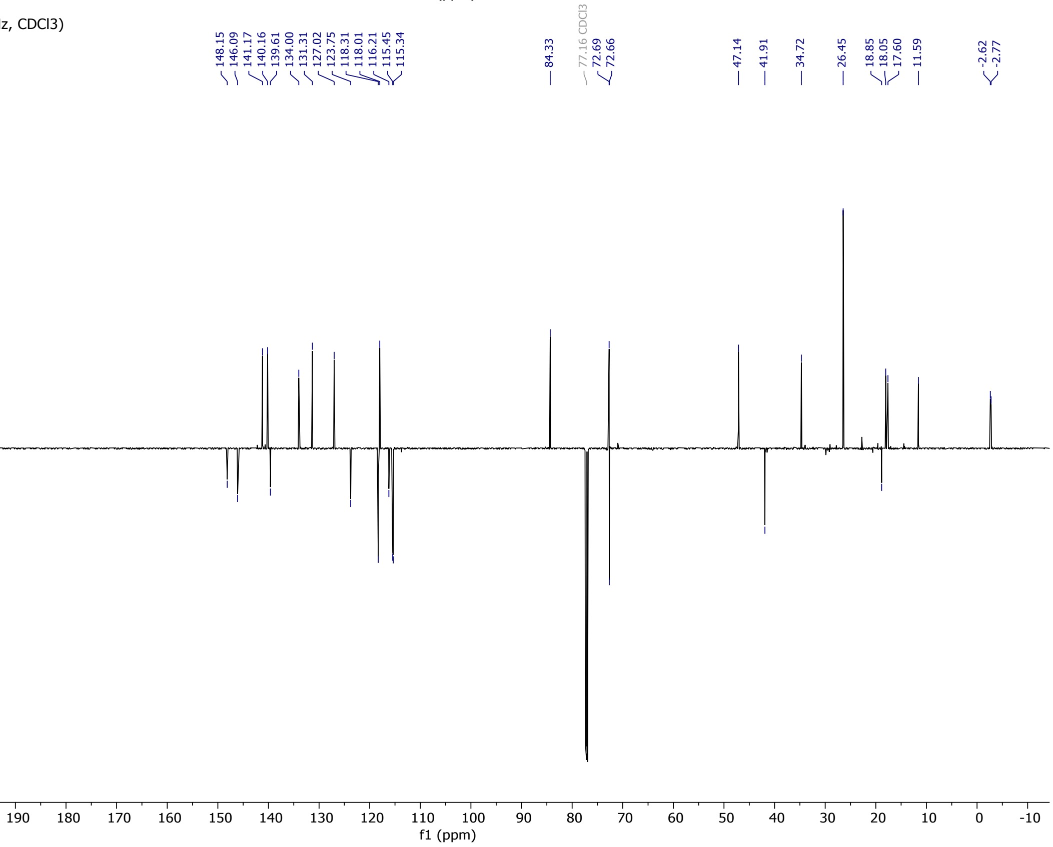

50. (1*R*,2*S*,4*S*,5*R*)-9-(((*S*,*E*)-1-(allyloxy)pent-3-en-2-yl)oxy)-8-hydroxy-2,7-dimethyl-1,4-divinylspiro[4.5]deca-7,9-dien-6-one (43)

^{1}H NMR (600 MHz, CDCl3)

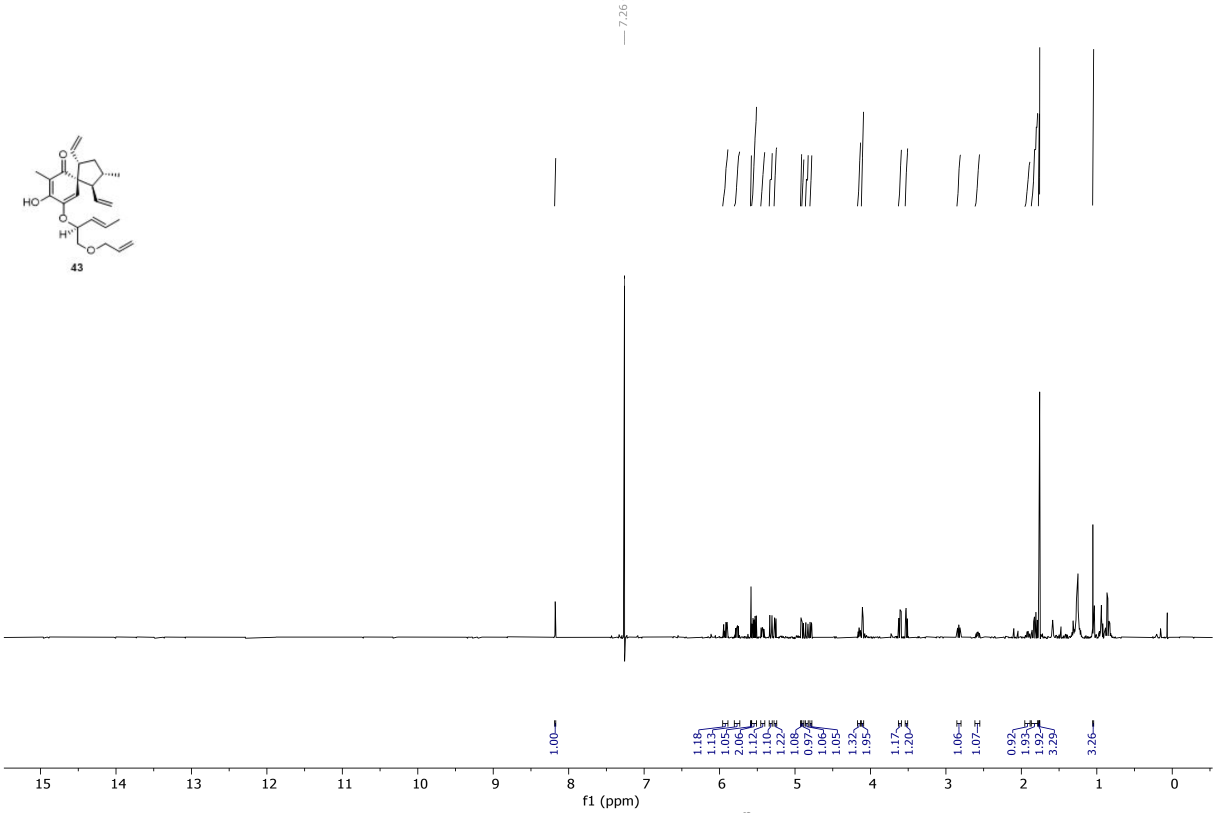

^{13}C{^{1}H} NMR (151 MHz, CDCl3)

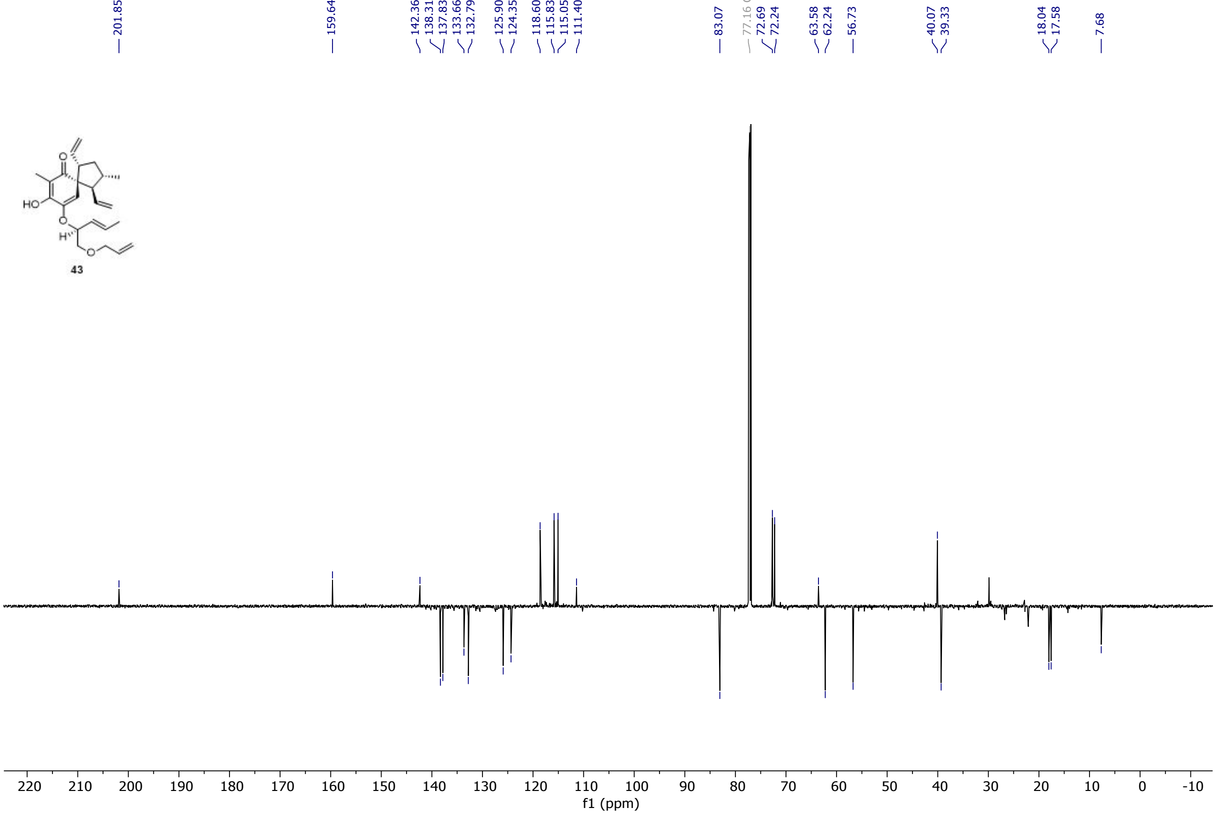

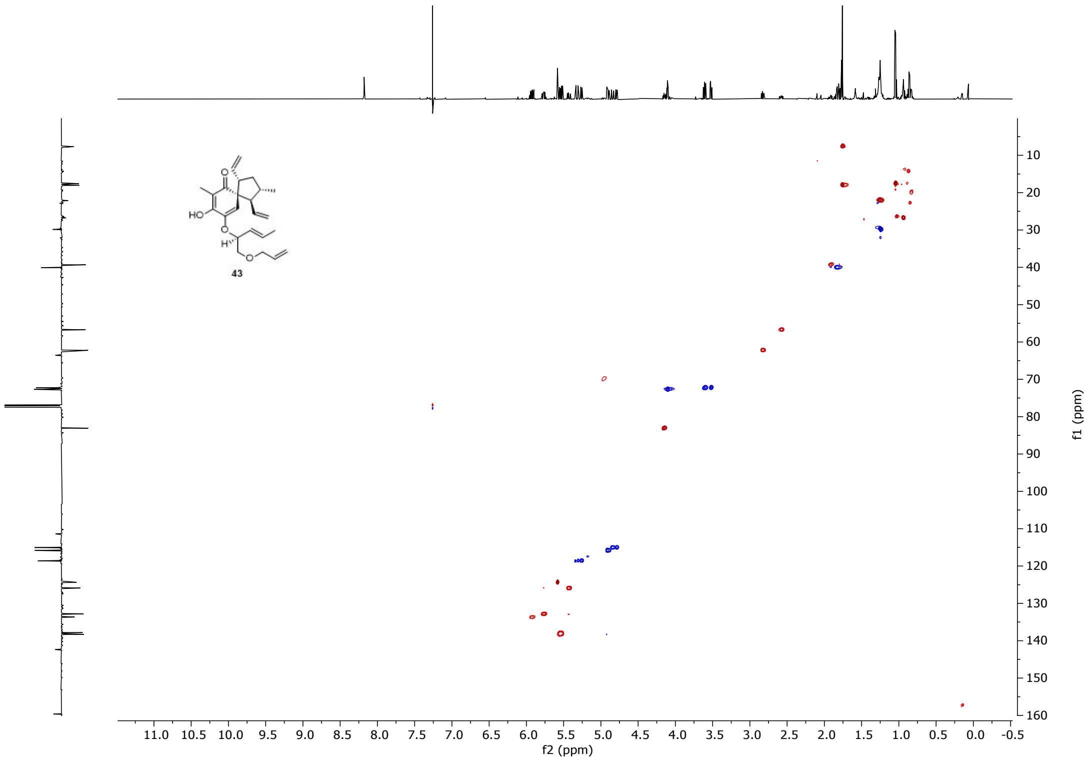

43
f2 (ppm)
f1 (ppm)

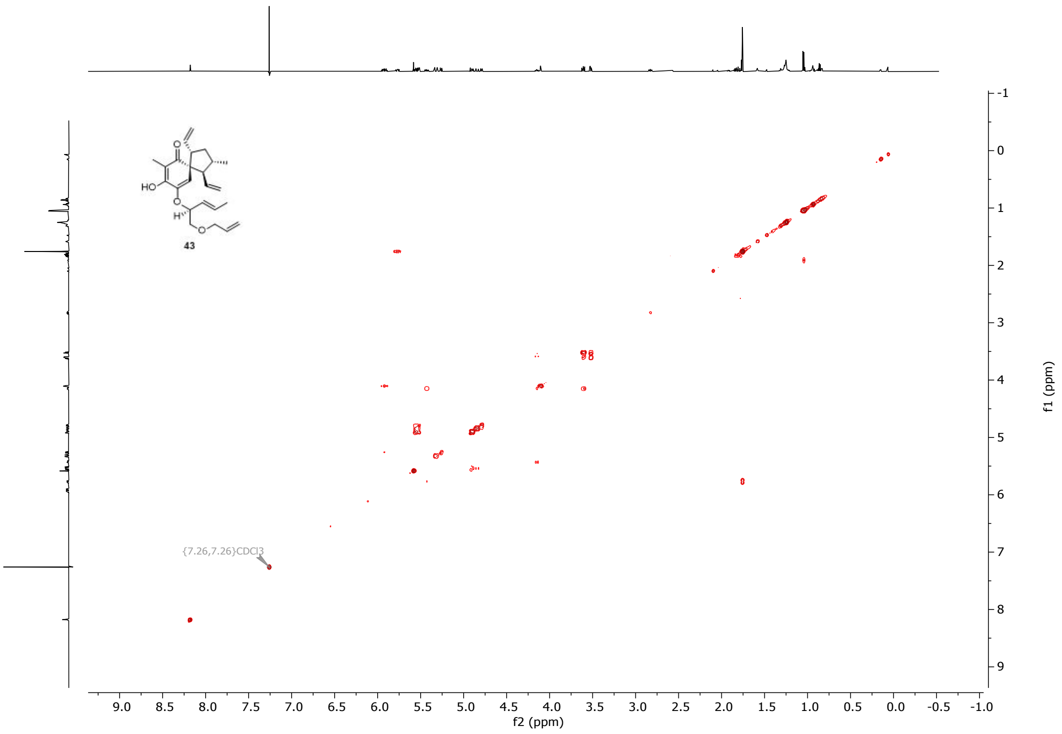

43
{7.26,7.26}CDCl3
f2 (ppm)
f1 (ppm)

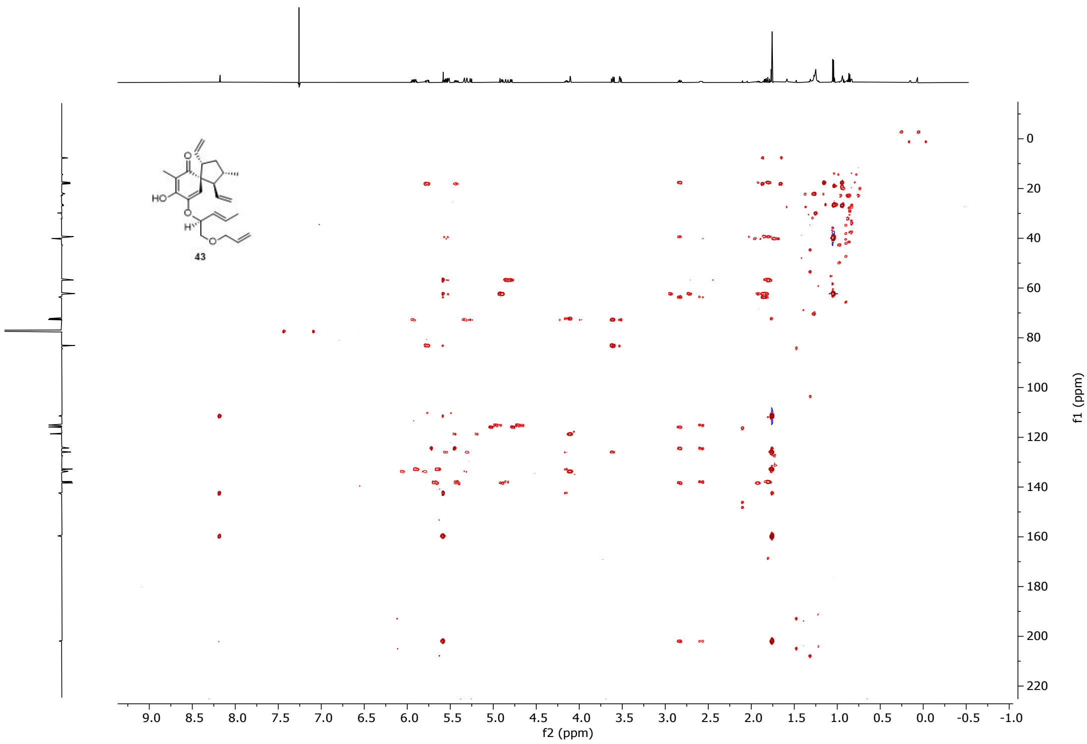

HO
H
43
f2 (ppm)
f1 (ppm)

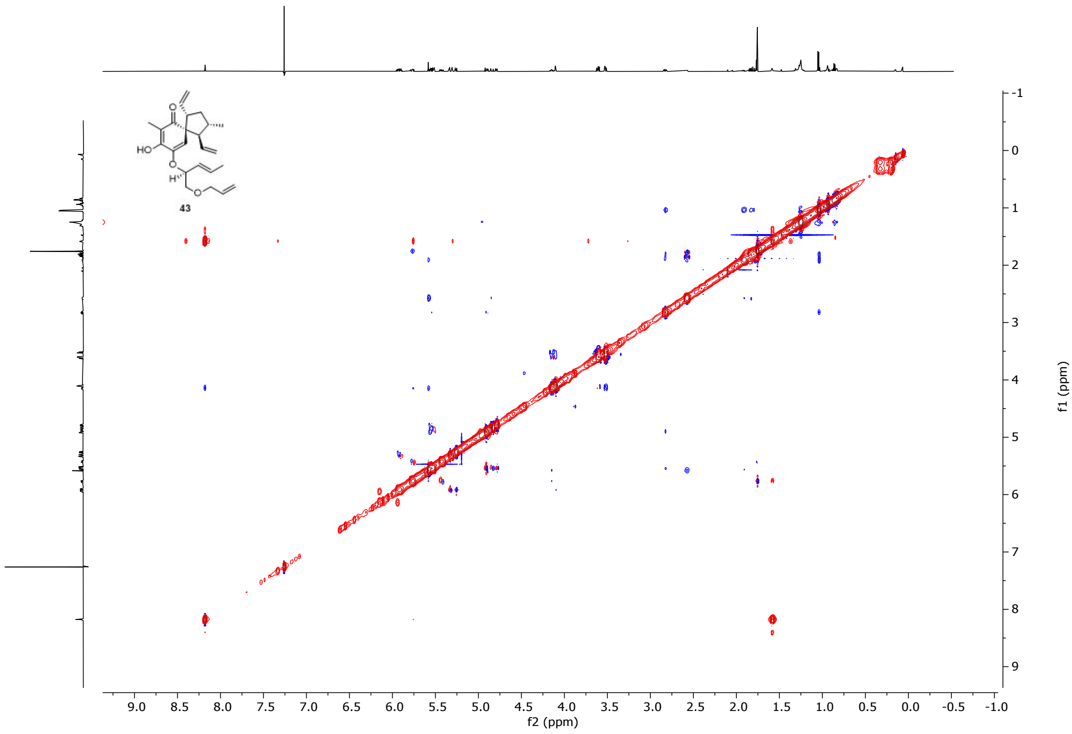

HO
H
43
f2 (ppm)
f1 (ppm)

Structure determination by single-crystal X-ray diffraction

Experimental

Suitable single crystals were preselected under a polarizing microscope, embedded in perfluorinated polyether and mounted on MiTeGen® loops. The single crystal X-ray diffraction measurements were performed on a Bruker-AXS APEXII four-circle diffractometer equipped with a CCD camera. Intensity data were collected at –173 °C (–123 °C) using graphite monochromatized Mo *K*α radiation (λ = 0.71073 Å). Correction for absorption effects were carried out with the multi-scan approach of SADABS [1]. Each crystal structure was solved by using Direct Methods and was refined by the full-matrix least-squares technique on F^2 with the SHELXTL program package [2]. H atoms were positioned geometrically (C–H = 0.95-1.00 Å) and were refined as riding with U_{iso}(H) = 1.2U_{eq}(C) for aromatic and methine H atoms, and with U_{iso}(H) = 1.5U_{eq}(C) for methyl H atoms.

Deposition numbers 2161381 (**S7**), 2161382 (**9**), 2161383 (**7epi8**) and 2161384 (**36**) contain the supplementary crystallographic data for this paper. These data are provided free of charge by the joint Cambridge Crystallographic Data Centre and Fachinformationszentrum Karlsruhe Access Structures service at https://www.ccdc.cam.ac.uk/structures/?.

Single crystals of compounds **S7**, **9**, **7epi-8** and **36** were grown by dissolution in **dichloromethane** (DCM, CH_2Cl_2) and subsequent slow evaporation at **22 °C**.

Table 1. Crystal data and details of structure refinements.

	**S7**	**9**	**7-epi-8**	**36**
Formula	$C_{22}H_{28}O_5$	$C_{33}H_{58}O_3Si_2$	$C_{36}H_{64}O_5Si_2$	$C_{24}H_{38}O_4Si$
M_r	372.44	558.97	633.05	418.63
Crystal system, space group	Orthorhombic, $P2_12_12$	Monoclinic, $P2_1$	Monoclinic, $P2_1$	Monoclinic, $P2_1/c$
Temp (°C)	–123 °C	–173 °C	–173 °C	–173 °C
a, *b*, *c* (Å)	19.789 (8), 8.663 (4), 12.349 (5)	15.1627 (15), 12.4327 (12), 18.4602 (19)	8.8721 (7), 19.1511 (16), 11.3472 (10)	23.973 (2), 12.3616 (13), 8.0781 (8)
α, *β*, *γ* (°)	90, 90, 90	90, 91.985 (3), 90	90, 94.999 (2), 90	90, 97.942 (4), 90
V (Å^3)	2117.1 (15)	3477.9 (6)	1920.7 (3)	2370.9 (4)
Z	4	4	2	4
Radiation	Mo *K*α	Mo *K*α	Mo *K*α	Mo *K*α
μ (mm^{-1})	0.08	0.13	0.13	0.13
Cryst. size (mm)	0.25 × 0.08 × 0.02	0.45 × 0.40 × 0.18	0.20 × 0.20 × 0.12	0.25× 0.15 × 0.09
T_{min}, T_{max}	0.582, 0.745	0.578, 0.746	0.646, 0.745	0.678, 0.746
No. of meas., indepen. obs. [$I > 2\sigma(I)$] reflections	26916, 2792, 2030	134427, 16114, 14691	57662, 7739, 7109	67111, 5771, 4725
R_{int}	0.136	0.058	0.047	0.074
θ_{max} (°)	22.6	27.7	26.4	28.2
$(\sin \theta/\lambda)_{max}$ (Å^{-1})	0.541	0.654	0.625	0.664
Refinement				
$R[F^2 > 2\sigma(F^2)]$, $wR(F^2)$, S	0.053, 0.133, 1.05	0.059, 0.140, 1.10	0.043, 0.095, 1.06	0.073, 0.159, 1.20
No. of reflections	2792	16114	7739	5767
No. of parameters	276	724	405	271
No. of restraints	21	1	1	0
H-atom treatment	H-atom parameters constrained	H-atom parameters constrained	H-atom parameters constrained	H-atom parameters constrained
$\Delta\rho_{max}$, $\Delta\rho_{min}$ (e Å^{-3})	0.29, −0.29	0.53, −0.35	,	0.46, −0.37
Absolute structure	Flack x determined using 697 quotients [(I+)-(I-)]/[(I+)+(I-)] (Parsons, Flack and Wagner, Acta Cryst. B69 (2013) 249-259).	Flack x determined using 6265 quotients [(I+)-(I-)]/[(I+)+(I-)] (Parsons, Flack and Wagner, Acta Cryst. B69 (2013) 249-259).	Flack x determined using 3096 quotients [(I+)-(I-)]/[(I+)+(I-)] (Parsons, Flack and Wagner, Acta Cryst. B69 (2013) 249-259).	–
Absolute structure parameter	−1.2 (10)	−0.01 (4)	−0.10 (5)	–

Figure. 1. Molecular structure of S7 with displacement ellipsoids drawn at the 50% probability level.

S7

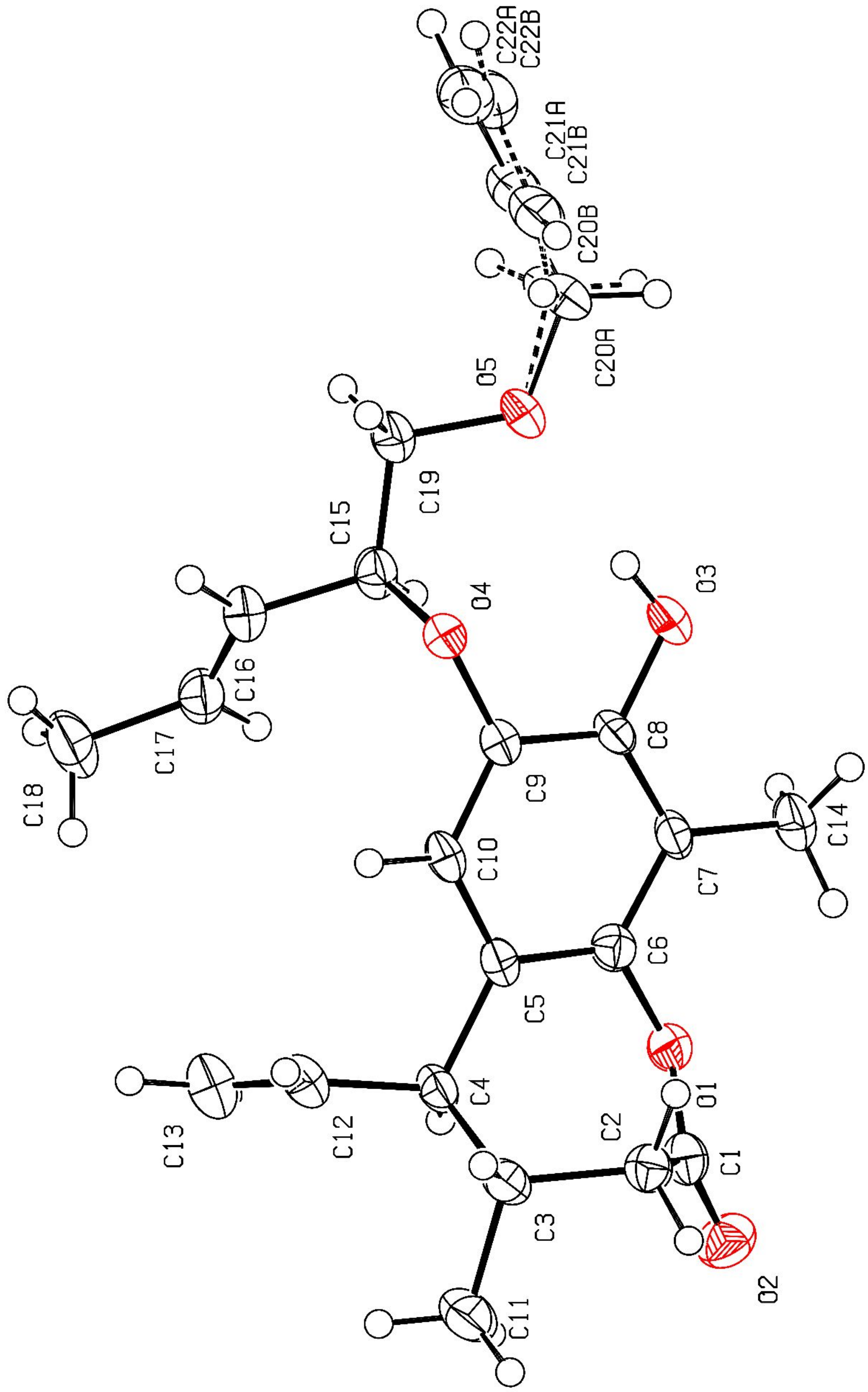

Figure 2. Molecular structures of the two independent molecules of 9 with displacement ellipsoids drawn at the 50% probability level.

9

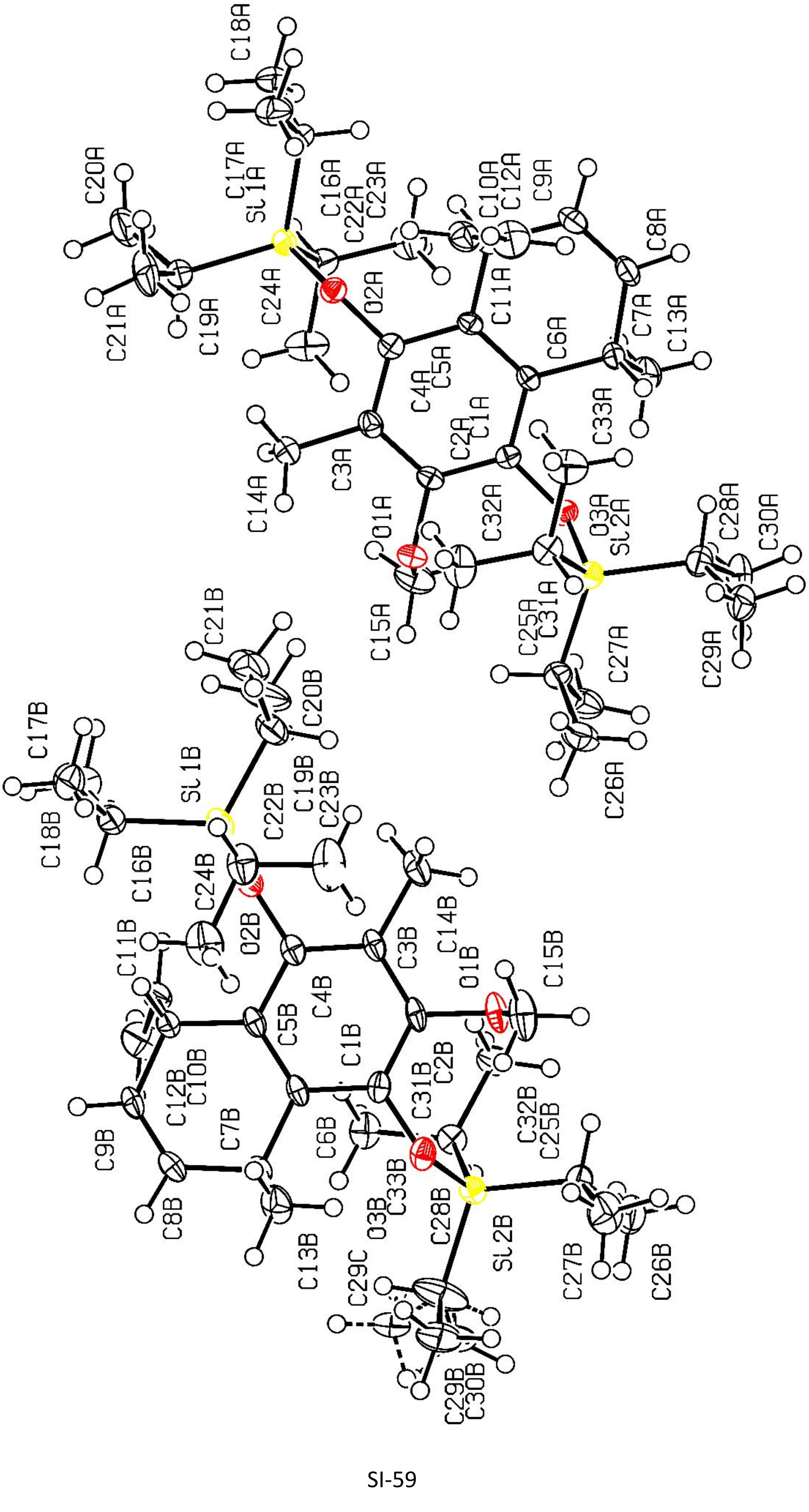

Figure 3. Molecular structure of 7-epi-8 with displacement ellipsoids drawn at the 50% probability level.

7-epi-8

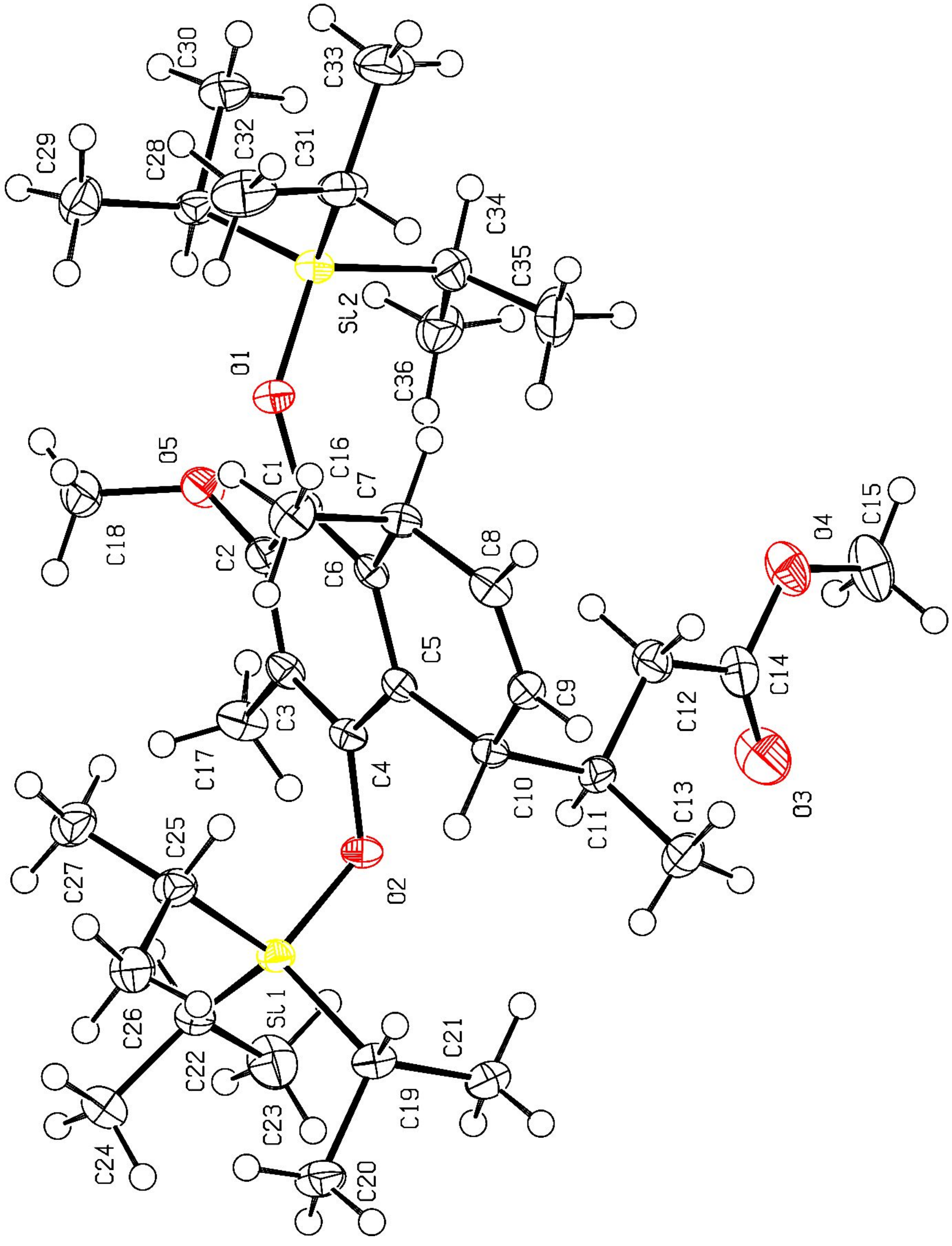

Figure 4. Molecular structure of 36 with displacement ellipsoids drawn at the 50% probability level.

36

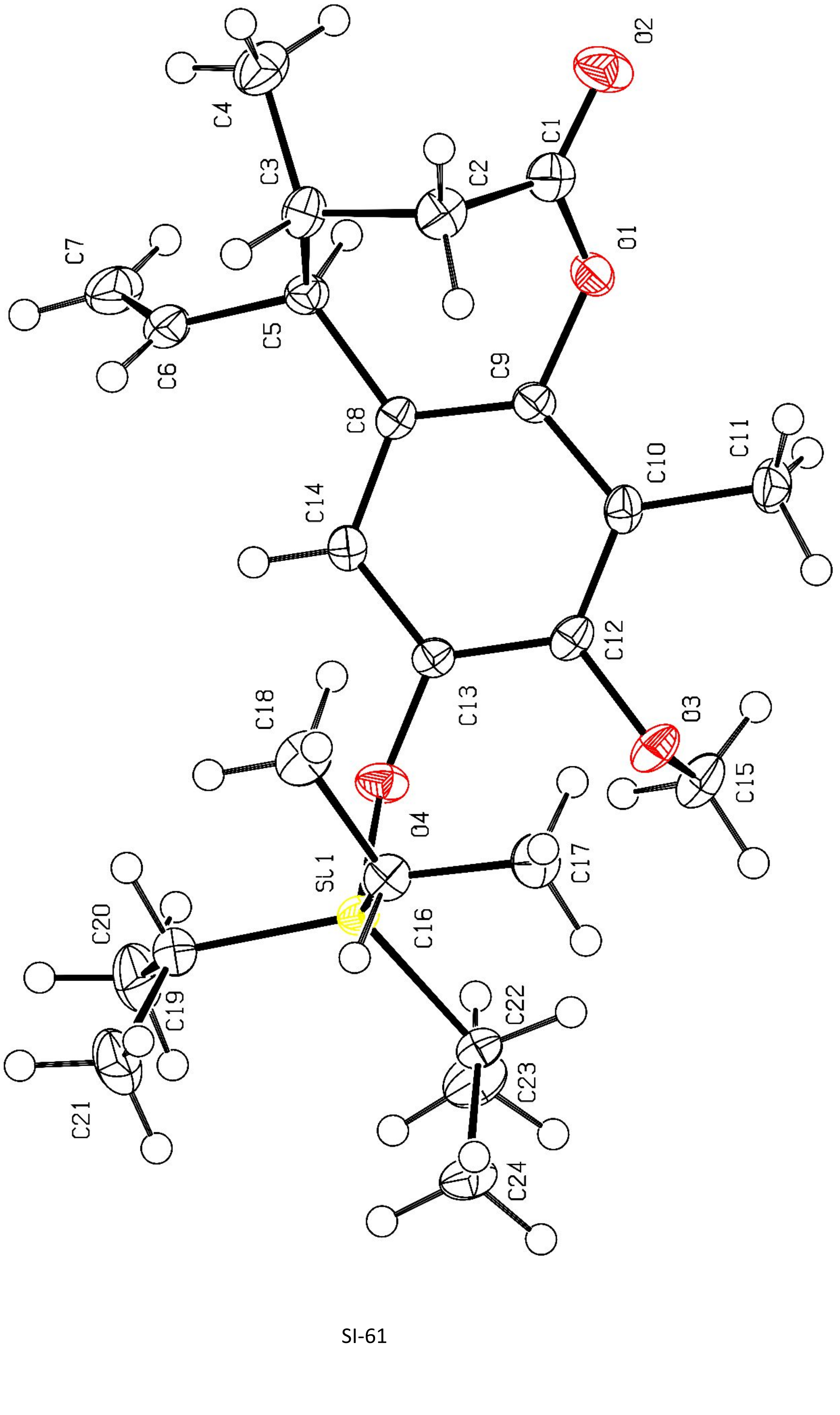

Supplement: Supplementary file 1 — jo2c01914_si_001.pdf [file jo2c01914_si_001.pdf]
